# Unravelling the Mechanism and Governing Factors in Lewis Acid and Non-Covalent Diels–Alder Catalysis: Different Perspectives

**DOI:** 10.3390/ijms24054938

**Published:** 2023-03-03

**Authors:** Lise Vermeersch, Frank De Proft, Vicky Faulkner, Freija De Vleeschouwer

**Affiliations:** Research Group of General Chemistry (ALGC), Vrije Universiteit Brussel (VUB), Pleinlaan 2, B-1050 Brussels, Belgium

**Keywords:** Diels–Alder catalysis, Lewis acids, non-covalent interaction donors, activation strain model, energy decomposition analysis

## Abstract

In the current literature, many non-covalent interaction (NCI) donors have been proposed that can potentially catalyze Diels-Alder (DA) reactions. In this study, a detailed analysis of the governing factors in Lewis acid and non-covalent catalysis of three types of DA reactions was carried out, for which we selected a set of hydrogen-, halogen-, chalcogen-, and pnictogen-bond donors. We found that the more stable the NCI donor–dienophile complex, the larger the reduction in DA activation energy. We also showed that for active catalysts, a significant part of the stabilization was caused by orbital interactions, though electrostatic interactions dominated. Traditionally, DA catalysis was attributed to improved orbital interactions between the diene and dienophile. Recently, Vermeeren and co-workers applied the activation strain model (ASM) of reactivity, combined with the Ziegler-Rauk-type energy decomposition analysis (EDA), to catalyzed DA reactions in which energy contributions for the uncatalyzed and catalyzed reaction were compared at a consistent geometry. They concluded that reduced Pauli repulsion energy, and not enhanced orbital interaction energy, was responsible for the catalysis. However, when the degree of asynchronicity of the reaction is altered to a large extent, as is the case for our studied hetero-DA reactions, the ASM should be employed with caution. We therefore proposed an alternative and complementary approach, in which EDA values for the catalyzed transition-state geometry, with the catalyst present or deleted, can be compared one to one, directly measuring the effect of the catalyst on the physical factors governing the DA catalysis. We discovered that enhanced orbital interactions are often the main driver for catalysis and that Pauli repulsion plays a varying role.

## 1. Introduction

**Scheme 1:**
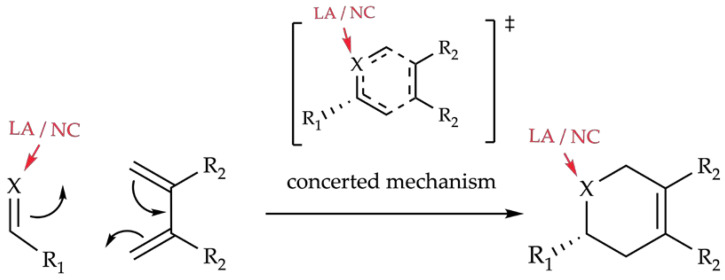
Schematic representation for the *endo* hetero-Diels–Alder reaction between a catalyzed dienophile and a diene. LA denotes Lewis acid, NC is for non-covalent interaction donor, ‡ indicates the transition state.

Due to its versatility, the Diels–Alder (DA) reactions are one of the most widely used chemical reactions today. These reactions have become the best practice for the creation of regio-, stereo-, and enantioselective six-membered rings [1], which explains the high interest in increasing the rate and selectivity of these reactions. Besides their applicability in organic synthesis and natural product synthesis, the reversibility of these cycloaddition reactions makes them interesting candidates for use in macromolecular synthesis, e.g., offering the possibility to recycle or repair polymer network materials. Their many applications highlight the need to better understand what influences the kinetics [2]. In this work, the influence of non-covalently bonded catalysts on the DA kinetics was analyzed (see Scheme 1).

It is widely known that Lewis acids, such as AlCl_3_ [3], BF_3_, TiCl_4_ [4], EtAlCl_2_, and many more [1], can catalyze the DA reaction by binding to the dienophile [5]. Furthermore, in the past decade, much research focused on how non-covalent interactions can stabilize the transition state and thus lower the reaction barrier [6]. Diels–Alder catalysis through non-covalent interactions brought to light a whole range of possible catalysts. Hydrogen bonds in substituted thioureas, for example, were shown to have a large rate enhancement (1–30 times) for a set of Diels–Alder reactions [7]. For these systems, it was shown that the lower interaction compared to Lewis acids was actually an advantage, as it avoided product inhibition. Even when Diels–Alder reactions were performed in hydrogen-bonding solvents, catalysis was observed, and the relative yield of the reaction increased as well [8]. However, the effectiveness of a non-covalent catalyst depends more on the type of substituents rather than on the solvent used [7].

Non-covalent catalysis can also be obtained via interaction with the σ holes of halogen-, chalcogen-, and pnictogen-based catalysts [9]. The σ hole is a region of positive potential, found along the axis of a covalent bond between, for example, the chalcogen atom and the remaining structure. It was only very recently that these catalysts gained interest, and the exact driving force behind the catalysis has yet to be identified. The number of σ holes and their accessibility differ for halogens, chalcogens, and pnictogens, and their strength will increase when going from left to right in the periodic table [9]. Both computationally and experimentally [10,11,12,13], halogen catalysts of different nature were shown to accelerate DA reactions by lowering the activation barrier up to 15 kcal mol^−1^ [14]. The effectiveness of the catalyst was related to the strength of the formed non-covalent bond and increased going from Cl to I species [15]. Chalcogen catalysts were shown to increase the reaction rate of the DA reaction [16], reaching a factor well beyond 1000 for a reaction between quinolines and imines [17]. Computational studies predicted the pnictogen catalysts to be even more active than halogen and chalcogen catalysts [18,19] for DA reactions with α,β-unsaturated ketones, while the opposite was shown for 1,3 butadiene and methyl acrylate DA reactions [20].

Traditionally, the catalysis via Lewis acids was ascribed to the lowering of the LUMO level of the dienophile, increasing the orbital interactions between the diene and dienophile [21]. In 2008, conceptual density functional theory was used on the Lewis acid catalyzed isoprene–acrolein DA reaction. The authors found that the Lewis acids significantly decreased the LUMO energy of acrolein and increased the dienophile’s electrophilicity, yielding a good linear correlation with the activation energies [22]. In more recent studies, however, Bickelhaupt and co-workers have shown that the catalysis may be caused by the reduction in Pauli repulsion of the π-molecular orbitals [23], rather than improved orbital interactions. These findings were based on the activation strain model (ASM), which allows differentiating between the strain energy ∆E_strain_(ζ) and the interaction energy ∆E_int_(ζ), both defined along the reaction coordinate ζ. The interaction energy can then be further decomposed into contributions of Pauli repulsion energy, electrostatic energy, orbital energy, and dispersion energy using a Ziegler-Rauk-type energy decomposition analysis (EDA). The authors demonstrated that the HOMO(diene)–LUMO(dienophile) interaction is indeed more favorable for a Lewis acid-catalyzed DA reaction. However, the HOMO(dienophile)–LUMO(diene) interaction is weakened to a similar degree, making the total orbital interactions of the catalyzed and uncatalyzed reactions comparable in magnitude. They also observed a clear difference in strain energy between the uncatalyzed and catalyzed reactions, with less strain for the latter, which they ascribed to an increasing asynchronicity of the DA reaction. In 2021, Vermeeren et al. showed that electronic reaction barriers of the DA reaction between cyclopentadiene and acroleine could be lowered by up to 7 kcal mol^−1^ using bifunctional hydrogen-bond organocatalysts, again due to a lowering of the Pauli repulsion between the closed-shell filled π orbitals of the diene and dienophile [24]. In addition, a shift from endo to exo selectivity was seen upon catalysis, which could be connected to a diminished strain energy for the latter caused by increased asynchronicity of the exo DA reaction. This was confirmed in a study performed by Yepes et al. on the effect of the bulkiness of the Lewis acid catalyst on the exo selectivity of DA reactions with acyclic dienes. Exo selectivity was the result of increased electrostatics and dispersion interaction energies, next to reduced strain energy [25]. Another study by Portela et al. reported similar findings for bidentate iodine-based halogen donors, as their quantitative analysis using ASM showed that the catalysis also originates from a reduction in Pauli repulsion between the diene and dienophile [26].

The goal of this study is to extend the novel findings by Bickelhaupt and co-workers for various types of non-covalent Lewis acid-like organocatalysts in combination with yet unexplored DA reactions since the magnitude of these non-covalent interaction energies can have different origins and the type of DA reaction may have a significant effect as well. The effectiveness of the catalysts will be examined based on the Gibbs free energies of activation. Furthermore, the mechanism behind the catalysis will be scrutinized using different computational techniques and research problem perspectives. In the ASM approach, potentially governing physical factors between the uncatalyzed and catalyzed reactions are evaluated along a reaction energy profile. This is completed using a suitable projection of the reaction coordinate, for example, the shorter of the two newly forming DA bonds. In our alternative perspective, focus is put on the transition-state geometry for the catalyzed reaction, and one tries to find an answer to the following research question: which physical factors govern the catalytic effect in the newly adopted transition-state geometry?

**Scheme 2:**
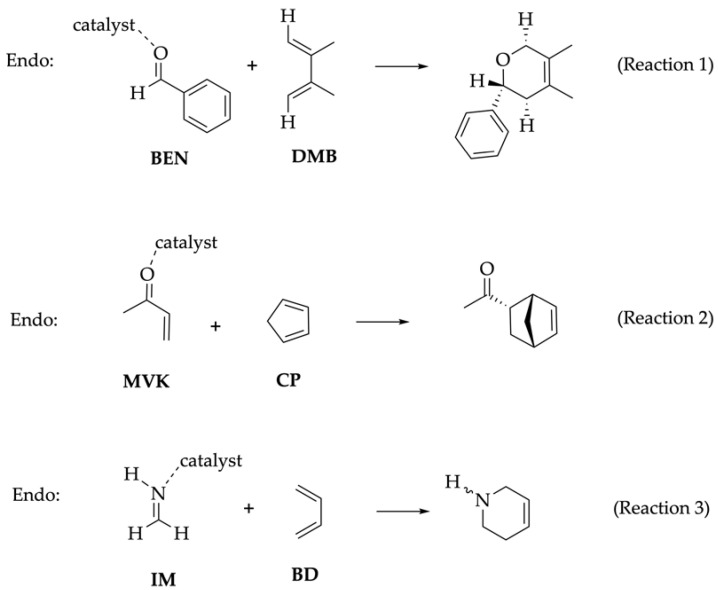
Three (hetero-)Diels–Alder reactions considered for this study, with indication of the catalyst’s position.

Three different DA reactions were studied in the gas phase (Scheme 2), a (hetero-)DA reaction between benzaldehyde (BEN) as a dienophile and 2,3-dimethyl 1,3-butadiene (DMB) as a diene (reaction 1), between methyl vinyl ketone (MVK) and cyclopentadiene (CP) (reaction 2), and between methylene imine (IM) and 1,3-butadiene (BD) (reaction 3), all for endo adduct formation. Aside from reaction 2, the analyzed hetero-DA reactions in this work are unalike from those studied by Vermeeren et al. [23,24] since the geometry of the catalyzed and uncatalyzed transition states is very different, as will be discussed vide infra.

**Figure 1:**
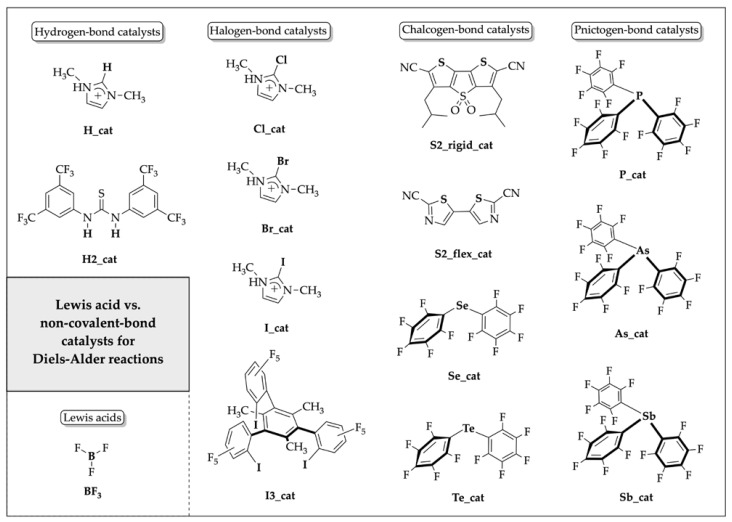
Variety of Lewis acid and non-covalent Lewis acid-like catalysts considered in this study.

The used catalysts are shown in Figure 1. For all three reactions, we considered 13 catalysts: (I) a classical Lewis acid, BF_3_; (II) two hydrogen-bond catalysts, the positively charged imidazolium (H_cat) [15] and a neutral bidentate hydrogen-bond donor (H2_cat) [7]; (III) three imidazolium-substituted catalysts [12,15] with the halogens chlorine (Cl_cat), bromine (Br_cat), and iodine (I_cat); (IV) four chalcogen-bond donors, of which two are the bidentate sulphur-donor catalysts [17] S2_rigid_cat and S2_flex_cat, which display a difference in the rigidity of the molecular framework, as well as the monodentate Se_cat and Te_cat [17], both substituted with electron-withdrawing pentafluorophenyl groups; and finally, (V) three pnictogen-bonding catalysts [17] that show a variation in pnictogen atoms, going from phosphorus (P_cat) over arsenic (As_cat) to antimony (Sb_cat) in order to establish a possible trend in strength when moving down in the periodic table. In addition, for reaction 2 between MVK and CP, we included a bulky but neutral halogen-bond catalyst involving three iodine atoms that could potentially form three halogen bonds with the dienophile (I3_cat) [14]. All the mentioned non-covalent-bonding donors have been reported in the literature as possessing (potential) catalytic activity. Thanks to this wide range of different types of interactions, a more detailed analysis of the influence of the non-covalent bonding strength on the possible acceleration of the Diels–Alder reaction rates can be conducted. However, in this study, we restrict ourselves to gas-phase reactions whereas, experimentally, most reactions occur in solution.

## 2. Results

### 2.1. Complex Stability

At first, the stability of the initially formed complex between the dienophile (R1) and each of the catalysts (cat) is evaluated (Table 1) for the Diels–Alder reaction between benzaldehyde and 2,3-dimethyl 1,3-butadiene. The results for the other two reactions are reported in the Supplementary Materials. The type of non-covalent interaction may play a role on how the dienophile’s intrinsic reactivity is influenced, which in turn can affect the DA reaction kinetics when combined with the diene.

At 298.15 K, only the complexes with the Lewis acid, both hydrogen-bond catalysts, the iodine catalyst, and the antimony catalyst are found to be stable, with the positively charged hydrogen-bond complex R1_H_cat and iodine-bond complex R1_I_cat clearly reflecting the strongest interactions. At 0 K, however, H2_cat forms the most stable complex due to its bidentate functionality, which is entropically more penalized at 25 °C. In addition, the two chalcogen-bond donor atoms present in S2_rigid_cat and S2_flex_cat yield more stable complexes than the larger chalcogen atom complexes, despite the less pronounced σ holes in the former. For a series of similar catalysts, e.g., Cl/Br/I_cat, Se/Te_cat, or P/As/Pb_cat, the interaction between benzaldehyde’s oxygen and the Lewis acid-like donor atom strengthens when moving down in the periodic table, in agreement with earlier observations in the literature [27,28]. Similar conclusions can be drawn for the complexes with methyl vinyl ketone and methylene imine (Tables S1 and S2 in the Supplementary Materials).

**Figure 2:**
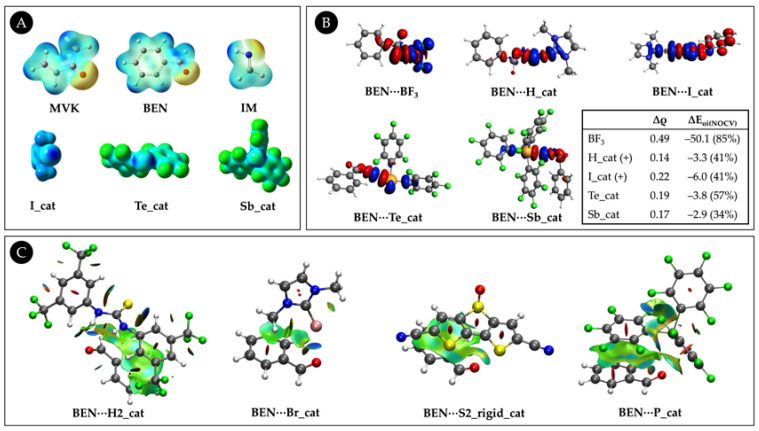
(**A**) Molecular electrostatic potential plots of the dienophiles and three catalysts taken at an electron density isovalue of 0.01 a.u. and with yellow–red colors denoting electron-rich regions and blue denoting electron-poor regions. (**B**) NOCVs for the catalyst-benzaldehyde complexes with corresponding ∆E_oi(NOCV)_ in kcal mol^−1^ and ∆ρ values in a.u. for an isovalue of 0.0005. The values between brackets for the ∆E_oi(NOCV)_ correspond to their relative contribution to the total orbital energies. Electron depletion is indicated in red, whereas electron accumulation is indicated in blue. (**C**) Visualization of the non-covalent interaction index (NCI) of benzaldehyde and four catalysts. Green indicates π-π and Van der Waals interactions, blue strong non-covalent bonds and red repulsive interactions such as ring strain.

This trend in more stabilizing interactions when moving down in the periodic table for pnictogen-, chalcogen-, and halogen-bonded systems is often connected to the presence of a more positively charged σ hole that can electrostatically interact with the high electron density in the oxygen or nitrogen of the Lewis base, as can be witnessed from the molecular electrostatic potential (MEP) plots in Figure 2A. The electrostatic potential is the most negative on the nitrogen of the methylene imine, but the active region is also the smallest. The figure also shows a comparison between a halogen, chalcogen, and pnictogen catalyst, for which the number of σ holes increases from 1 to 2 to even 3. This positive potential is largest for I_cat since the positive charge of the ring induces a significant electron-withdrawing effect. For all non-hydrogen-bonded systems, the largest electrostatic interactions are thus expected between the IM dienophile and I_cat.

Next to the often-important electrostatics, polarization, charge-transfer, dispersion interactions, and strain energy can also play a critical role. One way to quantitatively analyze their influence is to partition the complexation energy into additive contributions associated with the different stabilizing and destabilizing factors. In this study, we have used the Ziegler-Rauk-type energy decomposition analysis as already highlighted in the introduction. Table 2 lists the different energy components for the formation of the encounter complexes between benzaldehyde and the set of 13 potential catalysts, now based on the PBE/TZ2P electronic energies. In the Supplementary Materials, Tables S3 and S4 report the values for the other two reactions.

Except for BF_3_, the strain or deformation energy is limited. In the BF_3_–BEN complex, this can be fully attributed to the loss of planarity of BF_3_ upon formation of the bond with benzaldehyde. The stronger interaction in this complex, accompanied by a significantly shorter bond length compared to the non-covalent interactions, yields much enhanced values for the different energy components. Electrostatics and orbital interactions are of similar magnitude, which is contrary to the families of non-covalent catalysts. In terms of attractive interactions, the electrostatic interactions contribute the most, ranging from 45% to even 61% of the total stabilization, while the importance of the orbital interactions and dispersion interactions fluctuates for the different catalysts. For the positively charged catalysts, the electrostatic energies are strong enough to overcome the destabilizing Pauli repulsion, having a similar or larger magnitude than ∆E_Pauli_, which agrees with the MEP predictions. Of all non-covalent catalysts, the orbital interactions are found to be highest for I_cat, both quantitatively and qualitatively; however, they are still considerably less than in the case of BF_3_. Figure 2B depicts the charge density reorganization (∆ρ) upon complex formation through the relevant NOCV pairs, together with the associated orbital interaction energies. Only for the BF_3_ complex is a significant electron donation, moving from the lone pair of the carbonyl group (∆ρ < 0) to the catalyst (∆ρ > 0), observed, which contributes significantly to the stabilization of the complex (85% of total orbital interactions). For the non-covalently bound complexes, the NOCVs correspond to about half of the total orbital energies, indicating that density rearrangements within the fragments, i.e., polarization, are contributing as well. However, the typical depletion-accumulation pattern as seen for BF_3_–BEN is also visible for the other complexes.

Finally, the dispersion energy, in many cases, substantially contributes to the interaction energy and often more so than the orbital interactions. Especially for the bidentate hydrogen and sulphur catalysts, as well as the pnictogen catalysts, the dispersion component is in the same order of magnitude as the total interaction energy and comprises more than 30% of the stabilization. This is mainly caused by the stacked coordination of the benzene ring of benzaldehyde with ring moieties in the catalysts, as shown by the green regions in the NCI plots in Figure 2C. We also observe significantly more dispersion energy for Cl_cat and Br_cat, compared to I_cat. This can be traced back to a different geometrical arrangement in their corresponding transition state (TS), where the C–H…π interaction present in the Br_cat-catalyzed TS structure is more stabilizing than a halogen-bond interaction of increased strength due to a better positioning of the halogen atom with respect to the carbonyl group of benzaldehyde.

These discussed trends are mostly followed for the other reactions. The interaction energies of the complexes with methyl vinyl ketone are generally divided into similar contributions as was the case for benzaldehyde. Some differences are related to the smaller amount of dispersion, for example, for H2_cat, also yielding a lower Pauli repulsion, or for Cl_cat and Br_cat resulting in a larger portion of electrostatic interactions, which is more in agreement with what is observed for I_cat. Interestingly, I3_cat scores lower on the interaction scale, despite the larger dispersion energy, similar Pauli repulsion, and equivalent ratio between orbital and electrostatic interactions. However, their absolute values are significantly lower for the neutral tripodal catalyst (although acting more like a bidentate catalyst) compared to the cationic I_cat. For the complexes with imine (Table S4), the dispersion energy component is significantly reduced, contributing at most 18% to the total attractive interactions. Enhanced electrostatic interactions, on average 10% larger than for benzaldehyde, agree with the MEP results. Contributions from orbital interactions, however, are very comparable.

In summary, the most stable non-covalent complexes are the hydrogen-bond catalysts and the catalyst based on iodine. This is caused by both the low strain and strong interaction energy. The H_cat complexes are stabilized due to low Pauli repulsion between occupied orbitals of the two fragments and the high electrostatic interactions, while those with I_cat are mostly stabilized by electrostatic and orbital interactions. Whereas the complexation energy between H2_cat and BEN is determined, for a large part, by dispersion interactions, orbital interactions play a more important role in the combinations with MVK and IM. Finally, although the BF_3_ complexes show a very large interaction energy and would thus be thought to be a stable complex, this is counterbalanced by the large strain and Pauli repulsion.

### 2.2. Activation Barriers of Potentially Catalyzed Reactions

To evaluate the effectiveness of each catalyst, the reaction barriers of the different systems are compared for the endo DA reaction between benzaldehyde and 1,3-dimethyl 2,3-butadiene (see Table 3). For the uncatalyzed reaction, the activation energy amounts to a significant 28.4 kcal mol^−1^ at 0 K and even 36.6 kcal mol^−1^ at 298.15 K. At 0 K, the DA reaction can be catalyzed by all Lewis acid-like systems, even though the efficiency of BF_3_ cannot be matched. At 25 °C, only half of the reactions are catalyzed when looking at the Gibbs free activation energy. Besides the BF_3_ catalyst, the largest reduction in activation barrier is obtained for the positively charged H_cat and I_cat. Note that this corresponds with the most stabilized reaction complexes, both at 0 K and 298.15 K. Thus, this indicates that in the transition-state structure, a larger energy stabilization than in complex occurs. This extra stabilization in the TS compared to the reactant complexes (R1_cat_R2) is very similar for all non-covalent catalysts, with an R^2^ value of 0.91 at 0 K and even 0.94 at 25 °C (cf. Tables S5 and S6 in Supplementary Materials). The most effective catalysts also lead to stable product complexes, and energy will be required to remove the catalysts from the adduct. The uncatalyzed reaction barriers for reactions 2 and 3 are significantly lower than for reaction 1, but the reduction in barrier upon catalysis is also much smaller in absolute value. Similar trends in catalysis to reaction 1 are observed for reaction 2 between MVK and CP, with the charged hydrogen- and halogen-bond donors acting as effective catalysts at 25 °C (Tables S7 and S8 in Supplementary Materials). For reaction 3 between IM and BD, some pronounced differences can be observed (see Tables S9 and S10). Only BF_3_ and I_cat truly catalyze the reaction at 25 °C and surprisingly also at 0 K, meaning that the reactant complexes are slightly more stabilized than the transition states.

### 2.3. What Makes Lewis Acids and Non-Covalent-Interaction Donors Catalyze the Diels–Alder Reaction? Different Perspectives

Due to a lowering in LUMO energy of the dienophile in the presence of a catalyst, the reduced orbital energy difference between the diene’s HOMO and the dienophile’s LUMO, or LUMO+1/2/3 depending on the catalyst used (cf. Table S11 in Supplementary Materials), was long thought to be the source of a reduction in the activation barrier of the DA reaction. Although the lowering in ∆E^‡^ is seen for all catalysts and for all three reactions, when the sum of the energies of the separate reagents is taken as a reference, there exists merely a reasonable linear trend in the reduction in ∆E^‡^ versus the reduction in the HOMO-LUMO gap. The linear correlation plots, added to the Supplementary Materials in Figure S2, yield an R^2^ = 0.83 for reaction 1, R^2^ = 0.84 for reaction 2, and R^2^ = 0.72 for reaction 3; however, only the uncharged non-covalently catalyzed dienophiles are considered.

This result does not entirely agree with the observations made by Vermeeren and co-workers in their study on the rate enhancement of the Diels–Alder reaction catalyzed by Lewis acids (LA) such as BF_3_, AlCl_3_, SnCl_4_, TiCl_4_, and ZnCl_2_. They found that the lowering in orbital energy for the different LA-bound dienophiles does not follow the trend in reactivity at all. By applying the activation strain model, they put forward that the lower energy barriers due to LA catalysis originate from reduced Pauli repulsion and, to a lesser extent, lower strain energy and thus not from improved orbital interactions.

Firstly, we apply the activation strain model, combined with the EDA approach, as done in the work of Vermeeren et al. [23,24]. Next, we propose an alternative and complementary way to uncover the determining physical factors for the catalyzing process. Dissimilarities in the outcome would indicate that applying a different analysis model can influence the assessment of the main factors of chemical reactivity. We first focus on the BF_3_-catalyzed Diels–Alder reaction between benzaldehyde (BEN) and 2,3-dimethyl 1,3-butadiene (DMB).

#### 2.3.1. BF_3_-Catalyzed Reaction between BEN and DMB

**Figure 3:**
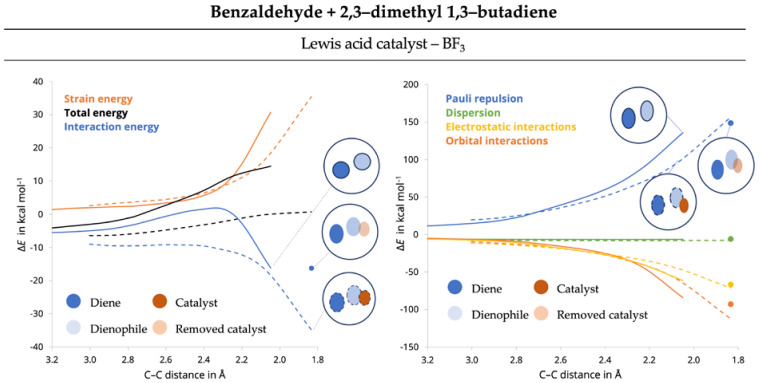
ASM (**left**) and EDA (**right**) analysis of the uncatalyzed and BF_3_-catalyzed reactions between benzaldehyde and 2,3-dimethyl 1,3-butadiene. The solid line represents the reaction without the catalyst, and the dashed line represents the reaction with the catalyst. The dots represent the values for the transition-state geometry of the catalyzed reaction with the catalyst deleted. Pictograms symbolize the system considered.

In the activation strain model (ASM) of reactivity, the potential energy along the intrinsic reaction coordinate, obtained via an IRC calculation, is decomposed into a destabilizing strain energy—associated with the deformation of the reagents from their equilibrium structure to the geometry they adapt when they start to interact—and an interaction energy that can be further broken down into Pauli repulsion energy, and electrostatic, orbital, and dispersion interaction terms. When considering Diels–Alder reactions, one usually projects the intrinsic reaction coordinate onto the bond length of one of the formed DA bonds, a geometrical parameter that clearly defines the evolution of the DA reaction and that can be easily measured as such, allowing for the comparison of uncatalyzed and catalyzed reactions. For reaction 1 between benzaldehyde and 2,3-dimethyl 1,3-butadiene, we opt for the C⋯C bond distance and not the O⋯C bond distance since the former one becomes markedly shorter upon catalysis. In Figure 3, the ASM and accompanying EDA analyses are plotted for the uncatalyzed and BF_3_-catalyzed BEN + DMB reactions. Note that for all ASM and EDA analyses to follow, fragment 1 consists of the dienophile, in the absence or presence of the catalyst, and fragment 2 is the diene.

When the C–C distance in the reactant complex shortens to around 2.3 Å, the strain energy sharply rises, which is where the interaction energy for the uncatalyzed reaction is the smallest, i.e., maximum in the curve. However, the increase in deformation energy is much steeper for the uncatalyzed reaction (solid line) compared to the BF_3_-catalyzed reaction (dashed line). At the same time, the interaction energy steeply becomes more negative. The interaction energy lines of catalyzed and uncatalyzed reactions finally seem to coincide when the transition state (TS) of the uncatalyzed reaction is reached. To summarize, when the reagents in the reactant complex start to approach each other, the difference in potential energy curves with and without catalysts can be attributed to a difference in interaction energy between the DA fragments, in favor of the catalyzed case. However, before the TS of the uncatalyzed reaction is reached, the reduced strain becomes the dominant factor and not the interaction energy.

According to the EDA plot on the right in Figure 3, the Pauli repulsion energy of the catalyzed reaction (dotted line) is consistently lower than that of the uncatalyzed reaction (solid line). This lower Pauli repulsion is completely offset by a decrease in attractive orbital and electrostatic interactions near the TS of the uncatalyzed reaction. Nonetheless, at the TS structures of the uncatalyzed and catalyzed reactions, the slope of the orbital interaction curve is approaching the same magnitude as that of the Pauli repulsion curve, whereas the slope for the electrostatic interactions remains relatively constant along the full reaction path. This hints at orbital interactions playing a significant role as well.

These results do not entirely agree with the conclusions drawn by Vermeeren et al. for the Lewis acid-catalyzed DA reaction between isoprene and methyl acrylate. The main issue is that in the region of importance, i.e., close to the transition-state structures, there is very little “overlap” between the two cases due to a substantial difference in C–C distance in the two transition-state geometries, being 2.049 Å for the uncatalyzed and 1.834 Å for the catalyzed reaction. This difference in C–C distance relates to the asynchronous behavior upon bond formation in the catalyzed DA reaction, in which the C–C bond length in the TS is markedly smaller than the C–O bond length. As a consequence, one compares the uncatalyzed TS configuration with a configuration of BF_3_–BEN and DMB fragments in preparation mode and, later, the catalyzed TS with a configuration that is halfway through bond formation for the uncatalyzed case. We know that the magnitudes of the EDA contributions are highly sensitive to changes in fragment distances, and therefore, any comparison should take this into account. Nevertheless, the ASM/EDA assessment presented in Figure 3 goes against chemical intuition. This was also put forward by Vermeeren and co-authors, who emphasized that one should exhibit caution when comparing the ASM energy values of different reactions with TSs occurring at different points along the reaction coordinate [29].

To resolve this issue, we propose the following approach. To measure the influence of the catalyst on the stabilizing and destabilizing interactions between BEN and DMB in the catalyzed activated complex, a comparison is made between the EDA for the TS with the catalyst and the EDA at the same TS geometry but with the catalyst deleted. As such, the concern for varying fragment distance does not present itself, and differences in interaction energy values can be easily evaluated in a quantitative way. In addition, the relative contributions of the interaction energy components for the case where the catalyst was removed will be matched with those of the true uncatalyzed TS results to assure the consistency of our approach. The results for the current reaction are indicated as dots in Figure 3, at the C–C bond length of the BF_3_-catalyzed TS, and tabulated in Table 4.

The presence of BF_3_ in the transition-state geometry of the catalyzed reaction more than doubles the interaction energy. This increase is mainly caused by the stronger orbital interactions (–19.6 kcal mol^−1^) and better electrostatics (–5.0 kcal mol^−1^), whereas the dispersion energy remains approximately constant. Moreover, the Pauli repulsion increases by a substantial 7.8 kcal mol^−1^. These results contradict the ASM/EDA outcome. When we look at the relative contributions to the stabilizing part of the interaction energy, it is confirmed that the uncatalyzed TS values nicely agree with the BF_3_ deleted values. However, an increase of 3% in orbital interactions is noted upon the addition of BF_3_, at the expense of the electrostatic interactions. This also agrees with the substantial orbital-interaction component upon formation of the BF_3_**_**BEN complex, as was described earlier. A more thorough analysis of these surprising results will be given later. First, the effect of non-covalently bound catalysts will be scrutinized.

#### 2.3.2. Non-Covalent Catalysis of the Reaction BEN and DMB

**Figure 4:**
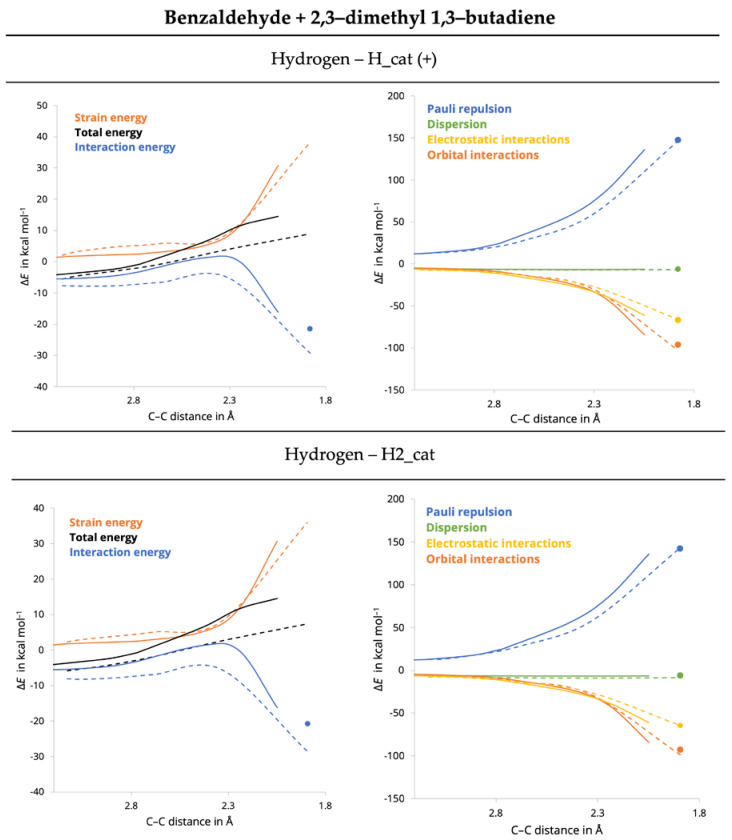
ASM (**left**) and EDA (**right**) analysis of the uncatalyzed and hydrogen-bond-catalyzed reactions between benzaldehyde and 2,3-dimethyl 1,3-butadiene. The solid line represents the reaction without the catalyst, and the dashed line represents the reaction with the catalyst. The dots represent the values for the transition-state geometry of the catalyzed reaction with the catalyst deleted.

**Figure 5:**
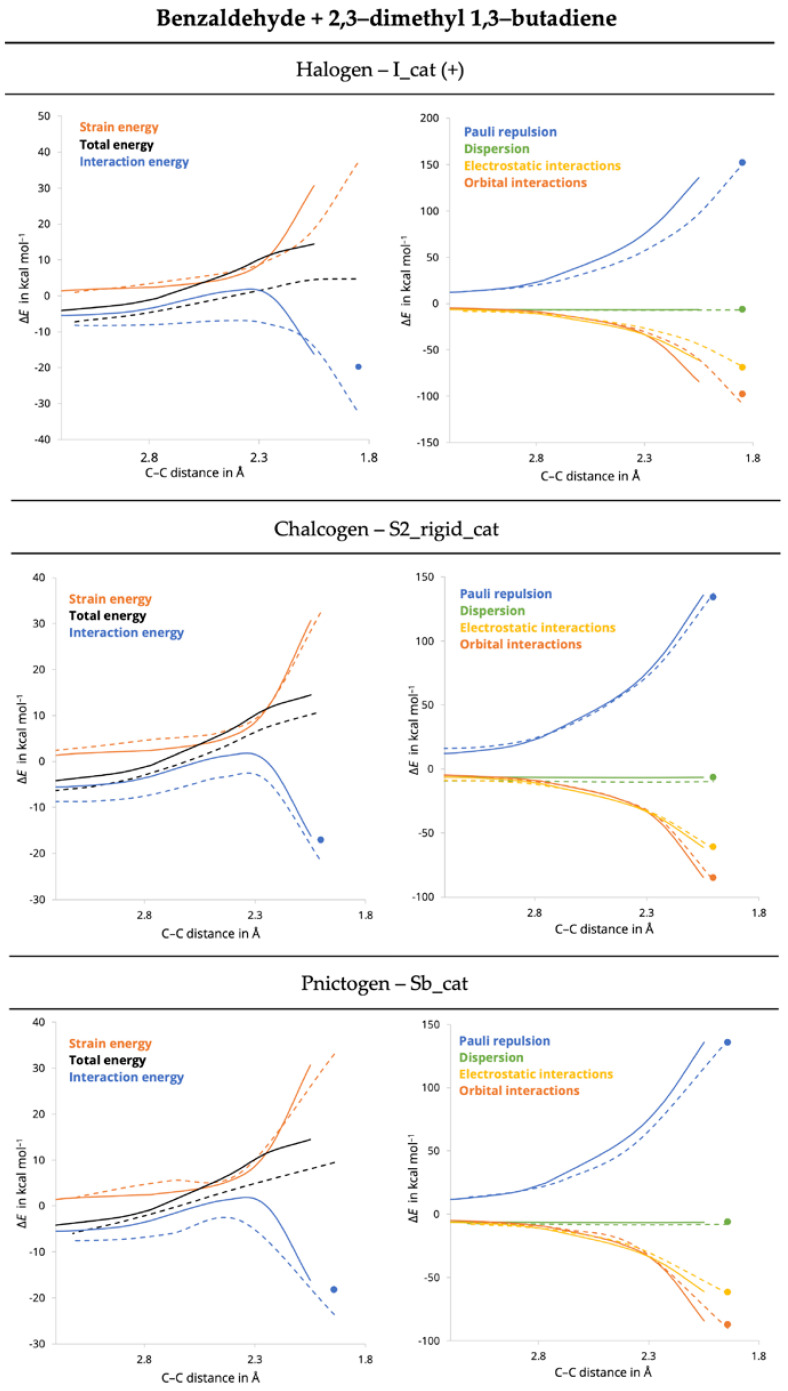
ASM (**left**) and EDA (**right**) analysis of the uncatalyzed and halogen-, chalcogen-, and pnictogen-bond-catalyzed reactions between benzaldehyde and 2,3-dimethyl 1,3-butadiene. The solid line represents the reaction without the catalyst, and the dashed line represents the reaction with the catalyst. The dots represent the values for the transition-state geometry of the catalyzed reaction with the catalyst deleted.

For the BEN–DMB reaction, we discovered in Section 2.2 that for every type of non-covalent interaction, a catalyst could be found, though the catalyzing effect was substantially smaller than for our Lewis acid. For our analysis, we selected H_cat and H2_cat for the hydrogen-, I_cat for the halogen-, S2_rigid_cat for the chalcogen-, and Sb_cat for the pnictogen-catalyzed reactions. The ASM/EDA plots are shown in Figure 4 and Figure 5, whereas the values using the alternative approach can be found in Table 5.

**Figure 6:**
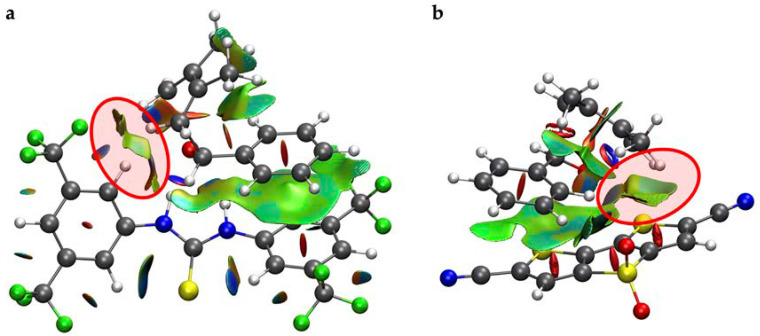
Additional dispersion interactions between the catalyst ((**a**) H2_cat; (**b**) S2_rigid_cat), in interaction with the dienophile, and the diene, further stabilizing the transition-state geometry (see red circles). Green indicates π-π and Van der Waals interactions, blue strong non-covalent bonds and red repulsive interactions such as ring strain.

For the hydrogen-bond catalysts, a slightly different picture emerges from the ASM/EDA plots. Strain plays a minor role, whereas the interaction energy due to the approaching fragments is clearly more stabilizing for the catalytic case along the full trajectory. This stabilization is mainly caused by a lowering in Pauli repulsion between occupied orbitals of the diene and dienophile. The orbital and electrostatic energy curves of both cases are more or less superimposed and are therefore of lesser importance. This is in accordance with the results of Vermeeren and co-workers in their work on hydrogen-bond-donor catalysis of Diels–Alder reactions with (thio)ureas [24]. Let us now consider our proposed alternative approach (dots in Figure 4 and values in Table 5). From the EDA analysis of the catalytic TS geometry with and without the catalyst present, we deduce that, besides the electrostatic interactions, the Pauli repulsion term also remains the same (within 1.1 kcal mol^−1^). The orbital interactions, however, get significantly stabilized by −6.7 and −6.0 kcal mol^−1^ for the positively charged H_cat and the bifunctional H2_cat, respectively. Moreover, dispersion interactions also increase in the case of the larger-sized H2_cat by roughly half of the orbital interaction stabilization, due to extra dispersion between the catalyst and DMB (cf. Figure 6a). The relative contributions of the components ∆E_elst_, ∆E_oi_, and E_disp_ for the case with the catalyst deleted are slightly different from those for the actual uncatalyzed reaction, with the ∆E_oi_ being 1.5% more important and ∆E_elst_ 0.9% less important for the former case. Nonetheless, the presence of H_cat makes orbital interactions gain an additional 1.6% further dominance at the expense of the electrostatic interactions, whereas with H2_cat, a 1.3% increase in dispersion is seen, again at the cost of electrostatic interactions. As in the case of Lewis acid BF_3_, the two alternative views yield other controlling factors for the hydrogen-bond-donor catalysis of this specific Diels–Alder reaction.

Halogen-bond donor I_cat was found to be the most catalyzing of all non-covalently bound catalysts. It follows a very similar pattern in the ASM/EDA profile as Lewis acid BF_3_, with the strain becoming more important near the uncatalyzed transition state and the reduction in Pauli repulsion upon catalysis being compensated by a less attractive orbital and electrostatic interactions. The alternative method, in which the I_cat is simply deleted, strongly indicates orbital interactions as the dominant factor with an additional stabilization of 10.8 kcal mol^−1^, and only a small reduction in Pauli repulsion energy (−1.7 kcal mol^−1^) is identified. In relative terms, the orbital interactions gain more dominance (i.e., increase of 2.6%), whereas the electrostatic interactions lose importance. Analogous conclusions are found for Br_cat and Cl_cat, with similar differences for Pauli repulsion, electrostatics and dispersion, though a less-pronounced difference in orbital interaction energy (Table 5).

Finally, we study the less catalyzing chalcogen-bond-donor S2_rigid_cat and pnictogen-bond-donor Sb_cat. The activation strain model, coupled with the energy decomposition analysis, shows that Sb_cat behaves like the hydrogen-bonding catalysts, with Pauli repulsion reduction being dominant. For S2_rigid_cat, nearly all curves overlap with the uncatalyzed DA reaction curves. This is not surprising, as the catalytic activity is very limited. The second approach again renders somewhat different findings. For Sb_cat, the Pauli repulsion energy is slightly higher (+1.3 kcal mol^−1^) compared to the deleted case, and roughly the same increase in electrostatic effects is observed. Orbital interactions are more stabilizing upon catalysis with a value of 3.3 kcal mol^−1^, which is, however, significantly less than for the other described catalysts. Moreover, dispersion energy contributes to a lowering of the transition-state energy. Somewhat unexpectedly, dispersion becomes the main factor distinguishing the degree of interaction in the TS geometry in the presence or absence of S2_rigid_cat, with a difference of a substantial 3.6 kcal mol^−1^ in favor of the former (cf. Figure 6b). Furthermore, more Pauli repulsion (+2.6 kcal mol^−1^) is present in the case of the catalyzed TS. Increased orbital (−2.1 kcal mol^−1^) and electrostatic (−1.7 kcal mol^−1^) interaction energies play only a minor role for this type of catalyst.

#### 2.3.3. Comparison of Both Approaches for Reactions of IM and BD and MVK and CP

**Figure 7:**
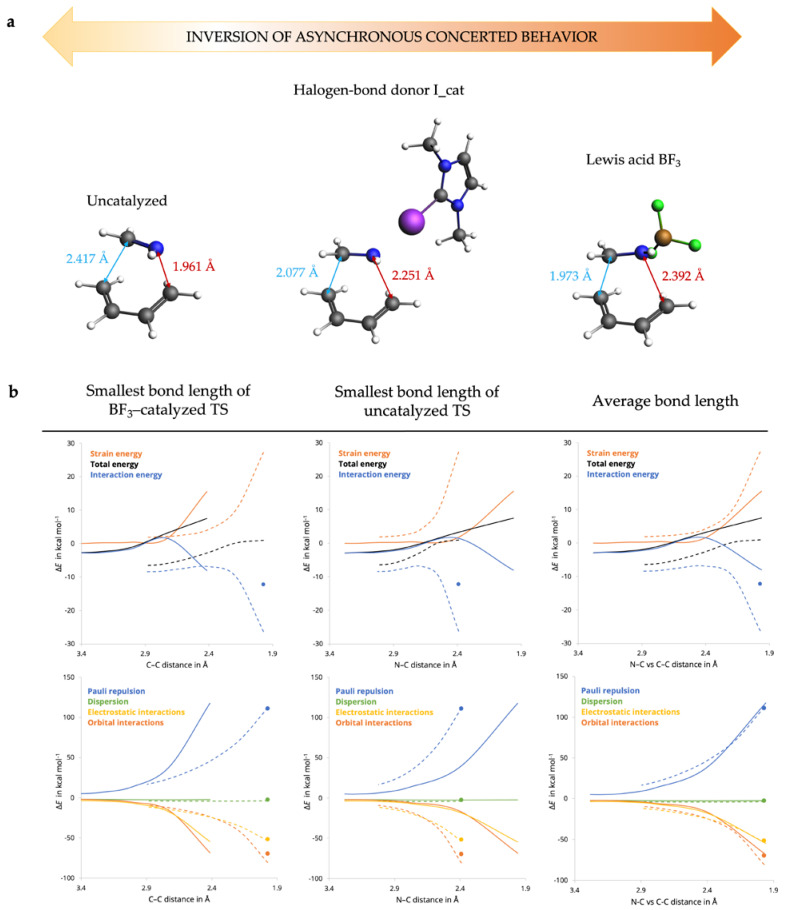
(**a**) Bond distances of the two bonds under formation in the transition-state geometries of the uncatalyzed, I_cat catalyzed, and BF_3_-catalyzed Diels–Alder reactions between methylene imine and 1,3-butadiene, and their connection to the asynchronous concerted behavior of the reactions. (**b**) ASM/EDA analyses of uncatalyzed and BF_3_-catalyzed reactions along the reaction coordinate projected onto the C–C bond length, the shortest bond being formed in the BF_3_-catalyzed TS geometry (**left**); the N–C bond length, the shortest bond being formed in the uncatalyzed TS geometry (**middle**); and the average bond distance (**right**).

Do the same conclusions apply for the other two reactions? First, we consider the Diels–Alder reaction between methylene imine and 1,3-butadiene, as this again involves a hetero-DA reaction, now with the inclusion of a nitrogen atom in the formed six-membered ring. The ASM/EDA analysis along the C–C distance relating to the shortest of the two bonds being formed, at least upon catalysis, is presented in Figure S5 in the Supplementary Materials whereas the catalyst-deletion method analysis is tabulated in Table S13. Only BF_3_ and I_cat catalyze the reaction, and both have a pronounced effect on the asynchronicity of the reaction. Whereas in the uncatalyzed transition state the length of the N–C bond is considerably smaller than that of the C–C bond, by almost 0.5 Å, the inverse is true for the catalyzed reactions, with a 0.4 Å larger bond length for N–C in the presence of the most catalyzing BF_3_ Lewis acid; see Figure 7a.

Due to this inversion in bond lengths, the conclusions drawn from the activation strain model of reactivity are dictated by our choice of projection of the reaction coordinate for the ASM/EDA plots. As shown in Figure 7b, selection of the C–C bond length, which corresponds to the smallest bond length of the two bonds under formation in the catalyzed transition-state geometry, leads to the supposition that strain is more prominent for the uncatalyzed reaction and that the smaller interaction energy between the uncatalyzed DA fragments is foremost determined by augmented Pauli repulsion (at least until the uncatalyzed TS is reached). However, when the reaction coordinate is projected onto the N–C bond length, which is the smallest bond length in the uncatalyzed TS geometry, we would deduct the following: reduced strain and Pauli repulsion for the uncatalyzed DA reaction but also significantly less stabilizing orbital and electrostatic interactions, yielding again a larger interaction energy for the catalyzed reaction. Finally, projecting the reaction coordinate onto the average bond length of the two bonds being formed in the Diels–Alder process results in the notion that strain does not play a significant role, and therefore, catalysis can be attributed to a stronger interaction between the catalyzed dienophile and diene. Strangely, though, at around 2.4 Å, where the interaction energy of the catalyzed reaction becomes steeply more negative, a switch in driving forces takes place, going from increased to reduced Pauli repulsion and from more to less attractive electrostatic and orbital interactions in favor of the catalyzed reaction. It is clear that an unambiguous projection of the reaction coordinate is lacking, giving rise to contrasting conclusions on the different components of the activation energy.

The second approach, where the effect of the catalyst is measured in the geometry of the catalyzed TS by comparing the EDA results in the presence and absence of that catalyst, does not depend on the selected projection (cf. dots in graphs of Figure 7b and Table S13). Using this method, we distinguish no change in Pauli repulsion in the presence of BF_3_ yet a large additional stabilization from orbital interactions. For I_cat, there is substantial reduction in Pauli repulsion, even though orbital interactions still contribute slightly more to the TS stabilization.

The issue of a univocal IRC-projection quantity does not present itself for the third reaction we considered in our study, between methyl vinyl ketone and cyclopentadiene. The difference in C–C bond length between the uncatalyzed and all catalyzed versions is limited to 0.1 Å. The resulting ASM/EDA plots are presented in Figure S3. The analysis is quite similar for all catalysts. The interaction energy is more determining than the strain energy until the earlier TS geometry is approached. The reduction in Pauli repulsion is responsible for the stronger interaction between the DA reagents upon catalysis. This is in line with the findings of Vermeeren et al. Our analysis approach partially corroborates with these results (Table S12). Except for BF_3_ and H2_cat, the dominant factor is indeed the Pauli repulsion reduction; nonetheless, the effect of increased orbital interactions is only slightly less important. Interestingly, in most cases, the electrostatic interactions are somewhat less stabilizing. Lewis acid BF_3_ is an exception due to much stronger orbital interactions, twice as important as Pauli repulsion reduction, which conflicts again with the findings of Vermeeren and co-authors [23]. The other exception, catalysis with H2_cat, results in improved orbital interactions and additional dispersion interactions between the catalyst and cyclopentadiene, accounting for the enlarged total interaction energy in the presence of H2_cat.

### 2.4. Molecular Orbital Picture: Orbital Interactions and Pauli Repulsion

To help summarize the above results, we have catalogued the most important findings using both approaches in Table 6. Note that there is never full agreement between the two perspectives, with the findings for the catalysis of the methyl vinyl ketone and cyclopentadiene Diels–Alder reaction perhaps agreeing the most, and those for the catalysis of the reaction between benzaldehyde and 2,3-dimethyl 1,3-butadiene being completely opposite. In general, we can conclude that in the ASM/EDA approach, orbital interactions play no role or, more typically, an adverse role in the catalysis of the DA reaction, whereas according to the catalyst-deletion method, they are usually the dominant physical factor explaining the catalytic activity of the Lewis acid(-like) compounds. The ASM/EDA approach always points to Pauli repulsion reduction, which is not always the case according to the other perspective, with the BF_3_-catalyzed BEN + DMB reaction as the most contrasting example.

**Figure 8:**
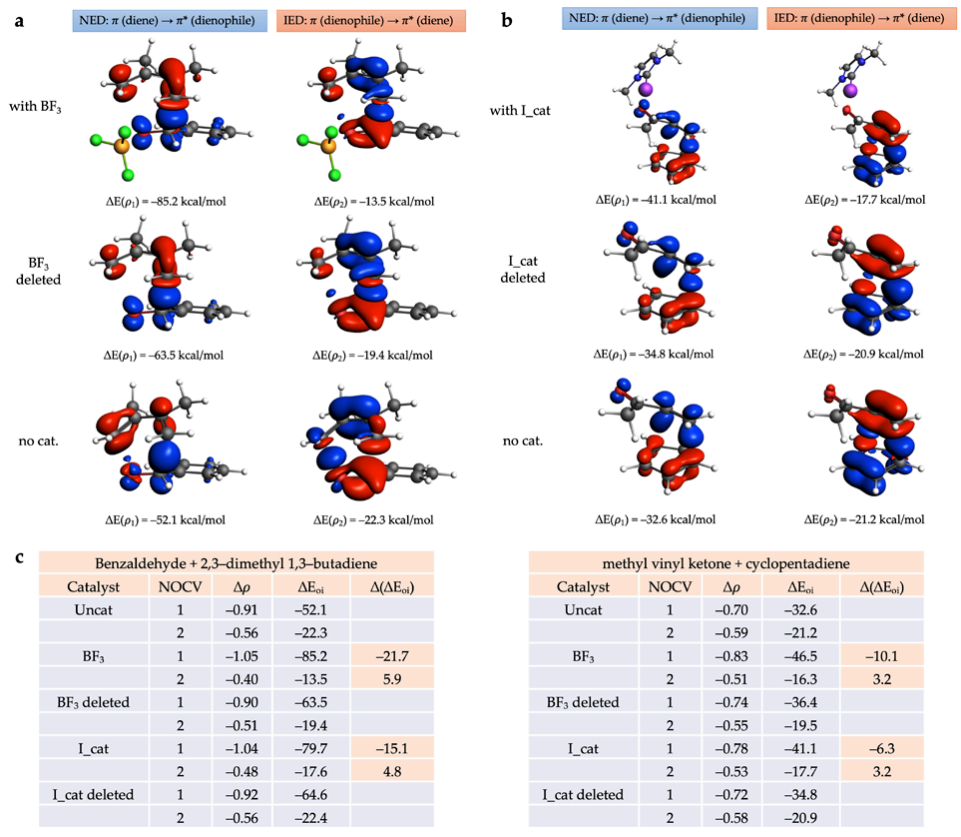
Contour plots of NOCV deformation densities (red for depletion, blue for accumulation) and associated orbital energies ΔE(ρ) for the normal-electron-demand (NED) and inverse-electron-demand (IED) interactions of (**a**) the BF_3_-catalyzed and uncatalyzed benzaldehyde + 2,3-dimethyl 1,3-butadiene Diels–Alder reaction, and of (**b**) the I_cat-catalyzed and uncatalyzed methyl vinyl ketone + cyclopentadiene Diels–Alder reaction. (**c**) NOCV deformation densities Δρ and associated orbital energies ΔE_oi_, with the difference between catalyzed and catalyst-deleted values for NED and IED interactions highlighted in orange.

To validate the findings of the catalyst-deletion approach, we first focus on the assessment of the orbital interactions in the absence and presence of the catalyst. To this end, the NOCV (natural orbitals for chemical valence) method was applied, in which charge-density reorganization can be visualized and measured. Two pairs of NOCVs are relevant for the Diels–Alder reaction. One pair relates to the normal-electron-demand (NED) orbital interaction, i.e., the interaction between the HOMO of the diene and the LUMO of the (catalyst-bound) dienophile, whereas the second pair is connected to the inverse-electron-demand (IED) orbital interaction, i.e., between the LUMO of the diene and the π-HOMO of the (catalyst-bound) dienophile. Since all three reactions are confirmed NED Diels–Alder reactions, the NED orbital interaction is stronger than the IED orbital interaction due to the smaller orbital energy gap for the former. Second, one expects that the NED orbital interaction is further enhanced when a Lewis acid-like catalyst is added to the DA system. Third, it is anticipated that the catalyst weakens IED orbital interactions. The interplay between all these effects will determine whether orbital interactions contribute to an enhancement or reduction of the total interaction energy in the transition-state geometry. In Figure 8, we have plotted and listed the NOCV deformation densities and the associated orbital interaction energies for the NED and IED interactions of the catalyzed, catalyst-deleted, and uncatalyzed BEN + DMB and MVK + CP Diels–Alder reactions. For the BEN + DMB reaction, a very asymmetric deformation density is observed for the NED interaction in the catalyzed and catalyst-deleted cases, with charge accumulation mainly on the carbon atom of the benzaldehyde double bond, which agrees with the asynchronous concerted mechanism. In the uncatalyzed transition-state structure, the asymmetry in density depletion in the diene is rather opposite to the previous cases; however, the density accumulation in the dienophile remains on the carbon atom–and not on the benzaldehyde’s oxygen. The NOCV plots for the IED interaction between π(dienophile) and π*(diene) show mainly density depletion at the carbon atom of BEN’s double bond for all three cases, whereas charge transfer follows the asynchronous behavior, as was seen for the NED interaction. As witnessed from the table in Figure 8c, total deformation densities for NED and IED match quite well between the uncatalyzed and catalyst-deleted pictures. However, when catalyst BF_3_ or I_cat is present, a significant increase in Δρ for NED and a decrease for IED are computed, accompanied by a more (former) or less (latter) stabilizing orbital interaction energy. The difference in NED and IED orbital interaction energy between the catalyzed and catalyst-deleted reaction amounts to, respectively, −21.7 and 5.9 kcal mol^−1^ for BF_3_ and −15.1 and 4.8 kcal mol^−1^ for I_cat, clearly favoring the catalyzed case. Since the NED and IED orbital interactions account for 90% of the total orbital interaction energy, this confirms the key role that orbital interactions play in the catalysis of the BEN + DMB Diels–Alder reaction. The same exercise can be done for the methyl vinyl ketone and cyclopentadiene DA reaction. For this reaction, a more symmetric density depletion and accumulation take place, associated with a more synchronous concerted mechanism. The differences in density deformation and orbital interaction energies are reduced for the NED interaction, compared to the previous reaction, and this translates to smaller differences in ΔE_oi_ between the catalyzed and catalyst-deleted reactions but still favoring the catalyzed reaction. Again, since the NED and IED orbital interactions contribute 90% to the total ΔE_oi_, these results are consistent with our proposed EDA analysis.

**Figure 9:**
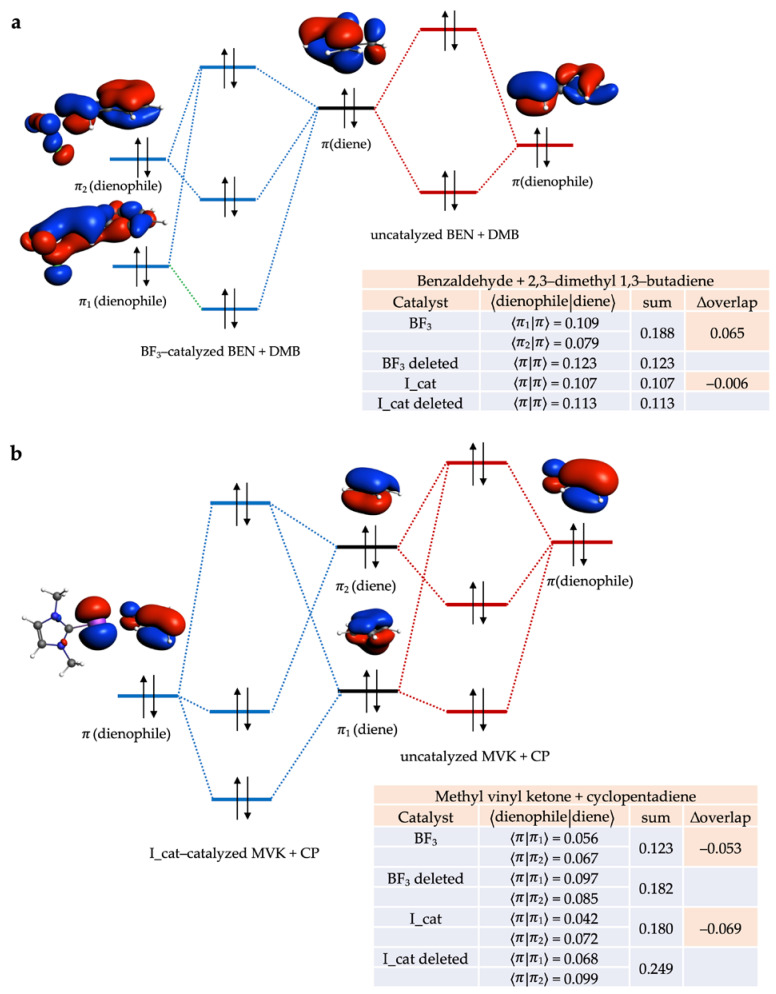
Molecular orbital diagrams showing the occupied π-MOs of diene and dienophile responsible for the main part of the Pauli repulsion energy term, and their occupied-occupied MO overlap values. (**a**) Catalyzed and catalyst-deleted benzaldehyde + 2,3-dimethyl 1,3-butadiene Diels–Alder reaction; (**b**) catalyzed and catalyst-deleted methyl vinyl ketone + cyclopentadiene Diels–Alder reaction.

Finally, we also examine the contribution from Pauli repulsion. As evident from Table 6, depending on the type of Diels–Alder reaction and the type of catalyst, Pauli repulsion reduction or enhancement takes place when a catalyst is present. For example, for the endo hetero-DA reaction between benzaldehyde and 2,3-dimethyl 1,3-butadiene, the energetically lowest occupied π-MO of the diene DMB, with all 4 carbon-centered p-orbitals in phase, can overlap with the π-MO of the dienophile BEN, causing substantial Pauli repulsion. When Lewis acid BF_3_ is present, an additional π-MO is constructed, in which the p-orbitals of the O=C double bond combine with one of the three p-orbitals of each of the fluorine atoms of BF_3_, yielding an even stronger overlap with the π-MO of DMB (Figure 9a). Summing up the overlap integrals with both π-MOs of BF_3_-catalyzed BEN, i.e., $\langle \pi_{1,\mathrm{dienophile}}|\pi_{\mathrm{diene}}\rangle =0.109$ and $\langle \pi_{2,\mathrm{dienophile}}|\pi_{\mathrm{diene}}\rangle =0.079$, leads to increased Pauli repulsion compared to the catalyst-deleted situation with $\langle \pi_{\mathrm{dienophile}}|\pi_{\mathrm{diene}}\rangle =0.123$.

For the I_cat-catalyzed BEN + DMB reaction, no additional π-MO for I_cat-catalyzed BEN can be found. When we compare the overlap between the π-MO predominantly located on the O=C double bond of BEN and the all-in-phase π-MO of DMB, we notice a slight reduction in occupied-occupied orbital overlap upon catalysis (0.107 vs. 0.113), which is consistent with the small difference in Pauli repulsion energy. Finally, we also examine the occupied-occupied orbital overlap for the DA reaction between methyl vinyl ketone and cyclopentadiene (Figure 9b). Both for the BF_3_- and I_cat-catalyzed cases, the main overlap contributing to Pauli repulsion is between the π-MO of the C=C double bond of MVK and two occupied π-MO’s of cyclopentadiene, i.e., the HOMO–1 and the energetically lower-lying HOMO–6. In both cases, the respective overlaps decrease upon the addition of the catalysts and, when summed, yield a reduction of −0.053 for BF_3_ and −0.069 for I_cat. This is again consistent with the drop in Pauli repulsion energy.

## 3. Materials and Methods

The systems were studied in the gas phase using density functional theory (DFT). The geometry optimizations and vibrational frequency analyses were carried out with the M06-2X functional [30] combined with cc-pVDZ [31] as a basis set, as implemented in the Gaussian 16 software [32]. For the heavy elements considered in this work, i.e., I, Te, and Sb, pseudopotentials were used to describe the core electrons, allowing the implicit inclusion of relativistic effects [33]. It was verified that transition-state structures are characterized by a single imaginary frequency, and subsequent intrinsic reaction coordinate (IRC) path calculations confirmed the connection of all stationary points on the PES. Refined electronic energies were obtained using M06-2X with additional Grimme D3 dispersion [34] and the cc-pVDZ(-PP), cc-pVTZ(-PP), cc-pVQZ(-PP) basis sets by extrapolating to the complete basis set (CBS) limit using the Feller three-point extrapolation [35,36], thus avoiding basis set superposition error.

Although the more empirical M06-2X functional already incorporates a significant amount of dispersion, M06-2X-D3 still performs better in many cases. The different parameters in the Grimme dispersion correction D3 as part of the M06-2X-D3 functional have been tuned for this DFA [37]. M06-2X-D3 is rated as the best hybrid functional in the GMTKN30 database study for general main group thermochemistry, kinetics, and non-covalent interactions, which also includes barrier heights of pericyclic reactions and reaction energies of Diels–Alder reactions. In addition, we have used the same level of theory in a previous study [38], for which we benchmarked M06-2X-D3/CBS against CCSD(T)/CBS data for the endo and exo Diels–Alder reactions between furan and maleimide, with identical results for the energy barriers.

The systems were further analyzed using molecular electrostatic potential (MEP) maps and non-covalent interaction index (NCI) plots [39] at the M06-2X-D3/cc-pVDZ(-PP) level of theory. The Ziegler-Rauk-type energy decomposition analysis (EDA) [40,41,42], the activation strain model (ASM) [43] analyses using the PyFrag 2019 (version 0.1.0) software [44], and the natural orbitals for chemical valence (NOCV) [45] analysis of the Gaussian16-optimized structures were computed using the PBE/TZ2P (small core) level of theory [46,47,48] in combination with the dispersion correction Grimme’s D3 method, using the Amsterdam Modeling Suite (AMS) [49]. Relativistic effects were considered by using the zeroth-order regular approximation (ZORA) [50]. The levels of theory used for the quantum-chemical analysis tools are based on previous work in our group [27,51,52], in which non-covalent interactions were analyzed in a similar fashion as the current study. In particular, the PBE functional was selected because it is as good as dispersion-correction free, allowing for a correct quantification of the dispersion energy term in EDA via Grimme’s D3 correction.

## 4. Conclusions

In this study, we tried to elucidate the potential catalytic effect of Lewis acid BF_3_ and a variety of non-covalent-bond donors on three (hetero-)Diels–Alder reactions. We considered hydrogen-, halogen-, chalcogen-, and pnictogen-bond systems, which have been proposed in the literature for their (potential) catalytic capacity in cycloaddition reactions. First, the stability of the catalyst–dienophile complex was examined. At 25 °C, reactant complex formation was not always favored, with the hydrogen-bond-donor and iodine catalysts forming the most stable complexes. The interaction energy can be mainly attributed to strong electrostatic interactions between the catalyst and the dienophile, though orbital interactions also constitute a significant part for the most stabilized complexes. For H_cat, we also noted reduced Pauli repulsion energy, in comparison to the other potential catalysts. After addition of the diene, about half of the non-covalent-bond donors catalyze reactions 1 (benzaldehyde + 2,3-dimethyl 1,3-butadiene) and 2 (methyl vinyl ketone + cyclopentadiene), whereas for reaction 3 (methylene imine + 1,3-butadiene), only the Lewis acid BF_3_ and halogen-bond-donor I_cat accelerate the reaction. We found that the most stabilized catalyst–dienophile complexes also yield the largest reductions in Diels–Alder activation energies.

For a wide variety of chemical reactions, the activation strain model of reactivity, combined with a Ziegler-Rauk-type energy decomposition analysis, has been applied to gain valuable information on which physical factors influence the reactive system. Very recently, Diels–Alder catalysis via Lewis acids or hydrogen-bond donors has been explored by Vermeeren et al. [23,24], for which the authors concluded that catalytic activity can be associated with Pauli repulsion reduction rather than increased orbital interactions. However, in our study of two hetero-Diels–Alder reactions (reactions 1 and 3), the inclusion of a catalyst was shown to largely affect the degree of asynchronicity of the reaction, even to such an extent that the uncatalyzed and catalyzed transition-state geometries were located at very different points along the reaction coordinate’s projection. Following the ASM principle and thus evaluating the interaction energy contribution curves at a consistent geometry, i.e., at the same value for the reaction coordinate’s projection, can sometimes lead to results open for interpretation since geometries are compared at a different stage in the bond formation process. We therefore opted for the following approach: a one-to-one comparison between EDA values in the presence and absence of the catalyst, both taken at the geometry of the catalyzed transition state, ignoring geometry changes but ensuring consistency in geometrical variables. We discovered that, upon catalysis, Pauli repulsion can either be reduced, enhanced, or of no significance depending on the diene–dienophile combination and the catalyst used. In all cases, however, orbital interactions were improved upon addition of the catalyst. In most cases, electrostatics and dispersion played only a minor role. The results were corroborated by a detailed look at the molecular orbital diagrams. We demonstrated that enhanced normal-electron-demand orbital interactions dominate diminished inverse-electron-demand orbital interactions and that Pauli repulsion can be increased when additional occupied π-MOs are formed between the catalyst and dienophile that can overlap with the occupied π-MO of the diene. This method gives different insights into the catalysis of DA reactions with non-covalent catalysts and shows an alternative, yet complementary approach to the original ASM/EDA method.

Finally, we would like to stress that we focused on the unperturbed, intrinsic effect of Lewis acid and non-covalent catalysts on the (hetero-)Diels–Alder reaction, i.e., without considering solvation effects or counterions, in the case of the cationic hydrogen- or halogen-bond catalysts. Wittkopp and Schreiner [7], for example, found that the catalytic effectiveness of their neutral bidentate hydrogen-bond donor for the DA reaction between methyl vinyl ketone and cyclopentadiene is largest in nonpolar solvents, such as in cyclohexane, but is present in polar solvents as well. Jungbauer and co-authors [53] observed the catalytic effect of the cationic iodine catalyst for the same DA reaction in deuterated dichloromethane. We expect that the presence of solvent molecules may not only induce geometrical changes but also alter the energetic contributions associated with the different physical factors as quantified by an energy decomposition analysis. Although out of the scope of this study, there exist ways to include solvation effects in the EDA. One possibility is to incorporate more and more solvent molecules explicitly in a stepwise manner [54]. A second option is to make use of a thermochemical cycle, with the solvent treated as a third agent. The energy profile of the solution phase is then decomposed into a solute term—in which the reaction partners are considered in vacuum, though, in their solution-phase geometries—and a solvation energy term. More information on this approach can be found in Ref. [55].

## Figures and Tables

**Table 1:** ∆E (0 K) and ∆G (298.15 K) values for the reactant complex formation between benzaldehyde (R1) and the catalyst (cat), in kcal mol^−1^. The + sign in between parentheses indicates a positively charged catalyst.

	∆E (0 K)	∆G (298.15 K)
	R1_cat Complex	R1_cat Complex
Uncatalyzed endo	0.0	0.0
BF_3_	−12.4	−0.8
**H_cat** (+)	−13.9	−4.3
**H2_cat**	−14.4	−0.6
**Cl_cat** (+)	−8.8	1.9
**Br_cat** (+)	−9.0	1.7
**I_cat** (+)	−13.9	−4.3
**S2_rigid_cat**	−9.9	2.6
**S2_flex_cat**	−7.4	3.7
**Se_cat**	−5.1	5.8
**Te_cat**	−6.9	4.3
**P_cat**	−9.3	3.3
**As_cat**	−9.8	3.2
**Sb_cat**	−12.5	−0.1

**Table 2:** Energy decomposition analysis for the reactant complexes formed between benzaldehyde and the set of catalysts. All energies are denoted in kcal mol^−1^. The numbers between brackets indicate the relative contribution of each energy component to the stabilizing part of the total interaction energy.

	∆E_complex_	∆E_strain_	∆E_int_	∆E_Pauli_	∆E_elst_	∆E_oi_	E_disp_
BF_3_	−11.9	16.5	−28.4	96.4	−64.6 (52%)	−58.9 (47%)	−1.2 (1%)
**H_cat (+)**	−15.1	0.3	−15.5	8.3	−14.5 (61%)	−8.0 (34%)	−1.3 (5%)
**H2_cat**	−12.9	1.9	−14.8	23.2	−18.1 (48%)	−8.6 (23%)	−11.3 (30%)
**Cl_cat (+)**	−8.5	1.1	−9.6	10.7	−10.5 (49%)	−7.0 (33%)	−3.9 (18%)
**Br_cat (+)**	−8.7	2.0	−10.7	9.6	−9.1 (47%)	−6.2 (32%)	−3.9 (20%)
**I_cat (+)**	−16.3	0.5	−16.8	19.7	−20.9 (57%)	−14.6 (40%)	−1.0 (3%)
**S2_rigid_cat**	−8.3	0.5	−8.9	17.4	−12.1 (46%)	−5.3 (20%)	−8.9 (34%)
**S2_flex_cat**	−6.7	0.6	−7.3	12.8	−9.0 (45%)	−4.2 (21%)	−6.8 (34%)
**Se_cat**	−3.8	1.5	−5.3	11.4	−8.9 (53%)	−4.5 (27%)	−3.3 (20%)
**Te_cat**	−6.0	0.5	−6.4	15.4	−11.9 (55%)	−6.6 (30%)	−3.3 (15%)
**P_cat**	−6.6	0.8	−7.4	19.9	−12.6 (46%)	−6.0 (22%)	−8.7 (32%)
**As_cat**	−7.6	0.3	−7.9	20.6	−13.3 (46%)	−6.5 (23%)	−8.7 (31%)
**Sb_cat**	−8.7	0.4	−9.1	24.7	−16.6 (49%)	−8.6 (25%)	−8.5 (25%)

**Table 3:** Activation energies ∆E^‡^ at 0 K and Gibbs free activation energies ∆G^‡^ at 298.15 K in kcal mol^−1^ for the endo DA reaction between benzaldehyde and 1,3-dimethyl 2,3-butadiene and the HOMO-LUMO energy gap, ∆(H-L), in eV for the diene and dienophile. The activation barriers are calculated relative to the most stable reactant complex, R1_cat + R2 or R1_cat_R2, if stable and to the separate reagents if not stable. V denotes that reaction is catalyzed, X when it is not.

	∆(H-L)	∆E^‡^	Catalyzed?	∆G^‡^	Catalyzed?
Uncatalyzed endo	−6.9	28.4		36.6	
BF_3_	−5.5	12.6	V	21.1	V
**H_cat (+)**	−4.0	26.6	V	32.1	V
**H_2__cat**	−6.2	24.1	V	32.4	V
**Cl_cat (+)**	−4.1	23.4	V	32.1	V
**Br_cat (+)**	−4.0	22.9	V	31.5	V
**I_cat (+)**	−4.2	24.7	V	30.5	V
**S2_rigid_cat**	−6.5	26.3	V	36.0	V
**S2_flex_cat**	−6.5	27.4	V	39.8	X
**Se_cat**	−6.6	26.6	V	40.9	X
**Te_cat**	−6.4	26.1	V	39.0	X
**P_cat**	−6.4	26.0	V	41.3	X
**As_cat**	−6.5	25.2	V	40.5	X
**Sb_cat**	−6.2	24.3	V	34.5	V

**Table 4:** EDA analysis of the transition state for the uncatalyzed and BF_3_-catalyzed reactions between benzaldehyde and 2,3-dimethyl 1,3-butadiene, the latter in the presence and absence of the catalyst. All values are given in kcal mol^−1^. The values in brackets represent the relative contribution of the given energies to the stabilizing part of the total interaction energy.

	∆E_int_	∆E_Pauli_	∆E_elst_	∆E_oi_	E_disp_
Uncatalyzed	−16.4	135.9	−61.3 (40%)	−84.5 (56%)	−6.4 (4%)
BF_3_	−34.8	156.6	−71.5 (37%)	−112.3 (59%)	−7.7 (4%)
BF_3_ deleted	−16.2	148.9	−66.5 (40%)	−92.6 (56%)	−6.0 (4%)

**Table 5:** EDA analysis of the transition state for the uncatalyzed and non-covalently catalyzed reactions between benzaldehyde and 2,3-dimethyl 1,3-butadiene, the latter in the presence and absence of the catalyst. All values are given in kcal mol^−1^. The values in brackets represent the relative contribution of the given energies to the stabilizing part of the total interaction energy.

	∆E_int_	∆E_Pauli_	∆E_elst_	∆E_oi_	E_disp_
Uncatalyzed	−16.4	135.9	−61.3 (40%)	−84.5 (56%)	−6.4 (4%)
**H_cat** (+)	−29.3	146.6	−66.2 (38%)	−102.9 (58%)	−6.8 (4%)
**H_cat** deleted	−21.5	147.4	−66.6 (39%)	−96.2 (57%)	−6.2 (4%)
**H2_cat**	−28.8	143.1	−64.7 (38%)	−98.7 (57%)	−8.5 (5%)
**H2_cat** deleted	−20.8	142.0	−64.1 (39%)	−92.7 (57%)	−5.9 (4%)
**Cl_cat** (+)	−25.3	130.5	−59.1 (38%)	−90.2 (58%)	−6.5 (4%)
**Cl_cat** deleted	−17.5	132.4	−59.7 (40%)	−84.0 (56%)	−6.1 (4%)
**Br_cat** (+)	−27.0	132.5	−59.9 (38%)	−93.1 (58%)	−6.6 (4%)
**Br_cat** deleted	−18.0	134.5	−60.7 (40%)	−85.8 (56%)	−6.0 (4%)
**I_cat** (+)	−32.6	150.3	−67.8 (37%)	−108.4 (59%)	−6.8 (4%)
**I_cat** deleted	−19.7	152.0	−68.6 (40%)	−96.8 (57%)	−6.1 (4%)
**S2_rigid_cat**	−21.7	137.1	−62.3 (39%)	−86.7 (55%)	−9.8 (6%)
**S2_rigid_cat** deleted	−17.0	134.5	−60.6 (40%)	−84.6 (56%)	−6.2 (4%)
**Sb_cat**	−23.7	137.4	−62.6 (39%)	−90.3 (56%)	−8.1 (5%)
**Sb_cat** deleted	−18.1	136.1	−61.4 (40%)	−87.0 (56%)	−5.9 (4%)

**Table 6:** Summary of the importance of the different interaction energy components using the activation strain model (ASM), combined with EDA, and the catalyst-deletion approach. V indicates a positive contribution to the catalyst-induced stabilization of the transition state whereas X denotes a negative contribution.

Reaction	Catalyst	Method	Pauli Repulsion	Electrostatic	Orbital	Dispersion
BEN + DMB	BF_3_	ASM	VVV	X	XX	/
		deletion	XX	V	VVVV	/
MVK + CP	BF_3_	ASM	V	X	/	/
		deletion	V	/	VV	/
IM + BD	BF_3_	ASM *^a^*	VVVV	XXX	XXX	/
		deletion	/	/	VVV	/
BEN + DMB	**I_cat**	ASM	VVV	XX	XX	/
		deletion	/	/	VVV	/
MVK + CP	**I_cat**	ASM	VV	X	X	/
		deletion	VV	X	VV	/
IM + BD	**I_cat**	ASM *^a^*	VVVV	XXX	XXX	/
		deletion	VV	X	VV	/

*^a^* taken with the C–C distance as the projection of the intrinsic reaction coordinate (cf. Figure 7b).

## Data Availability

The geometry of all structures has been included in the Supplementary Materials.

## Supplementary Materials

The following supporting information can be downloaded at https://www.mdpi.com/article/10.3390/ijms24054938/s1.

## References

[1] Pindur U., Lutz G., Otto C. (1993). Acceleration and Selectivity Enhancement of Diels-Alder Reactions by Special and Catalytic Methods. Chem. Rev..

[2] Briou B., Améduri B., Boutevin B. (2021). Trends in the Diels-Alder Reaction in Polymer Chemistry. Chem. Soc. Rev..

[3] Marchand A.P., Vidyasagar V., Buckner M.B., Holman P.O. (1987). Lewis Acid Catalysis of a Diels-Alder Cycloaddition—An Undergraduate Organic Experiment. J. Chem. Educ..

[4] Palacios F., Alonso C., Arrieta A., Cossío F.P., Ezpeleta J.M., Fuertes M., Rubiales G. (2010). Lewis Acid Activated Aza-Diels-Alder Reaction of N-(3-Pyridyl)Aldimines: An Experimental and Computational Study. Eur. J. Org. Chem..

[5] Sakata K., Fujimoto H. (2020). Roles of Lewis Acid Catalysts in Diels-Alder Reactions between Cyclopentadiene and Methyl Acrylate. ChemistryOpen.

[6] Mehranfar A., Izadyar M., Khavani M., Housaindokht M.R. (2019). Understanding the Role of Noncovalent Interactions on the Rate of Some Diels-Alder Reactions in Different Solvents. Int. J. Quantum Chem..

[7] Wittkopp A., Schreiner P.R. (2003). Metal-Free, Noncovalent Catalysis of Diels-Alder Reactions by Neutral Hydrogen Bond Donors in Organic Solvents and in Water. Chem. Eur. J..

[8] Huang Y., Rawal V.H. (2002). Hydrogen-Bond-Promoted Hetero-Diels-Alder Reactions of Unactivated Ketones. J. Am. Chem. Soc..

[9] Esrafili M.D., Mohammadian-Sabet F., Baneshi M.M. (2015). The Dual Role of Halogen, Chalcogen, and Pnictogen Atoms as Lewis Acid and Base: Triangular XBr:SHX:PH_2_X Complexes (X = F, Cl, Br, CN, NC, OH, NH_2_, and OCH_3_). Int. J. Quantum Chem..

[10] Haraguchi R., Hoshino S., Sakai M., Tanazawa S., Morita Y., Komatsu T., Fukuzawa S. (2018). Bulky Iodotriazolium Tetrafluoroborates as Highly Active Halogen-Bonding-Donor Catalysts. Chem. Commun..

[11] Heinen F., Engelage E., Dreger A., Weiss R., Huber S.M. (2018). Iodine (III) Derivatives as Halogen Bonding Organocatalysts. Angew. Chem.—Int. Ed..

[12] Takeda Y., Hisakuni D., Lin C., Minakata S. (2015). 2-Halogenoimidazolium Salt Catalyzed Aza-Diels−Alder Reaction through Halogen-Bond Formation. Org. Lett..

[13] Kaasik M., Metsala A., Kaabel S., Kriis K., Järving I., Kanger T. (2019). Halo-1,2,3-Triazolium Salts as Halogen Bond Donors for the Activation of Imines in Dihydropyridinone Synthesis. J. Org. Chem..

[14] Kee C.W., Wong M.W. (2016). In Silico Design of Halogen-Bonding-Based Organocatalyst for Diels-Alder Reaction, Claisen Rearrangement, and Cope-Type Hydroamination. J. Org. Chem..

[15] Nziko V.D.P.N., Scheiner S. (2016). Catalysis of the Aza-Diels-Alder Reaction by Hydrogen and Halogen Bonds. J. Org. Chem..

[16] Wonner P., Vogel L., Maximilian D., Kniep F., Mallick B., Werz D.B., Huber S.M. (2017). Chalcogen Bonding Carbon-Halogen Bond Activation by Selenium-Based Chalcogen Bonding. Angew. Chem.—Int. Ed..

[17] Benz S., Poblador-Bahamonde A.I., Low-Ders N., Matile S. (2018). Catalysis with Pnictogen, Chalcogen, and Halogen Bonds. Angew. Chem.—Int. Ed..

[18] Schmauck J., Breugst M. (2017). The Potential of Pnicogen Bonding for Catalysis—A Computational Study. Org. Biomol. Chem..

[19] Yaghoobi F., Mahboub M.S. (2018). Theoretical Study on the Aza−Diels−Alder Reaction Catalyzed by PHCl_2_ Lewis Acid via Pnicogen Bonding. J. Phys. Chem..

[20] Vermeeren P., Tiezza D., Van Dongen M., Fernández I. (2021). Lewis Acid-Catalyzed Diels-Alder Reactions: Reactivity Trends across the Periodic Table. Chem. Eur. J..

[21] Houk K.N. (1975). Frontier Molecular Orbital Theory of Cycloaddition Reactions. Acc. Chem. Res..

[22] Xia Y., Yin D., Rong C., Xu Q., Yin D., Liu S. (2008). Impact of Lewis Acids on Diels-Alder Reaction Reactivity: A Conceptual Density Functional Theory Study. J. Phys. Chem. A.

[23] Vermeeren P., Hamlin T.A., Fernández I., Bickelhaupt F.M. (2020). How Lewis Acids Catalyze Diels–Alder Reactions. Angew. Chem.—Int. Ed..

[24] Vermeeren P., Hamlin T.A., Bickelhaupt F.M., Fernández I. (2021). Bifunctional Hydrogen Bond Donor-Catalyzed Diels–Alder Reactions: Origin of Stereoselectivity and Rate Enhancement. Chem. Eur. J..

[25] Yepes D., Pérez P., Jaque P., Fernández I. (2017). Effect of Lewis Acid Bulkiness on the Stereoselectivity of Diels-Alder Reactions between Acyclic Dienes and α,β-Enals. Org. Chem. Front..

[26] Portela S., Cabrera-Trujillo J.J., Fernández I. (2021). Catalysis by Bidentate Iodine(III)-Based Halogen Donors: Surpassing the Activity of Strong Lewis Acids. J. Org. Chem..

[27] De Vleeschouwer F., Denayer M., Pinter B., Geerlings P., De Proft F. (2018). Characterization of Chalcogen Bonding Interactions via an In-Depth Conceptual Quantum Chemical Analysis. J. Comput. Chem..

[28] Pinter B., Nagels N., Herrebout W.A., De Proft F. (2013). Halogen Bonding from a Hard and Soft Acids and Bases Perspective: Investigation by Using Density Functional Theory Reactivity Indices. Chem. Eur. J..

[29] Vermeeren P., Hamlin T.A., Bickelhaupt F.M. (2021). Chemical Reactivity from an Activation Strain Perspective. Chem. Commun..

[30] Zhao Y., Truhlar D.G. (2008). The M06 Suite of Density Functionals for Main Group Thermochemistry, Thermochemical Kinetics, Noncovalent Interactions, Excited States, and Transition Elements: Two New Functionals and Systematic Testing of Four M06-Class Functionals and 12 Other Function. Theor. Chem. Acc..

[31] Dunning T.H. (1989). Gaussian Basis Sets for Use in Correlated Molecular Calculations. I. The Atoms Boron through Neon and Hydrogen. J. Phys. Chem. A.

[32] Frisch M.J., Trucks G.W., Schlegel H.B., Scuseria G.E., Robb M.A., Cheeseman J.R., Scalmani G., Barone V., Petersson G.A., Nakatsuji H. (2016). Gaussian 16.

[33] Peterson K.A. (2003). Systematically Convergent Basis Sets with Relativistic Pseudopotentials. I. Correlation Consistent Basis Sets for the Post-d Group 13–15 Elements. J. Chem. Phys..

[34] Grimme S., Antony J., Ehrlich S., Krieg H. (2010). A Consistent and Accurate Ab Initio Parametrization of Density Functional Dispersion Correction (DFT-D) for the 94 Elements H-Pu. J. Phys. Chem. A.

[35] Feller D. (1993). The Use of Systematic Sequences of Wave Functions for Estimating the Complete Basis Set, Full Configuration Interaction Limit in Water. J. Chem. Phys..

[36] Feller D. (1992). Application of Systematic Sequences of Wave Functions to the Water Dimer. J. Chem. Phys..

[37] Goerigk L., Grimme S. (2011). A Thorough Benchmark of Density Functional Methods for General Main Group Thermochemistry, Kinetics, and Noncovalent Interactions. Phys. Chem. Chem. Phys..

[38] Mangialetto J., Kiano G., Vermeersch L., Van Mele B., Van Den Brande N., De Vleeschouwer F. (2022). Hydrogen-Bond-Assisted Diels–Alder Kinetics or Self-Healing in Reversible Polymer Networks? A Combined Experimental and Theoretical Study. Molecules.

[39] Johnson E.R., Keinan S., Mori-Sánchez P., Contreras-Garciá J., Cohen A.J., Yang W. (2010). Revealing Noncovalent Interactions. J. Am. Chem. Soc..

[40] Ziegler T., Rauk A. (1979). A Theoretical Study of the Ethylene-Metal Bond in Complexes between Cu^+^, Ag^+^, Au^+^, Pt^0^, or Pt^2+^ and Ethylene, Based on the Hartree-Fock-Slater Transition-State Method. Inorg. Chem..

[41] Ziegler T., Rauk A. (1979). Carbon Monoxide, Carbon Monosulfide, Molecular Nitrogen, Phosphorus Trifluoride, and Methyl Isocyanide as.Sigma. Donors and.Pi. Acceptors. A Theoretical Study by the Hartree-Fock-Slater Transition-State Method. Inorg. Chem..

[42] Bickelhaupt F.M., Baerends E.J. (2007). Kohn-Sham Density Functional Theory: Predicting and Understanding Chemistry. Rev. Comput. Chem..

[43] Fernández I., Bickelhaupt F.M. (2014). The Activation Strain Model and Molecular Orbital Theory: Understanding and Designing Chemical Reactions. Chem. Soc. Rev..

[44] Sun X., Soini T.M., Poater J., Hamlin T.A., Bickelhaupt F.M. (2019). PyFrag 2019—Automating the Exploration and Analysis of Reaction Mechanisms. J. Comput. Chem..

[45] Mitoraj M.P., Michalak A., Ziegler T. (2008). A Combined Charge and Energy Decomposition Scheme for Bond Analysis. J. Chem. Theory Comput..

[46] Perdew J.P. (1986). Density-Functional Approximation for the Correlation Energy of the Inhomogeneous Electron Gas. Phys. Rev. Lett..

[47] Perdew J.P., Burke K., Ernzerhof M. (1996). Generalized Gradient Approximation Made Simple. Phys. Rev. Lett..

[48] Van Lenthe E., Baerends E.J. (2003). Optimized Slater-Type Basis Sets for the Elements 1–118. J. Comput. Chem..

[49] SCM Theoretical Chemistry; ASM2021.105. Vrije Universiteit: Amsterdam, The Netherlands. http://www.scm.com.

[50] Van Lenthe E., Baerends E.J., Snijders J.G. (1993). Relativistic Regular Two-Component Hamiltonians. J. Chem. Phys..

[51] Skara G., Pinter B., Top J., Geerlings P., De Proft F., De Vleeschouwer F. (2015). Conceptual Quantum Chemical Analysis of Bonding and Noncovalent Interactions in the Formation of Frustrated Lewis Pairs. Chem. Eur. J..

[52] De Vleeschouwer F., De Proft F., Ergün Ö., Herrebout W., Geerlings P. (2021). A Combined Experimental/Quantum-Chemical Study of Tetrel, Pnictogen, and Chalcogen Bonds of Linear Triatomic Molecules. Molecules.

[53] Jungbauer S.H., Walter S.M., Schindler S., Rout L., Kniep F., Huber S.M. (2014). Activation of a Carbonyl Compound by Halogen Bonding. Chem. Commun..

[54] Hansen T., Roozee J.C., Bickelhaupt F.M., Hamlin T.A. (2022). How Solvation Influences the SN2 versus E2 Competition. J. Org. Chem..

[55] Bickelhaupt F.M., Houk K.N. (2017). Analyzing Reaction Rates with the Distortion/Interaction-Activation Strain Model. Angew. Chem. Int. Ed..

