# Supplementary material for: Unravelling the Mechanism and Governing Factors in Lewis Acid and Non-Covalent Diels–Alder Catalysis: Different Perspectives
Source: Int J Mol Sci. 2023 Mar 3;24(5):4938. doi: 10.3390/ijms24054938 (PMC10003447; doi:10.3390/ijms24054938)
Supplement: Supplementary file 1 [file ijms-24-04938-s001.zip › ijms-2225398-supplementary.pdf]

Article

Unravelling the Mechanism and Governing Factors in Lewis Acid and Non-Covalent Diels–Alder Catalysis: Different Perspectives

Lise Vermeersch, Frank De Proft, Vicky Faulkner and Freija De Vleeschouwer *

Research Group of General Chemistry (ALGC), Vrije Universiteit Brussel (VUB), Pleinlaan 2, B-1050 Brussels, Belgium

* Correspondence: freija.de.vleeschouwer@vub.be

1.1. Complexes with methyl vinyl ketone

Table S1. *ΔE (0 K) and ΔG (298.15 K) values for the reactant complex formation between methyl vinyl ketone (R1) and the catalyst (cat), in kcal mol-1. The + sign in between parentheses indicates a positively charged catalyst. Level of theory: M06-2X-D3/CBS//M06-2X/cc-pVDZ.*

	ΔE (0 K)	ΔG (298.15 K)
	**R1_cat complex**	**R1_cat complex**
Uncatalyzed *endo*	0.0	0.0
BF_3	-12.9	-1.0
**H_cat** (+)	-14.4	-4.8
**H2_cat**	-13.0	-3.3
**Cl_cat** (+)	-9.6	-2.0
**Br_cat** (+)	-10.9	-1.9
**I_cat** (+)	-14.2	-5.1
**I3_cat**	-10.4	1.5
**S2_rigid_cat**	-9.1	3.4
**S2_flex_cat**	-5.9	4.7
**Se_cat**	-5.9	5.2
**Te_cat**	-7.7	3.4
**P_cat**	-5.1	6.1
**As_cat**	-3.3	8.7
**Sb_cat**	-8.3	3.8

Table S2. *Energy decomposition analysis for the reactant complexes formed between methyl vinyl ketone and the set of catalysts. All energies are denoted in kcal/mol. The numbers between brackets indicate the relative contribution of each energy component to the stabilizing part of the total interaction energy. Level of theory: PBE-D3/TZ2P//M06-2X/cc-pVDZ.*

	**$\Delta E_{complex}$**	**ΔE_{strain}**	**ΔE_{int}**	**ΔE_{Pauli}**	**ΔE_{elst}**	**ΔE_{oi}**	**E_{disp}**
BF_3	-11.8	20.1	-32.0	112.2	-75.3 (**52**)	-67.0 (**47**)	-1.8 (**1**)
**H_cat** (+)	-15.9	0.4	-16.3	9.9	-16.4 (**63**)	-8.1 (**31**)	-1.6 (**6**)
**H2_cat**	-15.4	1.7	-17.1	14.1	-17.2 (**55**)	-10.4 (**33**)	-3.6 (**12**)
**Cl_cat** (+)	-9.7	0.8	-10.5	10.9	-14.4 (**60**)	-8.4 (**35**)	-1.1 (**5**)
**Br_cat** (+)	-11.8	1.2	-13.0	7.4	-11.6 (**65**)	-5.5 (**30**)	-0.9 (**5**)
**I_cat** (+)	-16.0	0.4	-16.4	20.0	-20.8 (**57**)	-14.3 (**39**)	-1.2 (**3**)
**I3_cat**	-10.1	0.9	-11.0	19.0	-15.1 (**50**)	-9.8 (**33**)	-5.2 (**17**)
**S2_rigid_cat**	-7.7	1.0	-8.7	16.9	-11.9 (**47**)	-5.6 (**22**)	-8.0 (**31**)
**S2_flex_cat**	-5.4	0.5	-5.9	11.8	-9.3 (**53**)	-4.5 (**25**)	-3.9 (**22**)
**Se_cat**	-3.6	1.6	-5.2	12.3	-8.6 (**49**)	-4.7 (**27**)	-4.2 (**24**)
**Te_cat**	-5.8	0.7	-6.5	16.3	-11.9 (**52**)	-6.8 (**30**)	-4.2 (**18**)
**P_cat**	-4.1	0.8	-4.9	13.3	-8.4 (**46**)	-4.0 (**22**)	-5.8 (**32**)
**As_cat**	-1.7	4.6	-6.2	16.8	-11.0 (**48**)	-5.5 (**24**)	-6.5 (**28**)
**Sb_cat**	-2.5	5.5	-8.0	23.4	-16.0 (**51**)	-8.5 (**27**)	-6.9 (**22**)

1.2. Complexes with methylene imine

Table S3. *ΔE (0 K) and ΔG (298.15 K) values for the reactant complex formation between* **methylene imine** *(R1) and the catalyst (cat), in kcal mol*$^{-1}$*. The + sign in between parentheses indicates a positively charged catalyst. Level of theory: M06-2X-D3/CBS//M06-2X/cc-pVDZ.*

	ΔE (0 K) **R1_cat complex**	ΔG (298.15 K) **R1_cat complex**
Uncatalyzed *endo*	0.0	0.0
BF_3	-20.5	-9.2
**H_cat** (+)	-11.4	-2.6
**H2_cat**	-11.7	-2.6
**Cl_cat** (+)	-8.2	0.5
**Br_cat** (+)	-10.2	-1.1
**I_cat** (+)	-14.3	-4.8
**S2_rigid_cat**	-6.2	3.1
**S2_flex_cat**	-4.3	4.1
**Se_cat**	-5.3	4.1
**Te_cat**	-7.7	2.4
**P_cat**	-6.6	3.7
**As_cat**	-7.5	2.9
**Sb_cat**	-12.1	-1.8

Table S4. *Energy decomposition analysis for the reactant complexes formed between methylene imine and the set of catalysts. All energies are denoted in kcal/mol. The numbers between brackets indicate the relative contribution of each energy component to the stabilizing part of the total interaction energy.*

	**$\Delta E_{complex}$**	**ΔE_{strain}**	**ΔE_{int}**	**ΔE_{Pauli}**	**ΔE_{elst}**	**ΔE_{oi}**	**E_{disp}**
BF_3	-20.8	22.2	-42.9	133.9	-94.6 (**53**)	-80.9 (**46**)	-1.3 (**1**)
**H_cat** (+)	-9.4	1.0	-10.4	6.1	-11.0 (**67**)	-3.6 (**22**)	-1.9 (**11**)
**H2_cat**	-13.5	1.0	-14.5	15.0	-17.2 (**58**)	-9.0 (**31**)	-3.3 (**11**)
**Cl_cat** (+)	-9.6	0.6	-10.3	16.0	-18.1 (**61**)	-10.6 (**36**)	-0.9 (**3**)
**Br_cat** (+)	-12.2	1.4	-13.6	10.6	-13.2 (**63**)	-6.8 (**33**)	-0.9 (**4**)
**I_cat** (+)	-17.1	1.1	-18.2	31.2	-29.2 (**59**)	-19.3 (**39**)	-1.0 (**2**)
**S2_rigid_cat**	-6.8	0.3	-7.1	14.7	-13.0 (**60**)	-6.7 (**31**)	-2.0 (**9**)
**S2_flex_cat**	-4.6	0.8	-5.3	11.1	-9.2 (**56**)	-4.2 (**26**)	-3.0 (**18**)
**Se_cat**	-4.2	1.6	-5.7	13.6	-11.6 (**60**)	-5.6 (**29**)	-2.2 (**11**)
**Te_cat**	-7.6	0.8	-8.4	23.8	-19.6 (**61**)	-10.5 (**32**)	-2.1 (**7**)
**P_cat**	-5.4	0.5	-5.9	17.4	-13.5 (**58**)	-6.4 (**27**)	-3.4 (**15**)
**As_cat**	-6.4	0.4	-6.8	19.8	-15.7 (**59**)	-7.5 (**28**)	-3.4 (**13**)
**Sb_cat**	-8.1	1.3	-9.4	32.0	-25.3 (**61**)	-13.0 (**31**)	-3.2 (**8**)

2. Energies at 0K and Gibbs free energies at 25°C for all stationary points of the (catalyzed) Diels-Alder reactions

2.1. DA reactions with benzaldehyde and 2,3-dimethyl 1,3-butadiene

Table S5. *Energy at 0 K expressed in kcal/mol for the formed complexes, transition state and products as referred to the separate reagents for the DA reaction between benzaldehyde (R1) and 2,3-dimethyl 1,3-butadiene (R2) (Reaction1). ΔE‡ refers to the activation energy at 0K.*

ΔE (0 K)	**R1 + R2 + cat**	**R1_cat + R2**	**R1_(cat)_R2**	**TS**	**P_cat**	**P + (cat)**	**ΔE‡**
uncat *endo*	0.0	-	-5.4	23.0	-	-21.4	28.4
BF3	0.0	-12.4	-19.2	-6.7	-34.2	-21.4	12.5
**H_cat** (+)	0.0	-13.9	-22.6	4.0	-35.7	-21.4	28.6
**H2_cat**	0.0	-14.4	-20.7	3.4	-36.3	-21.4	24.1
**Cl_cat** (+)	0.0	-8.8	-15.9	7.5	-32.9	-21.4	23.4
**Br_cat** (+)	0.0	-9.0	-16.1	6.8	-33.2	-21.4	22.9
**I_cat** (+)	0.0	-13.9	-21.7	3.0	-35.4	-21.4	24.7
**S2_rigid_cat**	0.0	-9.9	-16.5	9.7	-31.5	-21.4	26.2
**S2_flex_cat**	0.0	-7.4	-13.1	14.3	-26.7	-21.4	27.4
**Se_cat**	0.0	-5.1	-11.3	15.3	-24.7	-21.4	26.6
**Te_cat**	0.0	-6.9	-13.0	13.2	-26.7	-21.4	26.2
**P_cat**	0.0	-9.3	-12.0	14.0	-30.9	-21.4	26.0
**As_cat**	0.0	-9.8	-12.2	13.0	-27.6	-21.4	25.2
**Sb_cat**	0.0	-12.5	-16.5	7.8	-31.4	-21.4	24.3

Table S6. *Gibbs free energy at 298.15 K expressed in kcal/mol for the formed complexes, transition state and products as referred to the separate reagents for the DA reaction between benzaldehyde (R1) and 2,3-dimethyl 1,3-butadiene (R2) (Reaction1). ΔG‡ refers to the Gibbs free energy of activation at 298.15K; k (s^{-1}) refers to the rate constant of the reaction (at the standard concentration of 1 mol L-1), computed via the Eyring-Polanyi equation from Transition State Theory.*

ΔG (298.15 K)	**R1 + R2 + cat**	**R1_cat + R2**	**R1_(cat)_R2**	**TS**	**P_cat**	**P + (cat)**	**ΔG‡**	**k (s^{-1})**	**V / X**
uncat *endo*	0.0	-	5.2	36.6	-	-8.4	36.6	9E-15	
BF_3	0.0	-0.8	5.1	20.3	-8.8	-8.4	21.1	8E-03	V
**H_cat** (+)	0.0	-4.3	-0.8	27.9	-10.8	-8.4	32.1	6E-09	V
**H2_cat**	0.0	-0.6	5.8	31.8	-9.6	-8.4	32.4	3E-11	V
**Cl_cat** (+)	0.0	1.9	5.8	32.1	-8.5	-8.4	32.1	2E-11	V
**Br_cat** (+)	0.0	1.7	5.5	31.5	-8.5	-8.4	31.5	5E-11	V
**I_cat** (+)	0.0	-4.3	-0.7	26.2	-11.6	-8.4	30.5	1E-07	V
**S2_rigid_cat**	0.0	2.6	8.2	36.0	-5.0	-8.4	36.0	3E-14	V
**S2_flex_cat**	0.0	3.7	10.8	39.8	-1.8	-8.4	39.8	4E-17	X
**Se_cat**	0.0	5.8	12.0	40.9	-0.1	-8.4	40.9	6E-18	X
**Te_cat**	0.0	4.3	10.2	39.0	-1.5	-8.4	39.0	2E-16	X
**P_cat**	0.0	3.3	11.1	41.3	-4.9	-8.4	41.3	3E-18	X
**As_cat**	0.0	3.2	12.5	40.5	-0.8	-8.4	40.5	1E-17	X
**Sb_cat**	0.0	-0.1	7.8	34.5	-5.0	-8.4	34.5	3E-13	V

2.2. DA reactions with methyl vinyl ketone and cyclopentadiene

Table S7. *Energy at 0 K expressed in kcal/mol for the formed complexes, transition state and products as referred to the separate reagents for the DA reaction between methyl vinyl ketone (R1) and cyclopentadiene (R2) (Reaction2). ΔE‡ refers to the activation energy at 0K.*

ΔE (0 K)	**R1 + R2 + cat**	**R1_cat + R2**	**R1_(cat)_R2**	**TS**	**P_cat**	**P + (cat)**	**ΔE‡**
uncat *endo*	0.0	-	-4.3	14.7	-	-21.9	19.0
BF_3	0.0	-12.9	-19.6	-5.5	-35.4	-21.9	14.1
**H_cat** (+)	0.0	-14.4	-20.2	-4.5	-30.4	-21.9	15.7
**H2_cat**	0.0	-13.0	-19.9	-3.5	-35.8	-21.9	16.4
**Cl_cat** (+)	0.0	-9.6	-14.7	2.2	-31.3	-21.9	16.9
**Br_cat** (+)	0.0	-10.9	-16.1	0.4	-32.5	-21.9	16.5
**I_cat** (+)	0.0	-14.2	-20.0	-4.3	-36.7	-21.9	15.7
**I3_cat**	0.0	-10.4	-16.0	-0.8	-34.1	-21.0	15.3
**S2_rigid_cat**	0.0	-9.1	-13.6	3.9	-29.9	-21.9	17.5
**S2_flex_cat**	0.0	-5.9	-11.7	7.2	-27.6	-21.9	19.9
**Se_cat**	0.0	-5.9	-10.8	6.8	-27.8	-21.9	17.6
**Te_cat**	0.0	-7.7	-13.1	4.1	-30.1	-21.9	17.2
**P_cat**	0.0	-5.1	-9.8	10.4	-26.7	-21.9	20.2
**As_cat**	0.0	-3.3	-7.8	9.7	-25.2	-21.9	17.6
**Sb_cat**	0.0	-8.3	-13.4	3.6	-30.8	-21.9	17.0

Table S8. *Gibbs free energy at 298.15 K expressed in kcal/mol for the formed complexes, transition state and products as referred to the separate reagents for the DA reaction between methyl vinyl ketone (R1) and cyclopentadiene (R2) (Reaction2). ΔG‡ refers to the Gibbs free energy of activation at 298.15K; k (s^{-1}) refers to the rate constant of the reaction (at the standard concentration of 1 mol L-1), computed via the Eyring-Polanyi equation from Transition State Theory.*

ΔG (298.15 K)	**R1 + R2 + cat**	**R1_cat + R2**	**R1_(cat)_R2**	**TS**	**P_cat**	**P + (cat)**	**ΔG‡**	**k (s^{-1})**	**V / X**
uncat *endo*	0.0	-	5.8	28.0	-	-8.7	28.0	2E-08	
BF_3	0.0	-1.0	4.1	20.5	-9.4	-8.7	21.5	1E-03	V
**H_cat** (+)	0.0	-4.8	0.1	18.8	-7.2	-8.7	23.6	3E-05	V
**H2_cat**	0.0	-3.3	3.7	22.2	-10.2	-8.7	25.5	1E-06	V
**Cl_cat** (+)	0.0	-2.0	4.3	24.1	-8.8	-8.7	26.1	5E-07	V
**Br_cat** (+)	0.0	-1.9	3.5	22.9	-9.9	-8.7	24.8	4E-06	V
**I_cat** (+)	0.0	-5.1	0.4	18.5	-13.3	-8.7	23.6	3E-05	V
**I3_cat**	0.0	1.5	7.2	25.1	-8.7	-8.7	25.1	2E-06	V
**S2_rigid_cat**	0.0	3.4	9.4	29.3	-3.5	-8.7	29.3	2E-09	X
**S2_flex_cat**	0.0	4.7	9.0	31.3	-3.5	-8.7	31.3	7E-11	X
**Se_cat**	0.0	5.2	12.3	32.2	-1.8	-8.7	32.2	2E-11	X
**Te_cat**	0.0	3.4	9.8	29.7	-4.3	-8.7	29.7	1E-09	X
**P_cat**	0.0	6.1	11.4	35.1	-1.5	-8.7	35.1	1E-13	X
**As_cat**	0.0	8.7	14.6	35.7	1.4	-8.7	35.7	4E-14	X
**Sb_cat**	0.0	3.8	9.5	29.8	-4.2	-8.7	29.8	9E-10	X

2.3. DA reactions with methylene imine and 1,3-butadiene

Table S9. *Energy at 0 K expressed in kcal/mol for the formed complexes, transition state and products as referred to the separate reagents for the DA reaction between methylene imine (R1) and 1,3-butadiene (R2) (Reaction3). ΔE‡ refers to the activation energy at 0K.*

ΔE (0 K)	**R1 + R2 + cat**	**R1_cat + R2**	**R1_(cat)_R2**	**TS**	**P_cat**	**P + (cat)**	**ΔE‡**
uncat *endo*	0.0	-	-2.8	18.1	-	-34.9	20.9
BF_3	0.0	-20.5	-26.2	-10.4	-62.5	-34.9	15.8
**H_cat** (+)	0.0	-11.4	-17.1	6.2	-47.4	-34.9	23.1
**H2_cat**	0.0	-11.7	-19.4	5.0	-42.2	-34.9	24.4
**Cl_cat** (+)	0.0	-8.2	-13.1	10.1	-44.1	-34.9	23.1
**Br_cat** (+)	0.0	-10.2	-15.0	7.5	-46.9	-34.9	22.5
**I_cat** (+)	0.0	-14.3	-19.2	1.3	-53.1	-34.9	15.6
**S2_rigid_cat**	0.0	-6.2	-13.9	10.6	-43.1	-34.9	16.8
**S2_flex_cat**	0.0	-4.3	-10.7	12.4	-36.3	-34.9	16.7
**Se_cat**	0.0	-5.3	-9.7	11.6	-42.1	-34.9	16.9
**Te_cat**	0.0	-7.7	-12.3	9.2	-45.9	-34.9	16.9
**P_cat**	0.0	-6.6	-12.2	10.2	-37.9	-34.9	16.8
**As_cat**	0.0	-7.5	-13.4	9.4	-44.0	-34.9	22.8
**Sb_cat**	0.0	-12.1	-18.5	5.1	-48.9	-34.9	23.6

Table S10. *Gibbs free energy at 298.15 K expressed in kcal/mol for the formed complexes, transition state and products as referred to the separate reagents for the DA reaction between methylene imine (R1) and 1,3-butadiene (R2) (Reaction3). ΔG‡ refers to the Gibbs free energy of activation at 298.15K; k (s^{-1}) refers to the rate constant of the reaction (at the standard concentration of 1 mol L-1), computed via the Eyring-Polanyi equation from Transition State Theory.*

ΔG (298.15 K)	**R1 + R2 + cat**	**R1_cat + R2**	**R1_(cat)_R2**	**TS**	**P_cat**	**P + (cat)**	**ΔG‡**	**k (s^{-1})**	**V / X**
uncat *endo*	0.0	-	4.6	29.5	-	-23.1	29.5	2E-09	
BF_3	0.0	-9.2	-4.4	13.9	-38.0	-23.1	23.1	7E-05	V
**H_cat** (+)	0.0	-2.6	3.1	27.5	-26.4	-23.1	30.1	5E-10	X
**H2_cat**	0.0	-2.6	1.1	28.0	-19.6	-23.1	30.6	2E-10	X
**Cl_cat** (+)	0.0	0.5	5.2	30.2	-22.9	-23.1	30.2	5E-10	X
**Br_cat** (+)	0.0	-1.1	2.6	28.7	-25.3	-23.1	29.8	9E-10	X
**I_cat** (+)	0.0	-4.8	-1.0	22.6	-31.7	-23.1	27.4	5E-08	V
**S2_rigid_cat**	0.0	3.1	6.3	33.0	-18.7	-23.1	33.0	4E-12	X
**S2_flex_cat**	0.0	4.1	7.9	34.3	-13.5	-23.1	34.3	5E-13	X
**Se_cat**	0.0	4.1	9.4	34.0	-19.4	-23.1	34.0	8E-13	X
**Te_cat**	0.0	2.4	6.9	32.2	-22.4	-23.1	32.2	2E-11	X
**P_cat**	0.0	3.7	8.2	33.7	-14.3	-23.1	33.7	1E-12	X
**As_cat**	0.0	2.9	7.3	32.7	-20.3	-23.1	32.7	7E-12	X
**Sb_cat**	0.0	-1.8	1.5	28.2	-25.5	-23.1	29.7	6E-10	X

3. Correlation between HOMO – LUMO energy gap and the activation energy

Table S11. *Summary of the (un)catalyzed dienophile's virtual orbitals and their energy, involved in the formation of the Diels-Alder products. The energy gap is calculated as the difference between this virtual orbital's energy and the HOMO energy of the corresponding dienes. BEN = benzaldehyde; MVK = methyl vinyl ketone; IM = methylene imine.*

catalyst	BEN	E_{virt}	ΔE_{gap}	MVK	E_{virt}	ΔE_{gap}	IM	E_{virt}	ΔE_{gap}
uncat	LUMO	-0.76	-6.93	LUMO	-0.44	-6.92	LUMO	1.09	-8.85
BF_3	LUMO	-2.21	-5.48	LUMO	-2.12	-5.24	LUMO	-1.19	-6.57
**H_cat** (+)	LUMO	-3.68	-4.01	LUMO	-3.57	-3.79	LUMO+1	-2.67	-5.09
**H2_cat**	LUMO	-1.48	-6.21	LUMO	-1.83	-5.53	LUMO+1	-0.85	-6.91
**Cl_cat** (+)	LUMO+1	-3.62	-4.07	LUMO+1	-2.88	-4.48	LUMO+2	-1.85	-5.91
**Br_cat** (+)	LUMO	-3.69	-4.00	LUMO+1	-3.09	-4.27	LUMO+3	-2.12	-5.64
**I_cat** (+)	LUMO	-3.49	-4.20	LUMO	-3.42	-3.94	LUMO+2	-2.66	-5.10
**S2_rigid_cat**	LUMO+1	-1.18	-6.51	LUMO+1	-0.92	-6.44	LUMO+2	-0.28	-7.48
**S2_flex_cat**	LUMO+1	-1.21	-6.48	LUMO+1	-1.09	-6.27	LUMO+2	0.26	-8.02
**Se_cat**	LUMO	-1.09	-6.60	LUMO	-0.83	-6.53	LUMO+2	0.19	-7.95
**Te_cat**	LUMO	-1.28	-6.41	LUMO	-1.06	-6.3	LUMO+3	-0.12	-7.64
**P_cat**	LUMO	-1.27	-6.42	LUMO+1	-0.77	-6.59	LUMO+3	0.10	-7.86
**As_cat**	LUMO	-1.21	-6.48	LUMO	-1.02	-6.34	LUMO+3	0.08	-7.84
**Sb_cat**	LUMO	-1.45	-6.24	LUMO	-1.25	-6.11	LUMO	0.02	-7.78

When a catalyst is included, the bond formation does not always involve the LUMO of the dienophile, and this strongly depends on the type of dienophile. Examples are given in the figure below.

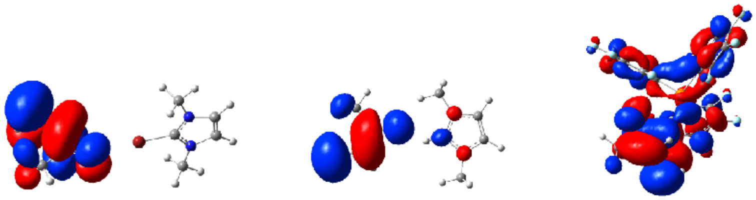

a b c

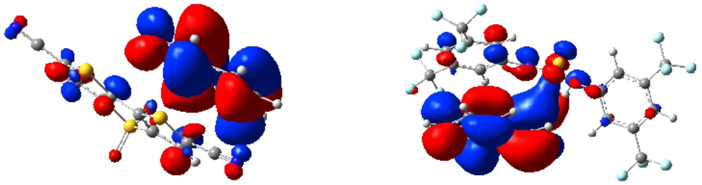

d e

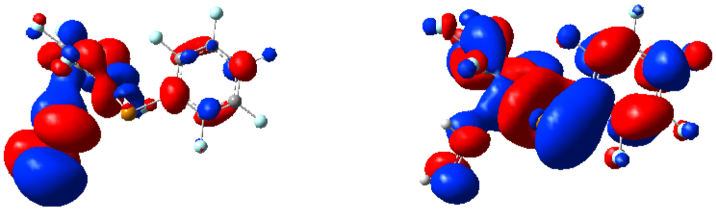

f

Figure S1. *Examples of virtual orbitals involved in the formation of the Diels-Alder products: a) LUMO+1 of methyl vinyl ketone in interaction (**MVK**) with **Br_cat**; b) LUMO of MVK in interaction with **H_cat**; c) LUMO+1 of **MVK** in interaction with **P_cat**; d) LUMO+1 of benzaldehyde in interaction with **S2_rigid_cat**; e) LUMO of benzaldehyde in interaction with **H2_cat**; f) LUMO and LUMO+2 of methylene imine in interaction with **Te_cat**.*

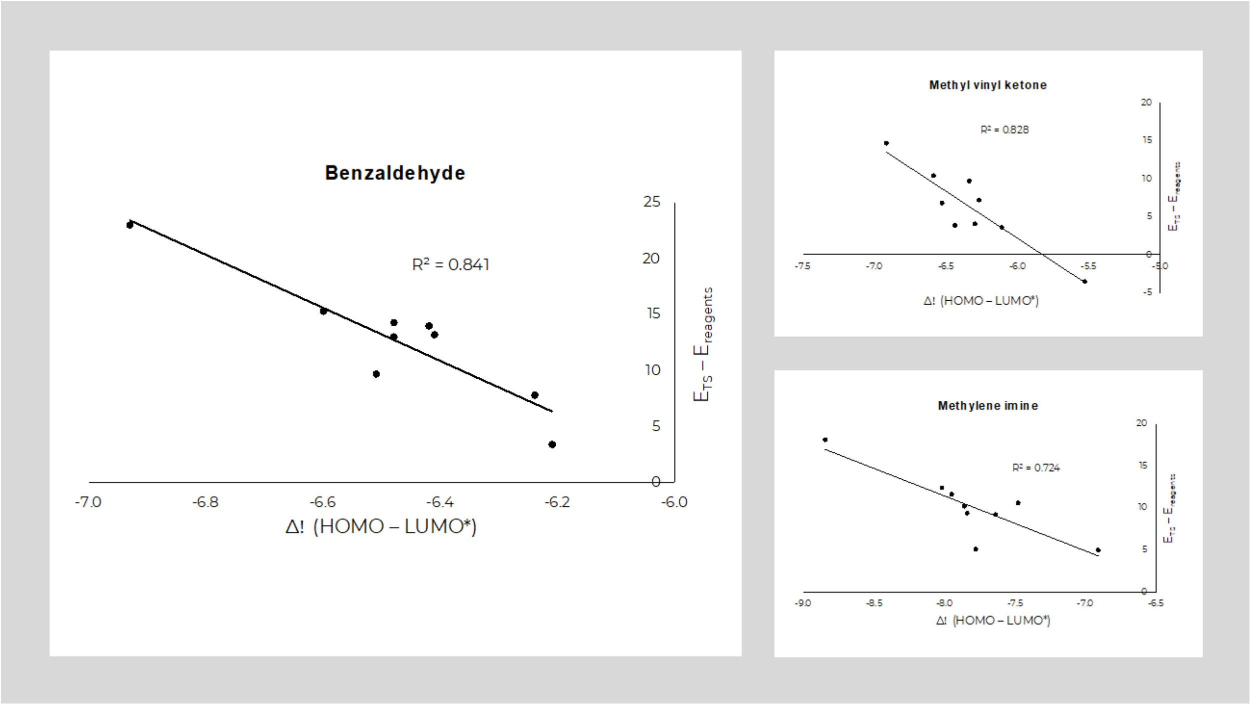

Figure S2. *Linear correlation plots for the orbital energy differences between the diene's HOMO and the dienophile's LUMO (or a virtual orbital of higher order, see Table S11) in eV versus the activation energies at 0K, computed with respect to the separate reagents, in kcal/mol. Only uncharged, non-covalent catalysts were considered.*

4. Activation strain model / energy decomposition analysis (PBE-D3/TZP results)

4.1. DA reactions with methyl vinyl ketone and cyclopentadiene

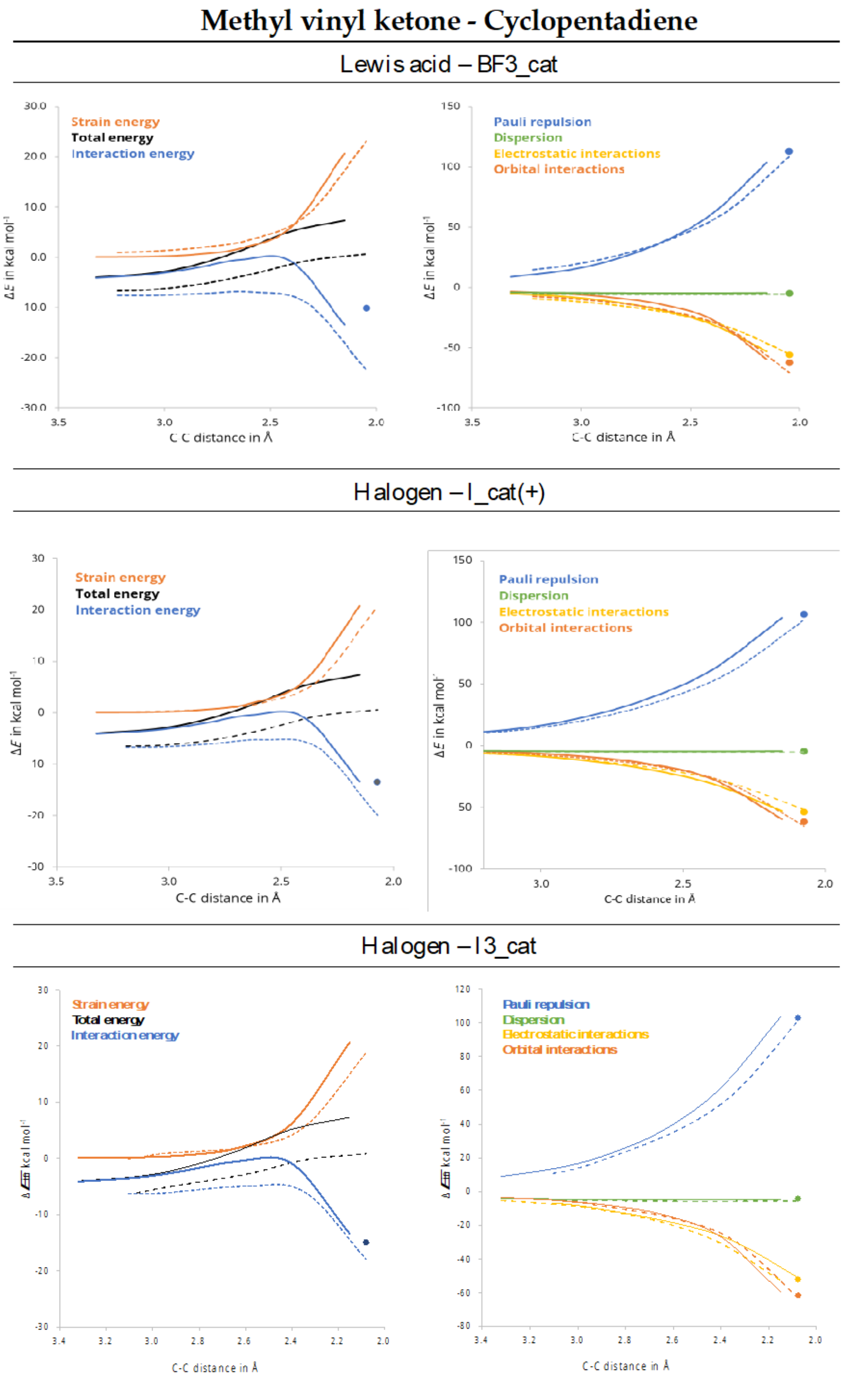

Figure S3. *ASM (left) and EDA (right) analysis of the uncatalyzed and Lewis-acid- and halogen-bond-catalyzed reactions between methyl vinyl ketone and cyclopentadiene. The full line represents the reaction without catalyst and the dashed line represents the reaction with catalyst. The dots represent the values for the transition state geometry of the catalyzed reaction with the catalyst deleted.*

Methyl vinyl ketone - Cyclopentadiene

Hydrogen – H_cat(+)

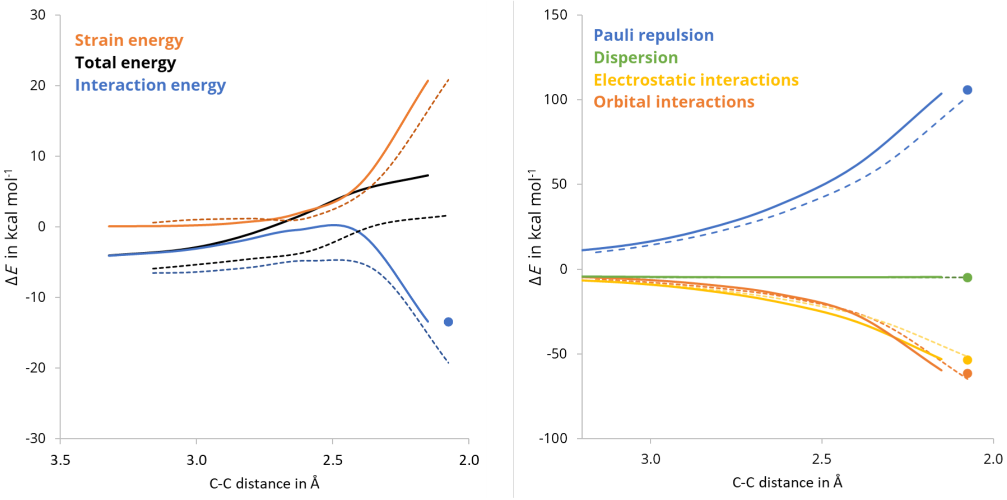

Hydrogen – H2_cat

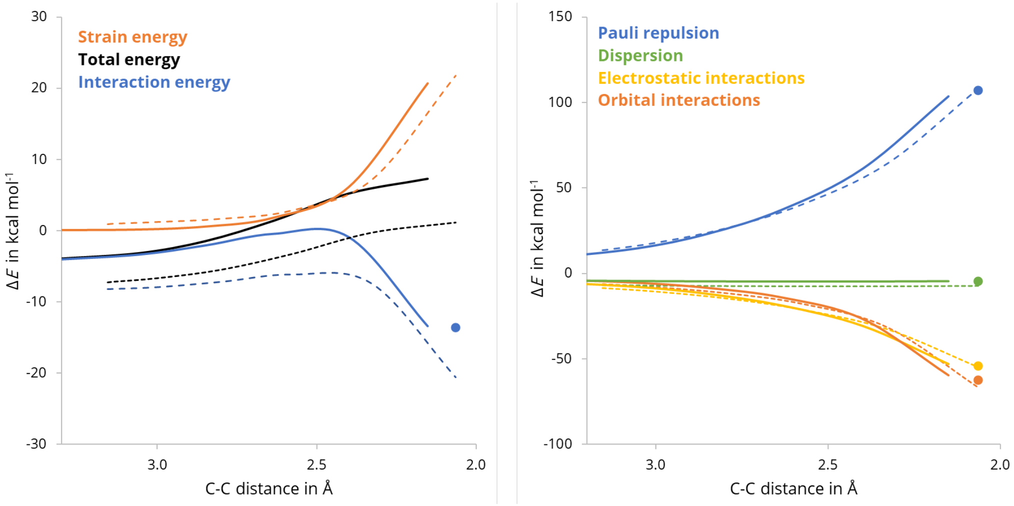

Figure S4. *ASM (left) and EDA (right) analysis of the uncatalyzed and hydrogen-bond-catalyzed reactions between methyl vinyl ketone and cyclopentadiene. The full line represents the reaction without catalyst and the dashed line represents the reaction with catalyst. The dots represent the values for the transition state geometry of the catalyzed reaction with the catalyst deleted.*

4.2. DA reactions with methylene imine and 1,3-butadiene

Methylene imine – 1,3-Butadiene

Lewis acid – BF3_cat

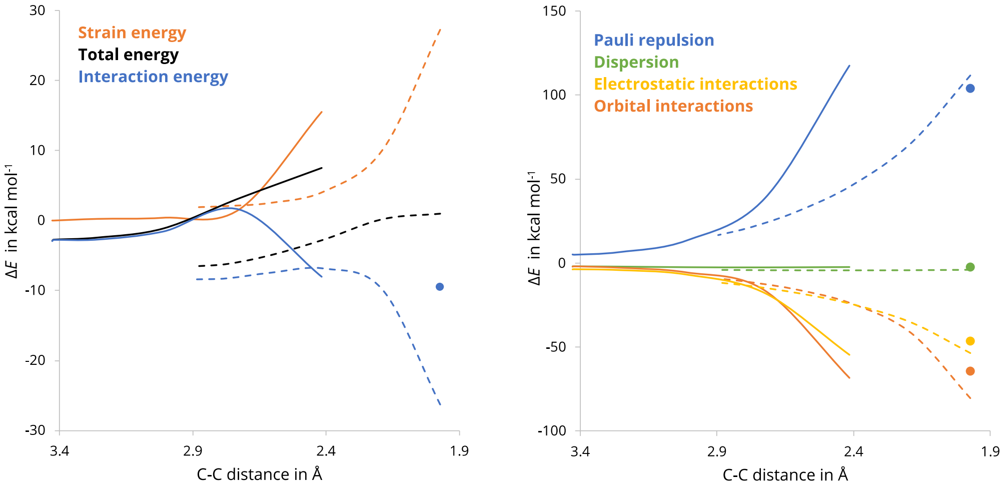

Halogen – I_cat(+)

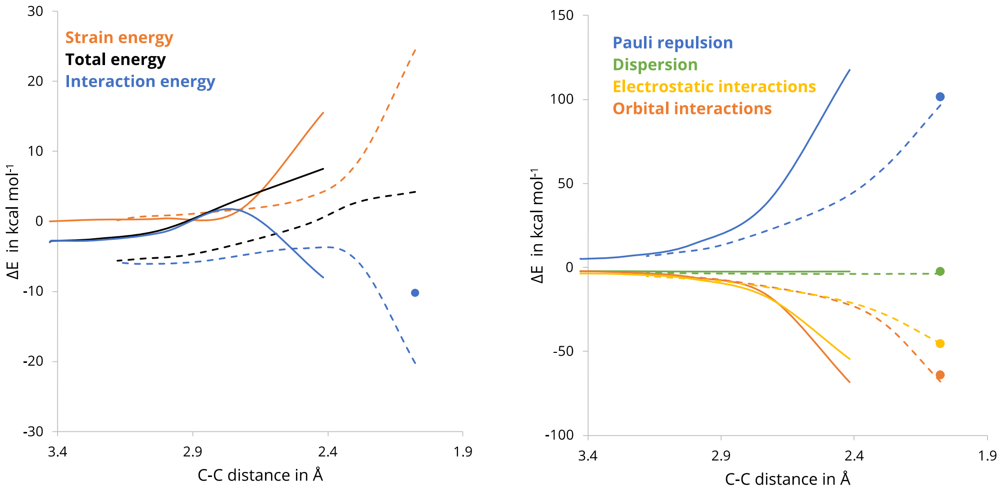

Figure S5. *ASM (left) and EDA (right) analysis of the uncatalyzed and Lewis-acid- and halogen-bond-catalyzed reactions between methylene imine and 1,3-butadiene. The full line represents the reaction without catalyst and the dashed line represents the reaction with catalyst. The dots represent the values for the transition state geometry of the catalyzed reaction with the catalyst deleted.*

5. Energy decomposition analysis at catalyzed TS: in absence and presence of catalyst (PBE-D3/TZ2P results)

5.1. DA reactions with methyl vinyl ketone and cyclopentadiene

Table S12. *EDA analysis of the transition state for the uncatalyzed and catalyzed reaction between methyl vinyl ketone and cyclopentadiene, the latter in presence and absence of the catalyst. All values are given in kcal/mol. The values in brackets represent the relative contribution of the given energies to the stabilizing part of the total interaction energy.*

	ΔE_{int}	ΔE_{Pauli}	ΔE_{elst}	ΔE_{oi}	ΔE_{disp}
Uncatalyzed	-13.4	103.8	-52.9 (**45**)	-59.7 (**51**)	-4.6 (**4**)
BF_3	-22.4	108.6	-54.9 (**42**)	-70.5 (**54**)	-5.7 (**4**)
BF_3 deleted	-10.1	113.0	-55.8 (**45**)	-62.4 (**51**)	-4.8 (**4**)
**H_cat** (+)	-19.2	101.7	-51.5 (**43**)	-64.7 (**54**)	-4.7 (**4**)
**H_cat** deleted	-13.4	105.9	-53.4 (**45**)	-61.3 (**51**)	-4.5 (**4**)
**H2_cat**	-20.7	108.2	-55.1 (**43**)	-66.6 (**52**)	-7.2 (**6**)
**H2_cat** deleted	-13.6	107.3	-54.1 (**45**)	-62.3 (**51**)	-4.5 (**4**)
**Cl_cat** (+)	-17.2	101.8	-51.8 (**43**)	-62.6 (**53**)	-4.7 (**4**)
**Cl_cat** deleted	-13.4	104.7	-53.1 (**45**)	-60.5 (**51**)	-4.5 (**4**)
**Br_cat** (+)	-18.0	101.5	-51.6 (**43**)	-63.2 (**53**)	-4.7 (**4**)
**Br_cat** deleted	-13.3	104.9	-53.1 (**45**)	-60.6 (**51**)	-4.5 (**4**)
**I_cat** (+)	-20.0	102.2	-51.7 (**42**)	-65.5 (**54**)	-5.1 (**4**)
**I_cat** deleted	-13.4	106.5	-53.7 (**45**)	-61.7 (**51**)	-4.5 (**4**)
**I3_cat**	-17.9	101.2	-51.1 (**43**)	-62.7 (**53**)	-5.4 (4)
**I3_cat** deleted	-14.9	103.0	-52.0 (**44**)	-61.6 (**52**)	-4.3 (**4**)

5.2. DA reactions with methylene imine and 1,3-butadiene

Table S13. *EDA analysis of the transition state for the uncatalyzed and catalyzed reaction between methylene imine and 1,3-butadiene, the latter in presence and absence of the catalyst. All values are given in kcal/mol. The values in brackets represent the relative contribution of the given energies to the stabilizing part of the total interaction energy.*

	ΔE_{int}	ΔE_{Pauli}	ΔE_{elst}	ΔE_{oi}	ΔE_{disp}
Uncatalyzed	-8.0	117.4	-54.6 (**43**)	-68.4 (**55**)	-2.4 (**2**)
BF_3	-26.3	111.7	-53.5 (**39**)	-80.5 (**58**)	-4.0 (**3**)
BF_3 deleted	-12.1	111.2	-51.6 (**42**)	-69.4 (**56**)	-2.4 (**2**)
**I_cat** (+)	-20.2	96.7	-45.3 (**39**)	-67.9 (**58**)	-3.7 (**3**)
**I_cat** deleted	-12.5	99.8	-46.7 (**42**)	-63.1 (**56**)	-2.5 (**2**)

6. Activation strain model / energy decomposition analysis (M06-2X/TZ2P results)

6.1. DA reactions with benzaldehyde and 2,3-dimethyl 1,3-butadiene

Benzaldehyde - Butadiene

Lewis catalyst – BF3_cat

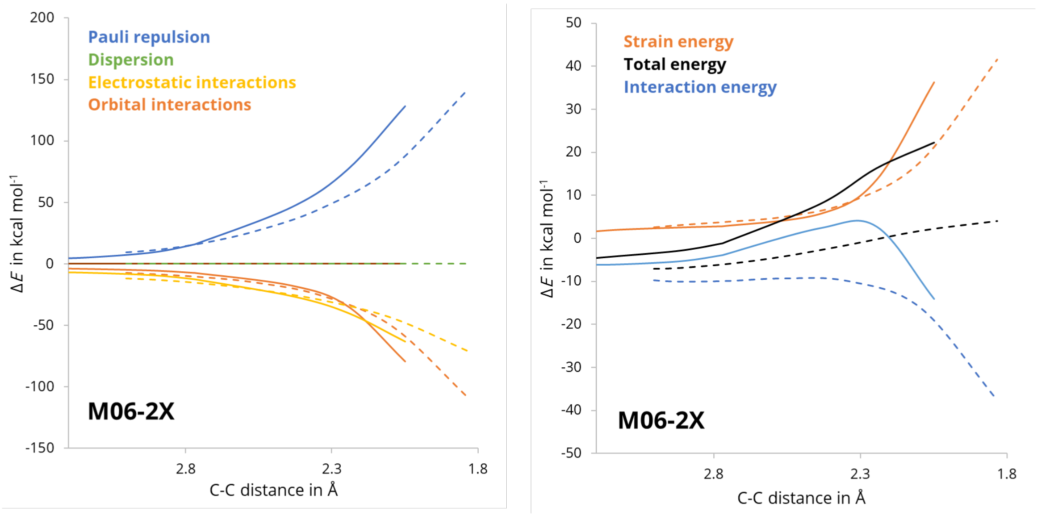

Iodine catalyst - I_cat(+)

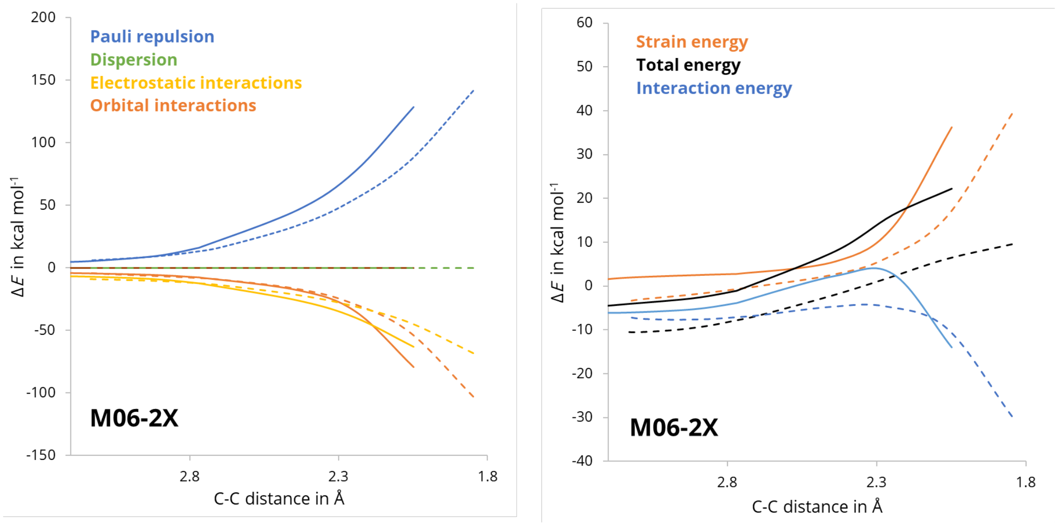

Figure S6. *ASM (left) and EDA (right) analysis of the uncatalyzed and Lewis-acid- and halogen-bond-catalyzed reactions between benzaldehyde and 2,3-dimethyl 1,3-butadiene. The full line represents the reaction without catalyst and the dashed line represents*

the reaction with catalyst. The dots represent the values for the transition state geometry of the catalyzed reaction with the catalyst deleted.

Benzaldehyde - 2,3-dimethyl 1,3-butadiene

Hydrogen – H_cat (+)

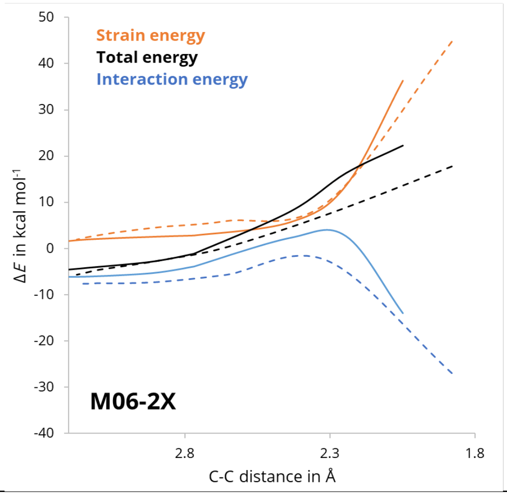

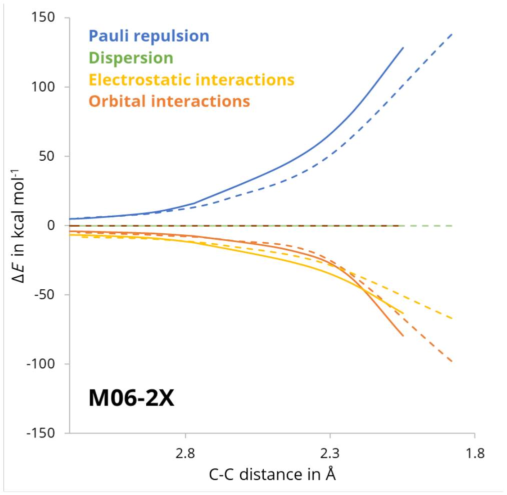

Hydrogen – H2_cat

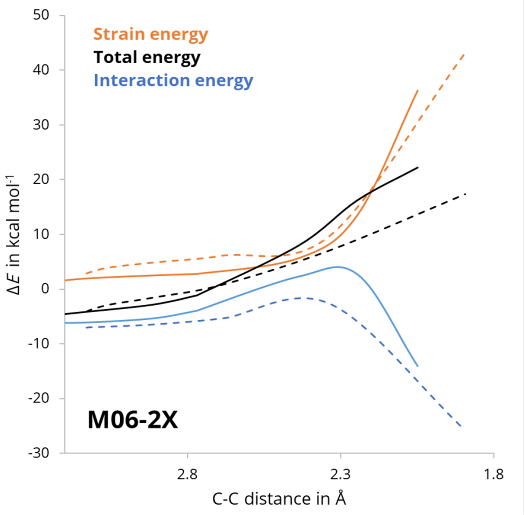

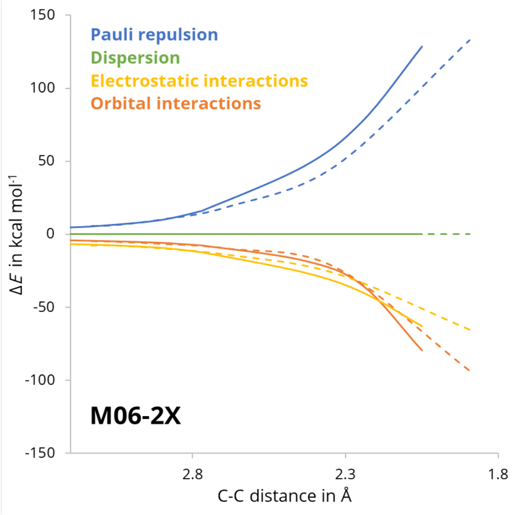

Figure S7. *ASM (left) and EDA (right) analysis of the uncatalyzed and hydrogen-bond-catalyzed reactions between benzaldehyde and 2,3-dimethyl 1,3-butadiene. The full line represents the reaction without catalyst and the dashed line represents the reaction with catalyst. The dots represent the values for the transition state geometry of the catalyzed reaction with the catalyst deleted.*

Benzaldehyde - 2,3-dimethyl 1,3-butadiene

Chalcogen – S2_rigid_cat

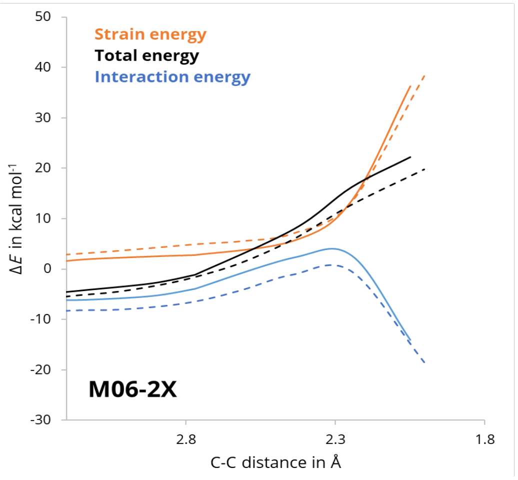

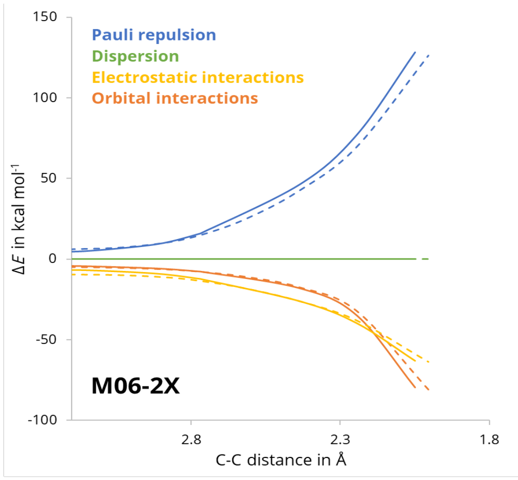

Pnictogen – Sb_cat

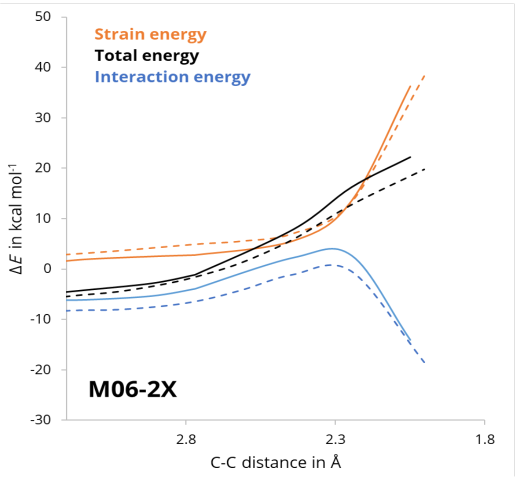

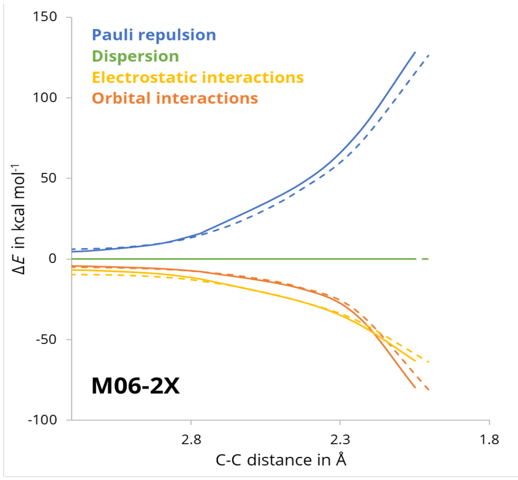

Figure S8. *ASM (left) and EDA (right) analysis of the uncatalyzed and chalcogen and pnictogen catalyzed reactions between benzaldehyde and 2,3-dimethyl 1,3-butadiene. The full line represents the reaction without catalyst and the dashed line represents the reaction with catalyst. The dots represent the values for the transition state geometry of the catalyzed reaction with the catalyst deleted.*

6.2. DA reactions with methyl vinyl ketone and cyclopentadiene

Methyl vinyl ketone - Cyclopentadiene

Lewis catalyst – $BF3\_cat$

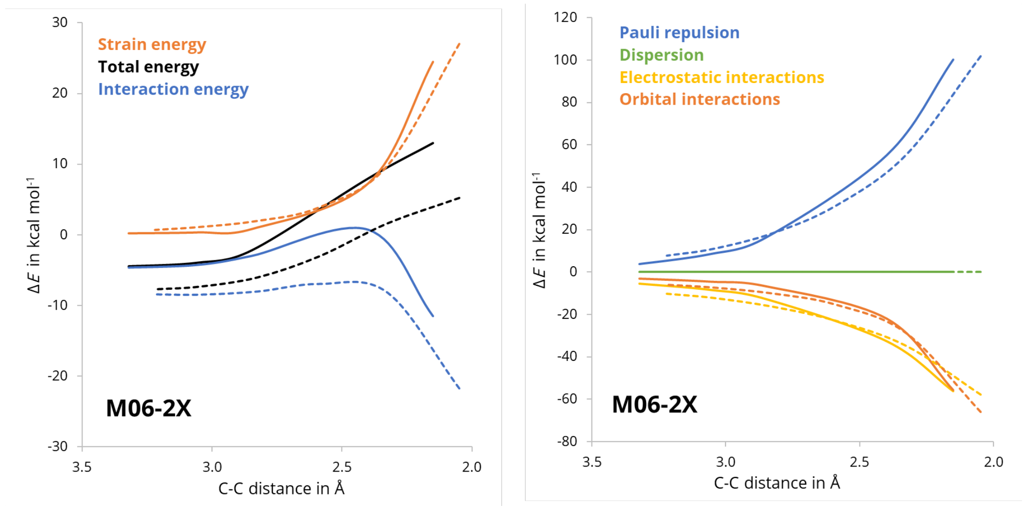

Iodine catalyst - I_cat(+)

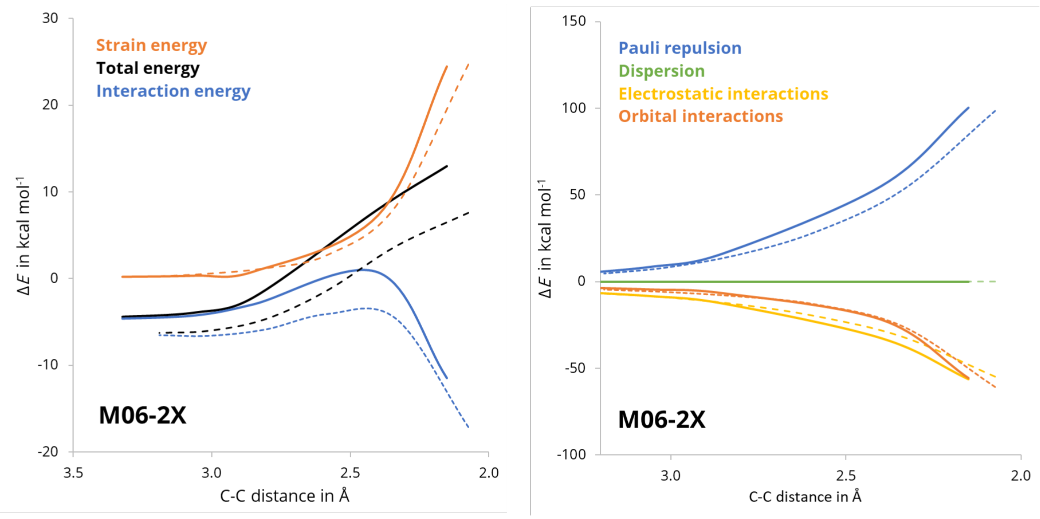

Figure S9. *ASM (left) and EDA (right) analysis of the uncatalyzed and Lewis-acid- and halogen-bond-catalyzed reactions between methyl vinyl ketone and cyclopentadiene. The full line represents the reaction without catalyst and the dashed line represents the reaction with catalyst. The dots represent the values for the transition state geometry of the catalyzed reaction with the catalyst deleted.*

Hydrogen bonding – H_cat(+)

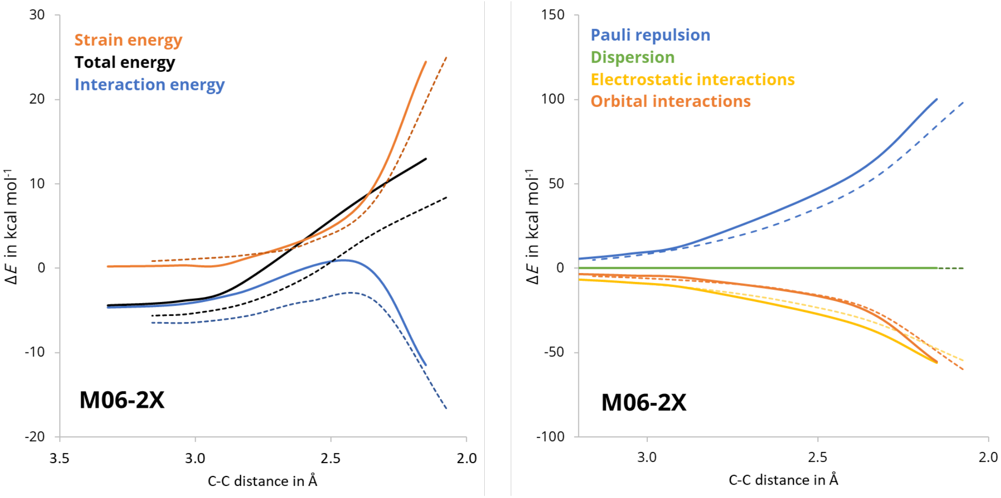

Hydrogen bonding – H2_cat

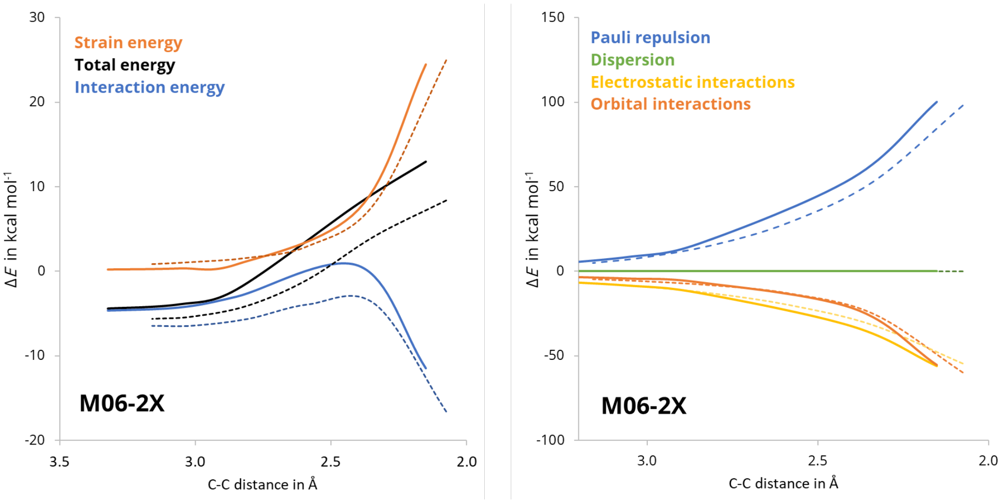

Figure S10. *ASM (left) and EDA (right) analysis of the uncatalyzed and hydrogen-bond-catalyzed reactions between methyl vinyl ketone and cyclopentadiene. The full line represents the reaction without catalyst and the dashed line represents the reaction with catalyst. The dots represent the values for the transition state geometry of the catalyzed reaction with the catalyst deleted.*

6.3. DA reactions with methylene imine and 1,3-butadiene

Imine – 1,3-Butadiene

Lewis catalyst – $BF3\_cat$

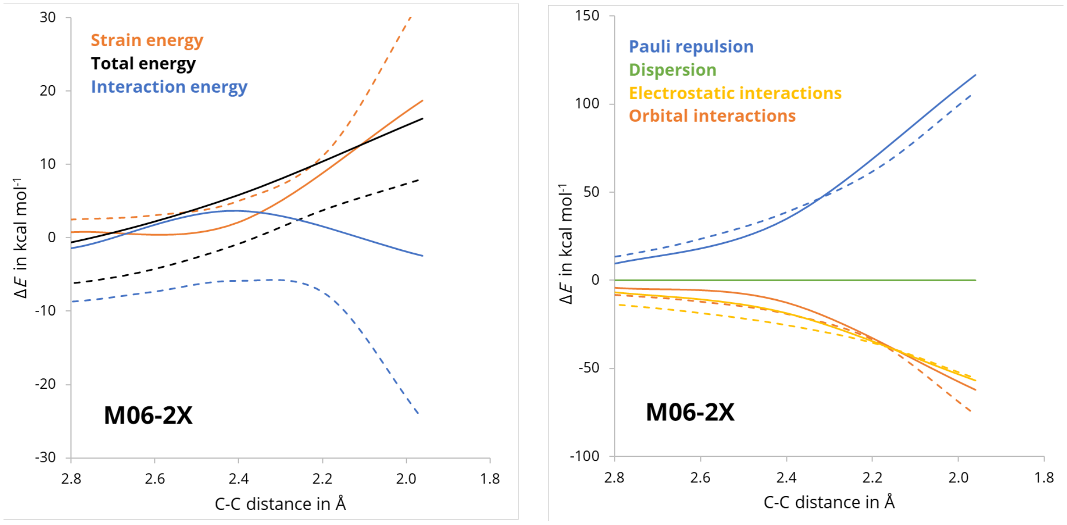

Iodine catalyst - I_cat(+)

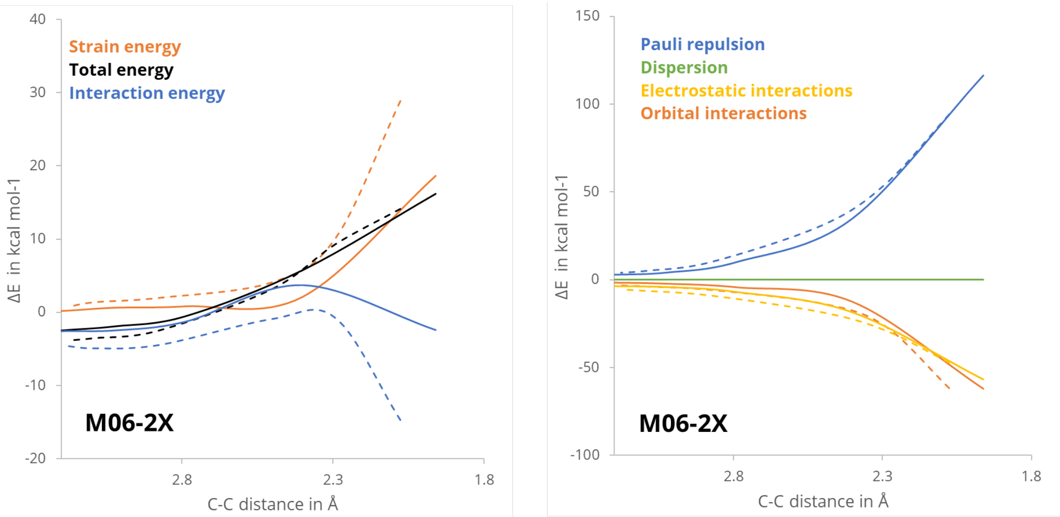

Figure S11. *ASM (left) and EDA (right) analysis of the uncatalyzed and Lewis-acid- and halogen-bond-catalyzed reactions between methylene imine and 1,3-butadiene. The full line represents the reaction without catalyst and the dashed line represents the reaction with catalyst. The dots represent the values for the transition state geometry of the catalyzed reaction with the catalyst deleted.*

7. Energy decomposition analysis at catalyzed TS: in absence and presence of catalyst (M06-2X/TZ2P results)

7.1. DA reactions with benzaldehyde and 2,3-dimethyl 1,3-butadiene

Table S14. *EDA analysis of the transition state for the uncatalyzed and catalyzed reaction between benzaldehyde and 2,3-dimethyl 1,3-butadiene, the latter in presence and absence of the catalyst. All values are given in kcal/mol. The values in brackets represent the relative contribution of the given energies to the stabilizing part of the total interaction energy.*

	ΔE_{int}	ΔE_{Pauli}	ΔE_{elst}	ΔE_{oi}	ΔE_{disp}
Uncatalyzed	-14.0	128.4	-63.1 **(44)**	-79.4 **(56)**	0 **(0)**
BF_3	-37.6	142.1	-70.9 **(40)**	-108.8 **(60)**	0 **(0)**
BF_3 deleted	-12.8	140.7	-66.9 **(44)**	-86.6 **(56)**	0 **(0)**
**H_cat** (+)	-27.0	137.8	-67.0 **(41)**	-97.8 **(59)**	0 **(0)**
**H_cat** deleted	-18.6	139.4	-67.7 **(43)**	-90.4 **(57)**	0 **(0)**
**H2_cat**	-25.7	132.9	-65.2 **(41)**	-93.5 **(59)**	0 **(0)**
**H2_cat** deleted	-17.7	134.4	-65.2 **(43)**	-86.9 **(57)**	0 **(0)**
**I_cat** (+)	-29.7	141.2	-68.3 **(40)**	-102.7 **(60)**	0 **(0)**
**I_cat** deleted	-16.3	144.6	-69.5 **(43)**	-91.4 **(57)**	0 **(0)**
**Sb_cat**	-21.0	127.5	-63.4 **(43)**	-85.1 **(57)**	0 **(0)**
**Sb_cat** deleted	-15.1	128.9	-62.9 **(44)**	-81.3 **(56)**	0 **(0)**
**S2_rigid_cat**	-18.4	126.1	-63.6 **(44)**	-81.0 **(56)**	0 **(0)**
**S2_rigid_cat** deleted	-14.6	126.7	-62.1 **(44)**	-79.2 **(56)**	0 **(0)**

7.2. DA reactions with methyl vinyl ketone and cyclopentadiene

Table S15. *EDA analysis of the transition state for the uncatalyzed and catalyzed reaction between methyl vinyl ketone and cyclopentadiene, the latter in presence and absence of the catalyst. All values are given in kcal/mol. The values in brackets represent the relative contribution of the given energies to the stabilizing part of the total interaction energy.*

	ΔE_{int}	ΔE_{Pauli}	ΔE_{elst}	ΔE_{oi}	ΔE_{disp}
Uncatalyzed	-11.5	100.2	-56.2 **(50)**	-55.5 **(50)**	0 **(0)**
BF_3	-21.8	101.9	-57.8 **(47)**	-65.9 **(53)**	0 **(0)**
BF_3 deleted	-8.1	108.3	-58.9 **(51)**	-57.5 **(49)**	0 **(0)**
**H_cat** (+)	-16.6	98.0	-54.7 **(48)**	-60.0 **(52)**	0 **(0)**
**H_cat** deleted	-11.0	102.3	-56.5 **(50)**	-56.8 **(50)**	0 **(0)**
**H2_cat**	-18.1	101.7	-58.0 **(48)**	-61.8 **(52)**	0 **(0)**

**H2_cat** deleted	-11.2	103.8	-57.2 **(50)**	-57.9 **(50)**	0 **(0)**
**I_cat** (+)	-17.1	98.0	-54.7 **(48)**	-60.0 **(52)**	0 **(0)**
**I_cat** deleted	-11.1	102.8	-56.8 **(50)**	-57.1 **(50)**	0 **(0)**

7.3. DA reactions with methylene imine and 1,3 butadiene

Table S16. *EDA analysis of the transition state for the uncatalyzed and catalyzed reaction between methylene imine and 1,3-butadiene, the latter in presence and absence of the catalyst. All values are given in kcal/mol. The values in brackets represent the relative contribution of the given energies to the stabilizing part of the total interaction energy.*

	ΔE_{int}	ΔE_{Pauli}	ΔE_{elst}	ΔE_{oi}	ΔE_{disp}
Uncatalyzed	-2.4	116.4	-56.8 **(48)**	-62.1 **(52)**	0 **(0)**
BF_3	-23.9	104.7	-54.5 **(42)**	-74.1 **(58)**	0 **(0)**
BF_3 deleted	-6.7	109.9	-53.3 **(46)**	-63.3 **(54)**	0 **(0)**
**I_cat** (+)	-14.7	94.2	-47.3 **(43)**	-61.6 **(57)**	0 **(0)**
**I_cat** deleted	-7.5	98.3	-48.4 **(46)**	-57.4 **(54)**	0 **(0)**

8. XYZ coordinates

CATALYSTS

BF3

B	0.00003300	0.00046700	0.00021700
F	-1.15098800	0.64505400	-0.00004000
F	1.13443300	0.67369500	-0.00004000
F	0.01653700	-1.31900800	-0.00004000

H2_cat

C	5.20689100	-0.03855800	0.07885100
C	4.32618400	-1.10211300	-0.11568800
C	4.70538200	1.25431400	0.01684100
H	6.26408500	-0.21803000	0.26870200
C	2.97700800	-0.89565700	-0.37480300
C	4.85103500	-2.51058100	-0.02086200
C	3.35075700	1.48567500	-0.21180700
C	5.62081100	2.43800400	0.16844700
C	2.48149000	0.41321900	-0.41053800
H	2.31466800	-1.73958400	-0.54796300
F	6.10997800	-2.59349100	-0.47348900
F	4.86133900	-2.94390200	1.24583600
F	4.10555800	-3.36767100	-0.72830500
H	2.97349300	2.50989400	-0.22584400
F	6.78145800	2.10289900	0.74001400
F	5.90512700	2.98975500	-1.02082300
F	5.05734500	3.39990300	0.91404500
N	1.13440900	0.70479000	-0.71355300
C	0.00370600	0.08456300	-0.23426800
H	1.00456200	1.59782800	-1.17806700
S	0.01012700	-1.20993600	0.80580300
N	-1.12965600	0.69397600	-0.71778200
H	-1.00192000	1.57252100	-1.20966100
C	-2.47771400	0.40442400	-0.41757400
C	-3.35374000	1.48279000	-0.27722800
C	-2.97080900	-0.90214800	-0.33909900
C	-4.70855600	1.25772400	-0.05449100
H	-2.98190300	2.50644900	-0.34528800
C	-4.32602100	-1.10160500	-0.09830600
H	-2.30534200	-1.75063300	-0.47273100
C	-5.21088800	-0.03488500	0.03902100
C	-5.62849700	2.43385600	0.12966500
C	-4.84803000	-2.50915400	0.02534900
H	-6.27385500	-0.20954400	0.20030200
F	-6.87276300	2.15603400	-0.27554700
F	-5.70428100	2.80214200	1.41531400
F	-5.20256200	3.50394500	-0.55856200
F	-4.83173100	-2.92673300	1.29715800
F	-4.11473800	-3.37432800	-0.68542000
F	-6.11507900	-2.59971900	-0.40177500

Cl_cat

C	-0.68037400	-1.83661100	-0.00001500
C	0.68101300	-1.83636700	-0.00011700
C	-0.00003300	0.25367200	-0.00001800
N	-1.09096300	-0.51880100	-0.00010000
N	1.09117200	-0.51843900	-0.00013100
H	-1.39097000	-2.65602700	-0.00019300
H	1.39182600	-2.65559600	-0.00038000
C	-2.47308900	-0.03085700	0.00021900
H	-2.64853700	0.57098700	0.89959800
H	-2.64941000	0.56987100	-0.89974300
H	-3.13494600	-0.90186000	0.00110400
C	2.47314000	-0.03009900	0.00025700
H	2.64934000	0.57071200	-0.89967500
H	2.64840500	0.57175900	0.89966300
H	3.13525000	-0.90090800	0.00112300
Cl	-0.00037400	1.93960700	-0.00010800

Br_cat

C	2.29850000	-0.68075300	-0.00024200
C	2.29846500	0.68086700	-0.00003000
C	0.20502500	0.00000600	-0.00061600
N	0.98144000	-1.09022200	-0.00042200
N	0.98138500	1.09027100	0.00000200
H	3.11807800	-1.39121600	-0.00022300
H	3.11800500	1.39137300	0.00014600
C	0.50260400	-2.47501100	0.00067400
H	-0.08594900	-2.65871000	0.90740900
H	-0.10960300	-2.65138300	-0.89165000
H	1.37764100	-3.13153200	-0.01380000
C	0.50243600	2.47503200	0.00046100
H	1.37751800	3.13163000	-0.00488400
H	-0.10237500	2.65366000	-0.89646900
H	-0.09362300	2.65633300	0.90273900
Br	-1.63376100	-0.00003800	-0.00005200

I_cat

C	-2.69142400	-0.68061900	0.00002600
C	-2.69129700	0.68091800	0.00011600
C	-0.59052500	-0.00006100	-0.00019900
N	-1.37509700	-1.08788600	-0.00047800
N	-1.37490600	1.08795300	-0.00027800
H	-3.51098800	-1.39113400	0.00124400
H	-3.51075900	1.39154600	0.00141500
C	-0.91063100	-2.47647700	0.00005400
H	-0.30767200	-2.66298100	-0.89670400
H	-0.31696900	-2.66561900	0.90248600
H	-1.79182700	-3.12508700	-0.00554300
C	-0.91026700	2.47653700	-0.00004300
H	-0.31315700	2.66451000	0.90033500
H	-0.31069000	2.66394600	-0.89888500
H	-1.79145400	3.12517500	-0.00147400
I	1.46921500	-0.00004900	0.00005100

S2_rigid_cat

C	1.28080700	0.74071000	0.00043700
C	-0.72772500	-0.51831400	0.00096900
C	-1.28080800	0.74071100	0.00086400
C	-3.18223000	-0.53163500	-0.00013600
C	-2.69012300	0.75570800	0.00007600
H	-3.32093500	1.64143300	-0.00017000
C	0.72774000	-0.51831700	0.00079200
C	2.69012200	0.75571800	-0.00025100
H	3.32092300	1.64145100	-0.00062600
C	3.18223800	-0.53162300	-0.00020400
C	-4.55056500	-0.92937400	-0.00069100
C	4.55057200	-0.92936100	-0.00069400
N	-5.66038300	-1.26243200	-0.00115000
N	5.66039900	-1.26239300	-0.00108500
S	-1.92642700	-1.74348400	0.00042500

S	1.92644500	-1.74348500	0.00053100
S	0.00000300	2.01336900	0.00003900
O	0.00027700	2.73599500	1.27359000
O	-0.00035100	2.73440100	-1.27439400

S2_flex_cat

S	-1.70701300	-0.88673100	0.31836300
C	-3.10900900	0.07068100	-0.05785000
C	-0.72568300	0.48937800	-0.05222800
C	-1.53440500	1.54137400	-0.42053000
H	-1.16454900	2.51851400	-0.72698500
C	0.72574200	0.48937400	0.05270100
C	1.53458300	1.54150100	0.42046200
S	1.70687100	-0.88682400	-0.31777200
H	1.16483100	2.51866300	0.72695600
C	3.10904000	0.07058100	0.05795700
N	-2.87409700	1.30192300	-0.41678000
N	2.87421900	1.30195800	0.41652600
C	4.41899900	-0.50491000	-0.03676400
N	5.45991100	-1.00332900	-0.12494400
C	-4.41901000	-0.50486700	0.03615700
N	-5.45996700	-1.00326100	0.12393300

Se_cat

C	1.39840400	-0.74105400	-0.16645600
C	2.41500100	-0.64179800	0.78003300
C	1.41573800	0.12831600	-1.25660000
C	3.43447300	0.29326900	0.63906400
C	2.41880900	1.07663500	-1.40365100
C	3.43179300	1.15227600	-0.45319100
C	-1.39819700	-0.73761300	0.17953800
C	-1.41520100	0.15157600	1.25376600
C	-2.41517300	-0.65566500	-0.76816500
C	-2.41829000	1.10259900	1.38376800
C	-3.43460600	0.28186200	-0.64388000
C	-3.43156400	1.16086100	0.43237100
F	2.42341100	1.90418500	-2.44190600
F	0.45448200	0.07543700	-2.17247300
F	4.39616600	2.04992400	-0.58922700
F	4.40128300	0.37904600	1.54469300
F	2.43471800	-1.43801600	1.84060000
F	-0.45366000	0.11565700	2.17031600
F	-2.42259800	1.94896200	2.40667300
F	-4.39647900	2.06030500	0.55258500
F	-4.40261900	0.35007400	-1.54982900
F	-2.43561800	-1.47125800	-1.81394700
Se	0.00003300	-2.03518900	0.01803200

Te_cat

C	1.51453500	-0.55084200	-0.20466500
C	2.64105000	-0.57238700	0.61089100
C	1.41122300	0.46507200	-1.15140600
C	3.64045000	0.38672800	0.49451500
C	2.39142400	1.44136700	-1.27744300
C	3.50968300	1.39643800	-0.45169300
C	-1.51453900	-0.55066200	0.20477000
C	-1.41100000	0.46559000	1.15110300
C	-2.64125000	-0.57246800	-0.61051800
C	-2.39113700	1.44197800	1.27698800
C	-3.64059300	0.38671100	-0.49429300
C	-3.50957900	1.39677700	0.45151200
F	2.27515800	2.40789000	-2.18060500
F	0.35446400	0.52989200	-1.95957300
F	4.45391700	2.31829900	-0.56916500
F	4.71043700	0.35037400	1.27980200
F	2.78911000	-1.52389000	1.53048000
F	-0.35408600	0.53064100	1.95906500
F	-2.27463600	2.40881200	2.17978400
F	-4.45376700	2.31870400	0.56885100
F	-4.71076600	0.35008700	-1.27930800
F	-2.78954000	-1.52435500	-1.52969600
Te	-0.00008100	-2.00584400	0.00009100

P_cat

C	1.11526600	1.18175300	-0.48148200
C	1.12452600	1.31740100	0.90650900
C	2.07768600	1.87808800	-1.20640400
C	2.04590000	2.12021200	1.55773500
C	3.01710500	2.68989900	-0.57678500
C	2.99691700	2.80767200	0.80600800
C	0.45082300	-1.50079000	-0.47249000
C	1.60781700	-2.10992100	-0.96170800
C	-0.18196100	-2.12253400	0.60278700
C	2.11566000	-3.28738200	-0.43315700
C	0.30024500	-3.30983400	1.14414100
C	1.44975700	-3.89230100	0.62609100
F	3.92737800	3.34571600	-1.28572300
F	2.12653900	1.78265500	-2.53135400
F	3.88400400	3.57672100	1.41700500
F	2.03368700	2.24429100	2.87913100
F	0.21501500	0.67398000	1.63745700
F	2.27329700	-1.53453400	-1.96631700
F	3.22207100	-3.83200400	-0.92368200
F	1.91484600	-5.01792900	1.14586000
F	-0.32780800	-3.88387300	2.16310000
F	-1.27348400	-1.60871100	1.16392200
C	-1.68793400	0.45464400	-0.68166800
C	-2.69674300	-0.48548100	-0.88988000
C	-2.07454900	1.67702100	-0.13940800
C	-4.01912000	-0.25579200	-0.54895200
C	-3.39613800	1.93874000	0.20868300
C	-4.36991800	0.97153100	0.00380600
F	-1.19241500	2.65652200	0.06718200
F	-3.72824000	3.11282500	0.73206000
F	-5.62939200	1.21496700	0.33356900
F	-4.94410800	-1.18681500	-0.74500200
F	-2.37347200	-1.67120500	-1.41429200
P	-0.02688600	0.04726600	-1.38928100

As_cat

C	1.15643600	1.23938900	-0.41954900
C	1.09819100	1.35524800	0.96661800
C	2.13980000	1.95682700	-1.08983900
C	1.97824200	2.16211400	1.67036600
C	3.03863600	2.77297400	-0.40957800
C	2.95287700	2.87201100	0.97267900
C	0.50105000	-1.56944600	-0.42125600
C	1.67610700	-2.16954300	-0.86905700
C	-0.14249600	-2.16689000	0.65913500
C	2.19576100	-3.32137600	-0.29632300
C	0.35126800	-3.32635400	1.24893000
C	1.52051900	-3.90363800	0.76976400
F	3.97170900	3.45055800	-1.06697500
F	2.24737100	1.87730400	-2.41495000
F	3.80084400	3.64463600	1.63369800
F	1.90395200	2.26743700	2.99154300
F	0.16746900	0.68812000	1.65013500
F	2.34940800	-1.61211700	-1.88120900
F	3.31992400	-3.86208400	-0.74968400
F	1.99519700	-5.00402000	1.33347300
F	-0.28413800	-3.87999600	2.27472300
F	-1.25364700	-1.65437800	1.18276000

C	-1.75859000	0.44762100	-0.64784100
C	-2.75115800	-0.51422200	-0.80709600
C	-2.14021500	1.66592200	-0.09940400
C	-4.06342500	-0.30688400	-0.41414800
C	-3.44963100	1.90717700	0.30401100
C	-4.41176400	0.91868500	0.14461200
F	-1.26042100	2.65668500	0.06120400
F	-3.78420800	3.07757200	0.83436800
F	-5.66109900	1.14185800	0.52410500
F	-4.98029200	-1.25462100	-0.56462100
F	-2.42271000	-1.69825400	-1.33538500
As	-0.01011800	0.04042400	-1.44941900

Sb_cat

C	1.31696500	-1.35234400	-0.24146300
C	1.03749700	-1.89839200	1.00661000
C	2.56021500	-1.64910900	-0.79263400
C	1.95138700	-2.70594400	1.67678800
C	3.49694600	-2.44619100	-0.15124500
C	3.18251000	-2.97871300	1.09416400
C	-1.84876100	-0.52019000	-0.44561200
C	-2.78490100	-1.29682600	-1.11673700
C	-2.21605500	-0.02078000	0.80003800
C	-4.04054200	-1.57698100	-0.58952800
C	-3.46173900	-0.27577000	1.35494900
C	-4.37521100	-1.05810100	0.65385300
F	4.67551000	-2.70289000	-0.70597500
F	2.88761700	-1.14197200	-1.98705300
F	4.05875200	-3.74422300	1.72683700
F	1.65703200	-3.21609000	2.86733300
F	-0.12421000	-1.67118800	1.62054000
F	-2.48636100	-1.81102500	-2.31701900
F	-4.91032100	-2.32741900	-1.25625300
F	-5.56511100	-1.31036100	1.17805900
F	-3.79119400	0.21155000	2.54551200
F	-1.36667300	0.71947900	1.51177900
C	0.49774300	1.75350800	-0.52636300
C	-0.46200600	2.72046000	-0.25765200
C	1.82056700	2.08630600	-0.26718300
C	-0.13811100	3.95358600	0.29138600
C	2.18424200	3.30971500	0.28271800
C	1.19470800	4.24419700	0.56374800
F	2.79743800	1.21881900	-0.56021100
F	3.45734800	3.59580900	0.52944700
F	1.52187100	5.41816800	1.08369700
F	-1.07841200	4.85439300	0.55095400
F	-1.74961600	2.47563500	-0.52757700
Sb	0.01282800	-0.13428900	-1.49717000

METHYL VINYL KETONE (R1) + CYCLOPENTADIENE (R2)

Uncat endo

R1

C	-1.92837700	0.16532200	-0.00001100
C	-0.87057900	-0.64769200	0.00003900
H	-1.81834200	1.25206200	0.00001700
H	-0.98221400	-1.73469900	-0.00004000
C	0.54912400	-0.18782300	0.00005900
O	1.44252600	-1.00665100	-0.00002700
H	-2.94566500	-0.22927000	-0.00010300
C	0.83341600	1.29833800	0.00001000
H	0.39396300	1.77443900	-0.88867900
H	0.39216800	1.77509100	0.88744200
H	1.91838000	1.44671300	0.00099500

R2

C	0.73425200	0.99117300	0.00050500
C	1.17767300	-0.28119700	-0.00035400
C	-1.17689500	-0.28422900	0.00022300
C	-0.73684400	0.98926000	-0.00045700
C	0.00155300	-1.21456400	-0.00007000
H	0.00239000	-1.87694600	-0.88329800
H	0.00246200	-1.87710400	0.88302600
H	2.21627900	-0.60737400	0.00039100
H	-2.21464400	-0.61313900	0.00106300
H	-1.35796100	1.88418700	-0.00059800
H	1.35304500	1.88771800	0.00033200

R1_R2

C	-1.10484	-0.97697	-1.1218
H	-0.6311	-1.8716	-1.52271
C	-0.88117	0.29248	-1.51921
H	-0.19699	0.60417	-2.30838
C	-1.70141	1.20236	-0.7077
H	-1.72148	2.28681	-0.81714
C	-2.42009	0.4797	0.17678
H	-3.12902	0.86701	0.90705
C	0.2954	0.33358	2.08426
C	0.98403	-0.53028	1.3345
H	0.34701	1.41092	1.91485
H	0.92537	-1.60966	1.49901
C	1.88251	-0.14072	0.20997
C	-2.12602	-0.9787	-0.02265
H	-3.03457	-1.5318	-0.32102
H	-1.75217	-1.46307	0.89498
O	2.44637	-0.99748	-0.43695
C	2.07697	1.33163	-0.07902
H	1.1081	1.82279	-0.25237
H	2.55364	1.82485	0.78155
H	2.71565	1.4355	-0.96306
H	-0.3531	-0.00799	2.89356

TS

C	-0.82698653	-0.94669606	-0.86626645
H	-0.36916953	-1.83124306	-1.30859045
C	-0.83215653	0.32037894	-1.44269845
H	-0.25499453	0.61454894	-2.31869245
C	-1.56963253	1.18966294	-0.61811845
H	-1.66647353	2.26584594	-0.76066445
C	-2.01779953	0.48119994	0.50158255
H	-2.73272653	0.87864594	1.22271955
C	-0.19941653	0.34109594	1.64261055
C	0.57591447	-0.55167706	0.91283055
H	0.05540747	1.40075694	1.66188755
H	0.55689847	-1.61757706	1.14761755
C	1.75603947	-0.15996206	0.10551355
C	-1.93915353	-0.98144506	0.14247055
H	-2.86997253	-1.24018806	-0.39696245
H	-1.81304353	-1.67959906	0.97651755
O	2.49522847	-1.00037806	-0.36820245
C	2.02481847	1.31922294	-0.08705545
H	1.11290747	1.86071494	-0.37862945
H	2.37523347	1.75134994	0.86314855
H	2.80354947	1.44246694	-0.84797945
H	-0.74038753	-0.01609306	2.51994155

Product

C	-0.70625300	-0.60599400	0.94513100
H	-0.48640900	-1.19879700	1.83915200
C	-1.01573800	-1.38926100	-0.32017100
H	-0.66856500	-2.40055500	-0.51959000
C	-1.64495400	-0.55835000	-1.16090800
H	-1.92248900	-0.75477000	-2.19640100
C	-1.74791300	0.79232200	-0.47410700
H	-2.49083500	1.48934900	-0.87782500
C	-0.29526200	1.36228300	-0.44173400
C	0.40932000	0.41734800	0.58379900
H	0.16476400	1.32643500	-1.43951600
H	0.67035100	1.00338400	1.48154800
C	1.70027100	-0.20799900	0.08870500
C	-1.91866100	0.34352500	0.98941900
H	-2.86464700	-0.18557200	1.16118900
H	-1.80346000	1.16554700	1.71271100
O	1.82972900	-1.40455900	-0.03367300
C	2.83648300	0.74758900	-0.20840600
H	2.50736300	1.54639300	-0.88870200
H	3.15520000	1.23145900	0.72785500
H	3.67870200	0.19848100	-0.64320800
H	-0.28156500	2.40634000	-0.09819800

BF3

R1_cat

C	3.46322600	-0.24147200	-0.00970600
C	2.24933100	-0.79969300	0.00981400
H	3.61424000	0.83860600	-0.02145500
H	2.11727600	-1.88316900	0.01794000
C	0.98638500	-0.04520200	0.01176800
O	-0.04310000	-0.74031000	0.00866500
H	4.35764500	-0.86594400	-0.01762900
C	0.92657700	1.44443300	0.00575700
H	0.45053300	1.75348700	-0.93595100
H	0.24441700	1.75887000	0.80713900
H	1.90684100	1.91273200	0.11754800
B	-1.57474400	-0.10305900	-0.00292200
F	-1.58721200	0.68684800	-1.12838100
F	-2.35503300	-1.20877100	-0.05796800
F	-1.63837200	0.60801200	1.17211600

R1_cat_R2

C	1.66884800	-1.37279000	-0.81394400
H	1.07164800	-1.75405000	-1.64126900
C	1.32061100	-1.39051800	0.49157900
H	0.38386400	-1.78123600	0.88981300
C	2.39967700	-0.77844000	1.27870000
H	2.39298100	-0.67279700	2.36380600
C	3.39178500	-0.38880700	0.44909900
H	4.33249400	0.07850900	0.73687600
C	1.66686900	2.34316600	-0.35106000
C	0.64968800	1.66286700	-0.89151900
H	1.78515000	2.46481900	0.72577900
H	0.54482500	1.54347900	-1.97172600
C	-0.41141700	1.01445400	-0.11060000
C	3.03299600	-0.76755900	-0.95926100
H	3.75026800	-1.50579500	-1.36093200
H	3.05053700	0.08753200	-1.65596300
O	-1.28236300	0.42302700	-0.77538700
H	2.42203800	2.80753200	-0.98748200
C	-0.47990400	1.12621200	1.37676300
H	-1.14918400	1.96985000	1.60838000
H	-0.93671900	0.22420000	1.79613000
H	0.50839300	1.30432700	1.81141300
B	-2.52478300	-0.38476000	-0.09412700
F	-3.13256000	0.54104400	0.72217300
F	-3.25866000	-0.79184900	-1.16053400
F	-1.90971300	-1.40678300	0.60951200

TS

C	1.66900700	-1.37347100	-0.81281900
H	1.07182400	-1.75559500	-1.63976000
C	1.32059900	-1.38928700	0.49267400
H	0.38357200	-1.77893600	0.89132300
C	2.39978500	-0.77648600	1.27906100
H	2.39305500	-0.66949600	2.36404000
C	3.39214000	-0.38835900	0.44904200
H	4.33310200	0.07876800	0.73629800
C	1.66728000	2.34208100	-0.35218200
C	0.64956200	1.66234800	-0.89237000
H	1.78582600	2.46391000	0.72459700
H	0.54458200	1.54269600	-1.97253500
C	-0.41171200	1.01459500	-0.11117600
C	3.03333900	-0.76884500	-0.95883900
H	3.75045700	-1.50771800	-1.35960400
H	3.05099400	0.08537700	-1.65661600
O	-1.28252200	0.42259800	-0.77568700
H	2.42275200	2.80572000	-0.98878100
C	-0.48055100	1.12729600	1.37608400
H	-1.15166200	1.96965000	1.60704800
H	-0.93569200	0.22463100	1.79588100
H	0.50726100	1.30765700	1.81087100
B	-2.52478400	-0.38490400	-0.09404000
F	-3.13268000	0.54112300	0.72193400
F	-3.25877500	-0.79257400	-1.16016000
F	-1.90950800	-1.40656700	0.61001200

Product

C	1.69842500	-0.92722300	0.70789000
H	1.18087500	-1.70573300	1.27739300
C	2.01863800	0.36357200	1.43796000
H	1.49321700	0.72994300	2.31994300
C	2.96095700	1.00061900	0.72877600
H	3.36241600	1.99671900	0.91568600
C	3.27062300	0.14961100	-0.48855000
H	4.21776300	0.35663100	-0.99727200
C	2.01718700	0.24441100	-1.41763600
C	0.94343900	-0.55379500	-0.63648800
H	1.73249800	1.28583600	-1.61657700
H	0.70009400	-1.50042600	-1.13578600
C	-0.37275900	0.10019800	-0.35193500
C	3.07679900	-1.25733000	0.10618200
H	3.81620800	-1.49474200	0.88113700
H	3.04244300	-2.05444400	-0.65062500
O	-1.34051300	-0.66899100	-0.24378200
H	2.21126400	-0.23614900	-2.38572100
C	-0.51779300	1.57627000	-0.19541800
H	-0.94596900	1.95564100	-1.13753800
H	-1.24886100	1.79156800	0.59277400
H	0.44647600	2.05468100	-0.00344200
B	-2.87385000	-0.15556500	0.08738200
F	-3.11698700	0.78740700	-0.88430900
F	-3.60193000	-1.29538100	-0.01397900
F	-2.77976800	0.37155500	1.35480800

H_cat

R1_cat

C	-4.96939600	-0.57002500	-0.00042900
C	-3.64661900	-0.75517600	-0.00036100
H	-5.41572100	0.42664600	-0.00013800
H	-3.21385900	-1.75810600	-0.00060200
C	-2.66002600	0.34961400	0.00007700
O	-1.46366800	0.08566400	0.00015300
H	-5.65589100	-1.41782300	-0.00069800
C	-3.14253500	1.77593700	0.00042200
H	-3.76292300	1.96791200	-0.88661700
H	-3.76332500	1.96735600	0.88730100
H	-2.28286700	2.45379200	0.00083100
C	3.18327000	-1.28134200	-0.00010200
C	3.71361400	-0.02629100	-0.00027200
C	1.51440400	0.16117700	0.00017700
N	1.81316900	-1.13851000	0.00019100
N	2.65435200	0.85616000	-0.00011300
H	3.66120500	-2.25503800	-0.00012400
H	4.74447800	0.31103200	-0.00051100
C	0.83024400	-2.22477100	0.00034500
H	0.97238200	-2.83580000	0.89908900
H	-0.16689000	-1.77017100	0.00010200
H	0.97267200	-2.83627500	-0.89802400
C	2.76106900	2.31426000	-0.00020100
H	3.29703200	2.64016500	-0.89894400
H	1.75155700	2.73723400	-0.00015200
H	3.29717100	2.64026900	0.89842100
H	0.49753000	0.55963100	0.00035400

R1_cat_R2

C	-2.64965700	-0.91886700	-1.47235000
H	-1.76951000	-1.51177300	-1.71699500
C	-2.80545700	0.40743400	-1.67199000
H	-2.06814300	1.08286000	-2.10681800
C	-4.14635700	0.80765500	-1.22635600
H	-4.54727500	1.81945800	-1.28703300
C	-4.79788800	-0.27931100	-0.75982400
H	-5.81785100	-0.30989600	-0.37971200
C	-3.27523200	0.05557300	2.07893900
C	-2.04675500	-0.28587800	1.67653600
H	-3.64822300	1.07804800	2.00028600
H	-1.68458300	-1.31364500	1.75769700
C	-1.07845500	0.67210500	1.10183700
C	-3.91914700	-1.48610800	-0.91038000
H	-4.36959300	-2.20609000	-1.61742600
H	-3.77053600	-2.03540800	0.03394600
O	0.03968300	0.28536000	0.77723100
H	-3.95449200	-0.68412300	2.50623900
C	-1.47069800	2.11917200	0.96261100
H	-2.42810600	2.21158700	0.43158700
H	-1.59814500	2.56211900	1.96194700
H	-0.68843600	2.66227600	0.42153200
C	4.47017700	-1.37707900	-0.12120600
C	5.00813600	-0.18431500	-0.50099000
C	2.88327700	0.15134500	-0.00465000
N	3.14717600	-1.14242900	0.18281900
N	4.00033300	0.75312200	-0.42039000
H	4.91352200	-2.36380100	-0.04240900
H	6.01233000	0.07504800	-0.81843500
C	2.17378300	-2.13790800	0.63862100
H	2.53151600	-2.58543000	1.57301100
H	1.22149900	-1.61997800	0.80162200
H	2.06480100	-2.91036100	-0.13143300
C	4.13219000	2.17433300	-0.73593500
H	4.43577300	2.28861100	-1.78285000
H	3.16234300	2.65688800	-0.57928300
H	4.87984400	2.62689900	-0.07453700
H	1.90573500	0.61007100	0.16502000

TS

C	2.93792600	-0.67451900	1.30181900
H	2.12743800	-1.17316800	1.83290900
C	3.32444800	0.64535500	1.48647800
H	2.80356300	1.37824800	2.10144300
C	4.42856400	0.91309900	0.65458000
H	4.91196900	1.88284700	0.53617200
C	4.72141500	-0.23079600	-0.10104600
H	5.63894900	-0.34122400	-0.67967700
C	3.27179200	-0.11829700	-1.58292900
C	2.07015900	-0.45954200	-0.96221800
H	3.43974700	0.90458100	-1.92009200
H	1.74650500	-1.50103100	-0.92859900
C	1.03210800	0.50225500	-0.62291500
C	4.05633700	-1.38731900	0.60476800
H	4.75842100	-1.73845200	1.38439000
H	3.77767300	-2.24414900	-0.01703400
O	-0.08999400	0.13180700	-0.25927400
H	3.77451200	-0.87522500	-2.18674800
C	1.32750400	1.97587400	-0.76617400
H	2.33922000	2.22683500	-0.41992400
H	1.26693800	2.24898700	-1.83138900
H	0.58481400	2.55703500	-0.20746500
C	-4.61754400	-1.34144500	0.05714200
C	-5.16847100	-0.10452700	0.20729900
C	-2.97860600	0.13693200	0.04754200
N	-3.25468500	-1.16492100	-0.04011300
N	-4.12785000	0.80033300	0.19831000
H	-5.07647500	-2.32308100	0.01175000
H	-6.20162200	0.20622000	0.31853700
C	-2.25314200	-2.22009900	-0.21316100
H	-2.45425700	-2.75321900	-1.14957400
H	-1.26905800	-1.73714000	-0.24903500
H	-2.31402100	-2.91236800	0.63449800
C	-4.25841200	2.24968900	0.33082400
H	-4.72369000	2.48805500	1.29415200
H	-3.25913200	2.69402000	0.28507700
H	-4.87193500	2.63771500	-0.49040300
H	-1.96633200	0.55221500	-0.00422100

Product

C	2.56870000	-0.89730200	0.63670900
H	1.75915700	-1.52376000	1.02720200
C	3.11577600	0.17048600	1.56811200
H	2.58060200	0.60336800	2.41349800
C	4.31051300	0.54263300	1.08740700
H	4.94946400	1.34346900	1.45966700
C	4.56963100	-0.26642200	-0.17014700
H	5.60997000	-0.32117800	-0.50551900
C	3.58104200	0.28194600	-1.24808100
C	2.19782600	-0.18592800	-0.72624900
H	3.66821300	1.37033400	-1.35561300
H	1.76651000	-0.95302600	-1.38599100
C	1.10138700	0.83283100	-0.51597600
C	3.87444900	-1.58810600	0.20522600
H	4.36568200	-2.10312100	1.03993000
H	3.74671300	-2.27202000	-0.64668800
O	-0.05041400	0.44181700	-0.37275800

H	3.79152700	-0.16504500	-2.22876900
C	1.42776300	2.29785200	-0.46109600
H	2.28340800	2.47178900	0.20670800
H	1.72944800	2.63585300	-1.46478700
H	0.55274000	2.86789200	-0.13067600
C	-4.45006200	-1.48246700	0.04113300
C	-5.11684800	-0.30791800	0.22048200
C	-2.97129000	0.15370100	-0.01763600
N	-3.11649000	-1.16927600	-0.10473400
N	-4.17447400	0.69761600	0.18002500
H	-4.80918100	-2.50529900	0.00520700
H	-6.17129200	-0.10371300	0.37180800
C	-2.02311500	-2.12031100	-0.32050800
H	-2.20749600	-2.67226300	-1.24935300
H	-1.09274100	-1.54509800	-0.39415600
H	-1.98012900	-2.81376400	0.52729900
C	-4.44648400	2.12627100	0.32883300
H	-4.89703600	2.31113800	1.31070600
H	-3.50023000	2.67068600	0.24971300
H	-5.12673600	2.45244300	-0.46618500
H	-2.01425500	0.67479000	-0.09822500

H2_cat

R1_cat

C	5.34173500	-0.51183600	0.11233000
C	4.53852600	-1.64001900	0.03095900
C	4.71761400	0.73664600	0.10047000
H	6.42512500	-0.59871600	0.17958300
C	3.14686100	-1.56462900	-0.06619700
C	5.16235200	-3.01031500	0.06857900
C	3.34102000	0.84088900	0.02090500
C	5.56399700	1.97708000	0.15640800
C	2.53502800	-0.30825600	-0.06520700
H	2.55212000	-2.47004600	-0.14371800
F	6.48416000	-2.96226700	-0.14714200
F	4.97500700	-3.60000400	1.25666200
F	4.62948200	-3.81856200	-0.85672800
H	2.86911100	1.82399300	0.02806900
F	6.41869600	1.94677300	1.18619100
F	6.29971900	2.12126200	-0.95435800
F	4.81914600	3.08932100	0.28399000
N	1.16575700	-0.05100900	-0.18621800
C	0.05763800	-0.84307800	-0.01833700
H	0.97222300	0.93983200	-0.32257900
S	0.06803400	-2.46393500	0.35866700
N	-1.07152100	-0.07843600	-0.18586500
H	-0.91249200	0.92395900	-0.28308000
C	-2.42824800	-0.39615800	-0.05944600
C	-3.27020600	0.67979000	0.24773100
C	-2.99435200	-1.65678200	-0.29083600
C	-4.64656300	0.51056700	0.30889000
H	-2.83407000	1.66220700	0.43847400
C	-4.37622300	-1.80393800	-0.20196000
H	-2.36661600	-2.50506200	-0.54728900
C	-5.22227200	-0.73534600	0.08505700
C	-5.49538300	1.70322500	0.64678000
C	-4.97162000	-3.17136400	-0.41295300
H	-6.30204600	-0.86918800	0.12011900
F	-6.76964000	1.53472900	0.28132300
F	-5.48720500	1.96529500	1.96088300
F	-5.04068200	2.81480300	0.03466200
F	-5.04240100	-3.85716100	0.73580000
F	-4.24661000	-3.90456100	-1.26595900
F	-6.21864100	-3.09438100	-0.90019700
C	-1.72112900	5.78483000	-0.71985600
C	-1.48886700	4.47392300	-0.61648700
H	-0.91191300	6.51793000	-0.73107200
H	-2.31216200	3.75547400	-0.60785300
C	-0.13191200	3.88654700	-0.50537000
O	-0.01177500	2.67333200	-0.39403900
H	-2.73948500	6.16814800	-0.79603200
C	1.07548700	4.78565200	-0.52932200
H	1.09804200	5.36421400	-1.46373800
H	1.02914100	5.50065900	0.30456800
H	1.98885500	4.18723100	-0.44620500

R1_cat_R2

C	5.73483500	-0.12736500	0.13739300
C	5.12742000	-1.37138900	0.02274400
C	4.92839100	1.00567300	0.04647900
H	6.80945300	-0.04045600	0.28986900
C	3.75450700	-1.51331400	-0.17752200
C	5.95694900	-2.62239000	0.14225300
C	3.55850700	0.89295000	-0.13182300
C	5.56215200	2.36701900	0.10918800
C	2.95388000	-0.36864900	-0.24397000
H	3.31599300	-2.50158000	-0.28002200
F	7.25861600	-2.37681000	-0.06258200
F	5.84509300	-3.17061500	1.35914300
F	5.57525500	-3.55306400	-0.74223000
H	2.94257900	1.79201700	-0.18132900
F	6.53374900	2.41911200	1.02830100
F	6.11722400	2.70677600	-1.06351900
F	4.66724200	3.32261600	0.40861000
N	1.57321700	-0.36220500	-0.47695700
C	0.61127800	-1.30429000	-0.19999300
H	1.21723200	0.55730700	-0.73672800
S	0.87787500	-2.77552900	0.53118600
N	-0.61436900	-0.82931300	-0.59453500
H	-0.62317900	0.13532300	-0.92900200
C	-1.90261000	-1.32055200	-0.35891000
C	-2.90831000	-0.34858600	-0.29259400
C	-2.25550200	-2.67315100	-0.26219700
C	-4.23901100	-0.71445800	-0.14030100
H	-2.63305300	0.70625200	-0.35697100
C	-3.59584700	-3.01119200	-0.09243000
H	-1.49592800	-3.44657700	-0.33010300
C	-4.60497200	-2.05220500	-0.03802500
C	-5.26829800	0.37450500	-0.05934600
C	-3.96411600	-4.46493900	0.04990400
H	-5.64868900	-2.34324300	0.06796800
F	-6.50152700	-0.06644900	-0.31391900
F	-5.29467200	0.94753200	1.15822000
F	-5.00247800	1.36883600	-0.93320200
F	-3.95079300	-4.84986400	1.33305200
F	-3.11602900	-5.26125600	-0.61040200
F	-5.19798500	-4.70609100	-0.41760400
C	-3.45459100	5.13140200	1.21063600
H	-4.03692100	6.02425600	0.98711500
C	-2.15261800	5.09120400	1.56820200
H	-1.49274100	5.95103700	1.68814300
C	-1.76091800	3.69483800	1.79993400
H	-0.76864000	3.36883900	2.11253500
C	-2.83100400	2.89673400	1.59130500
H	-2.87420500	1.81714000	1.72728700
C	-2.66106300	4.45047600	-1.85032100
C	-2.07513300	3.26144000	-1.67774600
H	-2.12686800	5.38879400	-1.69270700

H	-2.62733200	2.33253100	-1.83991800
C	-0.66118600	3.10256800	-1.26961700
C	-4.01302000	3.73983300	1.21814900
H	-4.81908200	3.63523200	1.96626700
H	-4.45826800	3.44138400	0.25461000
O	-0.19236700	1.97983800	-1.11732500
H	-3.70641600	4.52015300	-2.15625300
C	0.19413500	4.32512000	-1.07528800
H	0.29526000	4.86132700	-2.03047500
H	-0.27956700	5.00807100	-0.35613400
H	1.18499000	4.02839700	-0.71551200

TS

C	5.70996700	-0.14340500	0.07146400
C	5.11678400	-1.39723600	0.12625000
C	4.88507800	0.95645800	-0.16377800
H	6.78310900	-0.02269200	0.20778600
C	3.74307400	-1.58617600	-0.03668800
C	5.95809100	-2.61397900	0.40767700
C	3.51921400	0.80045900	-0.32344500
C	5.48601400	2.32859400	-0.28368500
C	2.92526200	-0.47250300	-0.25602400
H	3.31799400	-2.58420200	0.01197500
F	7.26632400	-2.36074200	0.26360000
F	5.77553900	-3.05596100	1.65975700
F	5.64872600	-3.62702400	-0.41165800
H	2.89101600	1.67376600	-0.50749500
F	6.53607800	2.48395600	0.53153000
F	5.91747400	2.57485900	-1.52885900
F	4.59459200	3.29004100	0.01479300
N	1.54063000	-0.47597700	-0.43957500
C	0.58614700	-1.45332200	-0.30753300
H	1.16997000	0.46214800	-0.60188100
S	0.85121300	-3.01882400	0.19445800
N	-0.63627900	-0.92657600	-0.64882300
H	-0.62049100	0.06207600	-0.90655100
C	-1.93866500	-1.38444200	-0.44591900
C	-2.91843400	-0.37920800	-0.48383900
C	-2.34264700	-2.71090700	-0.25186500
C	-4.25905900	-0.68446000	-0.31685600
H	-2.60560100	0.65443500	-0.64495500
C	-3.70002500	-2.98778700	-0.08306200
H	-1.61083100	-3.51364900	-0.24089500
C	-4.67596700	-1.99816300	-0.11291700
C	-5.24399300	0.44678600	-0.31166600
C	-4.09385800	-4.42280300	0.15071300
H	-5.72907400	-2.24360500	0.00859600
F	-6.49165700	0.04513300	-0.55688500
F	-5.25905500	1.08452000	0.87681200
F	-4.92749500	1.38517900	-1.22803800
F	-3.73871600	-4.83270600	1.37556300
F	-3.49939000	-5.24454500	-0.72332000
F	-5.41762900	-4.60134700	0.03970700
C	-3.28556600	5.19072100	0.77405500
H	-3.85019600	6.09107000	0.52949400
C	-2.24877900	5.17498700	1.71940400
H	-1.71982200	6.05509800	2.08547000
C	-1.90729900	3.84008400	2.00397200
H	-1.06270800	3.51699200	2.61145400
C	-2.75346700	3.00182100	1.28996800
H	-2.75845100	1.91324600	1.34923200
C	-2.25176300	4.83690800	-0.97809500
C	-1.86511500	3.49655800	-0.93235200
H	-1.49920800	5.62179400	-0.90182600

H	-2.51723000	2.73083900	-1.35666900
C	-0.50067900	3.06519200	-0.65877500
C	-3.91120300	3.81840400	0.80484300
H	-4.66778500	3.81523700	1.61220700
H	-4.39502800	3.47262700	-0.11343800
O	-0.15707000	1.88719900	-0.80754800
H	-3.11250500	5.10089700	-1.59408700
C	0.51975700	4.09112400	-0.23090700
H	0.83778700	4.66556300	-1.11494500
H	0.09957700	4.80338000	0.49259300
H	1.39583700	3.59194200	0.19911100

Product

C	5.73756400	-0.04854600	0.07956900
C	5.18298200	-1.31326000	-0.06334600
C	4.87727400	1.04896200	0.05202200
H	6.81113300	0.08198700	0.20634500
C	3.81180300	-1.51416800	-0.23155000
C	6.06959900	-2.52944400	-0.01020800
C	3.51183300	0.87760700	-0.09638800
C	5.44813200	2.43543300	0.15392400
C	2.95925200	-0.40603900	-0.23877800
H	3.41703800	-2.51822500	-0.35464900
F	7.35696800	-2.21569000	-0.21117400
F	5.99204300	-3.13998600	1.17966400
F	5.72231400	-3.43274300	-0.93603300
H	2.85334500	1.74693900	-0.10107100
F	6.39062700	2.51410600	1.10082500
F	6.01925700	2.81953800	-0.99646800
F	4.50328800	3.34600900	0.44516100
N	1.57364200	-0.43504800	-0.42764900
C	0.64636900	-1.43502800	-0.25216100
H	1.18206000	0.49516000	-0.57381900
S	0.97747400	-2.98276800	0.26093600
N	-0.59869600	-0.94432800	-0.55579200
H	-0.62050500	0.04417800	-0.80360300
C	-1.88611600	-1.44955700	-0.36069300
C	-2.90316300	-0.48856700	-0.46918400
C	-2.24067900	-2.77984200	-0.10596100
C	-4.23353400	-0.83541700	-0.30464300
H	-2.63283200	0.54650300	-0.68594200
C	-3.58949200	-3.10069000	0.05264900
H	-1.47938600	-3.55171300	-0.03977300
C	-4.60250300	-2.15254200	-0.04204200
C	-5.25856000	0.25924400	-0.37246400
C	-3.93762100	-4.53321100	0.36279900
H	-5.64762300	-2.43295600	0.07415800
F	-6.48455100	-0.20237600	-0.62603900
F	-5.32248600	0.94820300	0.78181200
F	-4.95341700	1.16084200	-1.32753200
F	-3.71074900	-4.82238000	1.65131700
F	-3.20730500	-5.38738100	-0.36414800
F	-5.22990600	-4.79416100	0.12060300
C	-3.48497700	5.18850800	0.28237900
H	-4.15531200	6.03774400	0.11399700
C	-2.57251600	5.28214000	1.49141200
H	-2.25372600	6.20987500	1.96704400
C	-2.14761800	4.04211900	1.77232100
H	-1.41408000	3.74896300	2.52373200
C	-2.77947300	3.10755600	0.75630100
H	-2.80089100	2.04356100	1.01572700
C	-2.56196200	4.85834300	-0.93372000
C	-2.09902300	3.40642100	-0.64210500
H	-1.73561400	5.57515100	-1.01910200

H	-2.53414000	2.70640000	-1.37111200
C	-0.61670700	3.10545600	-0.60888200
C	-4.12858200	3.81205600	0.52610200
H	-4.76078900	3.78675200	1.42229900
H	-4.67840900	3.42014800	-0.34035200
O	-0.24093300	1.94413100	-0.70403800
H	-3.13175900	4.89493600	-1.87175400
C	0.38135800	4.21751900	-0.44815300
H	0.41776700	4.79463900	-1.38561500
H	0.06313100	4.90818700	0.34467300
H	1.37726100	3.81250600	-0.23686500

Cl_cat

R1_cat

C	5.90713300	-0.16726200	-0.50987800
C	4.61706700	-0.26458800	-0.83955000
H	6.22838000	0.14281900	0.48686000
H	4.30151300	-0.57502000	-1.83800100
C	3.49685500	0.03054000	0.08912300
O	2.34924000	-0.08861200	-0.30704400
H	6.69205500	-0.39638800	-1.23199900
C	3.80286200	0.47062900	1.49979800
H	4.39191700	-0.29726600	2.02141400
H	4.39742800	1.39540100	1.49196800
H	2.86352300	0.64113200	2.03643300
C	-4.08310800	0.66390800	-0.05594300
C	-4.07734900	-0.69292700	0.04546000
C	-1.98437700	-0.00742200	-0.02853700
N	-2.76609200	1.07614500	-0.10147900
N	-2.75694900	-1.09627200	0.06071100
H	-4.90514700	1.36970900	-0.10045500
H	-4.89340100	-1.40437800	0.10696700
C	-2.28145500	2.45307300	-0.21390400
H	-1.68577300	2.70589500	0.67106500
H	-1.67151900	2.55336400	-1.11931900
H	-3.15270000	3.11165200	-0.27697600
C	-2.26050400	-2.47041800	0.15673400
H	-1.63336400	-2.69397000	-0.71419400
H	-1.67893800	-2.58722800	1.07878800
H	-3.12583800	-3.13951600	0.17433300
Cl	-0.29903000	-0.00409500	-0.05323400

R1_cat_R2

C	3.43927600	-1.41187100	-1.01824200
H	2.63467300	-1.54535200	-1.73970500
C	3.36982600	-1.62350400	0.31295700
H	2.49354700	-1.96637700	0.86390200
C	4.67372700	-1.32166400	0.91875400
H	4.90624200	-1.41805000	1.97928700
C	5.52817700	-0.92948400	-0.04994100
H	6.57404200	-0.65597700	0.08016100
C	4.19538300	2.10342200	0.05677400
C	3.01568100	1.78505000	-0.48385400
H	4.38805800	2.00386300	1.12656400
H	2.82763200	1.88060900	-1.55597900
C	1.85877700	1.27974100	0.29371400
C	4.83066700	-0.98286900	-1.37725900
H	5.31159900	-1.72153800	-2.04339400
H	4.85611800	-0.02048500	-1.91477100
O	0.81637100	1.01454800	-0.28432400
H	5.01654600	2.47578700	-0.55837600
C	1.99185800	1.13583900	1.79004100
H	2.86843100	0.52282800	2.04273100
H	2.13414000	2.12620800	2.24795100
H	1.08326100	0.67470100	2.19346400
C	-5.55531800	0.06518700	-0.15524100
C	-5.24029200	-1.24068500	0.06074700
C	-3.36001300	-0.09503300	-0.04161100
N	-4.36808100	0.76732100	-0.21716300
N	-3.86357800	-1.32317800	0.12863200
H	-6.51599300	0.55463800	-0.27106800
H	-5.87182100	-2.11529900	0.17055000
C	-4.21036900	2.20638300	-0.43641000
H	-3.74317900	2.66325600	0.44399200
H	-3.58677400	2.37465300	-1.32214800
H	-5.20525500	2.63299600	-0.59455200
C	-3.06733400	-2.53205500	0.34636400
H	-2.37688400	-2.67115500	-0.49369500
H	-2.50569700	-2.43834900	1.28331200
H	-3.75480600	-3.38084800	0.40748900
Cl	-1.72138300	0.30398600	-0.04424500

TS

C	-3.80220700	0.54449200	-1.36358900
H	-3.05405800	0.55801500	-2.15585900
C	-3.97922800	1.54053000	-0.41057500
H	-3.31950800	2.39402600	-0.25803000
C	-5.06895800	1.19365500	0.40953200
H	-5.39935300	1.74355300	1.29063100
C	-5.57053800	-0.04824600	-0.00031600
H	-6.51331700	-0.46131900	0.35933600
C	-4.14395700	-1.35277400	0.83785700
C	-3.00350400	-1.17857300	0.05819600
H	-4.16579400	-0.99100900	1.86594000
H	-2.83060700	-1.80497100	-0.81812900
C	-1.80783100	-0.45976700	0.50575200
C	-5.05828000	-0.27386700	-1.40131400
H	-5.76065400	0.23121500	-2.09096100
H	-4.95093300	-1.31587200	-1.71933100
O	-0.77010700	-0.49731900	-0.14924100
H	-4.77788500	-2.22080300	0.65185000
C	-1.86934900	0.29829000	1.81323900
H	-2.79592200	0.88343000	1.89804600
H	-1.85779000	-0.41921500	2.64832400
H	-0.99787200	0.95852300	1.89548900
C	5.62598600	-0.51190200	-0.14349800
C	5.53630400	0.84450100	-0.09018200
C	3.48895000	0.02566500	-0.06515300
N	4.33732200	-1.00757400	-0.12757600
N	4.19346600	1.16350400	-0.04228800
H	6.48986600	-1.16544300	-0.19225300
H	6.30633700	1.60787200	-0.08338800
C	3.93781600	-2.41541400	-0.17033700
H	3.41265600	-2.67668500	0.75596800
H	3.28157300	-2.58270800	-1.03236100
H	4.84474700	-3.01921700	-0.26880400
C	3.61283100	2.50523300	0.02227200
H	2.93784600	2.65253100	-0.82886300
H	3.06217500	2.62275400	0.96309100
H	4.43201200	3.22893500	-0.02290900
Cl	1.80668200	-0.09455900	-0.02859500

Product

C	-3.59256900	0.13379500	-1.20244600
H	-2.89403800	0.16478200	-2.04490200
C	-3.92697500	1.45889000	-0.53946500
H	-3.30691000	2.35493900	-0.57521900
C	-5.04674400	1.27618800	0.17452200

H	-5.52662800	1.99174100	0.84241300
C	-5.46697900	-0.17194900	0.00083000
H	-6.50710600	-0.40750100	0.24784600
C	-4.41607400	-1.02174400	0.78465300
C	-3.12771500	-0.85281000	-0.06110900
H	-4.31434900	-0.67494900	1.82089300
H	-2.84511300	-1.79674500	-0.54851100
C	-1.86107900	-0.34720400	0.59756800
C	-5.00706200	-0.41940700	-1.44819900
H	-5.56702200	0.17841900	-2.17811800
H	-5.01584200	-1.48147600	-1.73385900
O	-0.80513400	-0.45510600	-0.00269500
H	-4.71977800	-2.07675300	0.81704600
C	-1.92306200	0.29588900	1.95840400
H	-2.71306400	1.06021400	1.98309800
H	-2.18697800	-0.46427400	2.70985400
H	-0.95006300	0.73454700	2.20762400
C	5.63510600	-0.52311300	-0.19152400
C	5.54233200	0.83421700	-0.18181800
C	3.50128500	0.01177000	-0.05559300
N	4.34984400	-1.02136400	-0.11243600
N	4.20110400	1.15165800	-0.09691800
H	6.49916400	-1.17582600	-0.24842300
H	6.30937300	1.59928000	-0.22861000
C	3.95498900	-2.43122100	-0.09471600
H	3.44592900	-2.65802100	0.84940300
H	3.28683100	-2.63432700	-0.93980200
H	4.86274600	-3.03527000	-0.18348600
C	3.61869100	2.49405600	-0.05866500
H	2.92089500	2.61367100	-0.89564500
H	3.09374100	2.63989500	0.89276600
H	4.43472000	3.21712500	-0.14840000
Cl	1.82194500	-0.10851800	0.04390700

Br_cat

R1_cat

C	4.01683400	0.71591200	-0.26108100
C	4.02917400	-0.64299800	-0.33082400
C	1.94326700	0.00248500	-0.00240800
N	2.70799500	1.10141900	-0.05569300
N	2.72774300	-1.07115700	-0.16718700
H	4.82149300	1.43872100	-0.33864300
H	4.84670600	-1.33937600	-0.48120000
C	2.26549100	-2.45981900	-0.16512000
H	1.54852300	-2.60930600	-0.98099200
H	3.13696200	-3.10469500	-0.31244400
H	1.79147900	-2.68674300	0.79714300
C	2.22069300	2.47394700	0.08774800
H	1.50291500	2.69414000	-0.71122700
H	1.74068700	2.59199400	1.06641800
H	3.08062200	3.14602400	0.01118700
C	-4.90573000	0.19778600	-1.76145900
C	-3.73555700	0.12722400	-1.12270800
H	-5.85762500	0.13140400	-1.23041000
H	-2.78890200	0.19480200	-1.66508700
C	-3.60056700	-0.04490900	0.34611700
O	-2.48957000	-0.10013800	0.85107800
H	-4.95173400	0.32494900	-2.84395000
C	-4.83784000	-0.14954800	1.19864900
H	-5.45384900	0.75465500	1.09024500
H	-5.44768900	-1.00817100	0.88295500
H	-4.53948900	-0.27139100	2.24470100
Br	0.11604900	-0.02823500	0.26541000

R1_cat_R2

C	-3.57609700	1.50586500	-0.88722600
H	-2.84563800	1.63920000	-1.68375100
C	-3.34893400	1.60918500	0.43937500
H	-2.39505600	1.84828900	0.91088500
C	-4.59952000	1.35033700	1.16552900
H	-4.70966000	1.38122900	2.24954500
C	-5.57972800	1.09184600	0.27398900
H	-6.62332000	0.88037000	0.50149600
C	-4.48065600	-2.03630800	-0.00103500
C	-3.33780000	-1.74008400	-0.62705200
H	-4.57000900	-2.01066400	1.08654900
H	-3.25246700	-1.76141000	-1.71602000
C	-2.08974900	-1.35774100	0.07295300
C	-5.02638600	1.20105600	-1.11626700
H	-5.51819500	2.02099100	-1.66996800
H	-5.18262600	0.28850200	-1.71496700
O	-1.09260100	-1.09623600	-0.58519100
H	-5.37459800	-2.31536500	-0.56163600
C	-2.07350300	-1.33244400	1.58069200
H	-2.89100300	-0.70590200	1.96405400
H	-2.22261300	-2.35094100	1.96947600
H	-1.11031900	-0.94601300	1.93354600
C	5.38931500	0.08950700	0.11987700
C	5.00831800	1.39478200	0.17071300
C	3.18646900	0.16127100	-0.02416700
N	4.24075600	-0.66547400	-0.00197900
N	3.63174400	1.42090000	0.07931000
H	6.37276800	-0.36568500	0.15874000
H	5.59385500	2.30283800	0.26287700
C	4.16802400	-2.12402200	-0.09769200
H	3.59048300	-2.51912300	0.74636600
H	3.68994900	-2.40576500	-1.04331900
H	5.18944500	-2.51481100	-0.06555600
C	2.78507100	2.61426100	0.08670600
H	3.43667700	3.48907000	0.17136800
H	2.21324400	2.66381400	-0.84747900
H	2.10151500	2.57426200	0.94300600
Br	1.41691300	-0.35074500	-0.18266800

TS

C	-4.04623800	1.36792000	-0.49051800
H	-3.35041600	1.94097700	-1.10291800
C	-4.08283100	1.36862500	0.89837100
H	-3.35201000	1.84578600	1.55043600
C	-5.14075600	0.54324100	1.32345300
H	-5.37183000	0.29168300	2.35850800
C	-5.76135500	-0.01818500	0.19981400
H	-6.70960000	-0.55433800	0.24647500
C	-4.39871200	-1.53862800	-0.29288200
C	-3.29686600	-0.87342700	-0.82539500
H	-4.32750600	-2.02735600	0.67885700
H	-3.22992800	-0.67929600	-1.89665400
C	-2.02752400	-0.70328400	-0.12163200
C	-5.35865900	0.83280000	-0.97910500
H	-6.06493400	1.68328700	-1.02339700
H	-5.34999700	0.34035000	-1.95670100
O	-1.03885400	-0.26778400	-0.71165400
H	-5.10854100	-2.00298000	-0.97883500
C	-1.94297900	-1.11077800	1.33161400
H	-2.82660100	-0.78112700	1.89568800
H	-1.90486600	-2.20943000	1.39545200
H	-1.03467700	-0.69349900	1.78309000
C	5.48456600	-0.41832900	0.22272700

C	5.36945300	0.93727700	0.22717800
C	3.34494800	0.08344400	-0.01192000
N	4.21337300	-0.93398700	0.07247700
N	4.02941500	1.23213400	0.07961400
H	6.35575200	-1.05769500	0.31217300
H	6.12034800	1.71390200	0.32119400
C	3.85488900	-2.35091300	0.00846900
H	3.18554300	-2.59852000	0.84080600
H	3.35800900	-2.56035500	-0.94616300
H	4.77653200	-2.93559100	0.08502700
C	3.43696800	2.56864500	0.02387700
H	4.24606900	3.29989700	0.11174000
H	2.91725700	2.69944200	-0.93265100
H	2.73038400	2.69208800	0.85306200
Br	1.51265400	-0.07092700	-0.23002600

Product

C	-3.92124300	0.96934700	-0.68054800
H	-3.29546300	1.62747100	-1.29197900
C	-4.13446500	1.37417900	0.76779600
H	-3.47836200	2.02897700	1.34183600
C	-5.19760300	0.69386600	1.21966900
H	-5.58555500	0.67808100	2.23830800
C	-5.70036900	-0.17518000	0.08143400
H	-6.72616300	-0.54635300	0.17214500
C	-4.63052200	-1.29739500	-0.10974900
C	-3.41147700	-0.52596300	-0.67723400
H	-4.42529100	-1.81932300	0.83360000
H	-3.21668700	-0.80038100	-1.72353400
C	-2.07320800	-0.63112900	0.01980800
C	-5.37662400	0.73194400	-1.12039200
H	-5.96593600	1.65729800	-1.12321900
H	-5.46079700	0.21985800	-2.09003700
O	-1.07573900	-0.24782000	-0.57167600
H	-4.98138500	-2.04606300	-0.83263200
C	-1.99036700	-1.17708900	1.42028800
H	-2.74364000	-0.69638200	2.06067200
H	-2.22149000	-2.25347600	1.40402800
H	-0.98351400	-1.02474200	1.82650900
C	5.52886200	-0.41576600	0.11400800
C	5.40347300	0.93721600	0.18568700
C	3.38002900	0.07660000	-0.02197300
N	4.25745100	-0.93616600	-0.01550000
N	4.05708000	1.22655800	0.09905400
H	6.40761200	-1.05048200	0.14317400
H	6.15129600	1.71561400	0.28961800
C	3.90837700	-2.35233100	-0.13256900
H	3.26821800	-2.64358300	0.70853500
H	3.38385000	-2.52323900	-1.08007900
H	4.83676000	-2.93086300	-0.11137700
C	3.45316000	2.55929500	0.12743800
H	4.25981700	3.29231000	0.22242800
H	2.90031600	2.73089200	-0.80371900
H	2.77523600	2.63694300	0.98560900
Br	1.54506400	-0.08309400	-0.18123900

I_cat

R1_cat

C	-4.85447000	0.37592800	1.87005800
C	-3.72262800	0.22956400	1.17658400
H	-5.83587600	0.28455500	1.40006900
H	-2.74750500	0.32363400	1.66159500
C	-3.68010500	-0.06676400	-0.27492800
O	-2.60171900	-0.18641500	-0.84438200
H	-4.83663700	0.59360300	2.93884000
C	-4.96210400	-0.22011800	-1.04671700
H	-5.56405500	-1.03810200	-0.62572100
H	-5.55794100	0.70149000	-0.98102700
H	-4.72431000	-0.43463400	-2.09321300
C	4.12254100	0.76843300	0.30282600
C	4.15176500	-0.58540200	0.43390200
C	2.05277200	0.01513100	0.04399500
N	2.81215800	1.12268700	0.06109200
N	2.85876000	-1.03508200	0.27006100
H	4.91620700	1.50515400	0.35978400
H	4.97590800	-1.26299400	0.62782400
C	2.32439000	2.48517500	-0.15052500
H	1.86734400	2.56272700	-1.14427400
H	1.58760900	2.73753700	0.62134400
H	3.17928700	3.16487700	-0.08224900
C	2.43048100	-2.43255800	0.32554500
H	1.69678100	-2.56180300	1.13006600
H	1.98739100	-2.72082200	-0.63523400
H	3.31223800	-3.04872700	0.52638500
I	-0.00594300	-0.06310800	-0.28965400

R1_cat_R2

C	2.71359800	1.82018200	0.00088400
H	1.64826000	2.01156300	-0.12302700
C	3.56961800	1.40709600	-0.95811200
H	3.31651700	1.19398400	-1.99701300
C	4.91667200	1.29503500	-0.38329600
H	5.80695700	0.99912500	-0.93811200
C	4.86967600	1.63803100	0.92151100
H	5.70982300	1.67390500	1.61324900
C	3.77686000	-1.37409600	1.67016100
C	2.61577400	-1.26970400	1.01629200
H	4.69651300	-1.68078700	1.16890100
H	1.70305800	-0.95514000	1.52993700
C	2.46570500	-1.55546000	-0.42752400
C	3.46830500	2.03932700	1.27856900
H	3.43398100	3.10185500	1.57961700
H	3.05909200	1.46461600	2.12628800
O	1.37782300	-1.42266000	-0.97974300
H	3.84613900	-1.15404400	2.73686900
C	3.65266600	-2.03627500	-1.21568700
H	4.48801600	-1.32873000	-1.11459000
H	3.98814500	-3.00925700	-0.82723900
H	3.36648200	-2.13806200	-2.26739400
C	-5.04284800	0.31319600	0.68489500
C	-4.69858600	1.52325000	0.16735000
C	-2.91982800	0.20172100	0.04924100
N	-3.92719600	-0.49299700	0.60296300
N	-3.37868400	1.43479500	-0.22182600
H	-5.97947300	-0.04131500	1.10074600
H	-5.27550100	2.43291300	0.04254000
C	-3.85067800	-1.88638700	1.04041300
H	-3.60302600	-2.52783200	0.18617600
H	-3.08463500	-1.98749000	1.81840800
H	-4.82778700	-2.16861800	1.44432300
C	-2.59993400	2.50784800	-0.83855800
H	-1.74367600	2.75670500	-0.20010400
H	-2.24910800	2.18750300	-1.82687500
H	-3.25008400	3.38200600	-0.94205700
I	-0.99156800	-0.51258100	-0.33919800

TS

C	3.14656800	1.56905700	0.32451400

H	2.19083800	2.01546500	0.04965600
C	4.24335800	1.44852500	-0.51665000
H	4.25417300	1.68033700	-1.58091900
C	5.30041300	0.85967300	0.20311500
H	6.26967600	0.58166600	-0.21074800
C	4.86181500	0.55910800	1.50043300
H	5.54026600	0.27227000	2.30435800
C	3.79665400	-1.18970200	1.17377800
C	2.62210300	-0.80104500	0.52923900
H	4.55064800	-1.75917500	0.63045100
H	1.73893600	-0.54390500	1.11749100
C	2.38129800	-0.99528300	-0.89232700
C	3.62536600	1.39341200	1.73340500
H	3.96493200	2.38319300	2.09235200
H	2.89882700	0.99821300	2.45088900
O	1.27318300	-0.79399300	-1.40739400
H	3.75219300	-1.41156100	2.24105500
C	3.49955900	-1.49612600	-1.76952300
H	4.45534300	-1.00888500	-1.53362000
H	3.62663400	-2.57619100	-1.59606900
H	3.23300600	-1.33501800	-2.81951500
C	-5.00961800	-0.10896300	1.09977200
C	-5.00724200	1.05978400	0.40405000
C	-3.01966000	0.10425000	0.13525900
N	-3.77007800	-0.68644700	0.92089800
N	-3.76630800	1.17398700	-0.18678300
H	-5.78125700	-0.57631100	1.70135100
H	-5.77635100	1.81381900	0.27873200
C	-3.33879300	-1.96405200	1.48535800
H	-3.08696700	-2.65916100	0.67547400
H	-2.46381800	-1.80764600	2.12736400
H	-4.16479900	-2.36944700	2.07790000
C	-3.32937300	2.27837200	-1.03910400
H	-2.46060700	2.77211000	-0.58770800
H	-3.06593200	1.89573800	-2.03242400
H	-4.15669700	2.98973200	-1.12375100
I	-1.04876400	-0.27770100	-0.49754500

Product

C	2.73624100	1.12846700	-0.03105900
H	1.75111900	1.42584100	-0.40903500
C	3.88490800	1.16635600	-1.02430700
H	3.78177200	1.10512800	-2.10774100
C	5.02205500	1.16706700	-0.31481000
H	6.03901000	1.10710500	-0.70236300
C	4.64518500	1.12258000	1.15467500
H	5.41957800	1.43166400	1.86362900
C	4.08075800	-0.31066900	1.41253900
C	2.74047800	-0.30611000	0.63297600
H	4.78163200	-1.08483600	1.07681400
H	1.88578800	-0.34981100	1.32587200
C	2.48628000	-1.37689900	-0.40456600
C	3.34034400	1.93985400	1.12856000
H	3.50447900	2.99050400	0.85870900
H	2.76169600	1.86748700	2.06150200
O	1.35161500	-1.54301000	-0.83617000
H	3.90108300	-0.46855900	2.48431100
C	3.61092200	-2.22102000	-0.92761900
H	4.46368700	-1.58692900	-1.20883700
H	3.95696200	-2.89238400	-0.12616500
H	3.26072900	-2.81405700	-1.77863900
C	-5.06028800	0.33320800	0.71818100
C	-4.75537600	1.48092800	0.05459800
C	-2.95191500	0.18857600	0.04291200
N	-3.92973800	-0.45594500	0.70043900
N	-3.44375100	1.37283100	-0.35684400
H	-5.97839000	0.01214700	1.19766700
H	-5.35491500	2.35880300	-0.15935200
C	-3.81191000	-1.78667300	1.29582800
H	-3.56670800	-2.51849200	0.51686800
H	-3.02819500	-1.77987800	2.06267500
H	-4.77389000	-2.03962400	1.75203100
C	-2.70409400	2.38153600	-1.11410500
H	-1.83122300	2.70959000	-0.53707300
H	-2.38180000	1.95956500	-2.07356600
H	-3.37174600	3.23078700	-1.28911400
I	-1.01888100	-0.53172400	-0.30692100

S2_1_cat

R1_cat

C	-1.42012200	-1.06450000	-0.17683900
C	0.57975600	0.02133700	-0.84396100
C	1.13451500	-1.12677300	-0.32665500
C	3.02912300	-0.02018000	-0.95972900
C	2.54107800	-1.17252300	-0.38127700
H	3.17098700	-1.98437800	-0.02558900
C	-0.87446200	0.05681700	-0.76139700
C	-2.82352000	-1.04027600	-0.06610800
H	-3.44732600	-1.81923600	0.36547100
C	-3.31861400	0.13387500	-0.59529800
C	4.39743100	0.31174300	-1.18039300
C	-4.68707000	0.52479900	-0.67414700
N	5.50844200	0.58934600	-1.35769000
N	-5.79778000	0.84815300	-0.74052800
S	1.77685100	1.10504900	-1.41106200
S	-2.07418400	1.19605600	-1.19537600
S	-0.13264600	-2.20174000	0.36174500
O	-0.03988700	-2.17159000	1.83235000
O	-0.20795500	-3.48226500	-0.34243200
C	2.02087200	0.49275200	2.62347700
C	1.59956300	1.60589700	2.01603100
H	1.33229400	-0.29772500	2.93428500
H	2.30132200	2.36824700	1.66670700
C	0.17385500	1.88733300	1.67923700
O	-0.08433900	2.71127900	0.82187700
H	3.08513400	0.32517700	2.80206200
C	-0.91068400	1.13061500	2.40763300
H	-0.73771000	0.04497300	2.37429100
H	-0.91077300	1.42822700	3.46751700
H	-1.88178400	1.37748400	1.96006400

R1_cat_R2

C	-2.24814300	-0.62393400	0.77518400
C	-0.47736400	-1.30608800	-0.64658700
C	-0.02160900	-1.86125000	0.52762000
C	1.55690200	-2.64877800	-0.92273000
C	1.14790400	-2.63595400	0.39399100
H	1.67242400	-3.14993200	1.19574100
C	-1.73711900	-0.58964800	-0.50242300
C	-3.44352400	0.10166900	0.94724300
H	-4.00082700	0.20921000	1.87462600
C	-3.83014800	0.66420900	-0.25108500
C	2.71523000	-3.28832400	-1.45240500
C	-4.99088900	1.45676800	-0.48802400
N	3.65639700	-3.80184900	-1.89237000
N	-5.93075600	2.10455200	-0.68783900
S	0.53002100	-1.70594100	-1.97068600
S	-2.71675500	0.33885500	-1.55270800

S	-1.12313700	-1.46907300	1.89587600
O	-0.50336400	-0.46750300	2.77840500
O	-1.70990500	-2.68560700	2.46113400
C	3.00624900	0.48700800	0.26263300
H	2.66861200	-0.23942900	-0.47490400
C	2.40654600	0.78213400	1.43598600
H	1.49790900	0.33368700	1.84081700
C	3.17783500	1.83643400	2.11048500
H	2.92236500	2.26405400	3.07979600
C	4.24182000	2.16692300	1.34863100
H	5.00727500	2.90371500	1.58769700
C	2.18330000	4.09674400	-0.50094200
C	1.70572900	3.11645800	-1.27220200
H	1.74953400	4.33300900	0.47201300
H	2.15087500	2.88353200	-2.24311600
C	0.55232100	2.24720500	-0.92010700
C	4.24822000	1.31588400	0.11297300
H	5.14813800	0.67600800	0.07651000
H	4.25228300	1.91150100	-0.81468900
O	0.21370000	1.36847600	-1.69488000
H	3.03588600	4.69900800	-0.82007100
C	-0.19988600	2.50983800	0.36369300
H	0.47877300	2.75542000	1.19020100
H	-0.87778500	3.36471000	0.20951200
H	-0.79070200	1.62840900	0.63871800

TS

C	2.23109500	0.49579200	0.79614000
C	0.48375700	1.31464900	-0.58172200
C	0.04224000	1.80902400	0.62495100
C	-1.55145100	2.67139300	-0.76493900
C	-1.12966000	2.58713900	0.54573100
H	-1.64361000	3.06017000	1.37906200
C	1.73053900	0.56712000	-0.48426300
C	3.43305000	-0.22890600	0.91518900
H	3.98586800	-0.40895000	1.83384800
C	3.83092900	-0.68656200	-0.32406600
C	-2.71187100	3.34452500	-1.24647600
C	4.99720200	-1.45244000	-0.61605600
N	-3.65693700	3.88390700	-1.64538600
N	5.94310300	-2.07580200	-0.86006100
S	-0.53691300	1.78493200	-1.87194600
S	2.72457200	-0.26164100	-1.60180000
S	1.13305600	1.30733100	1.96610400
O	0.46952300	0.28951200	2.79715500
O	1.76803900	2.46772800	2.59437500
C	-2.96251000	-0.64729700	0.09612300
H	-2.77148000	0.18667300	-0.57948400
C	-2.34143900	-0.84531500	1.32533000
H	-1.48781300	-0.29422900	1.71935000
C	-2.89524800	-1.99075900	1.92663600
H	-2.54654600	-2.44175800	2.85541600
C	-3.85051100	-2.54473800	1.06640800
H	-4.53693800	-3.33919600	1.36131900
C	-2.59735600	-3.50448200	-0.34385600
C	-2.05561700	-2.46488400	-1.09352600
H	-1.96751800	-4.06715600	0.34535600
H	-2.55595300	-2.12059300	-2.00021300
C	-0.65547200	-2.02126200	-1.00726900
C	-4.22659100	-1.45748300	0.09079000
H	-5.01543700	-0.84544000	0.56713900
H	-4.58710700	-1.78663300	-0.88919600
O	-0.19769300	-1.24459600	-1.83617800
H	-3.43736700	-4.06677300	-0.75394700
C	0.20452000	-2.57176700	0.11116200
H	-0.28467000	-2.44778700	1.08883800
H	0.36228900	-3.64966500	-0.04678600
H	1.17909900	-2.06899300	0.11918500

Product

C	2.24461000	0.42120300	0.81352400
C	0.50251500	1.29766300	-0.53637200
C	0.04070700	1.71682000	0.69128500
C	-1.52552000	2.67324100	-0.66871100
C	-1.12213200	2.51135500	0.64007600
H	-1.64282500	2.94225700	1.49195700
C	1.75476100	0.55477000	-0.46636100
C	3.44580200	-0.30723100	0.90772600
H	3.99062500	-0.53230000	1.82124000
C	3.85673400	-0.70053600	-0.34938400
C	-2.67262700	3.38380900	-1.12792600
C	5.02913800	-1.44554400	-0.66967500
N	-3.60451800	3.95757600	-1.50902300
N	5.98006000	-2.05132300	-0.93704700
S	-0.49903500	1.84733500	-1.81162300
S	2.76232700	-0.21388200	-1.61453400
S	1.12867600	1.16296300	2.01346100
O	0.47466000	0.09424800	2.78828200
O	1.74061300	2.29962400	2.70373600
C	-2.81362400	-0.80780600	0.01968500
H	-2.66143900	0.22093900	-0.32635900
C	-2.38592500	-1.12113000	1.44356700
H	-1.63437900	-0.57732400	2.01791500
C	-3.00790100	-2.25315500	1.80190900
H	-2.86193400	-2.81589500	2.72384100
C	-3.85086900	-2.70727000	0.62559000
H	-4.65215200	-3.42006400	0.84846800
C	-2.84605700	-3.19887800	-0.46493500
C	-2.17664600	-1.88207700	-0.94486600
H	-2.13541800	-3.92970800	-0.05752100
H	-2.50317800	-1.63455900	-1.96386700
C	-0.66334000	-1.81121600	-1.00966000
C	-4.25927700	-1.34211300	0.04158900
H	-4.90474500	-0.76833200	0.71846200
H	-4.71259600	-1.40902700	-0.95839200
O	-0.14133700	-1.27037400	-1.96835600
H	-3.37603200	-3.68062800	-1.29794800
C	0.18509400	-2.33510700	0.12752800
H	0.15354900	-1.61951900	0.96588100
H	-0.18622200	-3.28930500	0.52211300
H	1.22232400	-2.44174600	-0.21698100

S2_2_cat

R1_cat

C	-3.32989500	3.98916000	0.18955400
C	-2.12114600	3.51676300	-0.12155700
H	-4.03056500	3.42020300	0.80448400
H	-1.42300200	4.08513500	-0.74029600
H	-3.66292400	4.96550400	-0.16554000
C	-2.46821500	1.31235800	1.18601100
H	-2.79757300	1.85618000	2.08281200
H	-3.37096900	1.00318300	0.63601200
H	-1.89640200	0.42618100	1.48533100
S	2.09709400	0.56731800	-0.35525600
C	3.69835400	-0.01412400	-0.02819200
C	1.49223300	-0.95706600	0.19681100
C	2.54454900	-1.75463400	0.58999100
H	2.42925900	-2.77360100	0.95674800

C	0.08338100	-1.32863900	0.23773400
C	-0.56465600	-1.98498900	1.26125900
S	-1.01207400	-1.09872000	-1.07773400
H	-0.09629500	-2.24101100	2.21043400
C	-2.23947800	-1.88354500	-0.13518300
N	3.78858100	-1.21844900	0.47058100
N	-1.87445100	-2.29179100	1.05029800
C	-3.57385900	-2.00650800	-0.64721100
N	-4.64284500	-2.07255800	-1.08692500
C	4.83349000	0.82643000	-0.28321600
N	5.72512700	1.53119200	-0.50286800
C	-1.60390200	2.19114700	0.31375900
O	-0.49898700	1.83547100	-0.05228800

R1_cat_R2

C	2.87852300	1.01241300	0.28275000
H	3.08391100	0.23677100	-0.45407500
C	1.89821400	1.00582100	1.21040100
H	1.15783800	0.21799000	1.35908300
C	1.97759100	2.24247600	2.00126100
H	1.31205000	2.49486000	2.82680100
C	3.00237500	2.99311500	1.54726000
H	3.32120400	3.95881300	1.93645400
C	1.12401300	4.16128200	-1.12978200
C	0.90525200	2.94861900	-1.64481200
H	0.58117100	4.52736500	-0.25671200
H	1.45537900	2.58853900	-2.51810800
C	3.68758400	2.26743300	0.42646600
H	4.73661700	2.03610800	0.68359400
H	3.71727900	2.85628000	-0.50571000
H	1.86267600	4.83334600	-1.57059000
C	-0.95516400	2.34991100	0.05813500
H	-0.42667600	2.97041100	0.79255800
H	-1.82457800	2.91565400	-0.31283800
H	-1.30380900	1.42670500	0.53562300
S	0.78447500	-1.76023200	-0.95177700
C	1.84237700	-2.88473000	-0.16097000
C	-0.31716100	-1.95017600	0.36875200
C	0.21299700	-2.83633700	1.28360300
H	-0.29619800	-3.14736800	2.19467900
C	-1.58195500	-1.24167300	0.50404900
C	-2.14242500	-0.79298700	1.68330900
S	-2.63534500	-0.86507800	-0.80897500
H	-1.66816300	-0.89076700	2.65907000
C	-3.72500200	-0.14276900	0.32975400
N	1.43543900	-3.35304200	0.98886700
N	-3.34709500	-0.17179700	1.58089900
C	-4.94005500	0.47052000	-0.12275900
N	-5.90983900	0.96006000	-0.52278700
C	3.10168000	-3.22160100	-0.76081400
N	4.11155900	-3.45839000	-1.27499400
C	-0.07832000	1.96893000	-1.11235000
O	-0.18354400	0.88011800	-1.64840100

TS

C	3.09825300	-2.37359500	0.34545500
H	2.22138400	-2.97932800	0.11762900
C	3.29295800	-1.66029200	1.52346400
H	2.54218000	-1.50531700	2.29759600
C	4.55967400	-1.04925100	1.47829400
H	4.95382000	-0.35519000	2.22042400
C	5.16568300	-1.34902400	0.25268800
H	6.21012900	-1.13041200	0.02823400
C	4.11642000	-0.03267000	-1.05014200
C	2.84214900	-0.58344300	-1.13322500
H	4.27728900	0.89814200	-0.50635300
H	2.59490900	-1.29008000	-1.92714900
C	4.44200300	-2.55238200	-0.29859900
H	4.90750200	-3.45045900	0.14964200
H	4.44572800	-2.66536400	-1.38762400
H	4.82303800	-0.21379400	-1.86120500
C	1.85070500	1.12578900	0.50212800
H	2.70567600	0.94542600	1.16955500
H	2.06533700	2.04951900	-0.06018800
H	0.93669600	1.26641200	1.09272000
S	-2.14347600	-1.38740700	-0.53922600
C	-3.59660700	-2.16608300	-0.00056300
C	-2.64357800	0.02096200	0.33373600
C	-3.84551400	-0.22885400	0.95976200
H	-4.38335300	0.50839000	1.55419200
C	-1.90502900	1.27619900	0.39713000
C	-1.69922600	2.04803600	1.52006500
S	-1.25310600	2.08668700	-0.98030500
H	-2.00835000	1.75491600	2.52228100
C	-0.75470400	3.37467800	0.06816200
N	-4.37885200	-1.46665700	0.77872500
N	-1.04532500	3.22718400	1.33330200
C	-0.02191900	4.49044500	-0.45745000
N	0.57897400	5.36791700	-0.91515100
C	-3.87208200	-3.51993000	-0.39064400
N	-4.06016200	-4.61197800	-0.72563200
C	1.65485700	-0.01394500	-0.47334400
O	0.53582700	-0.43455400	-0.73218300

Product

C	3.14313700	-2.15728800	-0.07077700
H	2.36095600	-2.89214400	-0.28609500
C	3.42993400	-1.87254300	1.39330500
H	2.73022400	-2.03702200	2.21298400
C	4.62806500	-1.27463300	1.45615700
H	5.10615300	-0.84966700	2.33898500
C	5.14963000	-1.14882000	0.03601000
H	6.22627100	-0.97948200	-0.07164000
C	4.25928600	-0.06695200	-0.65514600
C	2.88048100	-0.76663400	-0.76555500
H	4.23655000	0.86431500	-0.07499900
H	2.62320400	-0.97334000	-1.81416200
C	4.56685600	-2.43232900	-0.58482500
H	4.99777800	-3.34495200	-0.15360100
H	4.63521800	-2.45589100	-1.68249600
H	4.65071500	0.17408800	-1.65294300
C	1.80233100	1.06488400	0.77007500
H	2.55945700	0.84283400	1.53593900
H	2.15741300	1.94683300	0.21152700
H	0.83698000	1.28985100	1.23960400
S	-2.20695800	-1.37993600	-0.47500700
C	-3.71820700	-2.09291900	-0.00927500
C	-2.72593500	0.08228500	0.29153900
C	-3.97640200	-0.10417500	0.83918900
H	-4.53325300	0.67464100	1.35832200
C	-1.95178200	1.31611300	0.34495700
C	-1.79507400	2.14141800	1.43706900
S	-1.17418000	2.02583800	-1.02374500
H	-2.18400900	1.91577700	2.42887800
C	-0.69741800	3.35096800	-0.01061300
N	-4.53200400	-1.33477400	0.67660000
N	-1.08354500	3.28449200	1.23516400
C	0.11546000	4.40734100	-0.54070500

N	0.78362500	5.23321900	-1.00108600
C	-4.00650000	-3.45543600	-0.35683900
N	-4.20454600	-4.55611000	-0.65568300
C	1.65057500	-0.09920400	-0.17629700
O	0.55290300	-0.53131600	-0.47310600

Se_cat

R1_cat

C	1.79088000	-0.80518400	0.09750100
C	2.89556700	-0.83210100	0.94448300
C	1.97781100	-0.37794300	-1.21635900
C	4.15604200	-0.45265600	0.49572500
C	3.22558500	0.01919700	-1.67724500
C	4.31713400	-0.02452200	-0.81604600
C	-0.75371100	0.34922800	0.36215800
C	-0.39335900	1.47626300	1.09599300
C	-1.74096300	0.48463100	-0.60875500
C	-1.01016900	2.70370200	0.88259600
C	-2.38644800	1.69573300	-0.81688900
C	-2.01686200	2.80840800	-0.07018200
F	3.38786300	0.43105200	-2.93004000
F	0.94868200	-0.32745600	-2.05853000
F	5.51432100	0.34487800	-1.24918500
F	5.20260500	-0.48665200	1.31297700
F	2.77210000	-1.22503100	2.20660200
F	0.56067800	1.40328100	2.01697100
F	-0.65471100	3.77144000	1.58822000
F	-2.62206700	3.97071400	-0.26999700
F	-3.35544300	1.79391500	-1.72450600
F	-2.11655600	-0.55393500	-1.35116800
C	-4.46066300	-3.40027500	-1.14667900
C	-3.58586800	-3.03835300	-0.20666100
H	-5.31259200	-2.77098800	-1.41290300
H	-2.73013400	-3.66590500	0.05360800
C	-3.66525400	-1.76842300	0.56378400
O	-2.81175600	-1.51313000	1.39340100
H	-4.35014600	-4.34022000	-1.68937700
C	-4.78506800	-0.79550700	0.27814000
H	-4.66236700	-0.38836400	-0.73780600
H	-5.76782200	-1.28404500	0.33184100
H	-4.73404900	0.02019500	1.00816100
Se	0.05664700	-1.34760800	0.71934700

R1_cat_R2

C	-2.56850600	-0.96841200	0.15903000
C	-3.61269300	-1.30335500	-0.69865600
C	-2.87681500	-0.28825400	1.33638400
C	-4.93014700	-0.98144200	-0.39061900
C	-4.18417800	0.05487400	1.65352400
C	-5.21229800	-0.29964400	0.78642300
C	-0.15728700	0.37830200	-0.29948600
C	-0.65478700	1.26418700	-1.25175600
C	0.79291500	0.84445200	0.60543100
C	-0.21542100	2.58212300	-1.31452600
C	1.24129600	2.15856500	0.55543100
C	0.74145200	3.02580100	-0.40957300
F	-4.46221700	0.70911400	2.77621100
F	-1.91035400	0.06414300	2.18037100
F	-6.46498800	0.01686000	1.08402100
F	-5.91747000	-1.31076100	-1.21647400
F	-3.37517700	-1.94272000	-1.83805600
F	-1.57632100	0.86854900	-2.12407200
F	-0.70085500	3.41604100	-2.22771100
F	1.16974400	4.27967400	-0.45773800
F	2.15211200	2.59847900	1.42254600
F	1.30182600	0.04480600	1.53390900
C	4.32784800	-1.10269000	1.71355300
H	3.36970400	-1.53919500	1.99057200
C	4.54802700	0.18273800	1.36627400
H	3.80073400	0.97435000	1.33584400
C	5.97175700	0.35834600	1.04922000
H	6.42907000	1.29887000	0.74217000
C	6.61094200	-0.81900100	1.21344700
H	7.67418000	-1.00574700	1.07003700
C	5.48216000	-1.63668500	-1.79550200
C	4.26941300	-2.02759400	-1.39640500
H	5.67912500	-0.61543500	-2.12747700
H	4.07613000	-3.04579500	-1.04951100
C	3.07706900	-1.14120200	-1.35085000
C	5.62785000	-1.85189900	1.68144300
H	5.89295900	-2.22186000	2.68824400
H	5.59438000	-2.73718700	1.02508300
O	2.03495400	-1.56769800	-0.88890700
H	6.32886700	-2.32537600	-1.79179300
C	3.19426100	0.26395200	-1.89442800
H	3.91455000	0.83828000	-1.29174400
H	3.56380300	0.24371900	-2.92975100
H	2.21200700	0.74991700	-1.86579700
Se	-0.75291100	-1.43910500	-0.25858300

TS

C	-2.58151400	-0.96803400	0.10182500
C	-3.63741200	-1.26435700	-0.75583500
C	-2.87364100	-0.32734800	1.30511100
C	-4.94918000	-0.94247600	-0.42407600
C	-4.17469400	0.01607000	1.64677600
C	-5.21430600	-0.29942500	0.77831300
C	-0.15948900	0.36858900	-0.30787200
C	-0.64413100	1.30114000	-1.22112600
C	0.78936800	0.78687300	0.62136100
C	-0.19293800	2.61681300	-1.22342100
C	1.24857200	2.09772000	0.63253400
C	0.76215700	3.01202800	-0.29483800
F	-4.43642000	0.63320100	2.79446500
F	-1.89656900	-0.01390900	2.15315100
F	-6.46144600	0.01696100	1.09956600
F	-5.94727800	-1.23527400	-1.25104100
F	-3.41693900	-1.86688400	-1.91871700
F	-1.56554200	0.95439500	-2.11370800
F	-0.66673700	3.49440500	-2.10113000
F	1.20171000	4.26314600	-0.28427300
F	2.15802800	2.49077600	1.52537900
F	1.28965100	-0.05703500	1.51696700
C	4.36782500	-1.28794000	1.41363900
H	3.47642300	-1.79166800	1.78708000
C	4.51328900	0.09110800	1.30381000
H	3.72168800	0.82180300	1.45792400
C	5.80894100	0.37257200	0.83256500
H	6.18333200	1.36083500	0.56592800
C	6.48606500	-0.83209800	0.61234100
H	7.55342000	-0.89312800	0.39735700
C	5.59412300	-1.45805000	-1.21997100
C	4.31200500	-1.84934200	-0.85185500
H	5.74489800	-0.54571400	-1.79721400
H	4.10741500	-2.88512000	-0.57657000
C	3.08983700	-1.08512900	-1.15444900
C	5.74424700	-1.88717100	1.39505100
H	6.13104200	-1.85928300	2.43127400

H	5.82217100	-2.91370300	1.02228900
O	1.98861000	-1.56689600	-0.93496800
H	6.35896300	-2.22175200	-1.36680200
C	3.23490700	0.29073700	-1.77009500
H	3.91694600	0.92042600	-1.17814300
H	3.66856100	0.19473000	-2.77723800
H	2.24883600	0.76411400	-1.85075200
Se	-0.77390800	-1.44259200	-0.35117800

Product

C	-2.61480500	-0.96038600	0.09292300
C	-3.66340900	-1.23652600	-0.78027000
C	-2.91238200	-0.32667100	1.29857800
C	-4.97486900	-0.90157500	-0.46087200
C	-4.21326600	0.02951700	1.62761600
C	-5.24617800	-0.26581900	0.74412800
C	-0.16714800	0.34988400	-0.27983300
C	-0.63648300	1.30109700	-1.18226800
C	0.79522200	0.73905000	0.64836900
C	-0.15443900	2.60554700	-1.17610800
C	1.29192600	2.03644200	0.66278800
C	0.81904600	2.96917800	-0.25327000
F	-4.48100400	0.64000900	2.77713100
F	-1.94133200	-0.03222800	2.15954700
F	-6.49279500	0.06318900	1.05321900
F	-5.96642300	-1.17481800	-1.30192600
F	-3.43562200	-1.83086700	-1.94581300
F	-1.56901100	0.98161400	-2.07359500
F	-0.61331700	3.50168800	-2.04265500
F	1.29097200	4.20801000	-0.23944700
F	2.21908000	2.39799900	1.54860300
F	1.27799700	-0.12447500	1.53469600
C	4.43523000	-1.52284700	1.01707100
H	3.65432000	-2.08576000	1.53810700
C	4.51572900	-0.03588700	1.31547000
H	3.69716500	0.57062000	1.70054700
C	5.72023400	0.38767700	0.90648000
H	6.08360400	1.41537800	0.88581600
C	6.45162000	-0.80834000	0.32433900
H	7.53953300	-0.71645000	0.23616500
C	5.72669000	-1.14075600	-1.01921500
C	4.34948200	-1.67454400	-0.55019100
H	5.66035000	-0.25586900	-1.66512700
H	4.24559600	-2.74748400	-0.76530000
C	3.07379000	-1.03803400	-1.07021000
C	5.90404800	-1.92984800	1.22757500
H	6.22148100	-1.82369500	2.27283600
H	6.13283800	-2.94007100	0.85658700
O	2.01843400	-1.60718100	-0.87188400
H	6.27368300	-1.91609500	-1.57325000
C	3.12956000	0.28872700	-1.78821600
H	3.68975100	1.02270600	-1.18871600
H	3.67081000	0.16685800	-2.73941100
H	2.11191600	0.64285600	-1.99354900
Se	-0.80891200	-1.45123400	-0.34255200

Te_cat

R1_cat

C	1.93315700	-0.74085400	-0.09390100
C	3.05728600	-1.01077600	0.67895600
C	2.10430600	0.04946500	-1.22720200
C	4.31200300	-0.51256500	0.34682300
C	3.34422300	0.57012600	-1.57557300
C	4.45093700	0.28300800	-0.78392400
C	-0.83100700	0.41598100	0.32866500
C	-0.41284800	1.43585700	1.17748100
C	-1.85343200	0.70313500	-0.56832800
C	-0.99909200	2.69702400	1.14824600
C	-2.47189300	1.94562400	-0.60000200
C	-2.03943300	2.94718700	0.26134000
F	3.48539500	1.32852700	-2.65820600
F	1.06570800	0.33481600	-2.01468400
F	5.64131300	0.76786200	-1.11108800
F	5.37341300	-0.78424000	1.09909400
F	2.95696900	-1.76789700	1.77172100
F	0.57002800	1.22854000	2.05046900
F	-0.58531200	3.65729800	1.96780700
F	-2.61867000	4.13965100	0.23599900
F	-3.47784600	2.17774000	-1.44174800
F	-2.29563500	-0.22350100	-1.42184200
Te	0.02678100	-1.50972600	0.44499900
C	-4.92890500	-2.86215600	-1.56092100
C	-3.91974200	-2.79028000	-0.69093900
H	-5.72857300	-2.11862300	-1.57717400
H	-3.11736700	-3.53170600	-0.68050200
C	-3.77331800	-1.71429800	0.32309500
O	-2.80700700	-1.71347300	1.06687600
H	-4.98571800	-3.66766500	-2.29439700
C	-4.80359000	-0.61396600	0.39614200
H	-4.76370800	-0.01933700	-0.53025100
H	-5.81966200	-1.02019800	0.49562800
H	-4.57171100	0.02681100	1.25426500

R1_cat_R2

C	2.67208000	-0.85248600	-0.27857400
C	3.73391500	-1.39803900	0.43442400
C	2.96911200	0.12059700	-1.22876500
C	5.04688300	-0.99395900	0.22138000
C	4.27023000	0.55277400	-1.45366800
C	5.31143600	-0.01190400	-0.72532800
C	0.03042400	0.50292400	0.29254600
C	0.55174300	1.31942900	1.29090800
C	-0.94740600	1.03860000	-0.53987600
C	0.11205700	2.62729000	1.47079300
C	-1.39954700	2.34246000	-0.38070800
C	-0.87223900	3.13581700	0.63229500
F	4.53036300	1.48933400	-2.36077500
F	1.99610000	0.67523700	-1.95450400
F	6.55928500	0.38565500	-0.93726200
F	6.04607600	-1.53153000	0.91398200
F	3.51405200	-2.34033500	1.35268700
F	1.49549200	0.86528400	2.11307700
F	0.62066400	3.39007300	2.43261400
F	-1.30313000	4.37979000	0.79144800
F	-2.33927900	2.84189800	-1.18318700
F	-1.48939500	0.30896400	-1.51274300
Te	0.67457900	-1.49412700	0.07753400
C	-4.49361500	-0.81132600	-1.80412900
H	-3.54728800	-1.18500200	-2.19155500
C	-4.70910300	0.40707500	-1.26467900
H	-3.96948700	1.19934700	-1.15792300
C	-6.11721500	0.51118000	-0.85960700
H	-6.56851000	1.38998400	-0.39900900
C	-6.75240400	-0.64066000	-1.16322700
H	-7.80588900	-0.86322600	-1.00005000
C	-5.51625100	-1.84182600	1.61999400
C	-4.31419600	-2.17329800	1.14155200
H	-5.70393400	-0.87501400	2.09090000

H	-4.13019200	-3.13553100	0.65752100
C	-3.12460200	-1.28700900	1.19103100
C	-5.78381400	-1.57767400	-1.82356700
H	-6.09311500	-1.79544300	-2.86177200
H	-5.71277000	-2.55152800	-1.31166800
O	-2.08848800	-1.65671000	0.66417700
H	-6.36283300	-2.52658900	1.54641200
C	-3.22789100	0.03525700	1.91169900
H	-3.97932600	0.67091600	1.41932700
H	-3.55350800	-0.12624600	2.94954200
H	-2.25297600	0.53595900	1.90807000

TS

C	2.51132615	0.83081546	0.24215470
C	3.57480915	1.33266046	-0.49991930
C	2.80504615	-0.10648254	1.22849870
C	4.88453715	0.92121846	-0.28045130
C	4.10235015	-0.54548854	1.46212770
C	5.14479215	-0.02428554	0.70378970
C	-0.13138085	-0.52091754	-0.27554030
C	0.39357015	-1.36823254	-1.24601330
C	-1.10871285	-1.03468754	0.57091270
C	-0.04142385	-2.68277054	-1.38581130
C	-1.55590385	-2.34432754	0.45204870
C	-1.02502985	-3.16851354	-0.53379230
F	4.35815415	-1.44750354	2.40539470
F	1.83116515	-0.62043254	1.98361470
F	6.38966815	-0.42857854	0.92270770
F	5.88507215	1.41678446	-1.00251030
F	3.36047315	2.23891446	-1.45533630
F	1.33734915	-0.93886754	-2.08122630
F	0.47100015	-3.47298954	-2.32320330
F	-1.45262685	-4.41832054	-0.65379230
F	-2.49542485	-2.82131054	1.27069970
F	-1.65913485	-0.27829754	1.52080670
Te	0.51448315	1.48237846	-0.12529130
C	-4.69844685	0.93388146	1.53999870
H	-3.82731785	1.35089246	2.04498970
C	-4.83905885	-0.39864154	1.16845770
H	-4.05929785	-1.15519454	1.23086270
C	-6.10745285	-0.57296354	0.58443770
H	-6.46958685	-1.49175754	0.12326970
C	-6.76974385	0.65980946	0.55274370
H	-7.82490885	0.76919746	0.30003770
C	-5.79402185	1.59241346	-1.08549530
C	-4.53138285	1.90809946	-0.59535430
H	-5.91277785	0.80057846	-1.82520330
H	-4.34490785	2.87527946	-0.12640530
C	-3.29899585	1.20109246	-0.96525730
C	-6.06924685	1.54494846	1.55396670
H	-6.51396185	1.33409846	2.54486270
H	-6.12302485	2.62321246	1.37188270
O	-2.20895385	1.64023546	-0.61651430
H	-6.55155985	2.37581146	-1.13266830
C	-3.40627985	-0.04789754	-1.81093230
H	-4.14152285	-0.75034654	-1.39018630
H	-3.75702085	0.23017246	-2.81675330
H	-2.42376985	-0.52681154	-1.89695930

Product

C	-2.70114300	-0.85771500	0.22836000
C	-3.76404400	-1.33743800	-0.52917600
C	-2.98967000	0.07052200	1.22485400
C	-5.07010400	-0.91256400	-0.31390900
C	-4.28345100	0.52205900	1.45393900
C	-5.32613100	0.02323800	0.68080100
C	-0.03099300	0.46730200	-0.25877000
C	-0.53988800	1.33417000	-1.22085000
C	0.95453300	0.95427300	0.59426800
C	-0.07949300	2.64122000	-1.34667800
C	1.43062500	2.25492200	0.48679000
C	0.91433800	3.09870900	-0.49041000
F	-4.53546900	1.41476400	2.40642200
F	-2.01486600	0.56225000	1.99245900
F	-6.56724800	0.43977700	0.89538900
F	-6.07071400	-1.38610900	-1.04983200
F	-3.55226100	-2.23328000	-1.49440100
F	-1.49080700	0.93037100	-2.06083000
F	-0.57661500	3.45075700	-2.27542600
F	1.36625700	4.34047100	-0.59907800
F	2.37690800	2.70506200	1.31050500
F	1.48689800	0.17701100	1.53753400
Te	-0.71299500	-1.52612400	-0.13095700
C	4.56788600	-1.25581500	1.18518800
H	3.79859800	-1.68877000	1.83243900
C	4.69237500	0.25758200	1.19571400
H	3.90493300	0.94834300	1.49407000
C	5.88766400	0.56273800	0.67063000
H	6.27251300	1.55784300	0.44651500
C	6.56781500	-0.74102100	0.29470600
H	7.65257500	-0.69873500	0.14940800
C	5.77940400	-1.29590800	-0.93505600
C	4.41631200	-1.70318600	-0.32087500
H	5.69799000	-0.54178700	-1.72835400
H	4.29161500	-2.79511800	-0.31890600
C	3.13061200	-1.15774600	-0.90722600
C	6.03588500	-1.65824200	1.41232400
H	6.39889500	-1.36722700	2.40624000
H	6.22752600	-2.72616400	1.23005200
O	2.08194200	-1.69412000	-0.59731600
H	6.28797100	-2.17207300	-1.36020200
C	3.16244400	0.03591200	-1.82716000
H	3.75740600	0.84631400	-1.37961900
H	3.66048700	-0.24893700	-2.76726400
H	2.14111400	0.36999200	-2.04554900

P_cat

R1_cat

C	1.93036000	-0.33109900	0.51518700
C	2.38631200	-0.61443000	-0.77128700
C	2.88190800	-0.20327200	1.52339900
C	3.73345900	-0.77390900	-1.05307300
C	4.24153500	-0.35406600	1.26691100
C	4.66282300	-0.64013800	-0.02425500
C	-0.13700900	1.40839000	-0.17333600
C	0.14598100	2.64193900	0.41607700
C	-0.63005300	1.42441800	-1.47708700
C	-0.06645600	3.84486200	-0.23997400
C	-0.85678200	2.61769700	-2.15304500
C	-0.57432200	3.82817900	-1.53294000
F	5.13255800	-0.22701200	2.24278200
F	2.51266900	0.06961600	2.77149300
F	5.95308900	-0.78822100	-0.28170400
F	4.14663100	-1.05261500	-2.28364300
F	1.51282200	-0.75398500	-1.76654900
F	0.65349900	2.67960500	1.64509100
F	0.21304200	5.00061700	0.34992600
F	-0.78365800	4.96630600	-2.17873800

F	-1.33524700	2.60640200	-3.39313400
F	-0.91809800	0.30026300	-2.13092600
C	-0.71774700	-1.43265900	0.09830400
C	-2.07006800	-1.28380000	-0.20402800
C	-0.19627300	-2.71758600	-0.03348100
C	-2.87131600	-2.33323900	-0.62143600
C	-0.97076700	-3.78990900	-0.46497500
C	-2.31506100	-3.60000000	-0.75471800
F	1.07942700	-2.97508100	0.26316800
F	-0.43136600	-4.99707800	-0.58656500
F	-3.06324900	-4.61899900	-1.15187100
F	-4.16001400	-2.13401000	-0.88030400
F	-2.63979200	-0.07699000	-0.10260900
C	-3.58529200	-0.97719300	2.84647700
C	-2.79086100	0.09475800	2.89893300
H	-4.51504600	-0.97657100	2.27175100
H	-1.85026000	0.08510500	3.45546400
C	-3.06022700	1.37477300	2.17712600
O	-2.16800300	2.19027100	2.05810300
H	-3.32695400	-1.89873200	3.37124900
C	-4.44077500	1.61808700	1.61444300
H	-4.46088500	2.60869000	1.14763300
H	-4.68367800	0.85333500	0.86316700
H	-5.19486000	1.56137700	2.41248500
P	0.15524600	-0.03703700	0.94975700

R1_cat_R2

C	1.93036000	-0.33109900	0.51518700
C	2.38631200	-0.61443000	-0.77128700
C	2.88190800	-0.20327200	1.52339900
C	3.73345900	-0.77390900	-1.05307300
C	4.24153500	-0.35406600	1.26691100
C	4.66282300	-0.64013800	-0.02425500
C	-0.13700900	1.40839000	-0.17333600
C	0.14598100	2.64193900	0.41607700
C	-0.63005300	1.42441800	-1.47708700
C	-0.06645600	3.84486200	-0.23997400
C	-0.85678200	2.61769700	-2.15304500
C	-0.57432200	3.82817900	-1.53294000
F	5.13255800	-0.22701200	2.24278200
F	2.51266900	0.06961600	2.77149300
F	5.95308900	-0.78822100	-0.28170400
F	4.14663100	-1.05261500	-2.28364300
F	1.51282200	-0.75398500	-1.76654900
F	0.65349900	2.67960500	1.64509100
F	0.21304200	5.00061700	0.34992600
F	-0.78365800	4.96630600	-2.17873800
F	-1.33524700	2.60640200	-3.39313400
F	-0.91809800	0.30026300	-2.13092600
C	-0.71774700	-1.43265900	0.09830400
C	-2.07006800	-1.28380000	-0.20402800
C	-0.19627300	-2.71758600	-0.03348100
C	-2.87131600	-2.33323900	-0.62143600
C	-0.97076700	-3.78990900	-0.46497500
C	-2.31506100	-3.60000000	-0.75471800
F	1.07942700	-2.97508100	0.26316800
F	-0.43136600	-4.99707800	-0.58656500
F	-3.06324900	-4.61899900	-1.15187100
F	-4.16001400	-2.13401000	-0.88030400
F	-2.63979200	-0.07699000	-0.10260900
C	-3.58529200	-0.97719300	2.84647700
C	-2.79086100	0.09475800	2.89893300
H	-4.51504600	-0.97657100	2.27175100
H	-1.85026000	0.08510500	3.45546400
C	-3.06022700	1.37477300	2.17712600
O	-2.16800300	2.19027100	2.05810300
H	-3.32695400	-1.89873200	3.37124900
C	-4.44077500	1.61808700	1.61444300
H	-4.46088500	2.60869000	1.14763300
H	-4.68367800	0.85333500	0.86316700
H	-5.19486000	1.56137700	2.41248500
P	0.15524600	-0.03703700	0.94975700

TS

C	-2.42594734	-0.53987297	-0.66109413
C	-3.38555434	-0.07094297	0.23567187
C	-2.88797934	-1.37845797	-1.68124513
C	-4.73123334	-0.40533897	0.12337987
C	-4.22259734	-1.73516097	-1.81157513
C	-5.14979434	-1.24026597	-0.90304513
C	-0.61206834	1.62492003	-0.07983413
C	-0.45916634	2.63889903	-1.02687413
C	-0.65756134	2.00954303	1.26062987
C	-0.36301134	3.97740003	-0.66701113
C	-0.55529734	3.33837203	1.64530987
C	-0.41009534	4.32440303	0.67576787
F	-4.61803334	-2.54098497	-2.79064413
F	-2.03463034	-1.88438997	-2.56820213
F	-6.42845234	-1.56730897	-1.01419413
F	-5.61589934	0.06651703	0.99551087
F	-3.05881534	0.72547703	1.24925187
F	-0.41270134	2.34711103	-2.32110813
F	-0.22440034	4.92095403	-1.59303513
F	-0.31711534	5.59649303	1.03673987
F	-0.59412934	3.67685403	2.93075087
F	-0.77830434	1.10665503	2.23031687
C	0.13801966	-1.11770797	0.58042987
C	1.18587166	-0.71041797	1.40733687
C	-0.15229734	-2.48439697	0.59831287
C	1.88159366	-1.59496197	2.22137087
C	0.51315266	-3.38818097	1.41543887
C	1.54408166	-2.94011097	2.23101487
F	-1.08670334	-2.98146097	-0.21733013
F	0.18780166	-4.67600397	1.40075387
F	2.21108666	-3.79322697	2.99693187
F	2.89534966	-1.15904597	2.96330487
F	1.60239466	0.55784903	1.43627487
C	4.69708966	-0.60793897	-2.69053613
H	3.87824666	-0.55047597	-3.40739713
C	5.63807766	0.39232203	-2.47053413
H	5.59004866	1.40201303	-2.87684913
C	6.57046066	-0.06414097	-1.52002213
H	7.37011166	0.53272103	-1.08170413
C	6.20999966	-1.35226097	-1.11156313
H	6.84562366	-1.99141397	-0.49802613
C	4.57033566	-0.91504897	0.19744187
C	3.56068466	-0.50682697	-0.66661713
H	5.06084866	-0.19089597	0.84799487
H	2.84019466	-1.23084897	-1.05524213
C	3.10738266	0.89266203	-0.79651113
C	5.27565566	-1.89029097	-2.16612313
H	4.55623666	-2.64760997	-1.83775413
H	5.90034566	-2.32458797	-2.96941713
O	2.06717566	1.16128903	-1.37684513
H	4.56402566	-1.93755397	0.57943687
C	3.93024066	1.97921003	-0.14213213
H	3.60540766	2.95349803	-0.52474313
H	3.74560666	1.93887603	0.94282887

H	5.00798166	1.83943803	-0.30380013
P	-0.61541034	-0.08681997	-0.77570413

Product

C	-2.32568600	-0.28179100	-0.87481100
C	-3.16153600	-0.55061800	0.20808300
C	-2.91992200	-0.11579800	-2.12265900
C	-4.53510200	-0.66047500	0.06473900
C	-4.29760100	-0.21688300	-2.29403300
C	-5.10169400	-0.49000400	-1.19627800
C	-0.52621800	1.37058600	0.45396800
C	-0.61021300	2.61965600	-0.16433400
C	-0.43641500	1.35551400	1.84467400
C	-0.58200200	3.80684300	0.55106100
C	-0.39751800	2.53288000	2.58369000
C	-0.47025700	3.75889200	1.93520900
F	-4.84403400	-0.05480700	-3.49298800
F	-2.17941800	0.14700100	-3.19518900
F	-6.41351900	-0.59059000	-1.34538200
F	-5.31325200	-0.92640200	1.10731500
F	-2.63730400	-0.72524200	1.42003500
F	-0.73905100	2.68689200	-1.48591100
F	-0.66323900	4.97803900	-0.06881100
F	-0.44216000	4.88219400	2.63825900
F	-0.30152900	2.49026800	3.90898200
F	-0.37438800	0.21586800	2.53185100
C	0.02753300	-1.48322500	0.31056500
C	1.22850300	-1.39386100	1.01287000
C	-0.56282700	-2.74449300	0.26534400
C	1.81475800	-2.47358100	1.65232000
C	-0.00500900	-3.84704200	0.90517000
C	1.19123900	-3.71462100	1.59668600
F	-1.69565500	-2.95042700	-0.40931700
F	-0.60654500	-5.02885000	0.84379300
F	1.73650000	-4.76294800	2.19584900
F	2.96775200	-2.32961200	2.29684500
F	1.85677500	-0.21324800	1.10021300
C	4.17022600	0.35100800	-2.65236300
H	3.53277300	0.75150100	-3.44708700
C	5.44182500	1.12153500	-2.34215200
H	5.60226600	2.17849000	-2.55578800
C	6.24893600	0.30099000	-1.65512400
H	7.20300100	0.55221100	-1.19126700
C	5.52201200	-1.02204500	-1.49330200
H	6.14368000	-1.88823400	-1.24244800
C	4.35881800	-0.75408200	-0.48625800
C	3.39872200	0.15714000	-1.29373700
H	4.72233100	-0.29597200	0.44252600
H	2.46223600	-0.36925200	-1.53485300
C	2.96616100	1.49110500	-0.70595600
C	4.74189900	-1.06831000	-2.82076500
H	3.96598800	-1.84801600	-2.84410000
H	5.39891200	-1.15465800	-3.69575200
O	1.97986000	2.03957600	-1.15427400
H	3.86317600	-1.69712700	-0.21341200
C	3.76326200	2.11906700	0.41229500
H	3.41317800	3.14564300	0.56907700
H	3.60619400	1.53337100	1.33114800
H	4.83939400	2.10188500	0.19063100
P	-0.49345000	-0.04666600	-0.74358500

As_cat

C	-2.80894800	-0.10675500	-0.59337800
C	-3.56895300	0.58147600	0.35055900
C	-3.52502700	-0.88168400	-1.51036700
C	-4.95635200	0.48476900	0.40224000
C	-4.90740300	-1.00412100	-1.47971300
C	-5.62689400	-0.31420100	-0.51258500
C	-0.37974000	1.66296000	-0.05388700
C	0.07214000	2.68990800	-0.87881200
C	-0.34691500	1.88961900	1.32049700
C	0.53855500	3.89589200	-0.36753000
C	0.10535600	3.08666400	1.85702600
C	0.54999200	4.09330800	1.00576800
F	-5.54331000	-1.76267100	-2.36587200
F	-2.88383300	-1.54382200	-2.47423300
F	-6.94753800	-0.41323000	-0.46790600
F	-5.64278200	1.15684600	1.32120000
F	-3.00942100	1.38493400	1.25309900
F	0.08170200	2.54281200	-2.20037200
F	0.99229000	4.84543900	-1.18120500
F	0.99233400	5.23719300	1.50931900
F	0.12871700	3.27738200	3.17261900
F	-0.72082000	0.94237100	2.17699200
C	-0.18384100	-1.32964000	0.35998200
C	0.91337800	-1.17141000	1.20519300
C	-0.71740600	-2.61492300	0.28887200
C	1.44217100	-2.22384600	1.94305500
C	-0.22328700	-3.68411800	1.02472100
C	0.87192400	-3.48620300	1.85498300
F	-1.74065900	-2.86239900	-0.53400300
F	-0.77475500	-4.88836500	0.92473700
F	1.37217400	-4.49567300	2.55474300
F	2.49997600	-2.02785500	2.72623900
F	1.52800700	0.00605400	1.35405000
As	-0.84909100	-0.01303000	-0.97059300
C	5.10685000	-0.94074800	-2.35665100
H	4.42165100	-1.14926600	-3.17804100
C	5.77580200	0.26130500	-2.15861100
H	5.59291200	1.18261800	-2.71047000
C	6.61445800	0.13342500	-1.03461100
H	7.19593700	0.93844300	-0.58554300
C	6.46003300	-1.14697000	-0.49439800
H	7.10548200	-1.55605200	0.28332600
C	4.58821000	-0.93336300	0.51644300
C	3.65925700	-0.86831000	-0.51452100
H	4.81172100	-0.04380900	1.10589800
H	3.16788000	-1.77509000	-0.87456300
C	2.94673100	0.36110600	-0.89896700
C	5.83449500	-1.99476600	-1.57359800
H	5.23639500	-2.84752700	-1.23618600
H	6.65539600	-2.37296600	-2.21189600
O	1.93171600	0.30233300	-1.57957000
H	4.72375400	-1.87639100	1.04783900
C	3.44069600	1.69828200	-0.39224300
H	3.10609800	2.48545400	-1.08052600
H	2.97780900	1.87288000	0.59338000
H	4.53082900	1.73595500	-0.26854000

Sb_cat

R1_cat

C	2.30336000	-0.58476500	-0.36117800
C	2.59125000	-1.46249700	0.68015900
C	3.40316400	-0.00038500	-0.98763700
C	3.89128800	-1.70657500	1.11209200
C	4.71394100	-0.21576900	-0.58344300
C	4.95483300	-1.07498600	0.48109500
C	-0.85321800	-1.54143700	-0.22531500

C	-1.59851000	-2.44047300	-0.97526200
C	-1.02982500	-1.56831300	1.15399800
C	-2.48568400	-3.33984900	-0.39366600
C	-1.89793900	-2.45837200	1.76957800
C	-2.62821300	-3.34872600	0.98691400
F	5.72932500	0.37435600	-1.20537600
F	3.21949000	0.79856100	-2.04706500
F	6.19711300	-1.30235600	0.88431400
F	4.12484500	-2.54712000	2.11558900
F	1.62875500	-2.14063500	1.30945300
F	-1.50336500	-2.44797500	-2.30855600
F	-3.20970500	-4.16423100	-1.14642800
F	-3.46925900	-4.19720700	1.56230800
F	-2.05225800	-2.46377600	3.08985300
F	-0.38375400	-0.70075300	1.93313700
C	-0.12381800	1.59309300	-0.00986100
C	-1.29937800	1.78799800	0.70752100
C	0.76053300	2.66512100	-0.03164600
C	-1.58993500	2.98109200	1.35762900
C	0.51710400	3.86775000	0.62006300
C	-0.67532600	4.02578300	1.31508200
F	1.90222300	2.55800800	-0.72262100
F	1.39631500	4.86209000	0.57251000
F	-0.94177800	5.16917200	1.93154400
F	-2.74149900	3.13308000	2.00715700
F	-2.23265700	0.82928000	0.80004500
C	-4.66760700	2.60656400	-0.56157300
C	-3.68641600	2.20875400	-1.37436800
H	-5.32996500	1.89137100	-0.06876600
H	-3.00322700	2.92078500	-1.84471800
C	-3.35932000	0.78253200	-1.63962300
O	-2.29017400	0.50924100	-2.16008100
H	-4.82969600	3.66338000	-0.34442100
C	-4.31997400	-0.30354000	-1.22595900
H	-4.04838600	-1.23190700	-1.74265900
H	-4.22545600	-0.45615500	-0.13857500
H	-5.36162900	-0.03751600	-1.44737000
Sb	0.36517200	-0.10796700	-1.31378900

R1_cat_R2

C	-2.88385900	0.05566200	-0.75150200
C	-3.61103900	0.91635800	0.06561400
C	-3.63478300	-0.82439100	-1.52932000
C	-4.99948700	0.87246200	0.14794100
C	-5.02007600	-0.90077300	-1.47302200
C	-5.70459700	-0.04469100	-0.61987700
C	-0.14973100	1.76272000	-0.03955900
C	0.54536000	2.75085400	-0.72281900
C	-0.33449800	1.94894200	1.32624200
C	1.04082200	3.88604900	-0.09096000
C	0.13751600	3.07314600	1.98838200
C	0.82839000	4.04629800	1.27119200
F	-5.69112500	-1.76531100	-2.22727200
F	-3.01895900	-1.63747900	-2.39824300
F	-7.02776800	-0.09368300	-0.54876000
F	-5.65741100	1.70788900	0.94642000
F	-3.01154000	1.85409400	0.80295700
F	0.78884700	2.62209700	-2.03115100
F	1.73429000	4.79512100	-0.77198700
F	1.29272600	5.12091000	1.89438200
F	-0.04900400	3.22762100	3.29547100
F	-0.95209400	1.02020600	2.05630200
C	-0.22018500	-1.42783300	0.44897100
C	0.74508400	-1.27163900	1.43839200
C	-0.82367300	-2.67767200	0.37839100
C	1.10151500	-2.29872600	2.30385000
C	-0.50923700	-3.72534800	1.23495400
C	0.46869300	-3.53132700	2.20216300
F	-1.74567400	-2.90749000	-0.56502300
F	-1.11692800	-4.90201400	1.12969200
F	0.80022600	-4.51918100	3.02275800
F	2.04915600	-2.11350000	3.21889800
F	1.39905300	-0.11327800	1.60401000
C	5.41568800	-1.06240500	-2.64578200
H	4.64800000	-1.37256100	-3.35307900
C	5.74414100	0.20711100	-2.32653200
H	5.28828100	1.10890000	-2.73532300
C	6.82396000	0.18862000	-1.33050700
H	7.28857000	1.07613900	-0.90075500
C	7.14558800	-1.09190600	-1.05100600
H	7.91711600	-1.43116900	-0.36155800
C	4.51361100	-1.29652400	1.02879200
C	3.62195900	-1.24173600	0.03579200
H	4.92013900	-0.39323500	1.48736100
H	3.20170300	-2.14630200	-0.41324900
C	3.05769800	0.01904100	-0.50789800
C	6.29897700	-2.00781400	-1.88600500
H	5.72554900	-2.72784500	-1.27871400
H	6.92268700	-2.60664800	-2.57358600
O	2.11298400	-0.03754400	-1.28070500
H	4.85840900	-2.25240100	1.42746000
C	3.60024600	1.34923400	-0.05024100
H	3.31920800	2.11689100	-0.78206400
H	3.13062700	1.59072000	0.91793400
H	4.68944100	1.33042300	0.08314100
Sb	-0.70875000	-0.01370300	-1.16082200

TS

C	-2.78675857	-0.07362933	-0.61068837
C	-3.50077857	0.74351067	0.26101163
C	-3.54131657	-1.00372533	-1.32399037
C	-4.87197257	0.61039467	0.45767863
C	-4.90973657	-1.16950833	-1.15462937
C	-5.57663257	-0.35531933	-0.24862337
C	-0.12227157	1.80686867	-0.12183037
C	0.45202343	2.83475267	-0.85669837
C	-0.21190257	1.98635867	1.25403463
C	0.92119843	4.00196967	-0.26463137
C	0.23885543	3.14078267	1.87849263
C	0.80733043	4.15311167	1.11040763
F	-5.58279757	-2.07956433	-1.85232037
F	-2.94982057	-1.77937433	-2.24310137
F	-6.88402157	-0.48923833	-0.06924037
F	-5.51575157	1.40658567	1.30685163
F	-2.90698057	1.72580267	0.94387163
F	0.59696343	2.71764867	-2.18053337
F	1.49512843	4.95384667	-0.99691137
F	1.24901843	5.25847567	1.69621363
F	0.14563443	3.28835567	3.19673763
F	-0.71223257	1.02426767	2.02967363
C	0.05220743	-1.38674833	0.36435463
C	1.07256743	-1.16783833	1.28418063
C	-0.45777357	-2.67875333	0.31814163
C	1.56863643	-2.17520233	2.10294063
C	-0.00189157	-3.70949233	1.13069563
C	1.02791643	-3.45220433	2.02643763
F	-1.42654057	-2.96946933	-0.55932337
F	-0.52450157	-4.92858133	1.04878063

F	1.49619943	-4.42149133	2.80240463
F	2.56882043	-1.92871433	2.94702763
F	1.64948743	0.03294367	1.42211863
C	5.27765043	-1.14334333	-2.21972537
H	4.60207243	-1.38374033	-3.04034437
C	5.95116043	0.06132467	-2.06338537
H	5.78792043	0.95739567	-2.66101537
C	6.76793943	-0.02140033	-0.91882737
H	7.35040943	0.79807867	-0.49803137
C	6.59199743	-1.27393133	-0.32206637
H	7.22373943	-1.65221433	0.48204763
C	4.72336743	-0.99135933	0.65607363
C	3.79809143	-0.97528833	-0.38090437
H	4.94767243	-0.07376433	1.20036763
H	3.30614243	-1.89735533	-0.69820137
C	3.10577243	0.23450867	-0.83598137
C	5.97595843	-2.16542833	-1.37143337
H	5.36164143	-2.99465133	-1.00584537
H	6.80449743	-2.58333533	-1.97408837
O	2.09117643	0.14630467	-1.52330037
H	4.84409443	-1.90534133	1.23956563
C	3.60528943	1.59385467	-0.40728237
H	3.29472143	2.33932467	-1.15090437
H	3.12229043	1.83550067	0.55434463
H	4.69274043	1.62585667	-0.26243837
Sb	-0.64555757	-0.00722833	-1.19948137

Product

C	-2.89482100	0.03183800	-0.56444100
C	-3.51794400	0.78550000	0.42576200
C	-3.72978100	-0.78922000	-1.32043100
C	-4.88331800	0.70037900	0.68087700
C	-5.09483000	-0.90472900	-1.09465000
C	-5.67226500	-0.15223000	-0.07974000
C	-0.15235600	1.78552500	-0.10049800
C	0.34399200	2.86890400	-0.81196200
C	-0.12317000	1.87652400	1.28723300
C	0.84656500	4.00592700	-0.18976000
C	0.36844700	2.99643900	1.94347000
C	0.85314200	4.06706100	1.19677800
F	-5.84767600	-1.71082900	-1.83627100
F	-3.22008200	-1.50568800	-2.33157500
F	-6.97422800	-0.23997300	0.15546500
F	-5.44021500	1.43393700	1.64010500
F	-2.83560100	1.65208100	1.17848100
F	0.38430700	2.83360800	-2.14875200
F	1.34459800	5.00953400	-0.90692900
F	1.32987100	5.14005600	1.81277800
F	0.38635600	3.05898600	3.27100400
F	-0.55650500	0.86633500	2.04168700
C	-0.08748900	-1.43688600	0.18855700
C	0.99045200	-1.32695500	1.06088100
C	-0.72400000	-2.67169700	0.15779100
C	1.40158700	-2.37772500	1.87194600
C	-0.34518800	-3.74600500	0.95367100
C	0.73155300	-3.59396400	1.81697300
F	-1.74986400	-2.85997700	-0.68164500
F	-0.98932700	-4.90546400	0.88985500
F	1.12112400	-4.60238700	2.58401300
F	2.43806500	-2.23367600	2.69669700
F	1.69912200	-0.19172100	1.16363400
C	4.91776300	-1.25968300	-1.82445200
H	4.46933400	-1.59762300	-2.76454900
C	5.92191000	-0.12524400	-1.91527400
H	5.96954900	0.60729800	-2.72123900
C	6.63444200	-0.12914800	-0.77986400
H	7.38015800	0.60288800	-0.46906800
C	6.10474300	-1.25696700	0.08628800
H	6.76181200	-1.59782200	0.89327600
C	4.68715300	-0.80525800	0.56340300
C	3.84240400	-0.87731600	-0.73476500
H	4.71089800	0.19832100	1.00868000
H	3.12666200	-1.71151100	-0.69807400
C	3.01482500	0.32793400	-1.11826200
C	5.72868200	-2.27971300	-1.00194200
H	5.12015900	-3.11516700	-0.62544500
H	6.60173900	-2.65655300	-1.54973300
O	1.96934100	0.16148100	-1.72530300
H	4.28546800	-1.49034300	1.32121700
C	3.45653900	1.71851400	-0.73885500
H	3.01066400	2.44295500	-1.43190700
H	3.06525700	1.91467400	0.27502700
H	4.54778200	1.81997500	-0.70635100
Sb	-0.79074400	0.05876100	-1.25958300

BENZALDEHYDE (R1) + 2,3-DIMETHYL BUTADIENE (R2)

Uncat endo

R1

C	0.052451	-1.095647	0.000034
C	0.530043	0.219480	0.000078
C	-0.362628	1.292968	0.000061
C	-1.735922	1.056072	0.000007
C	-2.209949	-0.255393	-0.000034
C	-1.316972	-1.331424	-0.000021
C	1.993591	0.471294	0.000146
O	2.828780	-0.399198	-0.000184
H	-3.284562	-0.442937	-0.000077
H	2.284029	1.550249	-0.000162
H	0.023003	2.315352	0.000094
H	-2.436848	1.891153	-0.000004
H	-1.696779	-2.353615	-0.000052
H	0.777233	-1.910720	0.000049

R2

C	0.445887	1.431267	1.191426
C	0.013829	0.744816	0.126904
C	-0.013829	-0.744816	0.126904
C	-0.445887	-1.431267	1.191426
H	0.444549	2.523161	1.194781
H	0.822640	0.917944	2.077435
C	-0.445887	1.434830	-1.131767
C	0.445887	-1.434830	-1.131767
H	-0.444549	-2.523161	1.194781
H	-0.822640	-0.917944	2.077435
H	0.461518	-2.524153	-0.999348
H	-0.221242	-1.201675	-1.976302
H	1.455757	-1.099604	-1.415945
H	-0.461518	2.524153	-0.999348
H	0.221242	1.201675	-1.976302
H	-1.455757	1.099604	-1.415945

R1_R2

C	-1.155240	0.156533	-1.269989
C	-1.318619	0.907001	-0.100573
C	-2.154958	0.449905	0.919413

C	-2.838296	-0.756238	0.772860
C	-2.672641	-1.505628	-0.391942
C	-1.828778	-1.052287	-1.411146
C	-0.597711	2.195865	0.055161
O	0.074083	2.702781	-0.810718
H	-3.203997	-2.451417	-0.508138
C	1.870501	0.871159	1.687716
C	1.617399	-0.250553	0.998768
C	2.211334	-0.495384	-0.345939
C	2.424109	0.502504	-1.214597
C	0.732793	-1.334518	1.558170
C	2.536705	-1.925388	-0.695265
H	-0.724398	2.687268	1.050202
H	2.549815	1.633471	1.303105
H	1.412489	1.042318	2.664686
H	2.877028	0.300869	-2.187967
H	2.131400	1.529962	-0.994624
H	3.198568	-2.378411	0.059588
H	1.622511	-2.538892	-0.733036
H	3.030778	-1.985668	-1.673336
H	0.280994	-1.022076	2.508880
H	-0.075054	-1.585361	0.851852
H	1.307819	-2.256738	1.737169
H	-2.268509	1.043794	1.829902
H	-3.496440	-1.114288	1.565080
H	-1.704058	-1.645077	-2.318165
H	-0.491203	0.540177	-2.045276

TS

C	-2.113510	-1.421610	-0.975808
C	-1.132947	-0.965396	-0.091188
C	-1.487125	-0.611403	1.213343
C	-2.818487	-0.696723	1.620737
C	-3.798458	-1.139204	0.731904
C	-3.442274	-1.508393	-0.566096
C	0.287881	-0.806234	-0.540406
O	1.147299	-0.583492	0.428774
C	0.339750	0.599613	-1.717358
C	-0.253671	1.651266	-0.988351
C	0.408349	2.260890	0.131009
C	1.701820	1.928660	0.396469
C	-1.695612	1.967848	-1.212889
C	-0.381697	3.142806	1.066065
H	-0.702518	-0.286820	1.897756
H	-3.090519	-0.426665	2.642082
H	-4.838162	-1.209353	1.053398
H	-4.202029	-1.871061	-1.259344
H	-1.835082	-1.715100	-1.991308
H	0.613549	-1.543718	-1.292028
H	-0.222261	0.264020	-2.592929
H	1.422582	0.573857	-1.844734
H	2.337566	1.383964	-0.298645
H	2.175175	2.251180	1.326382
H	-0.729558	4.054087	0.556686
H	-1.270580	2.622799	1.453763
H	0.237022	3.449142	1.917901
H	-2.074751	1.525756	-2.140347
H	-1.854937	3.056096	-1.228215
H	-2.288226	1.568931	-0.368796
B	2.602112	-1.029690	0.206114
F	3.321691	-0.436799	1.222089
F	2.622769	-2.406084	0.202504
F	2.994391	-0.523056	-1.057590

Product

C	-2.198019	-1.012799	-0.201702
C	-1.143849	-0.173293	-0.583657
C	-1.233579	1.189053	-0.294321
C	-2.361121	1.706071	0.347051
C	-3.408027	0.864712	0.713098
C	-3.322191	-0.501493	0.438981
C	0.048608	-0.818736	-1.279837
C	1.204232	0.116780	-1.605086
C	1.989244	0.488797	-0.367087
C	1.890004	-0.268254	0.735895
C	1.023702	-1.505111	0.747920
O	0.542986	-1.910959	-0.514657
C	2.569127	-0.011155	2.051624
C	2.847023	1.713950	-0.513720
H	-4.288089	1.270018	1.213990
H	-0.317868	-1.283753	-2.206947
H	1.870347	-0.388482	-2.323710
H	0.838187	1.024466	-2.111250
H	0.179946	-1.341041	1.449899
H	1.606304	-2.355604	1.135301
H	3.320581	-0.789573	2.262114
H	3.063535	0.965013	2.098463
H	1.831630	-0.052587	2.869594
H	3.481390	1.620099	-1.410152
H	2.220772	2.609117	-0.666553
H	3.502577	1.896092	0.344973
H	-0.415651	1.860860	-0.558258
H	-2.415972	2.773482	0.565176
H	-4.135680	-1.169146	0.726416
H	-2.116810	-2.082419	-0.406756

BF3

R1_cat

C	-2.071573	-1.279921	-0.000867
C	-1.173344	-0.205526	-0.002474
C	-1.638793	1.118394	-0.001835
C	-3.005313	1.358258	0.000276
C	-3.901844	0.283938	0.002174
C	-3.440517	-1.032756	0.001731
C	0.254413	-0.488084	-0.004671
O	1.098683	0.409047	-0.002873
H	-0.914572	1.933483	-0.003227
H	-3.380962	2.381346	0.000475
H	-4.975199	0.478598	0.004004
H	-4.147774	-1.861710	0.003346
H	-1.691573	-2.303540	-0.001399
H	0.594828	-1.537356	-0.008296
B	2.749553	0.035117	0.001190
F	3.181116	0.650031	-1.129130
F	3.157171	0.570563	1.180282
F	2.755029	-1.338882	-0.044916

R1_cat_R2

C	1.747472	-1.739464	0.994889
C	0.872961	-1.252527	0.016679
C	1.344740	-0.897108	-1.255027
C	2.697647	-1.036923	-1.541643
C	3.569266	-1.537305	-0.569110
C	3.098252	-1.889404	0.697067
C	-0.540623	-1.103093	0.334486
O	-1.363027	-0.800810	-0.537056
C	-0.306670	1.452406	1.955343
C	0.593059	1.777090	1.010262

C	0.201876	2.246769	-0.351684
C	-1.051264	2.126435	-0.815565
C	2.068787	1.666387	1.296357
C	1.281567	2.864247	-1.206506
H	0.638363	-0.508680	-1.989670
H	3.078417	-0.761212	-2.525265
H	4.629126	-1.650523	-0.801073
H	3.785834	-2.274744	1.449679
H	1.364132	-1.997371	1.984380
H	-0.888776	-1.284732	1.364428
H	0.030393	1.103328	2.934387
H	-1.382372	1.536826	1.800777
H	-1.856390	1.682072	-0.230886
H	-1.310495	2.477540	-1.815990
H	1.712374	3.754396	-0.722061
H	2.111848	2.162097	-1.382277
H	0.876035	3.168391	-2.179590
H	2.244983	1.278446	2.308038
H	2.562626	2.646579	1.213032
H	2.564123	0.996656	0.574405
B	-2.958655	-0.705944	-0.115930
F	-3.493836	0.060253	-1.103453
F	-3.353257	-2.010735	-0.078151
F	-2.922368	-0.100399	1.126103

TS

C	2.113510	-1.421610	0.975808
C	1.132947	-0.965396	0.091188
C	1.487125	-0.611403	-1.213343
C	2.818487	-0.696723	-1.620737
C	3.798458	-1.139204	-0.731904
C	3.442274	-1.508393	0.566096
C	-0.287881	-0.806234	0.540406
O	-1.147299	-0.583492	-0.428774
C	-0.339750	0.599613	1.717358
C	0.253671	1.651266	0.988351
C	-0.408349	2.260890	-0.131009
C	-1.701820	1.928660	-0.396469
C	1.695612	1.967848	1.212889
C	0.381697	3.142806	-1.066065
H	0.702518	-0.286820	-1.897756
H	3.090519	-0.426665	-2.642082
H	4.838162	-1.209353	-1.053398
H	4.202029	-1.871061	1.259344
H	1.835082	-1.715100	1.991308
H	-0.613549	-1.543718	1.292028
H	0.222261	0.264020	2.592929
H	-1.422582	0.573857	1.844734
H	-2.337566	1.383964	0.298645
H	-2.175175	2.251180	-1.326382
H	0.729558	4.054087	-0.556686
H	1.270580	2.622799	-1.453763
H	-0.237022	3.449142	-1.917901
H	2.074751	1.525756	2.140347
H	1.854937	3.056096	1.228215
H	2.288226	1.568931	0.368796
B	-2.602112	-1.029690	-0.206114
F	-3.321691	-0.436799	-1.222089
F	-2.622769	-2.406084	-0.202504
F	-2.994391	-0.523056	1.057590

Product

C	-2.199827	-1.242767	-1.045642
C	-1.196500	-0.723079	-0.221930
C	-1.476717	-0.477132	1.122450
C	-2.748644	-0.736813	1.632600
C	-3.748704	-1.243390	0.805624
C	-3.470232	-1.499522	-0.537036
C	0.143861	-0.401423	-0.840136
O	1.150786	-0.277100	0.205388
C	0.114586	0.888935	-1.662149
C	-0.140579	2.062391	-0.746739
C	0.643941	2.129055	0.339186
C	1.688559	1.057139	0.478095
C	-1.291925	2.960372	-1.079669
C	0.595813	3.148756	1.438762
H	-0.691061	-0.096035	1.774773
H	-2.955965	-0.545613	2.686136
H	-4.741514	-1.448057	1.207585
H	-4.242584	-1.909502	-1.188722
H	-1.981560	-1.458007	-2.094752
H	0.479645	-1.240481	-1.462144
H	-0.666253	0.795291	-2.428504
H	1.082425	0.988159	-2.181100
H	2.530043	1.200643	-0.219038
H	2.084439	0.984379	1.496472
H	-0.116021	3.955216	1.231720
H	0.311628	2.673466	2.391607
H	1.587399	3.603145	1.591388
H	-1.181558	3.363037	-2.098669
H	-1.398726	3.800866	-0.384973
H	-2.227057	2.376075	-1.063796
B	2.351572	-1.402276	0.064230
F	3.124956	-1.166020	1.164078
F	1.674009	-2.584303	0.043398
F	2.943345	-1.066292	-1.132223

H_cat

R1_cat

C	-3.848963	-1.359460	-0.000711
C	-2.733018	-0.514863	-0.000271
C	-2.899001	0.877216	0.000638
C	-4.177588	1.416569	0.001094
C	-5.291117	0.569085	0.000642
C	-5.130574	-0.816170	-0.000264
C	-1.393688	-1.115086	-0.000739
O	-0.346771	-0.488317	-0.000545
H	-6.294452	0.996706	0.001002
H	-1.374040	-2.227500	-0.001307
H	-3.709526	-2.442897	-0.001417
H	-6.003184	-1.468876	-0.000606
H	-4.317595	2.497500	0.001809
H	-2.017520	1.519106	0.001002
C	2.634501	-0.221120	-0.000028
N	2.780709	1.104351	-0.000392
N	3.846970	-0.779591	0.000352
C	4.125038	1.405054	-0.000271
C	1.677817	2.068245	-0.000715
C	4.796908	0.219648	0.000177
C	4.122006	-2.215629	0.001013
H	4.487275	2.427433	-0.000594
H	1.747290	2.691740	-0.899422
H	0.739808	1.501789	-0.000821
H	1.746975	2.691981	0.897849
H	5.859899	0.003862	0.000368
H	3.168492	-2.752931	-0.000368
H	4.693565	-2.477191	-0.896796
H	4.690897	-2.476966	0.900590

H	1.668606	-0.731466	-0.000037

R1_cat_R2

C	3.432633	1.001202	-1.305757
C	2.277361	1.260922	-0.560196
C	2.311885	1.209278	0.839870
C	3.500342	0.893360	1.486060
C	4.654565	0.636440	0.737892
C	4.624186	0.689910	-0.655941
C	1.032206	1.577865	-1.273354
C	0.118633	-1.502721	-1.562580
C	0.859824	-1.877319	-0.511317
C	0.269346	-1.937909	0.854855
C	-0.458404	-0.916422	1.326630
O	-0.035738	1.827570	-0.738862
C	0.561966	-3.167475	1.671645
C	2.304668	-2.274820	-0.646746
H	5.587286	0.395250	1.249385
H	1.110786	1.578339	-2.383471
H	-0.940755	-1.267798	-1.436076
H	0.542202	-1.453232	-2.568235
H	-0.900734	-0.957107	2.325548
H	-0.568791	-0.005381	0.734626
H	1.635041	-3.225631	1.910671
H	0.002701	-3.158551	2.615449
H	0.305465	-4.080849	1.113812
H	2.661731	-2.130337	-1.674448
H	2.932481	-1.677434	0.034519
H	2.445215	-3.333591	-0.379871
H	3.394949	1.046191	-2.396817
H	5.528017	0.491573	-1.231708
H	3.537684	0.851840	2.574727
H	1.402436	1.418093	1.403642
C	-2.808904	0.857590	-0.465900
N	-3.046064	1.175771	0.806607
N	-3.693719	-0.066854	-0.843794
C	-4.110385	0.428826	1.260709
C	-2.270441	2.140996	1.587570
C	-4.518598	-0.353836	0.222883
C	-3.758802	-0.692133	-2.163367
H	-4.485838	0.517666	2.274307
H	-1.985081	1.674053	2.537330
H	-1.371555	2.393705	1.013871
H	-2.877337	3.035184	1.772481
H	-5.318370	-1.082934	0.152446
H	-3.015747	-0.216065	-2.811314
H	-3.536536	-1.761817	-2.071737
H	-4.760184	-0.546805	-2.584515
H	-1.993177	1.270121	-1.061155

TS

C	-1.842332	-1.700551	-1.530004
C	-1.310756	-0.863270	-0.545448
C	-1.603301	-1.112580	0.799236
C	-2.427022	-2.181452	1.153511
C	-2.960626	-3.010301	0.165897
C	-2.663959	-2.769639	-1.176687
C	-0.458816	0.324260	-0.909618
C	-1.636939	1.623151	-1.589478
C	-2.570868	1.753161	-0.540721
C	-2.144159	2.211648	0.733293
C	-0.842462	2.631589	0.860822
O	0.256049	0.855831	0.030881
C	-3.031674	2.027336	1.938836
C	-3.916209	1.107959	-0.669662
H	-3.604443	-3.846007	0.441309
H	0.024005	0.215800	-1.902059
H	-0.861676	2.382178	-1.705693
H	-2.025056	1.246608	-2.539484
H	-0.410865	2.778865	1.853334
H	-0.278481	3.043797	0.030662
H	-3.399413	0.993800	2.022537
H	-2.495606	2.283616	2.861189
H	-3.913078	2.683271	1.875161
H	-4.162548	0.883116	-1.713480
H	-3.934073	0.163339	-0.096438
H	-4.697817	1.755668	-0.246821
H	-1.615138	-1.512655	-2.583011
H	-3.074512	-3.418988	-1.950675
H	-2.654965	-2.369486	2.203818
H	-1.182283	-0.449064	1.557838
C	3.081430	0.220046	0.038581
N	3.120344	-1.002016	0.572239
N	4.325329	0.564479	-0.306885
C	4.421606	-1.453396	0.568582
C	1.947368	-1.726973	1.067145
C	5.181846	-0.467134	0.015608
C	4.713764	1.831791	-0.921171
H	4.695273	-2.428070	0.957636
H	2.140481	-2.058855	2.093523
H	1.096294	-1.036862	1.036619
H	1.751338	-2.590440	0.420088
H	6.248618	-0.410683	-0.171794
H	3.815685	2.443478	-1.052561
H	5.421691	2.353592	-0.266936
H	5.174849	1.639400	-1.896784
H	2.152262	0.796836	-0.084840

Product

C	-0.830552	1.408242	-1.190240
C	0.304254	0.890452	-0.561075
C	1.023432	1.710164	0.319786
C	0.620735	3.024200	0.547201
C	-0.517047	3.533039	-0.085090
C	-1.245726	2.720801	-0.952886
C	0.738052	-0.537398	-0.831068
C	2.079819	-0.626787	-1.577177
C	3.258056	-0.456250	-0.645934
C	3.209443	-1.169865	0.490249
C	1.960580	-1.992100	0.690447
O	0.762937	-1.248344	0.412412
C	4.246327	-1.235242	1.572172
C	4.348212	0.475949	-1.080891
H	-0.829141	4.562444	0.094095
H	-0.036062	-1.005039	-1.470577
H	2.141708	-1.615677	-2.063009
H	2.082478	0.123777	-2.379345
H	1.860960	-2.316340	1.734519
H	1.980866	-2.898473	0.055937
H	3.844209	-0.844163	2.520841
H	4.541693	-2.279791	1.759255
H	5.152467	-0.671901	1.324644
H	4.746318	0.167059	-2.060179
H	3.944664	1.493582	-1.209669
H	5.182193	0.522410	-0.372084
H	-1.398769	0.776947	-1.880552
H	-2.130161	3.112419	-1.457366
H	1.195330	3.656966	1.224766

H	1.898680	1.307464	0.831776
C	-2.206878	-1.215874	0.453406
N	-2.512842	-0.113863	1.134617
N	-3.228794	-1.514009	-0.353382
C	-3.761474	0.316262	0.749523
C	-1.621577	0.557646	2.081293
C	-4.216015	-0.566315	-0.185028
C	-3.286859	-2.656183	-1.263138
H	-4.217860	1.206478	1.168496
H	-2.064032	0.521753	3.083837
H	-0.657108	0.038184	2.056476
H	-1.480315	1.594453	1.749728
H	-5.149463	-0.602859	-0.736163
H	-2.335832	-3.195710	-1.206028
H	-4.105368	-3.321741	-0.965385
H	-3.446509	-2.298483	-2.286848
H	-1.249694	-1.736347	0.525580

H2_cat

R1_cat

C	-1.726832	-0.987264	1.943260
C	-1.294373	-2.042126	1.132348
C	-2.190874	-3.034649	0.714974
C	-3.517807	-2.974546	1.122048
C	-3.953375	-1.911249	1.920981
C	-3.065171	-0.914088	2.325284
C	0.092666	-2.031079	0.633171
H	-5.002341	-1.854485	2.214842
H	0.779911	-1.319473	1.149837
H	-1.020889	-0.204689	2.234307
H	-3.417579	-0.071757	2.921666
H	-4.225766	-3.740001	0.803320
H	-1.825712	-3.837026	0.071880
O	0.482598	-2.694461	-0.310220
C	-4.743435	0.834624	-0.319925
C	-3.809299	1.804135	0.016953
C	-4.299609	-0.294731	-1.006304
H	-5.792570	0.950047	-0.049188
C	-2.459394	1.683699	-0.314810
C	-4.241982	2.985585	0.841575
C	-2.961950	-0.452749	-1.328202
C	-5.296956	-1.357258	-1.377005
C	-2.025607	0.530653	-0.974088
H	-1.757803	2.467944	-0.048715
F	-5.530474	3.289373	0.640404
F	-4.102209	2.726195	2.153802
F	-3.516141	4.076668	0.578925
H	-2.634406	-1.365286	-1.829078
F	-6.072259	-1.685532	-0.329481
F	-6.119610	-0.946852	-2.349689
F	-4.702614	-2.481014	-1.802163
N	-0.691292	0.259350	-1.327126
C	0.490184	0.661075	-0.757188
H	-0.605572	-0.498385	-1.998973
S	0.661397	1.840003	0.404672
N	1.539993	-0.080451	-1.261042
H	1.287827	-1.024812	-1.551077
C	2.873044	-0.007853	-0.792532
C	3.485302	-1.182136	-0.362651
C	3.590473	1.193721	-0.813366
C	4.821919	-1.154650	0.040127
H	2.913552	-2.113946	-0.339182
C	4.910555	1.198975	-0.388001
H	3.117032	2.109360	-1.160889
C	5.544140	0.028679	0.037672
C	5.458683	-2.446922	0.471560
C	5.688321	2.487115	-0.365642
H	6.586027	0.044576	0.354542
F	6.694078	-2.263484	0.950491
F	4.736854	-3.050366	1.426822
F	5.545921	-3.312086	-0.549003
F	6.939248	2.307433	-0.813924
F	5.788865	2.977285	0.877042
F	5.116396	3.431213	-1.122175

R1_cat_R2

C	1.689859	0.709941	1.364587
C	1.251881	1.531394	0.321031
C	2.107578	2.481202	-0.249294
C	3.403371	2.614072	0.239138
C	3.848561	1.781006	1.271622
C	2.998945	0.823862	1.829406
C	-0.097601	1.319183	-0.233672
C	-1.647024	2.990787	1.962371
C	-0.525550	3.723700	1.872784
C	-0.318464	4.731293	0.791499
C	-0.960013	4.660004	-0.383612
C	0.669230	5.837064	1.066798
C	0.577309	3.575555	2.890267
H	4.874415	1.870992	1.631422
H	-0.787567	0.721932	0.406487
H	-2.486429	3.126893	1.278941
H	-1.760289	2.247676	2.754859
H	-0.802161	5.422568	-1.148608
H	-1.651958	3.851145	-0.619286
H	1.680381	5.433128	1.233663
H	0.713395	6.538514	0.224160
H	0.393650	6.399817	1.972223
H	0.359334	2.755900	3.587112
H	1.546819	3.374736	2.406940
H	0.693472	4.501115	3.475472
H	1.012933	-0.041337	1.781067
H	3.359766	0.156977	2.613483
H	4.081820	3.347654	-0.197720
H	1.732521	3.102091	-1.064633
O	-0.440896	1.702337	-1.338585
C	4.777031	-1.414081	-0.275218
C	3.887242	-2.348373	0.235394
C	4.304274	-0.500301	-1.216398
H	5.814832	-1.390800	0.055697
C	2.551977	-2.400542	-0.167043
C	4.345534	-3.283448	1.320712
C	2.977922	-0.511565	-1.615026
C	5.257542	0.512085	-1.788747
C	2.085577	-1.454584	-1.082175
H	1.885266	-3.153178	0.241887
F	5.661835	-3.520414	1.252550
F	4.105731	-2.757675	2.535050
F	3.713026	-4.460150	1.272040
H	2.620417	0.243959	-2.317062
F	6.008684	1.075247	-0.827773
F	6.106027	-0.046618	-2.661234
F	4.619467	1.500725	-2.430802
N	0.753031	-1.357594	-1.525940
C	-0.423800	-1.633389	-0.874924
H	0.656496	-0.770390	-2.349890
S	-0.571795	-2.491192	0.542852
N	-1.487316	-1.066746	-1.549465

H	-1.237999	-0.241662	-2.094212
C	-2.802462	-0.965479	-1.036996
C	-3.374921	0.304828	-0.966718
C	-3.539797	-2.089875	-0.656267
C	-4.686417	0.446937	-0.518478
H	-2.785140	1.178814	-1.253834
C	-4.837606	-1.919290	-0.189297
H	-3.102011	-3.083179	-0.723991
C	-5.427438	-0.657255	-0.117036
C	-5.306862	1.816575	-0.510593
C	-5.622172	-3.116753	0.275652
H	-6.449391	-0.541947	0.242144
F	-6.294337	1.912014	0.386045
F	-4.400512	2.768012	-0.222438
F	-5.824137	2.130194	-1.706046
F	-6.924069	-2.992714	-0.019032
F	-5.536798	-3.273686	1.603302
F	-5.182573	-4.249157	-0.286559

TS

C	1.972776	1.064080	1.686089
C	1.365526	1.742676	0.626987
C	2.155853	2.461556	-0.275498
C	3.538516	2.526521	-0.099778
C	4.140035	1.853889	0.965423
C	3.356431	1.111761	1.851046
C	-0.121207	1.670526	0.410543
C	-0.922581	2.876078	1.632156
C	-0.288156	4.083997	1.288304
C	-0.469616	4.635911	-0.007981
C	-1.364227	4.019787	-0.847954
C	0.464550	5.709925	-0.507941
C	0.817586	4.606850	2.154861
H	5.223287	1.881275	1.085048
H	-0.589415	0.812336	0.938344
H	-1.938220	2.683838	1.280399
H	-0.723736	2.487750	2.634329
H	-1.372014	4.260749	-1.913014
H	-2.224528	3.468271	-0.479614
H	1.520307	5.432861	-0.368545
H	0.294636	5.907123	-1.573895
H	0.300006	6.653318	0.035004
H	0.795219	4.159403	3.155116
H	1.795382	4.377796	1.695136
H	0.751298	5.700711	2.248066
H	1.361056	0.469811	2.370940
H	3.825371	0.547579	2.658547
H	4.154981	3.077724	-0.811927
H	1.666602	2.947902	-1.122024
O	-0.555921	1.925195	-0.780901
C	4.789974	-1.617751	-0.151661
C	3.796714	-2.295981	0.541508
C	4.398497	-0.749184	-1.168940
H	5.841087	-1.749363	0.102758
C	2.442919	-2.141311	0.244879
C	4.170327	-3.138086	1.729477
C	3.061647	-0.577125	-1.486824
C	5.454973	0.025324	-1.904504
C	2.065402	-1.266000	-0.777849
H	1.693517	-2.682872	0.814686
F	5.432136	-3.581143	1.654949
F	4.075203	-2.424135	2.867988
F	3.370247	-4.199052	1.870744
H	2.781375	0.120546	-2.276704
F	6.254639	0.690598	-1.051475
F	6.249624	-0.774047	-2.626779
F	4.929360	0.932567	-2.740590
N	0.744021	-0.926068	-1.105086
C	-0.452767	-1.536729	-0.814673
H	0.669721	-0.007035	-1.539296
S	-0.619151	-3.057732	-0.158373
N	-1.492134	-0.704796	-1.159236
H	-1.217340	0.276074	-1.304503
C	-2.839410	-0.791576	-0.773193
C	-3.450109	0.430243	-0.463256
C	-3.593101	-1.968640	-0.710394
C	-4.778902	0.476373	-0.069015
H	-2.852963	1.341134	-0.522628
C	-4.926651	-1.894176	-0.311160
H	-3.144742	-2.926639	-0.956209
C	-5.534822	-0.688402	0.028074
C	-5.395948	1.808920	0.244646
C	-5.753690	-3.152320	-0.282379
H	-6.570566	-0.660835	0.364343
F	-6.288134	1.728427	1.236591
F	-4.459896	2.711312	0.615896
F	-6.027771	2.332326	-0.813482
F	-6.435016	-3.312960	-1.427079
F	-6.659322	-3.121477	0.706316
F	-5.002513	-4.245088	-0.114065

Product

C	1.773462	0.946882	1.888893
C	1.277446	1.828683	0.924915
C	2.179944	2.576200	0.159065
C	3.552704	2.458844	0.375776
C	4.038746	1.581408	1.345387
C	3.146367	0.818279	2.097527
C	-0.221235	1.955874	0.735588
C	-0.820189	3.118464	1.538957
C	-0.412129	4.438154	0.929117
C	-0.579515	4.536941	-0.398545
C	-1.146448	3.317912	-1.082405
C	-0.256984	5.711681	-1.274063
C	0.187838	5.465310	1.839781
H	5.113892	1.471523	1.490347
H	-0.691844	1.015048	1.073034
H	-1.920420	3.019118	1.535071
H	-0.487899	3.030839	2.582201
H	-0.991439	3.361581	-2.168684
H	-2.236808	3.252634	-0.899703
H	0.546182	5.456255	-1.985054
H	-1.134262	5.998758	-1.875282
H	0.059417	6.590392	-0.701328
H	-0.497485	5.685341	2.673310
H	1.115514	5.067799	2.283380
H	0.425050	6.406342	1.330851
H	1.077047	0.331243	2.465048
H	3.519532	0.097434	2.826529
H	4.250304	3.035928	-0.233250
H	1.802213	3.243735	-0.616282
O	-0.528171	2.087787	-0.662389
C	4.889141	-1.534206	-0.244404
C	3.928403	-2.271850	0.434735
C	4.453087	-0.573083	-1.153967
H	5.951163	-1.693581	-0.060294
C	2.563150	-2.084356	0.224635
C	4.354792	-3.228037	1.513920

C	3.102662	-0.372559	-1.390282
C	5.475799	0.290685	-1.835243
C	2.141117	-1.121515	-0.696967
H	1.838921	-2.670359	0.783002
F	5.612407	-3.653863	1.339160
F	4.308272	-2.635360	2.722620
F	3.562367	-4.302104	1.579794
H	2.787412	0.392778	-2.100167
F	6.168646	1.019412	-0.939485
F	6.374037	-0.434838	-2.511825
F	4.919520	1.155016	-2.695194
N	0.803490	-0.762369	-0.927310
C	-0.366884	-1.446546	-0.708955
H	0.702751	0.196031	-1.260029
S	-0.478687	-3.033258	-0.220507
N	-1.437695	-0.623365	-0.967178
H	-1.199755	0.364568	-1.090336
C	-2.791797	-0.799767	-0.644337
C	-3.475928	0.365280	-0.266253
C	-3.491590	-2.005978	-0.722246
C	-4.825912	0.324792	0.039274
H	-2.932024	1.308400	-0.209257
C	-4.848089	-2.023421	-0.391836
H	-2.990108	-2.916744	-1.036885
C	-5.532730	-0.876359	-0.010264
C	-5.544783	1.589714	0.415173
C	-5.569437	-3.344721	-0.437293
H	-6.592838	-0.915094	0.236612
F	-6.272947	1.433745	1.526915
F	-4.688481	2.605881	0.632503
F	-6.385699	1.986249	-0.548999
F	-5.211127	-4.063234	-1.509141
F	-6.899077	-3.183440	-0.481380
F	-5.296450	-4.089267	0.641885

Cl_cat

R1_cat

C	-3.244308	-0.287119	-0.921782
C	-2.587521	0.679550	-0.155327
C	-2.185007	0.387962	1.155051
C	-2.437868	-0.870356	1.692166
C	-3.074997	-1.844690	0.914599
C	-3.474410	-1.557026	-0.391351
C	-2.308962	2.021035	-0.729275
O	-1.508932	2.794089	-0.252614
H	-3.279829	-2.828534	1.339112
H	-2.875689	2.279319	-1.653040
H	-3.577552	-0.042948	-1.933370
H	-3.986758	-2.313937	-0.985748
H	-2.156197	-1.094505	2.721717
H	-1.701483	1.171150	1.741608
C	1.956685	0.047390	-0.107333
N	3.259498	0.055485	0.195258
N	1.601407	-1.182146	-0.494883
C	3.754465	-1.217446	-0.006495
C	4.006993	1.225224	0.662241
C	2.719506	-1.989232	-0.438069
C	0.251032	-1.573468	-0.912530
H	4.795641	-1.460935	0.174317
H	3.606494	1.556140	1.627768
H	5.054662	0.930970	0.774476
H	3.923555	2.030650	-0.076698
H	2.681160	-3.038569	-0.708883
H	-0.022857	-1.024718	-1.821839
H	0.263890	-2.648594	-1.115478
H	-0.462922	-1.349734	-0.109038
Cl	0.913491	1.366895	-0.025176

R1_cat_R2

C	1.262553	1.871513	-1.225348
C	1.094850	0.585498	-0.705643
C	0.898036	0.399473	0.669446
C	0.872640	1.503219	1.518566
C	1.018236	2.792907	0.993135
C	1.207546	2.978950	-0.377576
C	1.134051	-0.594011	-1.608852
C	4.247182	-1.009521	-1.582847
C	4.157718	-0.488205	-0.349338
C	3.841605	-1.331306	0.839418
C	3.082066	-2.432892	0.745726
O	0.700034	-1.681273	-1.295168
C	4.409838	-0.883817	2.161884
C	4.417339	0.975902	-0.101758
H	1.003549	3.656050	1.660300
H	1.567194	-0.415375	-2.618255
H	4.134687	-2.080244	-1.760625
H	4.480503	-0.375750	-2.441456
H	2.892072	-3.050073	1.626363
H	2.634018	-2.748797	-0.197194
H	4.015418	0.103362	2.451050
H	4.164402	-1.599946	2.955951
H	5.505251	-0.787807	2.108140
H	4.557792	1.516178	-1.047156
H	3.586690	1.442556	0.452390
H	5.325257	1.112392	0.505771
H	1.444422	2.004112	-2.294883
H	1.338334	3.983935	-0.779952
H	0.752576	1.364148	2.593962
H	0.809056	-0.618422	1.051964
C	-3.324461	-0.372980	-0.049429
N	-4.498251	-0.893324	0.325875
N	-3.424429	0.960011	-0.084720
C	-5.380491	0.147104	0.538713
C	-4.772502	-2.323682	0.478123
C	-4.709908	1.303533	0.281600
C	-2.339112	1.872873	-0.459372
H	-6.403678	-0.026535	0.853128
H	-4.130781	-2.739278	1.263961
H	-5.824157	-2.435920	0.757796
H	-4.583180	-2.835349	-0.472740
H	-5.034053	2.337286	0.327307
H	-2.040281	1.677568	-1.496375
H	-2.717111	2.895390	-0.365704
H	-1.481926	1.723203	0.210324
Cl	-1.931804	-1.240391	-0.427618

TS

C	1.358901	1.997789	-1.140888
C	1.180754	0.699423	-0.656540
C	0.987860	0.477321	0.713825
C	0.971191	1.557926	1.592494
C	1.125439	2.860331	1.102054
C	1.315559	3.081911	-0.263091
C	1.204291	-0.453047	-1.594251
C	4.296929	-0.948258	-1.633841
C	4.187795	-0.527113	-0.363763
C	3.792987	-1.447142	0.742524
C	3.052400	-2.544897	0.527405

O	0.762927	-1.545501	-1.310114
C	4.260218	-1.086484	2.129827
C	4.504515	0.899946	0.006124
H	1.117726	3.705110	1.792373
H	1.632149	-0.248127	-2.600735
H	4.145515	-1.993681	-1.907197
H	4.587132	-0.256527	-2.427681
H	2.806349	-3.215405	1.353368
H	2.675584	-2.807403	-0.461199
H	3.849562	-0.115323	2.449490
H	3.952106	-1.848466	2.856602
H	5.356883	-0.997478	2.166985
H	4.714065	1.499922	-0.888973
H	3.673705	1.368677	0.558024
H	5.389066	0.945309	0.659839
H	1.538640	2.158092	-2.206946
H	1.453821	4.096372	-0.638203
H	0.852354	1.390925	2.664101
H	0.895041	-0.550713	1.068378
C	-3.248042	-0.360365	-0.006643
N	-4.428880	-0.913876	0.290566
N	-3.379622	0.970120	-0.036361
C	-5.349351	0.101843	0.456317
C	-4.674264	-2.352369	0.412670
C	-4.694186	1.277339	0.251220
C	-2.299067	1.913534	-0.343652
H	-6.385117	-0.100647	0.705650
H	-4.074308	-2.758887	1.235406
H	-5.738558	-2.494967	0.621707
H	-4.409367	-2.849243	-0.527971
H	-5.047251	2.302023	0.285935
H	-1.965744	1.762993	-1.377770
H	-2.696478	2.925704	-0.222221
H	-1.458729	1.752181	0.344537
Cl	-1.816964	-1.187862	-0.324001

Product

C	0.985644	2.190882	-1.137109
C	1.204850	0.988473	-0.456511
C	1.146317	0.981149	0.941073
C	0.892716	2.160720	1.643950
C	0.683868	3.358493	0.958026
C	0.727834	3.370280	-0.437415
C	1.563524	-0.263742	-1.238416
C	3.066377	-0.288724	-1.565840
C	3.861956	-0.572052	-0.314159
C	3.431336	-1.613314	0.415548
C	2.211628	-2.322772	-0.119767
O	1.167480	-1.422904	-0.515254
C	4.019005	-2.136375	1.692661
C	4.999882	0.348281	0.007153
H	0.497993	4.281342	1.508822
H	0.996311	-0.257445	-2.185909
H	3.244559	-1.073939	-2.320033
H	3.349716	0.670123	-2.021130
H	1.760945	-2.968602	0.646186
H	2.479369	-2.963774	-0.980791
H	3.299585	-2.035418	2.521888
H	4.248767	-3.209765	1.600653
H	4.942645	-1.619946	1.976095
H	5.719524	0.374511	-0.826018
H	4.622450	1.376152	0.135923
H	5.539131	0.063599	0.917431
H	1.030438	2.207340	-2.229656
H	0.573843	4.303040	-0.982080
H	0.868294	2.146444	2.734665
H	1.310402	0.042669	1.471765
C	-3.051990	-0.496185	-0.026951
N	-4.183629	-1.202358	0.084834
N	-3.341990	0.807868	0.024030
C	-5.234002	-0.314917	0.210544
C	-4.264791	-2.663485	0.083261
C	-4.707781	0.939623	0.170461
C	-2.361516	1.892804	-0.094287
H	-6.258351	-0.653563	0.319511
H	-3.735270	-3.063145	0.956206
H	-5.321855	-2.941622	0.129638
H	-3.817984	-3.053316	-0.838711
H	-5.183709	1.911771	0.235137
H	-2.093697	2.034339	-1.148403
H	-2.817646	2.803536	0.306142
H	-1.463737	1.638834	0.483656
Cl	-1.500082	-1.130785	-0.202608

Br_cat

R1_cat

C	3.405954	0.383419	-0.966461
C	2.764428	-0.569040	-0.169655
C	2.393956	-0.255663	1.145420
C	2.665014	1.009201	1.657185
C	3.285129	1.969383	0.849040
C	3.651213	1.660716	-0.461864
C	2.470742	-1.916987	-0.716809
O	1.659113	-2.672369	-0.227693
H	3.503956	2.958675	1.253516
H	3.035126	-2.202208	-1.633456
H	3.714503	0.122691	-1.981668
H	4.150545	2.406873	-1.080378
H	2.410481	1.249058	2.690262
H	1.922941	-1.027200	1.757062
C	-1.923236	0.205438	-0.089392
N	-1.533972	1.441058	-0.428939
N	-3.239510	0.217014	0.155282
C	-2.639009	2.266142	-0.401247
C	-0.164752	1.826713	-0.784717
C	-3.704674	1.501973	-0.035175
C	-4.032820	-0.945724	0.558207
H	-2.571834	3.321159	-0.642912
H	0.524325	1.527636	0.016165
H	0.120866	1.338260	-1.724530
H	-0.145785	2.913876	-0.908033
H	-4.748577	1.759415	0.106484
H	-3.955520	-1.724873	-0.209305
H	-3.665424	-1.325789	1.518856
H	-5.073740	-0.624094	0.658393
Br	-0.803202	-1.249556	0.011700

R1_cat_R2

C	1.550123	1.886338	-1.322167
C	1.355666	0.636927	-0.727714
C	1.149740	0.536902	0.655252
C	1.142658	1.689320	1.436890
C	1.314161	2.942312	0.836022
C	1.512313	3.042885	-0.542111
C	1.376055	-0.592098	-1.560523
C	4.479012	-1.099610	-1.482917
C	4.384992	-0.504541	-0.283543
C	4.027683	-1.268283	0.946552

C	3.242079	-2.354641	0.903468
O	0.899259	-1.647526	-1.197427
C	4.584458	-0.757662	2.250831
C	4.681575	0.964057	-0.117379
H	1.313659	3.843769	1.450614
H	1.835595	-0.487297	-2.568007
H	4.340548	-2.175664	-1.599861
H	4.742660	-0.523776	-2.372949
H	3.021672	-2.914392	1.814831
H	2.802672	-2.714654	-0.027669
H	4.210827	0.254462	2.473955
H	4.307010	-1.419182	3.080915
H	5.682739	-0.692220	2.211955
H	4.850693	1.444074	-1.090160
H	3.856708	1.484941	0.395478
H	5.584148	1.111165	0.495612
H	1.737828	1.951829	-2.396742
H	1.662840	4.019451	-1.003166
H	1.016261	1.616637	2.518069
H	1.041545	-0.454064	1.098630
C	-3.251738	-0.148943	0.038401
N	-3.302112	1.188638	-0.007113
N	-4.449568	-0.615075	0.413745
C	-4.573825	1.586965	0.348864
C	-2.188968	2.061976	-0.392564
C	-5.290923	0.460273	0.613068
C	-4.788615	-2.029220	0.581202
H	-4.857112	2.633105	0.383913
H	-1.306885	1.829155	0.218576
H	-1.956469	1.912548	-1.454169
H	-2.501486	3.096422	-0.220143
H	-6.321321	0.330444	0.925208
H	-4.627568	-2.558433	-0.365408
H	-4.162178	-2.466465	1.367737
H	-5.842857	-2.090820	0.867183
Br	-1.760072	-1.148100	-0.358891

TS

C	1.476428	2.045594	-1.216988
C	1.438305	0.825971	-0.535483
C	1.278959	0.822927	0.855361
C	1.183746	2.025218	1.557049
C	1.226537	3.242037	0.871004
C	1.367658	3.249166	-0.518403
C	1.566862	-0.487730	-1.261034
C	3.455181	-0.675317	-1.656666
C	4.017803	-0.591972	-0.373569
C	3.683306	-1.559680	0.610070
C	2.879436	-2.609844	0.233163
O	1.173646	-1.547614	-0.654361
C	4.011636	-1.306192	2.060716
C	4.707956	0.671206	0.048679
H	1.157316	4.182968	1.418244
H	1.359059	-0.411803	-2.346836
H	3.299178	-1.652257	-2.115185
H	3.716253	0.113912	-2.366403
H	2.420227	-3.245167	0.993560
H	2.857000	-2.999032	-0.779499
H	3.672201	-0.313880	2.394564
H	3.551007	-2.066854	2.703555
H	5.099023	-1.348566	2.226043
H	4.975522	1.294810	-0.812118
H	4.051209	1.259372	0.714054
H	5.619975	0.441592	0.618706
H	1.600640	2.054159	-2.303480
H	1.405778	4.196675	-1.057774
H	1.077181	2.016029	2.643276
H	1.234414	-0.140458	1.367493
Br	-1.458643	-1.074438	-0.245535
C	-3.063246	-0.202202	0.006040
N	-4.258364	-0.756378	0.249246
N	-3.205716	1.129543	-0.025521
C	-5.190695	0.253532	0.375870
C	-4.509036	-2.192777	0.364983
C	-4.531993	1.432055	0.202368
C	-2.122922	2.084248	-0.285244
H	-6.235559	0.046126	0.578467
H	-3.955416	-2.595460	1.221549
H	-5.583294	-2.337033	0.514395
H	-4.190256	-2.694684	-0.556120
H	-4.890809	2.455057	0.222111
H	-1.799494	1.997886	-1.330001
H	-2.510093	3.089673	-0.093282
H	-1.274700	1.873594	0.379703

Product

C	1.179385	2.255965	-1.160854
C	1.407078	1.064912	-0.463832
C	1.356684	1.077944	0.934020
C	1.104241	2.266801	1.620743
C	0.889049	3.454103	0.918443
C	0.923822	3.445290	-0.477004
C	1.762581	-0.197230	-1.231209
C	3.268822	-0.247144	-1.536679
C	4.044337	-0.538149	-0.274129
C	3.590811	-1.571131	0.453456
C	2.367737	-2.265490	-0.093409
O	1.342012	-1.349251	-0.504719
C	4.155793	-2.098741	1.738978
C	5.191660	0.366413	0.058010
H	0.705271	4.384717	1.456789
H	1.208488	-0.192596	-2.186158
H	3.445871	-1.037180	-2.286077
H	3.571777	0.706104	-1.991003
H	1.897543	-2.900594	0.669740
H	2.633726	-2.912790	-0.949858
H	3.428653	-1.984166	2.559649
H	4.370597	-3.175694	1.653027
H	5.083767	-1.595113	2.031030
H	5.921019	0.379730	-0.766952
H	4.827555	1.399916	0.179481
H	5.716167	0.076532	0.975191
H	1.216017	2.256027	-2.253769
H	0.764084	4.369329	-1.034704
H	1.085868	2.268158	2.711659
H	1.524705	0.148094	1.478772
C	-3.015549	-0.313240	0.010332
N	-3.254314	1.003135	0.063539
N	-4.177574	-0.968000	0.135675
C	-4.610630	1.193351	0.224608
C	-2.233824	2.048911	-0.063919
C	-5.188480	-0.038373	0.271692
C	-4.327703	-2.423141	0.137274
H	-5.045207	2.184452	0.292696
H	-1.365971	1.795492	0.558936
H	-1.919913	2.132494	-1.111698
H	-2.673949	2.991954	0.274952
H	-6.225049	-0.333417	0.391492

H	-3.915880	-2.834072	-0.791920
H	-3.801441	-2.846929	1.000874
H	-5.395613	-2.651890	0.203166
Br	-1.341881	-1.056194	-0.194891

I_cat

R1_cat

C	-4.874353	-0.000314	1.351348
C	-3.996874	0.000096	0.261045
C	-4.489859	0.000256	-1.051808
C	-5.861033	0.000023	-1.266118
C	-6.736970	-0.000379	-0.174940
C	-6.248560	-0.000558	1.131891
C	-2.552646	0.000351	0.506802
O	-1.709870	0.000675	-0.377372
H	-7.813867	-0.000563	-0.348007
H	-2.244582	0.000197	1.576810
H	-4.477537	-0.000450	2.369153
H	-6.939256	-0.000878	1.974769
H	-6.257076	0.000140	-2.281597
H	-3.781950	0.000581	-1.881194
I	0.934758	0.000298	-0.146604
C	3.015719	-0.000121	0.023120
N	3.806091	1.083868	0.084687
N	3.805619	-1.084469	0.084437
C	5.120205	0.679673	0.187311
C	3.342967	2.470716	0.045540
C	5.119904	-0.680869	0.187160
C	3.341912	-2.471110	0.044959
H	5.936989	1.390131	0.250635
H	2.810352	2.654998	-0.895069
H	2.677950	2.661679	0.896232
H	4.220110	3.122035	0.108018
H	5.936390	-1.391684	0.250332
H	2.808985	-2.654867	-0.895576
H	4.218800	-3.122813	0.107004
H	2.677019	-2.662085	0.895747

R1_cat_R2

C	-3.998171	-1.715235	-1.241899
C	-3.136471	-1.440517	-0.174247
C	-3.647179	-1.107237	1.087862
C	-5.022946	-1.046397	1.272518
C	-5.883846	-1.328584	0.206094
C	-5.375994	-1.664637	-1.049639
C	-1.687836	-1.490477	-0.394235
C	-1.353442	1.515041	-1.444341
C	-2.456052	1.829887	-0.748903
C	-2.371759	2.330307	0.651711
C	-1.521444	1.787717	1.534082
O	-0.858171	-1.353201	0.492661
C	-3.309627	3.445966	1.032239
C	-3.832424	1.726101	-1.349965
H	-6.963212	-1.286167	0.357853
H	-1.364608	-1.669853	-1.443955
H	-0.361031	1.655864	-1.010927
H	-1.419357	1.145160	-2.470350
H	-1.461040	2.166418	2.556548
H	-0.901021	0.929704	1.269211
H	-4.356777	3.108986	0.984892
H	-3.109503	3.796543	2.052279
H	-3.212660	4.297104	0.340779
H	-3.797533	1.266493	-2.346258
H	-4.494682	1.125559	-0.705203
H	-4.288979	2.723174	-1.448067
H	-3.585886	-1.969595	-2.221282
H	-6.054201	-1.883824	-1.874129
H	-5.432871	-0.785242	2.248266
H	-2.951955	-0.894577	1.900332
I	1.680650	-0.694980	0.175772
C	3.633040	0.006476	-0.052881
N	4.762106	-0.715142	0.029721
N	3.979477	1.280179	-0.301121
C	5.844422	0.115264	-0.171119
C	4.826944	-2.151299	0.298718
C	5.353307	1.366959	-0.379337
C	3.043702	2.391970	-0.472571
H	6.864000	-0.253215	-0.147226
H	4.381270	-2.363381	1.277961
H	4.287352	-2.697445	-0.484161
H	5.880091	-2.448581	0.298740
H	5.859998	2.305681	-0.574014
H	2.379070	2.452039	0.397870
H	3.626365	3.314444	-0.557306
H	2.451658	2.241169	-1.383754

TS

C	3.515506	1.633883	-1.469037
C	2.792357	1.042382	-0.429823
C	3.205510	1.223749	0.891583
C	4.348912	1.973638	1.169074
C	5.077646	2.551296	0.129262
C	4.654799	2.386161	-1.190933
C	1.598520	0.171737	-0.714039
C	2.239072	-1.370660	-1.503645
C	3.201261	-1.813433	-0.566048
C	2.799119	-2.194797	0.743433
C	1.456141	-2.258811	1.009340
O	0.859563	-0.155695	0.309645
C	3.823350	-2.298557	1.845148
C	4.652037	-1.566636	-0.831758
H	5.969503	3.139628	0.346730
H	1.053146	0.475096	-1.630244
H	1.274177	-1.880394	-1.546188
H	2.624951	-1.096528	-2.488919
H	1.102531	-2.323378	2.040824
H	0.715369	-2.440898	0.236466
H	4.436337	-1.387526	1.912909
H	3.337569	-2.464788	2.814343
H	4.505932	-3.142529	1.664929
H	4.847568	-1.368289	-1.891315
H	4.989964	-0.691995	-0.246043
H	5.255757	-2.425433	-0.504046
H	3.187927	1.504934	-2.504376
H	5.214398	2.847707	-2.005164
H	4.668510	2.115659	2.202409
H	2.611555	0.774799	1.688761
I	-1.574799	-0.009682	0.125700
C	-3.689767	0.169822	-0.014254
N	-4.416636	1.240811	0.347834
N	-4.549828	-0.753876	-0.476744
C	-5.752651	0.992435	0.111933
C	-3.869294	2.475458	0.907574
C	-5.836712	-0.261880	-0.406741
C	-4.173219	-2.074039	-0.975757
H	-6.522658	1.723471	0.331877
H	-3.348577	2.256128	1.847287
H	-3.170699	2.927090	0.193248

H	-4.701134	3.161243	1.096140
H	-6.694311	-0.841795	-0.729106
H	-3.661556	-2.635537	-0.185027
H	-5.086508	-2.600495	-1.270211
H	-3.512186	-1.966483	-1.844015

Product

C	1.351100	2.303298	-1.380132
C	1.671366	1.188005	-0.600845
C	1.645308	1.303583	0.793481
C	1.318117	2.519119	1.393818
C	1.006043	3.631239	0.609082
C	1.022859	3.520494	-0.781531
C	2.060332	-0.113168	-1.278690
C	3.583825	-0.248889	-1.426776
C	4.225386	-0.583188	-0.100750
C	3.654146	-1.588868	0.581032
C	2.438770	-2.206101	-0.064831
O	1.511558	-1.218307	-0.548444
C	4.079093	-2.151369	1.905277
C	5.397561	0.245698	0.328059
H	0.762335	4.584414	1.080321
H	1.605481	-0.130777	-2.283513
H	3.793557	-1.047198	-2.158818
H	3.977954	0.686696	-1.846426
H	1.867760	-2.808287	0.655233
H	2.724107	-2.865658	-0.904737
H	3.296668	-1.991853	2.665448
H	4.232999	-3.239522	1.831186
H	5.010005	-1.706802	2.273412
H	6.190216	0.210923	-0.435598
H	5.092764	1.300661	0.423761
H	5.826612	-0.077225	1.282940
H	1.362847	2.220610	-2.469931
H	0.786830	4.385125	-1.403414
H	1.315736	2.601630	2.481928
H	1.880101	0.431727	1.405805
I	-1.125455	-0.885127	-0.182444
C	-3.067421	-0.198423	0.098966
N	-3.372307	1.081375	0.368338
N	-4.214281	-0.892790	0.043293
C	-4.740230	1.200538	0.487278
C	-2.396472	2.164543	0.517751
C	-5.268906	-0.037160	0.283596
C	-4.317899	-2.325403	-0.229858
H	-5.218069	2.149241	0.705021
H	-1.740954	1.958500	1.373086
H	-1.786447	2.246145	-0.391152
H	-2.948336	3.095138	0.683025
H	-6.297561	-0.380222	0.288335
H	-3.787818	-2.888200	0.547805
H	-5.378227	-2.595985	-0.225006
H	-3.883683	-2.544692	-1.212580

S2_rigid_cat

R1_cat

C	2.053502	0.923247	0.132613
C	0.191810	-0.200220	1.074347
C	-0.390325	1.030426	0.888779
C	-2.182125	-0.134108	1.700847
C	-1.756339	1.089090	1.234900
H	-2.405877	1.955406	1.135702
C	1.577698	-0.265694	0.636844
C	3.392394	0.875892	-0.301936
H	3.957217	1.699671	-0.731313
C	3.909539	-0.387727	-0.108498
C	-3.506654	-0.489850	2.086763
C	5.227744	-0.829940	-0.421051
N	-4.585345	-0.793510	2.382885
N	6.298898	-1.195466	-0.669621
S	-0.915339	-1.336430	1.724552
S	2.760618	-1.499161	0.586110
S	0.782414	2.198254	0.172262
O	1.121466	3.249098	1.134583
O	0.379684	2.555007	-1.195987
C	-3.213274	-1.361291	-1.370102
C	-1.855943	-1.148014	-1.632660
C	-1.397432	0.121643	-1.999025
C	-2.297657	1.177068	-2.107267
C	-3.652308	0.963388	-1.836574
C	-4.112248	-0.301431	-1.462465
C	-0.889096	-2.259124	-1.468908
O	0.312843	-2.117397	-1.475736
H	-4.358313	1.791631	-1.916714
H	-1.346478	-3.265598	-1.309687
H	-3.560525	-2.355765	-1.077744
H	-5.168088	-0.458859	-1.241016
H	-1.931200	2.167115	-2.379541
H	-0.332348	0.272831	-2.181843

R1_cat_R2

C	-2.116809	1.187499	0.699808
C	-0.239167	1.832578	-0.595759
C	0.192916	2.285900	0.630185
C	2.017964	2.796310	-0.644150
C	1.491493	2.830740	0.629303
H	2.022087	3.226321	1.491954
C	-1.553765	1.205765	-0.557335
C	-3.424688	0.668275	0.748469
H	-4.043946	0.570358	1.636905
C	-3.826313	0.284432	-0.514413
C	3.303956	3.255221	-1.051994
C	-5.071827	-0.309805	-0.870816
N	4.349178	3.623194	-1.391268
N	-6.083003	-0.791480	-1.168138
S	0.940451	2.078023	-1.810323
S	-2.607727	0.545881	-1.731413
S	-0.984024	1.862275	1.922197
O	-1.545546	3.045513	2.576827
O	-0.420237	0.766858	2.730879
C	3.412556	-1.548596	-0.826785
C	2.054360	-1.224328	-0.765279
C	1.511343	-0.691470	0.409623
C	2.327854	-0.464273	1.513152
C	3.691133	-0.766517	1.439409
C	4.233617	-1.310851	0.273860
C	1.193737	-1.434522	-1.951187
C	0.283750	-4.506330	-1.159567
C	0.438687	-3.966546	0.059305
C	-0.618582	-3.123961	0.693172
C	-1.548151	-2.483780	-0.031449
O	0.102965	-0.922470	-2.091953
C	-0.585515	-2.988820	2.193972
C	1.694028	-4.216503	0.856465
H	4.334832	-0.585827	2.301581
H	1.624715	-2.086645	-2.745866
H	-0.641620	-4.393475	-1.726142
H	1.078194	-5.106681	-1.607395

H	-2.320553	-1.893711	0.466776
H	-1.562929	-2.520108	-1.121191
H	-1.393081	-2.334476	2.545713
H	-0.684387	-3.969600	2.685188
H	0.368211	-2.551483	2.528677
H	2.171136	-3.273078	1.165152
H	1.465815	-4.777916	1.775761
H	2.417503	-4.798860	0.271321
H	3.825876	-1.979061	-1.742036
H	5.296024	-1.550850	0.225638
H	1.886865	-0.055518	2.424238
H	0.439938	-0.501967	0.458253

TS

C	2.180590	-1.104746	0.688604
C	0.320782	-1.663827	-0.670281
C	-0.149983	-2.154125	0.525389
C	-1.976423	-2.514257	-0.799374
C	-1.472431	-2.639154	0.476460
H	-2.040605	-3.040216	1.312079
C	1.653806	-1.087090	-0.582489
C	3.493297	-0.599442	0.783080
H	4.089125	-0.527310	1.689881
C	3.938049	-0.199773	-0.460211
C	-3.285715	-2.864181	-1.240154
C	5.202240	0.379468	-0.771576
N	-4.349671	-3.141899	-1.606316
N	6.226861	0.853687	-1.033321
S	-0.844468	-1.796363	-1.914389
S	2.753057	-0.435414	-1.719027
S	1.018670	-1.832983	1.855825
O	1.557678	-3.068451	2.428969
O	0.472846	-0.789365	2.740893
C	-3.419770	1.117344	-0.778241
C	-2.046653	1.130349	-0.514303
C	-1.581184	0.670342	0.720149
C	-2.475704	0.228231	1.693964
C	-3.845352	0.216967	1.426303
C	-4.314641	0.655128	0.185215
C	-1.055014	1.617137	-1.525881
C	-1.032010	3.609871	-1.310844
C	-0.718414	3.711631	0.049982
C	0.482630	3.142520	0.538576
C	1.347189	2.585317	-0.384098
O	0.159435	1.229688	-1.420449
C	0.679850	2.910932	2.017752
C	-1.788721	4.119357	1.023415
H	-4.549860	-0.139714	2.178892
H	-1.463793	1.757495	-2.544157
H	-0.243433	3.668198	-2.060983
H	-2.000054	3.997526	-1.638626
H	2.157980	1.940498	-0.033787
H	1.473963	2.995491	-1.382213
H	1.480956	2.178871	2.189895
H	0.963311	3.845307	2.527831
H	-0.228252	2.528771	2.505894
H	-2.204218	3.228126	1.525850
H	-1.376281	4.772654	1.805824
H	-2.609965	4.644512	0.521001
H	-3.788720	1.463429	-1.747471
H	-5.383055	0.631789	-0.032745
H	-2.083844	-0.127368	2.649394
H	-0.507902	0.652628	0.908432

Product

C	2.160392	-1.151619	0.744037
C	0.277457	-1.679126	-0.593849
C	-0.261788	-1.979963	0.632791
C	-2.068272	-2.386578	-0.708059
C	-1.611490	-2.389575	0.590100
H	-2.229426	-2.653961	1.444234
C	1.656438	-1.223519	-0.532793
C	3.496084	-0.705043	0.818663
H	4.079410	-0.577473	1.727495
C	3.982898	-0.445867	-0.445099
C	-3.382136	-2.709716	-1.155420
C	5.284733	0.023995	-0.783510
N	-4.448293	-2.966989	-1.530323
N	6.340112	0.409519	-1.067548
S	-0.855012	-1.884913	-1.859486
S	2.808699	-0.743088	-1.702889
S	0.917664	-1.644312	1.952636
O	1.309878	-2.875056	2.643372
O	0.493279	-0.469242	2.730980
C	-3.393757	0.809679	-0.839362
C	-2.085664	1.095763	-0.434469
C	-1.724833	0.870477	0.896321
C	-2.663205	0.397463	1.813861
C	-3.969313	0.126594	1.405754
C	-4.331222	0.325270	0.072149
C	-1.093237	1.680783	-1.423344
C	-1.148606	3.218565	-1.413558
C	-0.601366	3.730766	-0.102559
C	0.562402	3.188516	0.286028
C	1.160195	2.168301	-0.651245
O	0.221238	1.211923	-1.148069
C	1.301325	3.429176	1.569066
C	-1.447586	4.693356	0.673813
H	-4.703424	-0.248795	2.120251
H	-1.353092	1.321607	-2.433637
H	-0.550549	3.594673	-2.261424
H	-2.186799	3.541433	-1.573099
H	1.931354	1.579071	-0.130450
H	1.650440	2.672720	-1.506993
H	1.305819	2.507411	2.176354
H	2.353656	3.688063	1.370170
H	0.862859	4.234329	2.169507
H	-2.401893	4.210516	0.943289
H	-0.966427	5.041008	1.595183
H	-1.694825	5.573220	0.059027
H	-3.681075	0.958433	-1.884107
H	-5.341985	0.089469	-0.263080
H	-2.353150	0.224026	2.846064
H	-0.697108	1.036381	1.215949

S2_flex_cat

R1_cat

C	3.332096	-1.763716	-0.287926
C	1.973489	-1.857927	0.021212
C	1.483508	-1.344271	1.229065
C	2.357210	-0.734731	2.119995
C	3.714332	-0.620582	1.799638
C	4.203532	-1.133088	0.599216
C	1.034881	-2.451146	-0.958616
O	-0.169311	-2.364002	-0.875928
H	4.393846	-0.123606	2.492948
H	1.511833	-2.980050	-1.817485
H	3.702282	-2.164299	-1.234651

H	5.259327	-1.030316	0.348767
H	1.986219	-0.330253	3.062550
H	0.416519	-1.433160	1.442438
S	-2.582552	-0.554116	-0.364230
C	-4.125324	0.042610	0.163326
C	-1.890476	0.946698	0.151952
C	-2.874785	1.751091	0.682564
H	-2.700385	2.757725	1.059767
C	-0.472116	1.265177	0.055622
C	0.299741	1.901506	1.003119
S	0.494145	0.928685	-1.335995
H	-0.070643	2.210576	1.979554
C	1.847576	1.641281	-0.513351
N	-4.133597	1.236450	0.693024
N	1.604146	2.112739	0.678965
C	3.140717	1.660381	-1.133893
N	4.171340	1.647679	-1.661486
C	-5.298841	-0.770461	0.018154
N	-6.225209	-1.451819	-0.113835

R1_cat_R2

C	3.480069	-2.401150	-1.282619
C	2.401430	-1.541808	-1.061696
C	2.572153	-0.158190	-1.181920
C	3.812060	0.365066	-1.531859
C	4.885368	-0.500412	-1.767901
C	4.722291	-1.880410	-1.641547
C	1.077948	-2.102499	-0.699872
C	1.828259	-2.343309	2.475663
C	2.472350	-1.201380	2.189006
C	1.766064	0.111746	2.118594
C	0.443706	0.202492	1.913567
O	0.059305	-1.450271	-0.631761
C	2.601338	1.357808	2.273361
C	3.961403	-1.196964	1.956583
H	5.858240	-0.093012	-2.046309
H	1.069202	-3.199835	-0.500755
H	0.763751	-2.361560	2.713331
H	2.367253	-3.292301	2.508814
H	-0.043694	1.179241	1.919488
H	-0.181243	-0.674752	1.742347
H	3.348427	1.435116	1.466904
H	1.969606	2.255840	2.239655
H	3.152644	1.356495	3.226584
H	4.362896	-2.218426	1.975556
H	4.215903	-0.738535	0.988785
H	4.474973	-0.612180	2.735500
H	3.342419	-3.479811	-1.174491
H	5.564767	-2.549042	-1.820726
H	3.939427	1.445512	-1.616413
H	1.722788	0.489699	-0.970042
S	-3.086072	-0.965048	-0.344886
C	-4.805932	-1.101704	-0.143499
C	-3.249096	0.739047	-0.081929
C	-4.572119	1.048537	0.140125
H	-4.942015	2.058009	0.313263
C	-2.113579	1.649817	-0.065115
C	-1.897857	2.715299	0.785612
S	-0.782483	1.500963	-1.147813
H	-2.580797	3.008238	1.581559
C	-0.041167	2.847406	-0.352781
N	-5.447379	0.007325	0.108654
N	-0.725745	3.384972	0.623778
C	1.284196	3.246009	-0.729605
N	2.367266	3.494450	-1.056779
C	-5.442959	-2.382712	-0.252894
N	-5.922381	-3.432079	-0.347288

TS

C	2.990741	-2.492874	-1.349519
C	2.173575	-1.499374	-0.803090
C	2.530362	-0.158104	-0.964309
C	3.692346	0.192210	-1.650081
C	4.505540	-0.805023	-2.189931
C	4.150662	-2.147335	-2.040142
C	0.943180	-1.845387	-0.019710
C	1.662398	-2.422463	1.754860
C	2.376134	-1.273439	2.120161
C	1.697117	-0.038315	2.246021
C	0.326127	-0.046696	2.072890
O	0.081827	-0.926864	0.211894
C	2.463123	1.262077	2.290404
C	3.877105	-1.280400	2.049043
H	5.413688	-0.537649	-2.731388
H	0.566741	-2.872025	-0.184498
H	0.646561	-2.574779	2.119607
H	2.230012	-3.343558	1.599468
H	-0.202525	0.899969	1.939590
H	-0.290924	-0.885261	2.384139
H	3.212662	1.333868	1.486908
H	1.778943	2.117845	2.201442
H	3.000333	1.367998	3.246154
H	4.276128	-2.301840	2.042445
H	4.215340	-0.770856	1.129514
H	4.309868	-0.735898	2.900952
H	2.716223	-3.544570	-1.231293
H	4.781527	-2.928543	-2.466518
H	3.955143	1.245928	-1.761401
H	1.887641	0.602922	-0.523721
S	-2.921297	-0.935088	-0.300655
C	-4.654081	-1.032959	-0.241884
C	-3.074683	0.785716	-0.161257
C	-4.405030	1.125307	-0.068237
H	-4.766194	2.149527	0.012350
C	-1.930572	1.686832	-0.108511
C	-1.701284	2.700444	0.800333
S	-0.607230	1.585625	-1.202923
H	-2.382044	2.956415	1.611037
C	0.154902	2.870901	-0.334038
N	-5.295723	0.096872	-0.108982
N	-0.519483	3.360008	0.677305
C	1.481304	3.282847	-0.693356
N	2.566094	3.545476	-1.003246
C	-5.303726	-2.310500	-0.320695
N	-5.790709	-3.358635	-0.385994

Product

C	0.119082	-2.767041	0.707085
C	1.086007	-1.780320	0.905718
C	2.210970	-1.758626	0.073531
C	2.358153	-2.698573	-0.944607
C	1.378474	-3.674896	-1.143110
C	0.260029	-3.709981	-0.313265
C	0.847428	-0.696611	1.937398
C	2.121617	-0.267669	2.694301
C	2.880082	0.851723	2.015302
C	2.139160	1.861101	1.532036
C	0.644529	1.683824	1.666221

O	0.232048	0.391032	1.241051
C	2.624579	3.124113	0.884721
C	4.377591	0.753212	2.001798
H	1.491521	-4.408265	-1.942460
H	0.118721	-1.082592	2.673305
H	1.825247	0.075423	3.700254
H	2.757950	-1.151856	2.842316
H	0.101208	2.392556	1.026353
H	0.319798	1.861095	2.711600
H	2.285385	3.173720	-0.162620
H	2.204781	4.002005	1.402788
H	3.716436	3.217821	0.900633
H	4.764885	0.691138	3.031734
H	4.700799	-0.168002	1.489938
H	4.857470	1.603932	1.505166
H	-0.759700	-2.794132	1.355989
H	-0.505726	-4.473264	-0.457842
H	3.235062	-2.657091	-1.592420
H	2.965116	-0.978743	0.198362
S	-2.720405	-0.149610	0.503052
C	-4.411805	0.186407	0.301227
C	-2.421566	0.785994	-0.923638
C	-3.609416	1.281092	-1.406927
H	-3.691692	1.894185	-2.302926
C	-1.074609	0.996433	-1.442969
C	-0.414000	2.193099	-1.602463
S	-0.024506	-0.307155	-1.846779
H	-0.856729	3.166601	-1.395624
C	1.220266	0.854196	-2.150985
N	-4.732103	0.945363	-0.711882
N	0.883952	2.110142	-2.000901
C	2.538824	0.422190	-2.514869
N	3.594437	0.029140	-2.784013
C	-5.367200	-0.344587	1.231675
N	-6.105929	-0.792505	2.002168

Se_cat

R1_cat

C	-5.874396	-1.119534	-0.601273
C	-4.482148	-0.998858	-0.596065
C	-3.706523	-1.789144	0.261259
C	-4.331122	-2.697065	1.107490
C	-5.723924	-2.818473	1.099422
C	-6.497700	-2.031567	0.246944
C	-3.840499	-0.027447	-1.508314
O	-2.645880	0.141836	-1.611777
H	-6.209239	-3.532661	1.766172
H	-4.548096	0.570200	-2.130521
H	-6.467641	-0.495458	-1.273926
H	-7.583471	-2.129418	0.245536
H	-3.735127	-3.314146	1.780381
H	-2.622486	-1.669307	0.256890
C	1.789558	-1.090862	-0.214749
C	2.048516	-0.978577	1.150424
C	2.849741	-1.396248	-1.063859
C	3.326241	-1.162244	1.661415
C	4.133402	-1.597639	-0.568086
C	4.368386	-1.477911	0.795949
C	-0.087021	0.994174	-0.346502
C	0.800152	1.934944	-0.863875
C	-1.050656	1.421604	0.561796
C	0.734991	3.270605	-0.487813
C	-1.140387	2.756838	0.935655
C	-0.243534	3.679577	0.410651
F	5.136175	-1.892812	-1.387759
F	2.660275	-1.502928	-2.373243
F	5.590481	-1.662340	1.275187
F	3.560626	-1.046846	2.964245
F	1.068356	-0.674181	1.996684
F	1.743199	1.567836	-1.726645
F	1.591005	4.157414	-0.984210
F	-0.319435	4.953596	0.770697
F	-2.068659	3.156000	1.799072
F	-1.912097	0.564284	1.098041
Se	0.013097	-0.834621	-0.897698

R1_cat_R2

C	-3.966048	-2.937373	-1.183823
C	-3.155482	-1.890672	-0.738006
C	-2.779973	-1.806584	0.608363
C	-3.221281	-2.776575	1.502818
C	-4.022816	-3.830936	1.053854
C	-4.394784	-3.914901	-0.288372
C	-2.698629	-0.858833	-1.695214
C	-5.384554	0.899826	-1.607587
C	-5.425889	0.469114	-0.337790
C	-4.673314	1.152954	0.751893
C	-3.525820	1.807177	0.523967
O	-1.866668	-0.015877	-1.434496
C	-5.249932	1.058994	2.141841
C	-6.262778	-0.721068	0.055180
H	-4.362014	-4.591955	1.758190
H	-3.165626	-0.903970	-2.706127
H	-4.829902	1.796504	-1.888518
H	-5.931505	0.374281	-2.393556
H	-3.007395	2.313544	1.339865
H	-3.067142	1.837362	-0.466044
H	-5.277691	0.013578	2.487952
H	-4.648075	1.639644	2.852054
H	-6.284235	1.436301	2.170403
H	-6.704520	-1.200160	-0.828468
H	-5.659931	-1.468683	0.595141
H	-7.081200	-0.417378	0.726579
H	-4.259308	-2.982545	-2.235611
H	-5.021707	-4.737900	-0.632475
H	-2.938808	-2.716611	2.554442
H	-2.157593	-0.971286	0.931695
C	2.610432	-1.053695	0.006081
C	3.018760	-0.618148	1.266045
C	3.572335	-1.576594	-0.853936
C	4.347374	-0.693178	1.661449
C	4.905243	-1.673538	-0.468824
C	5.289949	-1.228322	0.789607
C	0.684970	0.965916	-0.356326
C	1.389892	1.786106	-1.232275
C	-0.103811	1.561951	0.622231
C	1.308586	3.170628	-1.144996
C	-0.201553	2.943847	0.718603
C	0.503271	3.747402	-0.170245
F	5.812945	-2.178391	-1.296976
F	3.238974	-2.000036	-2.067393
F	6.559651	-1.311867	1.161390
F	4.724044	-0.268361	2.862751
F	2.135345	-0.102832	2.116864
F	2.170891	1.258100	-2.168852
F	1.987306	3.944071	-1.985830
F	0.415735	5.067123	-0.079965
F	-0.956125	3.506633	1.659690

F	-0.778898	0.824101	1.497579
Se	0.769303	-0.936517	-0.524415

TS

C	-3.580191	-3.020458	-1.038856
C	-3.057158	-1.826954	-0.532596
C	-2.779964	-1.720657	0.833109
C	-3.046792	-2.791590	1.686374
C	-3.582090	-3.976049	1.180028
C	-3.842752	-4.090827	-0.187127
C	-2.819920	-0.640034	-1.419597
C	-4.641580	0.017522	-1.834003
C	-5.124749	0.237784	-0.536590
C	-4.459998	1.156796	0.308124
C	-3.402887	1.865905	-0.226670
O	-2.154822	0.350147	-0.951809
C	-4.714370	1.154606	1.795605
C	-6.108747	-0.728238	0.060126
H	-3.787770	-4.814164	1.847093
H	-2.682509	-0.886153	-2.489319
H	-4.229434	0.844772	-2.411795
H	-5.141117	-0.746378	-2.435407
H	-2.745033	2.421536	0.443677
H	-3.374789	2.167849	-1.269500
H	-4.695896	0.137041	2.214242
H	-3.955256	1.753934	2.315500
H	-5.700249	1.587124	2.028492
H	-6.622064	-1.311657	-0.713716
H	-5.586051	-1.432037	0.732457
H	-6.860438	-0.197820	0.662723
H	-3.787804	-3.108585	-2.108792
H	-4.250408	-5.019467	-0.588951
H	-2.827328	-2.703893	2.751619
H	-2.345805	-0.791203	1.204435
C	2.370546	-1.060940	0.008498
C	2.905077	-0.675180	1.236740
C	3.222004	-1.645481	-0.924918
C	4.249173	-0.860815	1.531077
C	4.567674	-1.852819	-0.642124
C	5.078945	-1.457048	0.587464
C	0.591277	1.100717	-0.227111
C	1.396845	1.851681	-1.078404
C	-0.190330	1.774721	0.704255
C	1.421209	3.239995	-1.014034
C	-0.199488	3.161689	0.762072
C	0.608984	3.896148	-0.096989
F	5.368556	-2.415164	-1.541069
F	2.768233	-2.020824	-2.115059
F	6.362326	-1.645535	0.862602
F	4.746714	-0.483028	2.704078
F	2.132622	-0.100683	2.154891
F	2.169752	1.250406	-1.978265
F	2.197584	3.942491	-1.832712
F	0.605258	5.221372	-0.041108
F	-0.997085	3.789084	1.626089
F	-0.967404	1.116262	1.559063
Se	0.505506	-0.801598	-0.375341

Product

C	-2.950258	-3.300110	-0.799447
C	-2.857450	-1.999727	-0.293099
C	-2.804537	-1.811136	1.089464
C	-2.860825	-2.906774	1.951115
C	-2.965897	-4.199291	1.441003
C	-3.006888	-4.394703	0.060036
C	-2.840627	-0.822758	-1.253258
C	-4.243865	-0.565392	-1.825344
C	-5.142142	-0.053405	-0.725950
C	-4.630646	0.942492	0.012779
C	-3.246122	1.404737	-0.372364
O	-2.325553	0.336539	-0.610663
C	-5.267451	1.628169	1.185001
C	-6.449071	-0.755174	-0.513692
H	-3.006624	-5.054627	2.116516
H	-2.150281	-1.055672	-2.081735
H	-4.160203	0.175220	-2.639159
H	-4.630192	-1.494947	-2.265447
H	-2.807372	2.002165	0.439283
H	-3.291958	2.046075	-1.274032
H	-4.688042	1.433578	2.102403
H	-5.273045	2.720292	1.039147
H	-6.300789	1.305515	1.355953
H	-7.046729	-0.747415	-1.438998
H	-6.261639	-1.812239	-0.261271
H	-7.050058	-0.311952	0.288638
H	-2.971498	-3.458003	-1.881219
H	-3.076071	-5.403758	-0.348569
H	-2.815528	-2.747985	3.029423
H	-2.694266	-0.800109	1.483010
C	2.346654	-0.973215	0.073526
C	3.022580	-0.506470	1.199709
C	3.082102	-1.619142	-0.916319
C	4.394110	-0.672598	1.339160
C	4.453433	-1.807695	-0.786281
C	5.107289	-1.330872	0.343009
C	0.515787	1.163461	-0.092673
C	1.261331	1.867866	-1.033794
C	-0.235886	1.886081	0.827109
C	1.260701	3.257471	-1.061312
C	-0.275673	3.273492	0.791687
C	0.480083	3.960400	-0.151067
F	5.143460	-2.428787	-1.736651
F	2.489472	-2.071187	-2.015161
F	6.415709	-1.501254	0.471094
F	5.027686	-0.217408	2.414723
F	2.363009	0.127739	2.164479
F	1.995614	1.220306	-1.934684
F	1.982251	3.916478	-1.961795
F	0.452641	5.285983	-0.185623
F	-1.044402	3.943475	1.646131
F	-0.968736	1.265386	1.747471
Se	0.448176	-0.745566	-0.094191

Te_cat

R1_cat

C	-5.963838	-1.087062	-0.414928
C	-4.566107	-1.068031	-0.410653
C	-3.852512	-1.741831	0.588610
C	-4.543511	-2.432080	1.576480
C	-5.941507	-2.452698	1.568731
C	-6.653937	-1.781627	0.575371
C	-3.858611	-0.329358	-1.475909
O	-2.653972	-0.271117	-1.603342
H	-6.479239	-2.996167	2.346926
H	-4.520000	0.199578	-2.201038
H	-6.508468	-0.555255	-1.198715
H	-7.743882	-1.799687	0.575139
H	-3.995589	-2.955971	2.360017

H	-2.762605	-1.703677	0.583068
Te	-0.000596	-1.065173	-0.765770
C	1.997404	-1.066247	-0.014623
C	2.307318	-0.520775	1.228216
C	3.037801	-1.613818	-0.757171
C	3.608982	-0.502732	1.713399
C	4.346648	-1.620132	-0.288820
C	4.629597	-1.058613	0.950170
C	-0.190635	1.013249	-0.400241
C	0.706030	1.917183	-0.961711
C	-1.206977	1.517091	0.403972
C	0.601535	3.283736	-0.730177
C	-1.338754	2.880183	0.637780
C	-0.428364	3.763416	0.069801
F	5.325641	-2.151368	-1.014005
F	2.801283	-2.157665	-1.950826
F	5.874863	-1.054501	1.407112
F	3.886067	0.030339	2.899316
F	1.352262	0.013476	1.990211
F	1.701796	1.491904	-1.736618
F	1.469569	4.132256	-1.271503
F	-0.542748	5.066003	0.292719
F	-2.319627	3.345045	1.405824
F	-2.090310	0.703396	0.981283

R1_cat_R2

C	-4.213106	-2.997846	-0.889159
C	-3.337434	-1.957240	-0.568771
C	-2.914824	-1.764138	0.752498
C	-3.374419	-2.620470	1.748075
C	-4.238165	-3.671482	1.424455
C	-4.657230	-3.863924	0.107408
C	-2.859758	-1.052193	-1.635155
C	-5.433755	0.841551	-1.654386
C	-5.483173	0.527521	-0.351063
C	-4.686563	1.267420	0.667933
C	-3.503236	1.826665	0.378832
O	-1.944967	-0.264898	-1.496965
C	-5.261020	1.342796	2.059711
C	-6.374186	-0.577869	0.153880
H	-4.590294	-4.343967	2.208029
H	-3.387520	-1.139176	-2.611879
H	-4.838493	1.681232	-2.016745
H	-6.015103	0.277663	-2.387408
H	-2.955923	2.378208	1.145308
H	-3.046617	1.734811	-0.608608
H	-5.344439	0.339405	2.506543
H	-4.624770	1.956040	2.709966
H	-6.272870	1.777448	2.049807
H	-6.844349	-1.116663	-0.679260
H	-5.804620	-1.298786	0.761912
H	-7.172608	-0.172054	0.794693
H	-4.543601	-3.126479	-1.922824
H	-5.333342	-4.683190	-0.138139
H	-3.057001	-2.474048	2.780964
H	-2.246238	-0.932549	0.979888
Te	0.725063	-1.046644	-0.617920
C	2.753284	-1.029587	0.045891
C	3.118992	-0.397594	1.231617
C	3.755439	-1.653404	-0.689403
C	4.437809	-0.367635	1.666662
C	5.080796	-1.648751	-0.269287
C	5.419551	-0.999666	0.911544
C	0.577174	1.057308	-0.418662
C	1.212087	1.905988	-1.319718
C	-0.162243	1.631091	0.609646
C	1.110995	3.288628	-1.210105
C	-0.275913	3.009194	0.737607
C	0.358267	3.838403	-0.180071
F	6.023148	-2.252595	-0.986189
F	3.465986	-2.283487	-1.827463
F	6.681108	-0.984727	1.321282
F	4.767800	0.247366	2.798080
F	2.201075	0.207576	1.986736
F	1.947515	1.416629	-2.313595
F	1.722832	4.085328	-2.080593
F	0.251607	5.154954	-0.066294
F	-0.987390	3.543732	1.728672
F	-0.780563	0.873913	1.514415

TS

C	-3.880141	-3.104449	-0.653367
C	-3.276704	-1.887406	-0.323757
C	-2.993280	-1.598452	1.014070
C	-3.330756	-2.511690	2.013087
C	-3.944999	-3.719568	1.681895
C	-4.214659	-4.017033	0.344548
C	-2.954521	-0.866455	-1.378661
C	-4.688307	-0.153500	-1.900629
C	-5.157496	0.325591	-0.667001
C	-4.434032	1.338315	0.007041
C	-3.344279	1.881295	-0.637108
O	-2.194630	0.120767	-1.060886
C	-4.680424	1.617383	1.469202
C	-6.203789	-0.449607	0.079617
H	-4.205665	-4.434511	2.463233
H	-2.829574	-1.292750	-2.392962
H	-4.236217	0.536048	-2.613747
H	-5.245866	-0.971833	-2.363415
H	-2.659317	2.515180	-0.073267
H	-3.278613	1.959996	-1.717687
H	-4.708882	0.690558	2.061844
H	-3.887823	2.260050	1.874408
H	-5.642701	2.131901	1.617666
H	-6.756228	-1.126481	-0.582916
H	-5.728930	-1.054405	0.872821
H	-6.915329	0.230439	0.570317
H	-4.092193	-3.336969	-1.700658
H	-4.684440	-4.965357	0.080095
H	-3.104633	-2.282533	3.055587
H	-2.500462	-0.654359	1.250567
Te	0.418411	-0.906077	-0.438728
C	2.498173	-1.096858	0.030804
C	3.044088	-0.508016	1.167974
C	3.353843	-1.819635	-0.792982
C	4.395492	-0.616667	1.471474
C	4.707479	-1.954371	-0.506250
C	5.227009	-1.346897	0.630003
C	0.531518	1.198764	-0.244851
C	1.419059	1.950918	-1.008135
C	-0.300753	1.878955	0.634444
C	1.473800	3.337002	-0.909963
C	-0.285536	3.264525	0.726060
C	0.607540	3.996344	-0.046049
F	5.507090	-2.651297	-1.308156
F	2.890685	-2.413545	-1.893306
F	6.517905	-1.465209	0.914140
F	4.898110	-0.040108	2.559476

F	2.275385	0.193342	2.002765
F	2.249674	1.357198	-1.862248
F	2.330076	4.036480	-1.647854
F	0.629163	5.320226	0.036458
F	-1.147038	3.891415	1.528178
F	-1.165874	1.228606	1.413906

Product

C	-2.797500	-3.669323	-0.349114
C	-2.956350	-2.298255	-0.137835
C	-2.933037	-1.800274	1.169522
C	-2.764889	-2.667339	2.246258
C	-2.610456	-4.037131	2.029738
C	-2.625624	-4.537435	0.729084
C	-3.179696	-1.369575	-1.311570
C	-4.670871	-0.991600	-1.455564
C	-5.052854	0.125703	-0.512266
C	-4.225904	1.183720	-0.512509
C	-3.014205	1.031765	-1.398686
O	-2.357846	-0.212253	-1.147531
C	-4.336289	2.437380	0.302605
C	-6.275028	-0.063994	0.334559
H	-2.469610	-4.712869	2.874182
H	-2.860426	-1.887015	-2.233359
H	-4.851182	-0.669498	-2.495313
H	-5.276225	-1.892810	-1.287939
H	-2.265949	1.810998	-1.202954
H	-3.285448	1.096688	-2.469720
H	-3.495257	2.512667	1.010608
H	-4.287334	3.326314	-0.347033
H	-5.269726	2.491518	0.874819
H	-7.153162	-0.275625	-0.296023
H	-6.137742	-0.936216	0.994658
H	-6.501942	0.807931	0.958944
H	-2.799660	-4.061407	-1.369187
H	-2.495550	-5.605715	0.551219
H	-2.740839	-2.269369	3.261551
H	-3.023961	-0.726274	1.330319
Te	0.366731	-0.945301	-0.448430
C	2.464395	-0.900725	-0.032552
C	2.969030	-0.270797	1.101785
C	3.375095	-1.509021	-0.889515
C	4.331477	-0.228370	1.370149
C	4.742245	-1.492481	-0.637846
C	5.218697	-0.846077	0.495840
C	0.244775	1.157525	-0.192592
C	1.014430	2.020575	-0.967207
C	-0.594051	1.721932	0.762933
C	0.954658	3.400489	-0.802536
C	-0.672529	3.097242	0.943715
C	0.102883	3.938624	0.154453
F	5.594438	-2.082305	-1.470708
F	2.953613	-2.136429	-1.987946
F	6.521398	-0.819598	0.746321
F	4.793041	0.386102	2.455247
F	2.147623	0.325390	1.967513
F	1.843092	1.544336	-1.892871
F	1.697719	4.206702	-1.553256
F	0.032336	5.252541	0.317393
F	-1.490087	3.614415	1.858817
F	-1.375799	0.958737	1.526016

P_cat

R1_cat

C	4.778520	1.564649	-0.215188
C	3.702442	1.384807	-1.086481
C	3.626943	0.243184	-1.895588
C	4.628319	-0.717230	-1.822945
C	5.693351	-0.548821	-0.930107
C	5.770615	0.588756	-0.127560
C	2.592562	2.366309	-1.093366
O	1.521017	2.161482	-1.617626
H	6.466524	-1.315003	-0.859017
H	2.792308	3.319855	-0.551292
H	4.820100	2.454228	0.417403
H	6.599366	0.711168	0.569777
H	4.577876	-1.610283	-2.447608
H	2.770716	0.130290	-2.563632
C	-1.910373	-1.165853	-0.536440
C	-1.936180	-2.136813	0.462536
C	-3.087181	-0.985460	-1.265519
C	-3.069382	-2.907373	0.704147
C	-4.234077	-1.731795	-1.039295
C	-4.219246	-2.704971	-0.047192
C	-1.081193	1.444380	-0.193454
C	-1.339539	2.595712	-0.932704
C	-1.354032	1.472542	1.172860
C	-1.828554	3.748856	-0.325820
C	-1.840322	2.607647	1.800292
C	-2.076633	3.750297	1.040083
F	-5.334395	-1.524219	-1.753147
F	-3.128663	-0.043452	-2.212026
F	-5.302242	-3.432276	0.186107
F	-3.060415	-3.829017	1.660768
F	-0.880511	-2.370279	1.240197
F	-1.145032	2.618916	-2.242778
F	-2.068011	4.841756	-1.042083
F	-2.543817	4.843281	1.625904
F	-2.080120	2.620511	3.107358
F	-1.123487	0.388813	1.915589
C	0.897941	-0.688842	-0.051649
C	1.674660	0.016675	0.860651
C	1.344538	-1.970070	-0.381255
C	2.834406	-0.520124	1.412947
C	2.492336	-2.531061	0.150488
C	3.250844	-1.790780	1.051003
F	0.627307	-2.697509	-1.244287
F	2.887827	-3.747515	-0.209643
F	4.377147	-2.290690	1.541088
F	3.564958	0.204007	2.255829
F	1.382078	1.270349	1.219543
P	-0.507549	-0.064262	-1.088204

R1_cat_R2

C	-4.163760	-0.065758	-1.320367
C	-3.250290	0.154637	-0.288704
C	-2.778357	-0.917887	0.480493
C	-3.224918	-2.207125	0.209057
C	-4.128793	-2.429016	-0.837114
C	-4.596840	-1.361592	-1.603055
C	-2.737642	1.524677	-0.042330
C	-4.988546	2.416194	2.030683
C	-5.002504	1.104323	2.310824
C	-4.046432	0.495983	3.280448
C	-2.822914	1.003786	3.488663
O	-1.759080	1.763330	0.629197

C	-4.508137	-0.744654	4.002293
C	-6.012284	0.183930	1.672493
H	-4.466160	-3.443097	-1.057361
H	-3.303211	2.342162	-0.546679
H	-4.300592	3.099474	2.531030
H	-5.687838	2.840139	1.306645
H	-2.150858	0.543009	4.215825
H	-2.445180	1.861964	2.932255
H	-5.452992	-0.565604	4.538979
H	-4.691256	-1.567609	3.292816
H	-3.754556	-1.077354	4.727214
H	-6.608665	0.718325	0.921324
H	-5.520502	-0.672757	1.183861
H	-6.700583	-0.225353	2.428402
H	-4.519971	0.777930	-1.916151
H	-5.294364	-1.539666	-2.421728
H	-2.865782	-3.046896	0.806412
H	-2.069655	-0.713838	1.285319
C	2.537434	-0.684374	0.779670
C	3.688364	-0.411810	0.041353
C	2.696425	-1.495755	1.907308
C	4.936104	-0.903904	0.409161
C	3.927731	-2.006674	2.292099
C	5.054591	-1.703309	1.537795
C	1.171041	1.652595	-0.203077
C	1.010146	2.722941	0.678578
C	1.495059	1.960571	-1.525021
C	1.172226	4.042490	0.276052
C	1.655357	3.270520	-1.952355
C	1.496059	4.313122	-1.046067
F	4.036909	-2.779661	3.366431
F	1.640758	-1.823164	2.648601
F	6.238009	-2.180349	1.891858
F	6.012216	-0.618292	-0.315693
F	3.644619	0.335495	-1.057529
F	0.698931	2.502688	1.951951
F	1.017776	5.039441	1.140584
F	1.653031	5.566089	-1.447584
F	1.959652	3.537227	-3.218530
F	1.642302	1.003083	-2.436012
C	0.193878	-1.014291	-0.908063
C	-0.662219	-0.524398	-1.895282
C	0.291765	-2.404293	-0.821723
C	-1.354047	-1.357718	-2.764161
C	-0.381004	-3.261868	-1.681673
C	-1.216828	-2.733974	-2.656018
F	1.016393	-2.966746	0.151197
F	-0.258390	-4.578777	-1.551720
F	-1.901629	-3.538395	-3.457242
F	-2.174232	-0.841729	-3.673655
F	-0.902585	0.783967	-2.018139
P	0.819133	-0.007306	0.523723

TS

C	-4.403507	-0.174825	-1.002809
C	-3.424984	-0.004441	-0.021695
C	-2.937104	-1.122713	0.665867
C	-3.438523	-2.392320	0.386086
C	-4.416153	-2.558413	-0.599015
C	-4.893488	-1.447776	-1.295473
C	-2.849892	1.346378	0.280594
C	-4.192727	2.226161	1.476726
C	-4.265794	1.279832	2.504753
C	-3.102439	0.945620	3.240159
C	-1.940028	1.634012	2.953683
O	-1.727795	1.403140	0.886981
C	-3.079808	-0.291709	4.104979
C	-5.474952	0.392054	2.603175
H	-4.794071	-3.554322	-0.833684
H	-3.063349	2.115501	-0.483990
H	-3.530314	3.086489	1.571115
H	-5.096038	2.409203	0.889206
H	-0.979418	1.254032	3.307988
H	-1.941009	2.679024	2.656733
H	-3.682072	-0.143342	5.015267
H	-3.488143	-1.171728	3.585354
H	-2.054310	-0.522970	4.421695
H	-6.328834	0.812437	2.058378
H	-5.250767	-0.602608	2.178695
H	-5.764667	0.240644	3.653208
H	-4.768020	0.693255	-1.558000
H	-5.642599	-1.575174	-2.077882
H	-3.051442	-3.261283	0.922112
H	-2.142097	-0.974894	1.400156
C	2.542485	-0.686339	0.624897
C	3.627817	-0.404403	-0.204469
C	2.803466	-1.510491	1.725200
C	4.901501	-0.906665	0.042786
C	4.061393	-2.032543	1.990762
C	5.117808	-1.724557	1.142724
C	1.072397	1.644279	-0.238899
C	0.951711	2.739215	0.616552
C	1.288299	1.920571	-1.589299
C	1.048543	4.049086	0.166256
C	1.386561	3.220218	-2.066175
C	1.268744	4.287211	-1.183265
F	4.262485	-2.818274	3.042954
F	1.822794	-1.837521	2.564753
F	6.326145	-2.211284	1.382673
F	5.909879	-0.609843	-0.770279
F	3.499341	0.367714	-1.279597
F	0.742416	2.558912	1.920752
F	0.929005	5.067777	1.012891
F	1.363603	5.530957	-1.631296
F	1.587184	3.453104	-3.359255
F	1.381839	0.941427	-2.483288
C	0.024429	-1.018965	-0.796220
C	-0.886557	-0.536850	-1.737141
C	0.105724	-2.408493	-0.685052
C	-1.639460	-1.380047	-2.545326
C	-0.632348	-3.274266	-1.479045
C	-1.517342	-2.754847	-2.413904
F	0.886999	-2.959390	0.250969
F	-0.525434	-4.590323	-1.321916
F	-2.260630	-3.567026	-3.152790
F	-2.501396	-0.870754	-3.419027
F	-1.118544	0.769122	-1.881604
P	0.800915	-0.003640	0.553989

Product

C	-4.598260	-0.723849	-0.856101
C	-3.669902	-0.139888	0.014801
C	-3.291383	-0.835129	1.164326
C	-3.831141	-2.093242	1.440296
C	-4.748126	-2.671932	0.565069
C	-5.132673	-1.981544	-0.586390
C	-3.102595	1.233928	-0.306554
C	-4.143894	2.328679	-0.033718

C	-4.416685	2.388662	1.449309
C	-3.330989	2.381878	2.236080
C	-1.990183	2.357569	1.543912
O	-1.903722	1.476525	0.416660
C	-3.299099	2.404783	3.735989
C	-5.848897	2.353260	1.889163
H	-5.155267	-3.662043	0.772352
H	-2.826238	1.258135	-1.373723
H	-3.744656	3.289261	-0.401904
H	-5.058033	2.111267	-0.603602
H	-1.200528	2.026994	2.234734
H	-1.721742	3.378071	1.210829
H	-4.291350	2.543141	4.180068
H	-2.874764	1.464747	4.125324
H	-2.650024	3.219005	4.096216
H	-6.415708	3.175772	1.424653
H	-6.314706	1.412321	1.551383
H	-5.968307	2.423754	2.976347
H	-4.884265	-0.198345	-1.771516
H	-5.838279	-2.432981	-1.284844
H	-3.516639	-2.632300	2.335509
H	-2.549093	-0.392990	1.831710
C	2.381589	-0.835050	0.875403
C	2.811259	-1.992592	0.229138
C	3.263157	-0.268814	1.798008
C	4.044934	-2.571674	0.511456
C	4.501732	-0.819053	2.091844
C	4.890538	-1.985378	1.443524
C	1.419381	1.399384	-0.401043
C	1.388651	2.722948	0.022595
C	2.034138	1.129554	-1.622903
C	1.923566	3.751310	-0.747196
C	2.575013	2.132648	-2.410810
C	2.516751	3.450885	-1.965332
F	5.310624	-0.245332	2.975343
F	2.917573	0.864102	2.418533
F	6.068450	-2.531712	1.708880
F	4.423671	-3.678822	-0.117074
F	2.067381	-2.597843	-0.693274
F	0.848760	3.047840	1.196042
F	1.877974	5.009103	-0.322113
F	3.031974	4.419352	-2.707927
F	3.144075	1.854695	-3.578110
F	2.095579	-0.124589	-2.068755
C	-0.210378	-1.000752	-0.412659
C	-0.837389	-0.605484	-1.589797
C	-0.554233	-2.254157	0.085521
C	-1.757824	-1.420199	-2.235667
C	-1.460030	-3.093131	-0.542454
C	-2.072937	-2.666236	-1.712779
F	-0.003243	-2.664642	1.234102
F	-1.780148	-4.266922	-0.009852
F	-2.977321	-3.429016	-2.310479
F	-2.393131	-0.976700	-3.317769
F	-0.622985	0.598053	-2.129230
P	0.762114	0.082772	0.721342

As_cat

R1_cat

C	4.819003	0.660759	-0.744432
C	3.648042	0.601938	-1.502056
C	3.189799	-0.623703	-2.003388
C	3.900997	-1.786710	-1.733363
C	5.065106	-1.728589	-0.957862
C	5.525859	-0.508487	-0.464856
C	2.840601	1.829909	-1.699424
O	1.704672	1.828438	-2.116821
H	5.614293	-2.644736	-0.735157
H	3.342767	2.782238	-1.408972
H	5.159701	1.620367	-0.348951
H	6.428458	-0.469852	0.145084
H	3.551858	-2.744845	-2.120893
H	2.271934	-0.639321	-2.594987
C	-2.146873	-0.947220	-0.344579
C	-2.983869	-0.597564	0.712201
C	-2.566499	-2.010484	-1.146382
C	-4.176065	-1.269499	0.963987
C	-3.746035	-2.704097	-0.915554
C	-4.556100	-2.326257	0.148290
C	-0.752441	1.748214	-0.213081
C	-1.019085	2.744080	-1.149752
C	-0.624036	2.139988	1.117831
C	-1.160535	4.078857	-0.788634
C	-0.756257	3.465399	1.505101
C	-1.027257	4.435770	0.545978
F	-4.104936	-3.716326	-1.696821
F	-1.819129	-2.404453	-2.178042
F	-5.687213	-2.973883	0.384127
F	-4.950601	-0.907193	1.980951
F	-2.684827	0.403041	1.537337
F	-1.157563	2.434886	-2.436288
F	-1.420057	5.007215	-1.702926
F	-1.158383	5.703418	0.909610
F	-0.625231	3.816906	2.780339
F	-0.353532	1.250699	2.070066
C	0.805499	-0.814674	0.329197
C	1.846617	-0.111853	0.930528
C	0.866605	-2.203086	0.421618
C	2.875870	-0.746916	1.612833
C	1.880396	-2.869209	1.097977
C	2.896617	-2.132615	1.691408
F	-0.054552	-2.952635	-0.193398
F	1.900466	-4.196693	1.152307
F	3.896814	-2.748731	2.306360
F	3.858178	-0.037273	2.159221
F	1.932167	1.219565	0.835657
As	-0.485653	-0.047332	-0.959380

R1_cat_R2

C	-4.243683	-0.055970	-1.370579
C	-3.341017	0.086617	-0.316136
C	-2.899772	-1.037436	0.395611
C	-3.364955	-2.300005	0.044136
C	-4.256130	-2.443768	-1.026527
C	-4.694124	-1.325306	-1.734850
C	-2.804151	1.428076	0.014603
C	-5.056142	2.268909	2.100744
C	-5.083968	0.944454	2.313560
C	-4.133720	0.276507	3.249543
C	-2.912415	0.770978	3.499696
O	-1.816796	1.604261	0.694418
C	-4.598610	-1.007536	3.888842
C	-6.105656	0.070377	1.630492
H	-4.607217	-3.436965	-1.310725
H	-3.354566	2.287750	-0.431389
H	-4.361581	2.919188	2.634755
H	-5.752112	2.736643	1.400918
H	-2.246061	0.265759	4.202140

H	-2.531758	1.664987	3.005120
H	-5.544391	-0.862081	4.433932
H	-4.781233	-1.783040	3.127661
H	-3.847306	-1.386907	4.592855
H	-6.698877	0.650865	0.911794
H	-5.625631	-0.764245	1.094323
H	-6.795783	-0.371386	2.366084
H	-4.576374	0.827393	-1.920547
H	-5.382162	-1.442701	-2.572200
H	-3.030221	-3.179645	0.596457
H	-2.202208	-0.893431	1.222896
C	2.637505	-0.693904	0.765990
C	3.757564	-0.296439	0.039788
C	2.849815	-1.647605	1.763601
C	5.022769	-0.821672	0.284835
C	4.097824	-2.194941	2.026135
C	5.191655	-1.774832	1.279502
C	1.104099	1.783030	-0.103545
C	0.891731	2.857355	0.757081
C	1.427491	2.077542	-1.425933
C	1.007223	4.176108	0.334606
C	1.545892	3.384779	-1.875318
C	1.336833	4.435673	-0.988518
F	4.254580	-3.107693	2.978490
F	1.828554	-2.076713	2.506545
F	6.392064	-2.282893	1.516228
F	6.068837	-0.417318	-0.428034
F	3.674738	0.613302	-0.928299
F	0.570168	2.643748	2.031549
F	0.804108	5.181778	1.179418
F	1.450513	5.687130	-1.410297
F	1.853384	3.642187	-3.142763
F	1.617562	1.107320	-2.316467
C	0.113824	-0.988965	-0.895074
C	-0.713812	-0.466568	-1.886211
C	0.216568	-2.377736	-0.849994
C	-1.388033	-1.273974	-2.793675
C	-0.436866	-3.212187	-1.746900
C	-1.253114	-2.653087	-2.721137
F	0.932476	-2.960650	0.118017
F	-0.316290	-4.532730	-1.656218
F	-1.922022	-3.432799	-3.559505
F	-2.189889	-0.733208	-3.705680
F	-0.947421	0.845768	-1.979748
As	0.780055	0.012358	0.676826

TS

C	-4.428770	-0.283015	-1.081511
C	-3.483968	-0.117406	-0.067324
C	-3.009136	-1.240373	0.621310
C	-3.486952	-2.510710	0.306854
C	-4.430111	-2.672059	-0.712140
C	-4.896756	-1.556302	-1.407227
C	-2.926286	1.234883	0.262982
C	-4.290774	2.090514	1.430180
C	-4.369177	1.149504	2.463458
C	-3.216000	0.843107	3.227617
C	-2.062797	1.553540	2.964164
O	-1.812505	1.293306	0.887722
C	-3.189617	-0.387450	4.101566
C	-5.564093	0.241593	2.543552
H	-4.789103	-3.668255	-0.973585
H	-3.126056	2.006424	-0.502924
H	-3.650137	2.965801	1.537114
H	-5.185752	2.253947	0.824469
H	-1.102598	1.199306	3.346144
H	-2.076094	2.591581	2.645086
H	-3.826436	-0.249879	4.989624
H	-3.557090	-1.280900	3.574310
H	-2.169900	-0.589662	4.454355
H	-6.412336	0.640779	1.974692
H	-5.312981	-0.753378	2.135519
H	-5.874787	0.096350	3.588471
H	-4.781840	0.588957	-1.637973
H	-5.618673	-1.679602	-2.215372
H	-3.108701	-3.383394	0.842934
H	-2.241971	-1.096089	1.385426
C	2.626037	-0.638361	0.638842
C	3.682982	-0.240600	-0.176529
C	2.935422	-1.558131	1.643490
C	4.973122	-0.737218	-0.015319
C	4.209758	-2.077114	1.825222
C	5.235792	-1.660626	0.986552
C	0.964656	1.769352	-0.195275
C	0.766201	2.880389	0.618700
C	1.192821	2.006985	-1.548104
C	0.802759	4.178464	0.126345
C	1.235374	3.292988	-2.069178
C	1.041729	4.381311	-1.226055
F	4.455534	-2.957812	2.789532
F	1.986164	-1.979354	2.482029
F	6.460735	-2.141311	1.144275
F	5.954755	-0.332014	-0.814820
F	3.516507	0.647261	-1.154913
F	0.532634	2.728358	1.925180
F	0.609925	5.218218	0.933519
F	1.081829	5.613088	-1.715098
F	1.452416	3.493380	-3.365162
F	1.355975	1.000170	-2.401755
C	-0.026719	-1.043717	-0.783433
C	-0.917090	-0.573095	-1.745665
C	0.098847	-2.427748	-0.686289
C	-1.626563	-1.427887	-2.580688
C	-0.593543	-3.307673	-1.506157
C	-1.469930	-2.800563	-2.456227
F	0.882409	-2.958538	0.260095
F	-0.451804	-4.622281	-1.364381
F	-2.171996	-3.624491	-3.222160
F	-2.479489	-0.936262	-3.473659
F	-1.172538	0.729393	-1.886327
As	0.744050	0.028359	0.687287

Product

C	-4.346219	-0.612997	-1.158211
C	-3.544174	-0.306314	-0.055901
C	-3.045326	-1.350395	0.729825
C	-3.352107	-2.675131	0.420881
C	-4.159552	-2.973056	-0.678311
C	-4.655541	-1.936966	-1.468924
C	-3.238027	1.145711	0.267538
C	-4.400888	1.789591	1.038165
C	-4.488365	1.181735	2.416662
C	-3.326054	1.098480	3.081025
C	-2.110279	1.636290	2.366924
O	-2.017514	1.249981	0.990531
C	-3.100889	0.525592	4.449197
C	-5.822495	0.665159	2.862955
H	-4.388588	-4.010267	-0.926818

H	-3.087924	1.685496	-0.680453
H	-4.219077	2.876267	1.102155
H	-5.330847	1.644464	0.470837
H	-1.185368	1.267287	2.833443
H	-2.087240	2.741164	2.427019
H	-4.033213	0.239067	4.948932
H	-2.452285	-0.364096	4.391680
H	-2.581710	1.254111	5.092386
H	-6.580582	1.463021	2.815735
H	-6.155644	-0.134231	2.180181
H	-5.809636	0.262470	3.882288
H	-4.716108	0.193864	-1.796311
H	-5.268923	-2.160103	-2.342697
H	-2.953013	-3.481542	1.038866
H	-2.396579	-1.115921	1.575593
C	2.610913	-0.492056	0.730061
C	3.651159	-0.133247	-0.123363
C	2.951385	-1.294781	1.820382
C	4.961656	-0.549304	0.091693
C	4.246978	-1.731316	2.057702
C	5.258901	-1.351513	1.184565
C	0.876618	1.751601	-0.343594
C	0.684838	2.926247	0.377495
C	1.082866	1.880021	-1.715503
C	0.696105	4.180103	-0.220874
C	1.098875	3.118346	-2.342187
C	0.905729	4.271763	-1.589789
F	4.525985	-2.501275	3.103686
F	2.009903	-1.683003	2.682541
F	6.503539	-1.754341	1.394292
F	5.928944	-0.184584	-0.743397
F	3.444780	0.632546	-1.193029
F	0.485143	2.880631	1.698022
F	0.510351	5.280417	0.501770
F	0.921425	5.457617	-2.181162
F	1.294789	3.212090	-3.653017
F	1.261289	0.809801	-2.484335
C	0.018695	-1.145711	-0.683981
C	-0.898906	-0.788824	-1.668133
C	0.259941	-2.507150	-0.524220
C	-1.504987	-1.727600	-2.491254
C	-0.339032	-3.472306	-1.322890
C	-1.230328	-3.076342	-2.310632
F	1.069614	-2.932133	0.452727
F	-0.088319	-4.763490	-1.128988
F	-1.841188	-3.983808	-3.059756
F	-2.365157	-1.346562	-3.429426
F	-1.259500	0.485726	-1.843289
As	0.706774	0.087232	0.691798

Sb_cat

R1_cat

C	4.974875	0.509891	-0.445971
C	3.864904	0.372374	-1.281619
C	3.500381	-0.888110	-1.774388
C	4.242277	-2.006876	-1.418199
C	5.340545	-1.870680	-0.560361
C	5.709018	-0.616578	-0.076222
C	3.015633	1.550874	-1.561181
O	1.891447	1.480383	-2.012007
H	5.910512	-2.753326	-0.266763
H	3.460381	2.538704	-1.303128
H	5.241984	1.495630	-0.058722
H	6.560426	-0.517958	0.597028
H	3.966316	-2.991499	-1.797889
H	2.631109	-0.967284	-2.430187
C	-2.360664	-0.883858	-0.362541
C	-3.189614	-0.391056	0.640639
C	-2.784233	-2.054345	-0.989007
C	-4.357648	-1.041502	1.026896
C	-3.940126	-2.733763	-0.628679
C	-4.729847	-2.219122	0.391919
C	-0.665458	1.946136	-0.281194
C	-0.758733	3.041323	-1.130352
C	-0.585876	2.202279	1.082987
C	-0.781486	4.348427	-0.657671
C	-0.604290	3.493928	1.589462
C	-0.704225	4.568611	0.711154
F	-4.299797	-3.853105	-1.248430
F	-2.067261	-2.566610	-1.998065
F	-5.840753	-2.846099	0.753029
F	-5.123293	-0.542516	1.992522
F	-2.911844	0.748085	1.277371
F	-0.837235	2.856868	-2.451614
F	-0.876276	5.378211	-1.493342
F	-0.724033	5.807317	1.184285
F	-0.523440	3.717965	2.897514
F	-0.472909	1.201073	1.956053
Sb	-0.531738	-0.001044	-1.224467
C	0.844062	-0.884890	0.236915
C	1.833337	-0.224891	0.957172
C	0.868449	-2.273517	0.293936
C	2.792380	-0.902607	1.699780
C	1.809281	-2.988457	1.023633
C	2.784260	-2.290826	1.725346
F	-0.030879	-2.974550	-0.410750
F	1.803004	-4.317745	1.034888
F	3.720330	-2.947607	2.397744
F	3.738203	-0.234213	2.354378
F	1.947642	1.109517	0.924285

R1_cat_R2

C	4.298429	-0.058793	1.466110
C	3.428459	-0.019045	0.375496
C	3.063970	-1.201152	-0.284019
C	3.569505	-2.418725	0.156935
C	4.424082	-2.459593	1.265710
C	4.788353	-1.283505	1.920278
C	2.842130	1.272952	-0.042580
C	5.083548	2.149378	-2.102639
C	5.163108	0.818760	-2.258334
C	4.248067	0.075596	-3.172894
C	3.022110	0.526496	-3.478482
O	1.853439	1.365954	-0.741710
C	4.755027	-1.231369	-3.728219
C	6.212319	0.016269	-1.530262
H	4.804906	-3.418055	1.621466
H	3.345580	2.183405	0.351021
H	4.372122	2.751147	-2.670241
H	5.755821	2.672109	-1.418881
H	2.384302	-0.032720	-4.166363
H	2.613136	1.442190	-3.050230
H	5.705348	-1.093088	-4.267094
H	4.945390	-1.956412	-2.920686
H	4.025416	-1.670472	-4.420207
H	6.777884	0.651538	-0.836159
H	5.761874	-0.811364	-0.958833
H	6.923964	-0.431288	-2.241349

H	4.571491	0.868687	1.974504
H	5.448483	-1.321466	2.786834
H	3.293083	-3.343080	-0.352782
H	2.396086	-1.138856	-1.145089
C	-2.817358	-0.677601	-0.784786
C	-3.889536	-0.124189	-0.091844
C	-3.107697	-1.784722	-1.579696
C	-5.172951	-0.658443	-0.155137
C	-4.373409	-2.348818	-1.665182
C	-5.412614	-1.777441	-0.941651
C	-1.005720	1.963485	0.009884
C	-0.695998	3.078051	-0.758436
C	-1.366817	2.181194	1.334101
C	-0.752702	4.369997	-0.249779
C	-1.432671	3.457226	1.875579
C	-1.125993	4.553403	1.075365
F	-4.601150	-3.411630	-2.430482
F	-2.141274	-2.347030	-2.318063
F	-6.632000	-2.293756	-1.009335
F	-6.172165	-0.103478	0.524190
F	-3.743382	0.966428	0.662809
F	-0.329276	2.927162	-2.035703
F	-0.456105	5.420593	-1.009441
F	-1.187646	5.777603	1.582276
F	-1.778110	3.645395	3.145742
F	-1.646428	1.155547	2.138621
Sb	-0.732535	0.034695	-0.937603
C	-0.004951	-1.020006	0.840504
C	0.762818	-0.482482	1.867552
C	-0.105001	-2.406159	0.811669
C	1.403490	-1.275537	2.812021
C	0.511666	-3.232932	1.741312
C	1.281187	-2.656763	2.744072
F	-0.791150	-2.993358	-0.179067
F	0.402258	-4.555788	1.662461
F	1.918395	-3.424024	3.618859
F	2.163203	-0.723844	3.754426
F	0.976810	0.835449	1.962663

TS

C	-4.327142	-0.423445	-1.275351
C	-3.482721	-0.321883	-0.168816
C	-3.123636	-1.478574	0.532742
C	-3.609428	-2.721418	0.132603
C	-4.450920	-2.819278	-0.979069
C	-4.808133	-1.668502	-1.681488
C	-2.900874	0.999679	0.243950
C	-4.299939	1.915057	1.231445
C	-4.555191	0.996385	2.260137
C	-3.526955	0.658898	3.177113
C	-2.325964	1.323287	3.070120
O	-1.849988	0.987772	0.982527
C	-3.670852	-0.557473	4.058662
C	-5.786144	0.139847	2.194141
H	-4.815039	-3.793996	-1.305897
H	-2.953091	1.773674	-0.543657
H	-3.652515	2.771687	1.421086
H	-5.109192	2.111217	0.523591
H	-1.444131	0.942291	3.591533
H	-2.241137	2.340116	2.699914
H	-4.442554	-0.394898	4.827050
H	-3.966149	-1.449381	3.485365
H	-2.728245	-0.775275	4.576807
H	-6.533724	0.558208	1.510023
H	-5.522785	-0.872610	1.840131
H	-6.236964	0.028587	3.191049
H	-4.585695	0.474554	-1.842337
H	-5.448588	-1.742652	-2.561007
H	-3.316518	-3.621798	0.675797
H	-2.435592	-1.384858	1.375999
C	2.841873	-0.475759	0.684580
C	3.825291	0.124124	-0.095742
C	3.272156	-1.512602	1.510221
C	5.148732	-0.306174	-0.094239
C	4.581663	-1.974125	1.538448
C	5.525343	-1.364019	0.722292
C	0.764108	1.952392	-0.132428
C	0.368730	3.084277	0.567014
C	1.044306	2.108650	-1.484060
C	0.260659	4.332712	-0.032104
C	0.949828	3.341177	-2.117011
C	0.558237	4.455979	-1.383438
F	4.940643	-2.974563	2.337931
F	2.406298	-2.105254	2.345515
F	6.784491	-1.782044	0.731993
F	6.057755	0.291455	-0.860044
F	3.552285	1.169599	-0.880723
F	0.072180	2.998987	1.873060
F	-0.120902	5.399628	0.667164
F	0.463705	5.639173	-1.976761
F	1.217939	3.467596	-3.413169
F	1.390166	1.060224	-2.230340
Sb	0.703303	0.073944	0.951461
C	-0.037346	-1.155998	-0.699051
C	-0.869070	-0.745926	-1.734758
C	0.147447	-2.527096	-0.568439
C	-1.484364	-1.646006	-2.596572
C	-0.449640	-3.458734	-1.406995
C	-1.280599	-3.008385	-2.424787
F	0.906676	-2.993014	0.433889
F	-0.257919	-4.763282	-1.230584
F	-1.893522	-3.877483	-3.218194
F	-2.291820	-1.214039	-3.561423
F	-1.165019	0.545178	-1.923302

Product

C	-4.182311	-0.885425	-1.320528
C	-3.503686	-0.576111	-0.138294
C	-3.060531	-1.618024	0.681106
C	-3.293593	-2.945856	0.322707
C	-3.976632	-3.246644	-0.856294
C	-4.422694	-2.211126	-1.677576
C	-3.271945	0.878501	0.227941
C	-4.528577	1.485449	0.872316
C	-4.740750	0.874622	2.236039
C	-3.655738	0.853399	3.025293
C	-2.405275	1.450219	2.429127
O	-2.142302	1.000414	1.091151
C	-3.548891	0.293985	4.412993
C	-6.087671	0.290112	2.534904
H	-4.148461	-4.285374	-1.141696
H	-3.028500	1.434415	-0.691057
H	-4.389460	2.577135	0.952744
H	-5.387355	1.311369	0.209501
H	-1.513913	1.169526	3.008962
H	-2.460154	2.554186	2.426150
H	-4.512450	-0.048090	4.807409
H	-2.847181	-0.556164	4.432875

H	-3.148805	1.051182	5.106076
H	-6.875310	1.048334	2.400671
H	-6.302070	-0.524659	1.823463
H	-6.166091	-0.111825	3.551546
H	-4.505826	-0.078500	-1.983130
H	-4.938360	-2.436249	-2.611750
H	-2.936133	-3.751064	0.966790
H	-2.514122	-1.380619	1.595486
C	2.809163	-0.260595	0.776676
C	3.756428	0.321076	-0.059673
C	3.294999	-1.167000	1.716433
C	5.108770	0.000353	0.010253
C	4.635228	-1.515743	1.814637
C	5.546942	-0.924625	0.948715
C	0.596710	1.932244	-0.300321
C	0.178529	3.102947	0.317091
C	0.857756	1.996532	-1.664517
C	0.017504	4.298469	-0.372523
C	0.710299	3.172832	-2.386875
C	0.288682	4.326913	-1.734118
F	5.052294	-2.392213	2.722730
F	2.451871	-1.738813	2.587261
F	6.833411	-1.237020	1.023365
F	5.985130	0.575129	-0.808131
F	3.414407	1.235212	-0.970082
F	-0.083091	3.109661	1.632851
F	-0.384979	5.401847	0.252064
F	0.144611	5.455772	-2.414846
F	0.960847	3.209792	-3.691533
F	1.248226	0.912249	-2.332999
Sb	0.644559	0.147408	0.935694
C	0.053930	-1.260936	-0.620760
C	-0.805849	-0.989945	-1.678235
C	0.416074	-2.589133	-0.439935
C	-1.263027	-1.981685	-2.535019
C	-0.028363	-3.611729	-1.268165
C	-0.879638	-3.299518	-2.319741
F	1.206830	-2.921686	0.590588
F	0.331729	-4.873606	-1.053076
F	-1.346490	-4.260462	-3.106001
F	-2.083635	-1.688214	-3.538996
F	-1.259490	0.250568	-1.896038

METHYLENE IMINE (R1) + 1,3-BUTADIENE (R2)

BF3

R1_cat

C	2.057662	0.032624	-0.000012
H	1.968524	1.122835	0.000070
H	3.037875	-0.453172	0.000174
N	0.980715	-0.633570	0.000371
H	1.053436	-1.653285	0.000804
B	-0.535964	0.045735	-0.000126
F	-1.115651	-0.423549	1.153367
F	-0.279524	1.402199	-0.000013
F	-1.114935	-0.423739	-1.153681

R1_cat_R2

C	3.074622	-0.908741	-0.166580
C	2.442318	-0.156739	0.744742
C	1.527724	0.949969	0.424138
C	1.676624	1.774015	-0.617993
C	-0.021221	-1.761064	-0.460553
H	0.665196	1.077483	1.084457
H	2.577889	-0.387590	1.806532
H	2.936380	-0.727472	-1.236783
H	3.758512	-1.706462	0.127597
H	-0.000585	-1.958478	0.614382
H	0.582205	-2.360845	-1.146995
H	2.551399	1.713980	-1.271370
H	0.926036	2.537644	-0.824731
N	-0.765709	-0.818793	-0.868031
H	-0.775442	-0.623800	-1.870737
B	-1.711247	0.108527	0.108931
F	-3.001027	-0.261842	-0.185455
F	-1.304910	-0.234111	1.389586
F	-1.416929	1.411488	-0.236721

TS

C	2.368095	-0.904983	-0.096198
C	2.148151	0.164883	0.760958
C	1.440215	1.316073	0.362137
C	0.968535	1.438659	-0.915090
C	0.497203	-1.559062	-0.386403
H	1.067878	1.987507	1.135618
H	2.317454	0.019747	1.830832
H	2.520031	-0.715289	-1.160409
H	2.883720	-1.787740	0.286960
H	0.301490	-1.874772	0.638334
H	0.832091	-2.327365	-1.086960
H	1.438228	0.914639	-1.747402
H	0.227707	2.198232	-1.163455
N	-0.296871	-0.586447	-0.838129
H	-0.441811	-0.552303	-1.844352
B	-1.447322	-0.003394	0.124335
F	-2.510323	-0.885966	0.057720
F	-0.857812	0.016038	1.391569
F	-1.783563	1.261708	-0.337773

Product

C	-2.114388	1.240670	-0.137652
C	-2.776902	-0.110538	-0.090094
C	-2.084234	-1.244801	0.031992
C	-0.584696	-1.285619	0.084344
C	-0.660621	1.156288	0.302606
H	-2.600959	-2.204731	0.092999
H	-3.866545	-0.146418	-0.155401
H	-2.189383	1.662293	-1.155335
H	-2.647873	1.945056	0.517790
H	-0.575172	0.994679	1.385896
H	-0.083869	2.051606	0.043679
H	-0.171586	-2.064741	-0.569051
H	-0.212627	-1.491177	1.100159
N	0.019828	0.000730	-0.331887
H	-0.071638	0.089964	-1.349433
B	1.633953	0.016763	-0.018125
F	2.083356	1.210645	-0.546592
F	1.724953	-0.051324	1.359767
F	2.129040	-1.099483	-0.662580

H_cat

R1_cat

C	-3.778094	0.474168	-0.471089
H	-3.471297	0.455051	-1.525131
H	-4.853630	0.578258	-0.268846
N	-2.890078	0.378358	0.432566

H	-3.323199	0.413516	1.364293
C	2.415927	0.115852	-0.124661
C	1.978809	-1.174698	-0.117185
C	0.218434	0.143428	0.089975
N	1.302044	0.918266	0.005651
N	0.608133	-1.130538	0.017355
H	3.415888	0.527329	-0.210632
H	2.522542	-2.110049	-0.195471
C	-0.296539	-2.279003	0.076866
H	-1.318249	-1.896951	0.179570
H	-0.204894	-2.862285	-0.846513
H	-0.039498	-2.900278	0.942420
C	1.304747	2.380247	0.045859
H	1.723669	2.769444	-0.889133
H	0.272701	2.726590	0.160157
H	1.904418	2.719669	0.898117
H	-0.828851	0.457142	0.203575

TS

C	-3.734795	0.175334	-0.383436
C	-2.997328	1.051101	0.398613
C	-2.038307	0.600898	1.324600
C	-1.812847	-0.747987	1.490081
C	-2.173217	-0.764272	-1.416455
H	-1.347394	1.322856	1.766633
H	-3.024068	2.117103	0.154258
H	-4.022792	-0.800090	0.011791
H	-4.407369	0.578259	-1.143052
H	-1.768153	0.142766	-1.877959
H	-2.915578	-1.297053	-2.020472
N	-1.386286	-1.418485	-0.568348
H	-1.760810	-2.357306	-0.401450
H	-2.627573	-1.462767	1.381594
H	-0.961452	-1.098798	2.077945
C	3.005848	1.253975	0.006953
C	3.437205	-0.030270	0.148411
C	1.250069	-0.077720	-0.186909
N	1.642967	1.198922	-0.200573
N	2.327341	-0.837902	0.022387
H	3.547845	2.193094	0.034639
H	4.429182	-0.431783	0.325249
C	2.300300	-2.297738	0.097934
H	1.275298	-2.624387	-0.109213
H	2.980864	-2.713728	-0.653659
H	2.603816	-2.620729	1.100527
C	0.768685	2.347934	-0.424075
H	1.062082	2.857677	-1.349358
H	-0.261437	1.984295	-0.506767
H	0.847694	3.036747	0.425242
H	0.228002	-0.491442	-0.324515

Product

C	-3.582466	-0.105691	-1.231896
C	-4.209272	0.061941	0.126721
C	-3.488416	0.078462	1.249816
C	-1.996278	-0.132160	1.254672
C	-2.072192	0.100291	-1.155324
H	-3.972148	0.237803	2.216186
H	-5.293850	0.180436	0.177117
H	-3.814110	-1.107510	-1.634225
H	-4.022324	0.611088	-1.941935
H	-1.858764	1.173760	-1.012312
H	-1.580385	-0.213245	-2.087461
N	-1.457365	-0.618904	-0.025463
H	-1.729086	-1.602415	-0.112895
H	-1.718657	-0.850895	2.040723
H	-1.483234	0.813359	1.515498
C	3.497143	1.001848	0.004803
C	3.758540	-0.334819	0.004867
C	1.561725	-0.070333	-0.017798
N	2.125337	1.140609	-0.009396
N	2.539994	-0.979582	-0.009223
H	4.164421	1.857022	0.013661
H	4.698831	-0.875520	0.013780
C	2.337359	-2.427370	-0.014426
H	1.260148	-2.622777	-0.021586
H	2.797683	-2.857961	-0.911105
H	2.786394	-2.862558	0.885729
C	1.395210	2.406874	-0.014152
H	1.661404	2.979473	-0.910066
H	0.322834	2.185264	-0.023107
H	1.647130	2.977726	0.886980
H	0.469843	-0.292152	-0.030104

H2_cat

R1_cat

C	0.021132	3.389067	0.780399
H	0.028397	4.355769	1.304805
H	0.027662	2.480310	1.400394
N	0.006018	3.262047	-0.484113
H	0.001446	4.175008	-0.953891
C	5.237451	-0.219296	0.057865
C	4.378860	-1.307255	-0.072683
C	4.694617	1.058406	-0.023557
H	6.305415	-0.364678	0.212878
C	3.011794	-1.148805	-0.280347
C	4.937975	-2.701009	0.042106
C	3.329544	1.244370	-0.206385
C	5.591249	2.262956	0.048264
C	2.474550	0.143155	-0.333702
H	2.371808	-2.017764	-0.401171
F	6.213429	-2.753638	-0.367523
F	4.915807	-3.134283	1.309519
F	4.239128	-3.578545	-0.687620
H	2.922790	2.256460	-0.247583
F	6.697210	2.020128	0.759920
F	5.984833	2.657207	-1.171552
F	4.966030	3.310982	0.609282
N	1.121221	0.425209	-0.576298
C	-0.004106	-0.266946	-0.195093
H	0.951765	1.379331	-0.896370
S	-0.010297	-1.702604	0.645270
N	-1.125035	0.431804	-0.578022
H	-0.951724	1.388782	-0.887729
C	-2.479104	0.150679	-0.337097
C	-3.330456	1.248968	-0.178244
C	-3.019358	-1.141865	-0.317008
C	-4.696752	1.060917	0.002007
H	-2.921188	2.261095	-0.192847
C	-4.385389	-1.303102	-0.111301
H	-2.381017	-2.008378	-0.462437
C	-5.242087	-0.216194	0.049062
C	-5.579061	2.272647	0.115039
C	-4.949858	-2.697041	-0.031830
H	-6.310224	-0.363374	0.199936
F	-5.003862	3.230674	0.860472
F	-5.821998	2.817728	-1.086421
F	-6.761833	1.982908	0.666442

F	-4.952523	-3.152119	1.228161
F	-4.240677	-3.564101	-0.763933
F	-6.217965	-2.738301	-0.465218

R1_cat_R2

C	1.857530	-3.564335	1.337821
C	0.524999	-3.702684	1.297476
C	-0.454464	-2.626153	1.545505
C	-0.188013	-1.316729	1.464617
C	0.043732	-3.401253	-2.787712
H	-1.471147	-2.943386	1.797893
H	0.106734	-4.690039	1.074391
H	2.329284	-2.618998	1.618709
H	2.522427	-4.402576	1.123767
H	0.214621	-4.474374	-2.615792
H	-0.226211	-3.094074	-3.806364
H	0.806296	-0.945407	1.193827
H	-0.948885	-0.562862	1.677695
N	0.141397	-2.519399	-1.879364
H	0.397738	-2.941811	-0.975303
C	5.006618	0.536963	0.163979
C	4.281573	1.707780	-0.040455
C	4.395898	-0.677985	-0.131695
H	6.030071	0.572379	0.534642
C	2.978080	1.690247	-0.531578
C	4.909054	3.035279	0.294479
C	3.095633	-0.721327	-0.620327
C	5.116709	-1.967912	0.140914
C	2.373651	0.461653	-0.812805
H	2.437388	2.617751	-0.700980
F	6.246978	2.976434	0.236606
F	4.506959	3.997504	-0.545039
F	4.585507	3.431631	1.531792
H	2.633183	-1.683775	-0.849395
F	6.438412	-1.848527	-0.016461
F	4.908211	-2.387044	1.402187
F	4.693197	-2.956053	-0.663565
N	1.070104	0.334072	-1.326661
C	-0.072796	0.899617	-0.805262
H	0.911464	-0.565779	-1.783487
S	-0.110646	2.206363	0.221244
N	-1.174178	0.201120	-1.242400
H	-0.955512	-0.724513	-1.618120
C	-2.483634	0.302606	-0.740055
C	-3.143807	-0.888085	-0.434531
C	-3.152311	1.522921	-0.582233
C	-4.453105	-0.862756	0.039571
H	-2.620412	-1.839547	-0.550952
C	-4.455157	1.523221	-0.098217
H	-2.656575	2.456130	-0.836496
C	-5.123154	0.339594	0.218642
C	-5.106640	-2.175920	0.363106
C	-5.160940	2.835427	0.117005
H	-6.146263	0.359045	0.590567
F	-5.219049	-2.951123	-0.726651
F	-4.378287	-2.876591	1.251485
F	-6.329641	-2.022451	0.877659
F	-4.679221	3.797390	-0.678979
F	-5.029279	3.262582	1.379842
F	-6.476216	2.728301	-0.122766

TS

C	2.905053	4.123656	-1.297775
C	2.086956	4.330326	-2.395276
C	1.190876	3.342487	-2.843124
C	1.117710	2.126473	-2.197366
C	1.448425	3.777517	0.202498
H	0.409164	3.624299	-3.551388
H	1.975731	5.344645	-2.787549
H	3.302494	3.127419	-1.097751
H	3.519043	4.943005	-0.918765
H	1.143352	4.825658	0.264207
H	2.234194	3.480491	0.904780
H	1.997694	1.684980	-1.730133
H	0.310083	1.425114	-2.421508
N	0.591475	2.841634	-0.200466
H	-0.231872	3.289003	-0.611697
C	4.986388	-1.026772	0.426969
C	4.045527	-2.046443	0.344858
C	4.516201	0.284385	0.462864
H	6.051791	-1.245924	0.463502
C	2.671773	-1.803747	0.285834
C	4.496658	-3.484092	0.340206
C	3.159014	0.552416	0.429484
C	5.467801	1.446178	0.479771
C	2.213230	-0.481502	0.327169
H	1.970496	-2.629591	0.206354
F	5.816826	-3.591509	0.138272
F	4.221528	-4.084872	1.505390
F	3.880845	-4.188491	-0.617328
H	2.811552	1.583591	0.489315
F	6.638755	1.137332	1.039947
F	5.724224	1.883346	-0.765810
F	4.954644	2.497488	1.146983
N	0.890915	-0.041151	0.254900
C	-0.302938	-0.703884	0.219709
H	0.816666	0.987412	0.185895
S	-0.526980	-2.350826	0.273092
N	-1.327467	0.222928	0.145337
H	-1.053993	1.180444	0.356836
C	-2.714716	0.002882	0.148316
C	-3.512445	0.912649	0.846517
C	-3.325705	-1.033038	-0.571403
C	-4.899536	0.792827	0.824076
H	-3.049616	1.716939	1.421686
C	-4.710471	-1.147431	-0.555292
H	-2.723883	-1.737958	-1.137973
C	-5.516258	-0.242096	0.133791
C	-5.719936	1.828521	1.541355
C	-5.360879	-2.292263	-1.285623
H	-6.600606	-0.340406	0.125586
F	-5.184565	2.142726	2.730363
F	-5.788222	2.970366	0.838203
F	-6.974272	1.417445	1.753030
F	-5.569785	-3.337035	-0.474283
F	-4.608205	-2.725602	-2.304265
F	-6.555268	-1.939427	-1.783238

Product

C	2.673441	-4.184975	0.867893
C	2.066981	-4.489856	2.209354
C	1.172026	-3.621927	2.688733
C	0.845978	-2.434609	1.822165
C	1.579492	-3.788256	-0.139811
H	0.683820	-3.753733	3.655712
H	2.355172	-5.387993	2.758301
H	3.390785	-3.352064	0.977429
H	3.244848	-5.029955	0.462816

H	1.045938	-4.695524	-0.456657
H	2.047478	-3.365826	-1.043240
H	1.690003	-1.721124	1.829344
H	-0.032707	-1.879774	2.178600
N	0.589929	-2.823036	0.410846
H	-0.308651	-3.305849	0.419750
C	4.999587	1.058396	-0.373304
C	4.048905	2.071577	-0.347341
C	4.540777	-0.256861	-0.376734
H	6.063670	1.285199	-0.393748
C	2.676395	1.817781	-0.317632
C	4.484717	3.513462	-0.374777
C	3.185379	-0.537402	-0.362896
C	5.498876	-1.413368	-0.347446
C	2.229885	0.490891	-0.323608
H	1.966958	2.639852	-0.289522
F	5.807587	3.638462	-0.203798
F	4.176269	4.094009	-1.542062
F	3.882575	4.225837	0.585775
H	2.854197	-1.575229	-0.387962
F	6.705729	-1.085775	-0.814194
F	5.667128	-1.877207	0.903876
F	5.042594	-2.450315	-1.073073
N	0.905119	0.046451	-0.284386
C	-0.288775	0.708378	-0.219849
H	0.829396	-0.981144	-0.229827
S	-0.516380	2.355030	-0.249001
N	-1.314367	-0.216426	-0.138290
H	-1.042629	-1.177164	-0.333841
C	-2.701922	0.008308	-0.137840
C	-3.503315	-0.881806	-0.856077
C	-3.307360	1.031424	0.604227
C	-4.890049	-0.756118	-0.830756
H	-3.044689	-1.674012	-1.450941
C	-4.691441	1.153601	0.590117
H	-2.701424	1.719871	1.186509
C	-5.501250	0.266903	-0.118394
C	-5.715971	-1.772051	-1.569727
C	-5.337363	2.285404	1.344534
H	-6.585163	0.370465	-0.108164
F	-5.176281	-2.071598	-2.760653
F	-5.798779	-2.924487	-0.885812
F	-6.964963	-1.345647	-1.781539
F	-5.550150	3.344267	0.552848
F	-4.579139	2.699972	2.366607
F	-6.529199	1.923731	1.841721

Cl_cat

R1_cat

C	4.529431	0.000040	0.571432
H	4.136061	0.000137	1.597195
H	5.624061	0.000052	0.462533
N	3.711866	-0.000069	-0.398258
H	4.217138	-0.000143	-1.293396
C	-2.807605	0.680363	0.133887
C	-2.807629	-0.680291	0.133906
C	-0.718249	-0.000004	-0.040557
N	-1.493301	1.088905	0.023940
N	-1.493339	-1.088881	0.023980
H	-3.623926	1.390974	0.201676
H	-3.623976	-1.390868	0.201732
C	-1.005711	-2.468727	-0.017792
H	-0.339373	-2.648411	0.834027
H	-1.872631	-3.133394	0.041132
H	-0.468540	-2.638979	-0.958138
C	-1.005614	2.468729	-0.017811
H	-0.339731	2.648559	0.834335
H	-0.467928	2.638793	-0.957898
H	-1.872549	3.133431	0.040512
Cl	0.967475	-0.000030	-0.184102

R1_cat_R2

C	4.794905	-0.252239	0.307046
C	4.030446	-1.310002	0.014178
C	2.831790	-1.700792	0.775737
C	1.994204	-0.842988	1.373118
C	2.769514	1.894976	-1.080862
H	2.640061	-2.776373	0.853627
H	4.318935	-1.963289	-0.814850
H	4.578958	0.378786	1.173153
H	5.676924	-0.010784	-0.288243
H	2.535265	0.922962	-1.537531
H	3.743169	2.338050	-1.339921
N	1.921316	2.423914	-0.299021
H	2.268373	3.320916	0.062170
H	2.129905	0.239023	1.289853
H	1.158651	-1.204784	1.977467
C	-3.158487	-1.671451	-0.320976
C	-3.897271	-0.645431	0.181338
C	-1.836668	0.079483	-0.114366
N	-1.871878	-1.202078	-0.497178
N	-3.055301	0.442012	0.304030
H	-3.433011	-2.691555	-0.565791
H	-4.942970	-0.593977	0.463817
C	-3.407101	1.769745	0.810089
H	-3.195726	2.522136	0.041356
H	-4.475706	1.767860	1.044484
H	-2.826956	1.983780	1.715416
C	-0.738135	-1.951015	-1.044920
H	-0.601838	-1.691127	-2.102014
H	0.166121	-1.705347	-0.473542
H	-0.962250	-3.017910	-0.948036
Cl	-0.472709	1.077234	-0.147560

TS

C	-4.473233	0.217707	-0.652005
C	-3.716352	1.260871	-0.147382
C	-2.854316	1.099506	0.953152
C	-2.746022	-0.124568	1.576646
C	-2.875164	-1.108866	-1.144095
H	-2.130918	1.887485	1.175318
H	-3.636939	2.178144	-0.737168
H	-4.848337	-0.563039	0.011218
H	-5.059180	0.372298	-1.559981
H	-2.356045	-0.410604	-1.808534
H	-3.608395	-1.763578	-1.627968
N	-2.245650	-1.478074	-0.038190
H	-2.723207	-2.283980	0.378921
H	-3.615497	-0.776165	1.659451
H	-1.961473	-0.303493	2.314407
C	3.574445	1.359723	-0.141863
C	4.069455	0.097577	-0.040072
C	1.866664	-0.033631	-0.035617
N	2.196135	1.260435	-0.136231
N	2.989512	-0.760453	0.024978
H	4.077321	2.317726	-0.216663
H	5.091020	-0.264782	-0.006395
C	3.037438	-2.218591	0.146331

H	2.506569	-2.673469	-0.697985
H	4.088315	-2.522447	0.132289
H	2.572830	-2.524797	1.090953
C	1.263785	2.382745	-0.248900
H	1.294738	2.780979	-1.270044
H	0.254999	2.025483	-0.018610
H	1.557063	3.156343	0.469252
Cl	0.296292	-0.669429	0.009439

Product

C	-3.799795	0.465276	-0.955261
C	-3.171110	1.371258	0.066971
C	-2.642701	0.808610	1.158654
C	-2.743351	-0.684655	1.312382
C	-2.885672	-0.743632	-1.189862
H	-2.207066	1.403141	1.965035
H	-3.172166	2.454112	-0.076869
H	-4.779857	0.121039	-0.580640
H	-3.984890	0.981036	-1.906456
H	-2.009093	-0.393219	-1.763653
H	-3.394094	-1.490822	-1.815155
N	-2.414340	-1.382554	0.053333
H	-2.783098	-2.329130	0.108238
H	-3.771160	-0.921546	1.651292
H	-2.063772	-1.047262	2.098274
C	3.334147	1.466506	-0.066753
C	3.930109	0.244343	-0.051136
C	1.746362	-0.062742	-0.030627
N	1.968548	1.257084	-0.052274
N	2.923158	-0.700575	-0.028879
H	3.758064	2.464593	-0.086273
H	4.977750	-0.035845	-0.052602
C	3.088109	-2.154751	0.001365
H	2.568458	-2.599463	-0.855207
H	4.158388	-2.373094	-0.059813
H	2.679305	-2.552647	0.937627
C	0.945919	2.305704	-0.082811
H	0.936736	2.772276	-1.075132
H	-0.032460	1.857962	0.127640
H	1.187497	3.050553	0.683495
Cl	0.222223	-0.804405	-0.004858

Br_cat

R1_cat

C	4.392095	-0.000350	0.660969
H	3.964042	-0.000922	1.672459
H	5.488922	-0.000448	0.584028
N	3.608324	0.000322	-0.336071
H	4.132400	0.000746	-1.219731
C	-3.052639	-0.680467	0.154028
C	-3.052677	0.680287	0.153979
C	-0.957317	-0.000031	-0.011336
N	-1.738227	-1.087163	0.050245
N	-1.738313	1.087076	0.050103
H	-3.869275	-1.391046	0.218271
H	-3.869386	1.390794	0.218117
C	-1.260775	2.469789	0.010671
H	-2.131792	3.128914	0.073760
H	-0.593092	2.652108	0.860967
H	-0.726843	2.646817	-0.930392
C	-1.260620	-2.469847	0.010368
H	-0.729854	-2.647658	-0.932344
H	-0.590029	-2.651260	0.858553
H	-2.131308	-3.129041	0.077101
Br	0.893866	0.000088	-0.162937

R1_cat_R2

C	-4.943395	0.384370	0.091519
C	-4.123770	1.392906	-0.225765
C	-3.018420	1.860356	0.628109
C	-2.271840	1.068694	1.408209
C	-2.872618	-1.899599	-0.934249
H	-2.821135	2.937683	0.614684
H	-4.294430	1.946168	-1.154429
H	-4.850962	-0.139633	1.046485
H	-5.752538	0.082746	-0.575692
H	-2.649849	-1.015772	-1.547201
H	-3.853579	-2.374000	-1.085516
N	-2.001205	-2.306746	-0.106219
H	-2.329028	-3.133365	0.407474
H	-2.414790	-0.015295	1.420523
H	-1.507200	1.485352	2.067714
C	3.971046	0.780713	0.069990
C	3.213307	1.853937	-0.283424
C	1.869673	0.113119	-0.074046
N	3.117097	-0.295922	0.196002
N	1.905633	1.420515	-0.365614
H	5.037325	0.690225	0.245457
H	3.488379	2.884177	-0.480599
C	0.753304	2.236911	-0.752003
H	0.995899	3.283897	-0.543929
H	0.549923	2.104543	-1.822062
H	-0.121372	1.931522	-0.164565
C	3.492577	-1.659579	0.570437
H	2.999180	-1.932312	1.510840
H	3.193790	-2.352980	-0.224428
H	4.578860	-1.686798	0.698532
Br	0.348048	-0.948916	-0.042147

TS

C	-4.640343	-0.019236	-0.734049
C	-3.963088	1.182255	-0.604030
C	-3.103771	1.443636	0.478621
C	-2.923453	0.500726	1.464097
C	-2.986933	-1.314888	-0.832154
H	-2.428187	2.299192	0.415872
H	-3.935283	1.859756	-1.461549
H	-4.975790	-0.559376	0.152619
H	-5.228649	-0.204532	-1.634782
H	-2.526847	-0.855917	-1.712696
H	-3.676436	-2.140783	-1.038047
N	-2.299948	-1.267617	0.303217
H	-2.684450	-1.931560	0.982683
H	-3.738392	-0.162717	1.751435
H	-2.133376	0.618050	2.208664
C	4.165479	0.053962	-0.201791
C	3.743927	1.346812	-0.242183
C	1.960781	0.055665	-0.025388
N	3.041635	-0.735704	-0.066793
N	2.368238	1.328342	-0.131388
H	5.161783	-0.370444	-0.257514
H	4.299714	2.272772	-0.340157
C	1.485306	2.493984	-0.121570
H	2.100518	3.385864	-0.274374
H	0.751459	2.404344	-0.931147
H	0.970141	2.557529	0.844488
C	3.015822	-2.195936	0.018877
H	2.581275	-2.499965	0.978430

H	2.419302	-2.601386	-0.806782
H	4.046227	-2.556670	-0.054109
Br	0.191576	-0.516320	0.149689

Product

C	-4.038223	0.152733	-1.114123
C	-3.528205	1.382515	-0.414457
C	-2.972817	1.224701	0.789845
C	-2.919759	-0.166423	1.359186
C	-2.993400	-0.965739	-1.017892
H	-2.605633	2.066522	1.379582
H	-3.627103	2.365338	-0.879022
H	-4.974983	-0.176661	-0.631423
H	-4.273479	0.343184	-2.168941
H	-2.167475	-0.723209	-1.708041
H	-3.423961	-1.919564	-1.353572
N	-2.432999	-1.126454	0.343361
H	-2.621326	-2.070191	0.677727
H	-3.930915	-0.443538	1.714379
H	-2.249563	-0.220979	2.229321
C	4.078557	0.091449	-0.219689
C	3.635282	1.377622	-0.210998
C	1.877435	0.050538	-0.012437
N	2.969676	-0.721113	-0.095002
N	2.261555	1.332637	-0.083515
H	5.080536	-0.314139	-0.306014
H	4.174348	2.315577	-0.286063
C	1.365179	2.486283	-0.001526
H	1.908908	3.364766	-0.362465
H	0.486016	2.307251	-0.630809
H	1.052392	2.639572	1.038765
C	2.967946	-2.183631	-0.064546
H	2.522450	-2.530002	0.875365
H	2.393964	-2.568751	-0.915592
H	4.005608	-2.524266	-0.131354
Br	0.109447	-0.543048	0.164116

I_cat

R1_cat

C	4.326147	0.000166	0.721068
H	3.896934	0.000475	1.731311
H	5.421335	0.000131	0.635144
N	3.544612	-0.000070	-0.278064
H	4.052698	-0.000308	-1.170406
C	-3.328210	0.680243	0.156264
C	-3.328198	-0.680189	0.156282
C	-1.219933	0.000050	0.011224
N	-2.012638	1.083021	0.065621
N	-2.012620	-1.082959	0.065697
H	-4.145372	1.390906	0.212174
H	-4.145362	-1.390848	0.212219
C	-1.549926	-2.469794	0.032389
H	-0.890438	-2.659673	0.887576
H	-2.427241	-3.121348	0.090758
H	-1.011674	-2.656925	-0.904393
C	-1.549868	2.469835	0.032310
H	-2.427117	3.121443	0.091104
H	-0.890043	2.659551	0.887270
H	-1.011960	2.657055	-0.904654
I	0.883824	-0.000043	-0.139587

R1_cat_R2

C	-5.265497	0.448164	-0.323980
C	-4.292372	1.151080	-0.916394
C	-3.247980	1.899422	-0.196876
C	-2.733787	1.546076	0.986595
C	-3.016852	-1.859900	-0.517708
H	-2.888950	2.811417	-0.684517
H	-4.277950	1.221026	-2.008800
H	-5.361724	0.419854	0.764531
H	-6.018516	-0.080059	-0.911343
H	-2.761733	-1.323333	-1.440241
H	-4.012192	-2.321345	-0.461550
N	-2.158756	-1.919208	0.415977
H	-2.498482	-2.441717	1.231447
H	-3.041867	0.626083	1.489877
H	-2.000754	2.174067	1.496119
C	3.593942	1.809956	-0.215444
C	4.215993	0.610839	-0.374496
C	2.055625	0.216773	-0.041527
N	2.255673	1.545140	-0.012626
N	3.246491	-0.363676	-0.263331
H	3.985353	2.821153	-0.230602
H	5.256915	0.369331	-0.559099
C	3.472726	-1.803830	-0.376080
H	2.904597	-2.202155	-1.225138
H	4.542701	-1.967718	-0.537372
H	3.157748	-2.299710	0.549695
C	1.223254	2.549757	0.237080
H	1.611151	3.523420	-0.078827
H	0.325847	2.302165	-0.341957
H	0.979203	2.574253	1.306697
I	0.201217	-0.763256	0.209319

TS

C	-4.756677	0.151795	-0.677980
C	-4.145072	1.328462	-0.271547
C	-3.313203	1.387029	0.862578
C	-3.098742	0.267507	1.626911
C	-3.097827	-0.987060	-1.089043
H	-2.685838	2.268501	1.006457
H	-4.141660	2.178789	-0.958555
H	-5.081656	-0.577264	0.066287
H	-5.334950	0.148592	-1.603925
H	-2.678425	-0.347728	-1.871338
H	-3.751870	-1.790903	-1.442553
N	-2.355584	-1.162082	0.003340
H	-2.648730	-1.986677	0.534556
H	-3.858201	-0.508450	1.712465
H	-2.320579	0.256094	2.392906
C	3.969804	1.365832	-0.146240
C	4.343582	0.065527	-0.010198
C	2.119140	0.133973	-0.034601
N	2.590328	1.386761	-0.158697
N	3.184787	-0.679995	0.056386
H	4.561697	2.270089	-0.234568
H	5.326325	-0.389446	0.044623
C	3.120090	-2.132367	0.206037
H	2.571552	-2.565825	-0.638554
H	4.143748	-2.519460	0.219168
H	2.616411	-2.386147	1.146430
C	1.763184	2.583130	-0.294159
H	2.425481	3.453819	-0.329484
H	1.177351	2.526308	-1.219478
H	1.090840	2.665734	0.568027
I	0.060870	-0.462109	0.005090

Product

C	-4.339334	0.273410	-1.000510
C	-3.981710	1.405247	-0.076991
C	-3.338867	1.102123	1.053089
C	-3.055176	-0.348419	1.324790
C	-3.125071	-0.645161	-1.172952
H	-3.052228	1.856495	1.786691
H	-4.241815	2.433554	-0.333162
H	-5.179599	-0.300757	-0.572655
H	-4.665265	0.628530	-1.986179
H	-2.381325	-0.124245	-1.796341
H	-3.405521	-1.567197	-1.699913
N	-2.478988	-1.004471	0.119615
H	-2.547503	-2.014079	0.252259
H	-3.994079	-0.854982	1.613853
H	-2.346566	-0.479572	2.154302
C	3.965070	1.320736	-0.115891
C	4.312368	0.006556	-0.068958
C	2.092381	0.125053	-0.028427
N	2.586911	1.372775	-0.089685
N	3.139144	-0.716959	-0.015265
H	4.575293	2.215764	-0.165051
H	5.285520	-0.471852	-0.068869
C	3.044282	-2.174207	0.046885
H	2.491241	-2.545906	-0.823893
H	4.059887	-2.582060	0.040331
H	2.532337	-2.472254	0.969616
C	1.783638	2.593413	-0.127028
H	2.465676	3.448975	-0.152692
H	1.153895	2.594765	-1.024709
H	1.154398	2.648798	0.769163
I	0.023032	-0.413696	0.036426

S2_rigid_cat

R1_cat

C	1.224036	-1.195168	-0.031983
C	-0.747723	0.116440	0.079121
C	-1.324711	-1.129682	-0.034142
C	-3.208057	0.150516	0.095790
C	-2.732784	-1.136036	-0.028198
H	-3.367501	-2.015210	-0.107906
C	0.713473	0.079221	0.084938
C	2.629738	-1.276289	-0.014813
H	3.217788	-2.187344	-0.094733
C	3.169805	-0.016791	0.120389
C	-4.571720	0.563013	0.138372
C	4.553578	0.321370	0.174055
N	-5.675784	0.913609	0.174735
N	5.674864	0.611330	0.217072
S	-1.941576	1.345773	0.193252
S	1.968671	1.244355	0.227496
S	-0.082450	-2.424709	-0.158283
O	-0.098629	-3.021236	-1.497025
O	-0.103164	-3.273330	1.036372
C	0.502230	4.166958	-0.881615
H	1.097830	3.659761	-1.653450
H	0.332359	5.246324	-1.012036
N	0.064346	3.484840	0.093975
H	-0.471622	4.076528	0.741964

R1_cat_R2

C	1.843473	-1.098409	0.156288
C	-0.251968	-0.410582	-0.713410
C	-0.618079	-1.652178	-0.241778
C	-2.620640	-0.941374	-1.070394
C	-1.975571	-1.973232	-0.421459
H	-2.464911	-2.889786	-0.100835
C	1.151286	-0.091623	-0.480303
C	3.218549	-0.849731	0.329752
H	3.933480	-1.513713	0.809452
C	3.544292	0.380231	-0.200985
C	-4.004812	-0.865878	-1.400725
C	4.828133	0.999386	-0.203939
N	-5.131802	-0.779310	-1.658152
N	5.866029	1.515032	-0.213555
S	-1.574265	0.401913	-1.434915
S	2.174870	1.224860	-0.873656
S	0.759935	-2.474495	0.568235
O	1.158883	-3.687750	-0.149201
O	0.524051	-2.515031	2.018529
C	-1.120763	0.577266	2.158454
C	-2.451047	0.588223	2.015739
C	-3.226429	1.725265	1.489976
C	-2.938801	3.013479	1.717389
C	0.290403	4.035051	-0.214700
H	-4.113466	1.477156	0.895743
H	-3.023147	-0.307365	2.279037
H	-0.514316	1.433536	1.844439
H	-0.598871	-0.298416	2.551989
H	1.349028	4.171362	-0.480221
H	-0.221461	4.896580	0.242400
N	-0.261164	2.916402	-0.442694
H	-1.243100	2.926613	-0.129794
H	-2.105574	3.296908	2.366556
H	-3.549360	3.812700	1.293366

TS

C	0.556750	2.087867	0.178047
C	1.085103	-0.168649	-0.322576
C	2.303959	0.225621	0.183319
C	2.809922	-1.917175	-0.418304
C	3.307910	-0.761544	0.142529
H	4.330506	-0.648028	0.494168
C	0.085843	0.894448	-0.322718
C	-0.363025	3.153632	0.128913
H	-0.184804	4.168136	0.477026
C	-1.545875	2.727485	-0.434335
C	3.509103	-3.142353	-0.622544
C	-2.724463	3.501062	-0.644922
N	4.065127	-4.144758	-0.793166
N	-3.690969	4.115636	-0.821980
S	1.131548	-1.791843	-0.877146
S	-1.530434	1.040206	-0.879265
S	2.269778	1.946170	0.714268
O	3.139472	2.760146	-0.139026
O	2.360045	2.034216	2.174252
C	-1.795426	-1.674483	1.436366
C	-2.524568	-2.792945	1.790641
C	-3.855155	-2.939465	1.352795
C	-4.478645	-1.979598	0.582075
C	-3.082156	-1.890513	-1.118205
H	-4.326699	-3.920360	1.456467
H	-2.009108	-3.660552	2.207082
H	-2.277279	-0.705792	1.309319
H	-0.724102	-1.629532	1.647850
H	-3.516232	-0.935939	-1.433166
H	-3.514481	-2.788004	-1.569400
N	-1.840784	-1.878177	-0.660159

H	-1.487813	-2.838600	-0.608740
H	-4.218043	-0.927906	0.701664
H	-5.471630	-2.170081	0.170878

Product

C	1.503452	1.745420	0.120681
C	0.976055	-0.541759	-0.204578
C	2.310764	-0.676696	0.105868
C	1.789179	-2.861271	-0.304374
C	2.798811	-1.997378	0.058083
H	3.818437	-2.309524	0.270665
C	0.512849	0.841461	-0.193454
C	1.101171	3.094878	0.094314
H	1.730804	3.953927	0.312931
C	-0.228489	3.180865	-0.255664
C	1.879869	-4.275575	-0.454302
C	-1.006079	4.368806	-0.382626
N	1.938746	-5.426207	-0.579634
N	-1.650733	5.325926	-0.489930
S	0.264012	-2.057952	-0.576214
S	-0.973845	1.627322	-0.534192
S	3.069731	0.920436	0.447387
O	4.047884	1.251026	-0.592475
O	3.421693	1.028767	1.865704
C	-2.626833	-0.966394	1.220325
C	-3.993690	-1.366027	1.714786
C	-5.061595	-1.397115	0.914276
C	-4.993972	-0.979585	-0.531492
C	-3.693891	-0.227830	-0.814008
H	-6.026943	-1.724356	1.308176
H	-4.085867	-1.638113	2.769377
H	-2.349648	0.019647	1.637656
H	-1.868697	-1.675042	1.589946
H	-3.749817	0.777307	-0.363964
H	-3.536542	-0.095077	-1.894273
N	-2.517058	-0.895394	-0.241192
H	-2.516381	-1.855691	-0.594824
H	-5.857262	-0.342677	-0.780326
H	-5.075346	-1.866719	-1.184627

S2_flex_cat

R1_cat

C	-0.337875	3.393330	0.878506
H	0.077297	4.293573	1.357737
H	-1.434224	3.322588	0.823751
N	0.353123	2.448192	0.393144
H	1.357702	2.637801	0.510186
S	-1.757263	0.483029	-0.421909
C	-3.108697	-0.564279	-0.119557
C	-0.729054	-0.749911	0.226242
C	-1.493492	-1.822858	0.624529
H	-1.080160	-2.737670	1.046633
C	0.722071	-0.645780	0.314127
C	1.510348	-0.916670	1.408102
S	1.725017	-0.210816	-1.024150
H	1.118725	-1.200102	2.383604
C	3.108914	-0.419698	0.005042
N	-2.837767	-1.713115	0.438771
N	2.855195	-0.786988	1.231325
C	4.429138	-0.181464	-0.503271
N	5.478332	0.024239	-0.947063
C	-4.437555	-0.133235	-0.449356
N	-5.492623	0.252215	-0.729198

R1_cat_R2

C	-3.368550	3.557514	-0.325904
C	-3.710608	2.395039	0.243697
C	-3.785718	1.104677	-0.464811
C	-2.987473	0.747743	-1.478337
C	0.059705	3.064209	-0.017845
H	-4.544082	0.400120	-0.108844
H	-3.985847	2.385085	1.303361
H	-3.155926	3.620608	-1.396347
H	-3.326372	4.480665	0.254632
H	-0.660262	3.891097	-0.133442
H	1.120846	3.293579	-0.197932
H	-2.181492	1.400726	-1.826102
H	-3.107801	-0.214904	-1.977861
N	-0.263810	1.882471	0.304947
H	-1.283868	1.818252	0.432773
S	2.356536	0.544025	0.427593
C	3.938945	-0.006896	-0.027775
C	1.716901	-0.923958	-0.232712
C	2.748067	-1.682526	-0.742874
H	2.611308	-2.666367	-1.189429
C	0.311315	-1.308597	-0.239239
C	-0.353952	-1.944154	-1.264807
S	-0.741358	-1.170923	1.123690
H	0.085072	-2.143853	-2.241076
C	-1.971447	-1.968634	0.199105
N	3.999910	-1.160280	-0.637551
N	-1.640389	-2.312726	-1.016026
C	-3.284535	-2.147906	0.746946
N	-4.339736	-2.255753	1.211643
C	5.086618	0.810859	0.244891
N	5.985450	1.500735	0.482376

TS

C	0.706399	4.149950	-0.465294
C	-0.635147	4.270601	-0.757661
C	-1.434328	3.152081	-1.064441
C	-0.895958	1.878859	-1.056079
C	0.582419	2.719722	1.237526
H	-2.519719	3.269794	-1.073202
H	-1.136604	5.219909	-0.551003
H	1.296699	3.354012	-0.918674
H	1.273568	5.017971	-0.124496
H	0.326726	3.517105	1.941765
H	1.649777	2.502929	1.119748
H	0.137209	1.695356	-1.352437
H	-1.558706	1.014818	-1.147506
N	-0.271967	1.761673	0.928654
H	-1.203781	1.997901	1.283093
S	1.842361	-0.334177	0.537353
C	3.236228	-1.205725	-0.026083
C	0.854989	-1.494380	-0.287353
C	1.660559	-2.420219	-0.913105
H	1.281399	-3.270268	-1.478463
C	-0.600296	-1.469188	-0.323401
C	-1.395070	-1.757324	-1.412316
S	-1.600962	-1.129936	1.041896
H	-1.007776	-1.986078	-2.404112
C	-2.988550	-1.394868	0.034321
N	3.003509	-2.251412	-0.771908
N	-2.739189	-1.708882	-1.209348
C	-4.309375	-1.240787	0.572296
N	-5.358569	-1.104714	1.042130
C	4.551848	-0.749327	0.320999

N	5.594288	-0.345809	0.622021

Product

C	-1.362156	3.576676	0.096781
C	-0.204484	4.179726	0.843064
C	0.821952	3.374917	1.128765
C	0.708293	1.937776	0.686501
C	-0.844634	2.743492	-1.084852
H	1.709041	3.722325	1.661971
H	-0.209024	5.235991	1.118752
H	-1.933397	2.915504	0.773224
H	-2.064346	4.336310	-0.270476
H	-0.453685	3.434359	-1.848465
H	-1.689203	2.200488	-1.543183
H	0.016637	1.402543	1.365213
H	1.672460	1.413183	0.756011
N	0.202540	1.781352	-0.692099
H	0.995515	1.910420	-1.317518
S	-1.740309	-0.473965	-0.561252
C	-3.047359	-1.459457	0.021795
C	-0.655437	-1.494925	0.323855
C	-1.373788	-2.467171	0.983883
H	-0.919719	-3.248886	1.591172
C	0.791506	-1.333651	0.359725
C	1.603435	-1.440538	1.467215
S	1.765107	-1.044433	-1.037467
H	1.232915	-1.605607	2.477875
C	3.167038	-1.080639	-0.013481
N	-2.724681	-2.440223	0.821258
N	2.939515	-1.292700	1.254983
C	4.471535	-0.863910	-0.569119
N	5.506790	-0.681990	-1.054134
C	-4.397287	-1.153002	-0.357685
N	-5.470685	-0.871871	-0.687688

Se_cat

R1_cat

C	-3.158854	3.633024	0.607532
H	-4.090233	4.220740	0.614724
H	-2.509911	3.718803	1.490099
N	-2.792588	2.876657	-0.340375
H	-3.475241	2.878990	-1.108554
Se	-0.118405	1.701843	-0.379045
C	1.495603	0.653379	-0.308151
C	-1.259068	0.220360	0.024934
C	1.696211	-0.410625	-1.185764
C	2.503295	0.949012	0.605140
C	-1.088721	-0.517109	1.193988
C	-2.292416	-0.139174	-0.835703
C	2.861729	-1.164782	-1.155759
F	0.757032	-0.738206	-2.069571
C	3.683868	0.215453	0.640463
F	2.360891	1.946252	1.471877
C	-1.916307	-1.591455	1.498319
F	-0.117627	-0.210538	2.050572
C	-3.140169	-1.198671	-0.537928
F	-2.500244	0.527545	-1.966057
C	3.859314	-0.843619	-0.241435
F	3.036044	-2.180522	-1.994816
F	4.639151	0.512339	1.515221
C	-2.947233	-1.927762	0.629730
F	-1.736986	-2.288264	2.615719
F	-4.128190	-1.526100	-1.365147
F	4.979980	-1.551752	-0.210926
F	-3.749413	-2.944752	0.915539

R1_cat_R2

C	-3.130776	-4.048419	-0.688686
C	-4.233160	-4.042345	0.070484
C	-4.573826	-3.019026	1.075404
C	-3.702050	-2.183592	1.651708
C	-3.624884	0.284906	-1.730536
H	-5.628639	-2.960803	1.359969
H	-4.962740	-4.847351	-0.058501
H	-2.384351	-3.252191	-0.621902
H	-2.945283	-4.853225	-1.400952
H	-4.656780	0.343155	-2.110154
H	-2.985085	1.164882	-1.900161
H	-2.632290	-2.219480	1.429130
H	-4.032706	-1.430895	2.368549
N	-3.139427	-0.712835	-1.119482
H	-3.832115	-1.466034	-1.015444
Se	-0.258968	-1.079956	-0.500646
C	1.597041	-0.888249	-0.027932
C	-0.646045	0.777986	-0.271185
C	1.966714	-0.416512	1.230594
C	2.605743	-1.222789	-0.926730
C	-0.076626	1.728764	-1.114641
C	-1.523013	1.210034	0.720030
C	3.300768	-0.274212	1.587568
F	1.036018	-0.073450	2.118035
C	3.947681	-1.101620	-0.582174
F	2.309132	-1.669636	-2.142031
C	-0.378211	3.080001	-0.985536
F	0.779800	1.362689	-2.062855
C	-1.845532	2.554879	0.854133
F	-2.080408	0.347059	1.558527
C	4.291821	-0.624443	0.676397
F	3.638631	0.184113	2.788056
F	4.900628	-1.427991	-1.448496
C	-1.269803	3.488814	-0.000640
F	0.173120	3.978547	-1.794403
F	-2.690499	2.957859	1.797319
F	5.568780	-0.499514	1.010171
F	-1.567887	4.774236	0.128818

TS

C	-4.934406	-3.106615	-0.952686
C	-5.555522	-2.630401	0.179385
C	-4.835907	-2.141697	1.289981
C	-3.454081	-2.083569	1.258816
C	-3.644636	-1.279850	-1.442540
H	-5.375333	-1.589608	2.062151
H	-6.632657	-2.443908	0.146508
H	-3.949682	-3.568353	-0.890668
H	-5.524176	-3.370683	-1.832265
H	-4.480609	-0.823069	-1.980153
H	-2.966219	-1.911310	-2.024475
H	-2.873016	-2.833511	0.720331
H	-2.904692	-1.565922	2.047309
N	-3.194323	-0.791843	-0.305908
H	-3.837398	-0.086804	0.067442
Se	-0.310491	-1.096619	0.015821
C	1.604004	-0.986780	0.180606
C	-0.544586	0.792378	-0.158607
C	2.189914	-0.319740	1.254986
C	2.440766	-1.580739	-0.759811
C	0.085934	1.499431	-1.179312
C	-1.360595	1.494576	0.723430

C	3.569395	-0.241247	1.392677
F	1.430205	0.275264	2.170758
C	3.824087	-1.525298	-0.631320
F	1.935041	-2.217825	-1.810017
C	-0.081401	2.872207	-1.317055
F	0.873667	0.871679	-2.048186
C	-1.553150	2.863884	0.589707
F	-1.987047	0.870726	1.717178
C	4.385635	-0.852236	0.446627
F	4.114777	0.402994	2.418833
F	4.611006	-2.099934	-1.534456
C	-0.907476	3.552658	-0.430768
F	0.530303	3.533558	-2.293521
F	-2.341896	3.520798	1.434163
F	5.703737	-0.789387	0.573236
F	-1.081697	4.860940	-0.558813

Product

C	-4.987231	-1.911584	-1.307782
C	-5.534104	-2.539100	-0.051565
C	-4.835710	-2.585173	1.085644
C	-3.466164	-1.969219	1.216967
C	-3.480894	-1.681169	-1.183270
H	-5.250713	-3.082176	1.966553
H	-6.535885	-2.974422	-0.091603
H	-5.200548	-2.556655	-2.174857
H	-5.506872	-0.957071	-1.506637
H	-3.106780	-1.035737	-1.991650
H	-2.950017	-2.644205	-1.266961
H	-2.703654	-2.766514	1.299537
H	-3.389836	-1.385557	2.146529
N	-3.099494	-1.094400	0.102909
H	-3.601011	-0.210451	0.217993
Se	-0.250467	-1.172022	0.190243
C	1.666819	-0.952999	0.232765
C	-0.579616	0.690963	-0.088306
C	2.273640	-0.164862	1.208724
C	2.483425	-1.587595	-0.698306
C	-0.044151	1.355626	-1.188938
C	-1.361446	1.420085	0.803587
C	3.652123	-0.005643	1.258548
F	1.533165	0.472312	2.112969
C	3.866776	-1.453017	-0.656514
F	1.957343	-2.342222	-1.657409
C	-0.271949	2.711204	-1.396731
F	0.710177	0.704064	-2.069706
C	-1.613865	2.771268	0.602213
F	-1.898067	0.838222	1.872714
C	4.448426	-0.658604	0.323707
F	4.215959	0.755112	2.191075
F	4.634199	-2.067953	-1.550258
C	-1.062732	3.417374	-0.498827
F	0.249449	3.330809	-2.449965
F	-2.370007	3.452278	1.457377
F	5.766494	-0.520478	0.367820
F	-1.294538	4.708634	-0.692286

Te_cat

R1_cat

C	-3.203445	3.517491	0.885520
H	-4.209472	3.960984	0.930931
H	-2.540238	3.685091	1.744778
N	-2.771056	2.832606	-0.088676
H	-3.468372	2.733431	-0.836320
Te	-0.143130	1.767703	-0.271213
C	1.614697	0.532071	-0.275917
C	-1.361649	0.067831	0.087330
C	1.697945	-0.622053	-1.048773
C	2.722609	0.869899	0.492267
C	-1.095283	-0.797873	1.143895
C	-2.444516	-0.229008	-0.733385
C	2.831799	-1.425163	-1.047983
F	0.672040	-0.999352	-1.814729
C	3.874931	0.092426	0.503006
F	2.709102	1.969983	1.248186
C	-1.873212	-1.926870	1.375779
F	-0.075432	-0.569108	1.969477
C	-3.244845	-1.342804	-0.513126
F	-2.753664	0.557709	-1.766863
C	3.924703	-1.061067	-0.269724
F	2.885914	-2.528801	-1.788031
F	4.922995	0.438087	1.244938
C	-2.953057	-2.196141	0.544304
F	-1.599863	-2.741453	2.389756
F	-4.279644	-1.602401	-1.307180
F	5.016888	-1.814994	-0.267195
F	-3.707874	-3.265305	0.761116

R1_cat_R2

C	-5.737396	-2.752669	-0.073704
C	-5.773355	-1.906214	0.962777
C	-4.600978	-1.499884	1.757649
C	-3.515340	-2.256193	1.956922
C	-3.480972	-1.128132	-2.337914
H	-4.636677	-0.500534	2.201974
H	-6.730065	-1.456370	1.245000
H	-4.796045	-3.193701	-0.413510
H	-6.647283	-3.033033	-0.606187
H	-4.569621	-1.142868	-2.502430
H	-2.832405	-1.237772	-3.217782
H	-3.456944	-3.278601	1.574276
H	-2.659699	-1.875824	2.515973
N	-2.947512	-0.996716	-1.196139
H	-3.646117	-0.912940	-0.446176
Te	-0.249303	-1.262053	-0.490457
C	1.803201	-0.962408	0.088427
C	-0.578363	0.831941	-0.389497
C	2.141174	-0.231965	1.223456
C	2.843836	-1.499129	-0.660329
C	0.221825	1.716464	-1.106341
C	-1.585913	1.370804	0.402730
C	3.463162	-0.024559	1.597164
F	1.189178	0.305966	1.990024
C	4.175576	-1.318500	-0.304398
F	2.587527	-2.216224	-1.756380
C	0.032159	3.092314	-1.036629
F	1.203493	1.266353	-1.885678
C	-1.803776	2.740368	0.478581
F	-2.385959	0.578406	1.121181
C	4.482416	-0.574967	0.828314
F	3.762898	0.684139	2.682225
F	5.154141	-1.843511	-1.036281
C	-0.987061	3.603028	-0.242701
F	0.808146	3.919496	-1.729574
F	-2.778270	3.231387	1.239439
F	5.749420	-0.391253	1.178420
F	-1.182794	4.913421	-0.174647

TS

C	-5.153921	-2.884051	-0.831080
C	-5.775614	-2.231708	0.212777
C	-5.054790	-1.726111	1.313195
C	-3.681788	-1.857344	1.369818
C	-3.717474	-1.315896	-1.453179
H	-5.553088	-1.030996	1.991846
H	-6.815823	-1.917344	0.091821
H	-4.250724	-3.467048	-0.652933
H	-5.725490	-3.165877	-1.717237
H	-4.489362	-0.780623	-2.013330
H	-3.134688	-2.057208	-2.008671
H	-3.180390	-2.724425	0.938807
H	-3.103390	-1.321578	2.125036
N	-3.161768	-0.796144	-0.373336
H	-3.671089	0.033547	-0.057344
Te	-0.404066	-1.182476	0.007947
C	1.728458	-1.000500	0.217737
C	-0.568909	0.919933	-0.215443
C	2.297436	-0.133949	1.145978
C	2.591647	-1.751497	-0.571386
C	0.185587	1.601785	-1.165619
C	-1.414442	1.668499	0.595684
C	3.674150	-0.002687	1.279783
F	1.523727	0.613151	1.936747
C	3.972917	-1.649787	-0.451400
F	2.108978	-2.604889	-1.476695
C	0.108585	2.983326	-1.303164
F	1.013386	0.944281	-1.975089
C	-1.519910	3.047678	0.467605
F	-2.167880	1.077218	1.527826
C	4.512358	-0.768536	0.477428
F	4.196461	0.837109	2.169034
F	4.779525	-2.379853	-1.216218
C	-0.750329	3.705820	-0.484319
F	0.839313	3.615019	-2.215631
F	-2.342963	3.740864	1.248859
F	5.829133	-0.658828	0.600395
F	-0.839224	5.022984	-0.613048

Product

C	5.077563	-0.865793	0.956906
C	5.593366	-2.154486	0.372427
C	4.808200	-2.995011	-0.304836
C	3.357330	-2.698728	-0.582761
C	3.550171	-0.854314	0.974681
H	5.206767	-3.939135	-0.684476
H	6.647611	-2.399303	0.522922
H	5.464401	-0.733495	1.979356
H	5.461353	-0.009253	0.374626
H	3.161327	0.150678	1.196022
H	3.177633	-1.530984	1.761177
H	2.714505	-3.370769	0.016677
H	3.118251	-2.907482	-1.636785
N	2.971049	-1.314535	-0.293143
H	3.314663	-0.717253	-1.049482
Te	0.226491	-1.337959	-0.333643
C	-1.881030	-0.868588	-0.256726
C	0.666717	0.710846	0.006311
C	-2.434579	0.117249	-1.067302
C	-2.737878	-1.546557	0.601806
C	0.182850	1.365813	1.135192
C	1.449338	1.445063	-0.878337
C	-3.787097	0.432261	-1.022844
F	-1.665962	0.805155	-1.915757
C	-4.097394	-1.261207	0.660996
F	-2.271571	-2.507068	1.402891
C	0.466297	2.705348	1.379979
F	-0.572157	0.721828	2.022186
C	1.758686	2.780085	-0.649328
F	1.948191	0.878708	-1.981592
C	-4.619675	-0.265531	-0.155197
F	-4.293769	1.383248	-1.803092
F	-4.898552	-1.925170	1.489590
C	1.260040	3.411297	0.484442
F	-0.004855	3.310820	2.464669
F	2.516918	3.458394	-1.505037
F	-5.915364	0.019039	-0.108229
F	1.543920	4.686834	0.710923

P_cat

R1_cat

C	0.563693	-2.925045	3.300828
H	0.791029	-3.842616	3.865623
H	0.066777	-2.110328	3.845247
N	0.831423	-2.751335	2.074242
H	1.284676	-3.580649	1.669806
C	-1.063420	-1.132224	0.192349
C	-0.570329	1.544711	0.488823
C	-1.076752	-1.113309	-1.200135
C	-2.001497	-1.933496	0.834922
C	0.000964	2.357959	-0.487638
C	-1.779201	1.980705	1.034037
C	-1.966824	-1.879523	-1.936908
F	-0.202040	-0.353804	-1.859884
C	-2.908020	-2.710372	0.121350
F	-2.066760	-1.970023	2.164214
C	-0.585567	3.558711	-0.876332
F	1.134252	2.024010	-1.102458
C	-2.392248	3.166908	0.659091
F	-2.394825	1.219495	1.945288
C	-2.886454	-2.681217	-1.266840
F	-1.952134	-1.857640	-3.264917
F	-3.794286	-3.471348	0.754853
C	-1.782918	3.964189	-0.302354
F	-0.010672	4.314818	-1.805061
F	-3.545510	3.540192	1.202106
F	-3.745546	-3.416900	-1.957384
F	-2.347309	5.103726	-0.676004
C	1.704611	-0.243278	0.433701
C	2.167054	-1.327165	-0.306640
C	2.644691	0.727545	0.772797
C	3.496189	-1.427875	-0.704357
C	3.973725	0.658035	0.386479
C	4.402092	-0.434949	-0.357314
F	1.359084	-2.323624	-0.672826
F	3.903864	-2.476763	-1.412751
F	5.669626	-0.528507	-0.734817
F	4.830933	1.616167	0.719951
F	2.248858	1.790277	1.481078
P	0.040733	-0.066641	1.223468

R1_cat_R2

C	-5.289424	0.724507	-0.595037
C	-4.632915	0.708809	0.724853
C	-2.491518	0.835206	-3.181465
H	-4.153606	1.637572	1.050556
H	-5.785080	1.658629	-0.880409

H	-3.547534	0.788495	-3.493662
H	-1.729359	0.656265	-3.952577
N	-2.115726	1.064967	-1.993782
H	-2.930987	1.182431	-1.376482
C	-0.690777	-0.957702	-0.210260
C	2.035233	-0.785551	-0.497124
C	-0.674801	-0.958706	1.183166
C	-1.624227	-1.774204	-0.842730
C	2.898945	-0.306186	0.485914
C	2.344074	-2.032621	-1.043356
C	-1.568071	-1.710583	1.928598
F	0.197858	-0.191254	1.835662
C	-2.531860	-2.537180	-0.116372
F	-1.680770	-1.849373	-2.169681
C	4.026631	-1.018896	0.881934
F	2.685580	0.855615	1.101243
C	3.455239	-2.769653	-0.661603
F	1.530115	-2.561227	-1.963221
C	-2.501416	-2.502571	1.269288
F	-1.578323	-1.648877	3.256364
F	-3.455781	-3.266568	-0.739587
C	4.305654	-2.251649	0.307592
F	4.833338	-0.529992	1.817531
F	3.707994	-3.954488	-1.206436
F	-3.394818	-3.187863	1.969860
F	5.374900	-2.936224	0.688415
P	0.507570	0.003427	-1.243682
C	0.526284	1.679555	-0.456057
C	-0.487549	2.269616	0.292170
C	1.594057	2.501030	-0.810485
C	-0.435615	3.604551	0.676183
C	1.679051	3.833143	-0.436819
C	0.649122	4.390101	0.311488
F	-1.564228	1.583453	0.686975
F	-1.426915	4.131378	1.390271
F	0.702962	5.663560	0.677013
F	2.725022	4.572747	-0.787682
F	2.598918	1.982030	-1.524055
C	-5.308108	-0.307879	-1.447328
H	-5.849648	-0.246559	-2.392997
H	-4.786145	-1.243551	-1.227068
C	-4.622529	-0.351315	1.539477
H	-5.141562	-1.276187	1.271965
H	-4.115089	-0.320457	2.506156

TS

C	-5.693582	0.160708	-1.824314
C	-5.279969	0.187581	-0.475857
C	-2.978410	0.483466	-2.534033
H	-5.745104	0.904050	0.204042
H	-6.484107	0.850216	-2.133185
H	-3.299950	1.335765	-3.140289
H	-2.492685	-0.342829	-3.060203
N	-2.744008	0.597853	-1.240223
H	-3.089159	1.502410	-0.902572
C	-0.485320	-1.179271	0.181348
C	2.019156	-0.418431	-0.677248
C	-0.127798	-1.227022	1.526885
C	-1.390310	-2.129483	-0.274066
C	2.952215	0.241403	0.120226
C	2.465012	-1.560394	-1.345082
C	-0.659309	-2.167205	2.396226
F	0.726029	-0.327814	2.015848
C	-1.946524	-3.078896	0.575198
F	-1.783295	-2.142414	-1.548914
C	4.268135	-0.198467	0.224433
F	2.628812	1.322077	0.828057
C	3.767679	-2.027652	-1.252068
F	1.604376	-2.254670	-2.096254
C	-1.576613	-3.097035	1.913149
F	-0.313285	-2.185540	3.678187
F	-2.845267	-3.943910	0.116750
C	4.676688	-1.333883	-0.462500
F	5.134509	0.456934	0.988768
F	4.147527	-3.121485	-1.902492
F	-2.099836	-3.994669	2.734428
F	5.927429	-1.759165	-0.359109
P	0.233505	0.010209	-1.035012
C	0.057286	1.649467	-0.192417
C	-0.853287	1.994227	0.802462
C	0.809142	2.689282	-0.736880
C	-0.997118	3.307272	1.240222
C	0.693041	4.004882	-0.317594
C	-0.223993	4.314875	0.679882
F	-1.635568	1.085258	1.388399
F	-1.876056	3.600785	2.193194
F	-0.358716	5.566240	1.095412
F	1.441865	4.959820	-0.856153
F	1.705044	2.407361	-1.687938
C	-4.983851	-0.509940	-2.795978
H	-5.272808	-0.419539	-3.844620
H	-4.397821	-1.393853	-2.544810
C	-4.117405	-0.456675	-0.098661
H	-3.810398	-1.369492	-0.607527
H	-3.703781	-0.316972	0.902249

Product

C	-5.455417	0.072744	-2.166662
C	-5.251085	0.017812	-0.847212
C	-2.968233	0.222407	-2.596151
H	-6.022496	0.289017	-0.123675
H	-6.407193	0.397907	-2.591517
H	-2.825575	1.223956	-3.028007
H	-2.158392	-0.412978	-2.986169
N	-2.826792	0.334731	-1.131305
H	-2.958130	1.317736	-0.890415
C	-0.453812	-1.232837	0.248257
C	1.962903	-0.315849	-0.689876
C	-0.068605	-1.248380	1.586430
C	-1.283945	-2.256713	-0.192377
C	2.884027	0.391591	0.080172
C	2.449736	-1.429223	-1.377029
C	-0.511101	-2.223507	2.467282
F	0.725290	-0.286378	2.057526
C	-1.752382	-3.241247	0.669845
F	-1.679251	-2.310990	-1.464125
C	4.225339	0.025934	0.138526
F	2.525365	1.450606	0.804556
C	3.778770	-1.823266	-1.329978
F	1.604147	-2.169356	-2.101502
C	-1.360946	-3.223338	2.001999
F	-0.142831	-2.209993	3.743257
F	-2.580969	-4.182328	0.229592
C	4.673574	-1.082412	-0.567400
F	5.078862	0.726214	0.877375
F	4.197143	-2.892442	-1.997718
F	-1.800592	-4.155292	2.834728
F	5.948975	-1.437468	-0.507932

P	0.140275	0.006649	-0.984453
C	-0.090891	1.632176	-0.126723
C	-0.982696	1.924371	0.901312
C	0.589217	2.712802	-0.685840
C	-1.175043	3.223843	1.359854
C	0.423309	4.017329	-0.248102
C	-0.471538	4.273190	0.784117
F	-1.703051	0.970956	1.497659
F	-2.034037	3.464770	2.344817
F	-0.652511	5.512764	1.216762
F	1.104162	5.012902	-0.802728
F	1.464171	2.485474	-1.670179
C	-4.325794	-0.354325	-3.062431
H	-4.502072	-0.085388	-4.112288
H	-4.255257	-1.455627	-3.029460
C	-3.874185	-0.391630	-0.387203
H	-3.729442	-1.472575	-0.560513
H	-3.719083	-0.208680	0.684332

As_cat

R1_cat

C	-0.303517	-3.186013	-3.154622
H	-0.441042	-4.201543	-3.556419
H	0.177541	-2.447865	-3.810609
N	-0.657297	-2.821176	-1.993705
H	-1.083084	-3.593920	-1.466670
C	1.167475	-1.129548	-0.137015
C	0.527970	1.637087	-0.472384
C	1.127052	-1.062670	1.251425
C	2.154757	-1.916540	-0.716352
C	-0.108546	2.404570	0.498088
C	1.731963	2.132696	-0.967972
C	2.020388	-1.766810	2.045991
F	0.199754	-0.318723	1.856532
C	3.064839	-2.633083	0.053128
F	2.260568	-2.001462	-2.043797
C	0.415145	3.618067	0.934079
F	-1.248271	2.011161	1.066784
C	2.284660	3.334998	-0.550957
F	2.406781	1.416834	-1.877692
C	2.993289	-2.554508	1.438131
F	1.957253	-1.699792	3.371146
F	3.999166	-3.383512	-0.521610
C	1.612677	4.084026	0.407511
F	-0.218520	4.330720	1.859531
F	3.437241	3.769064	-1.049488
F	3.854619	-3.231555	2.184387
F	2.117697	5.236970	0.824001
C	-1.759487	-0.334882	-0.420916
C	-2.155159	-1.414013	0.359312
C	-2.736064	0.602258	-0.738976
C	-3.462142	-1.546720	0.816321
C	-4.046516	0.502563	-0.297838
C	-4.409705	-0.586752	0.485382
F	-1.295442	-2.375006	0.710575
F	-3.810252	-2.589844	1.563892
F	-5.657415	-0.708544	0.917172
F	-4.946506	1.427094	-0.612685
F	-2.396442	1.664046	-1.478844
As	-0.021481	-0.092238	-1.307076

R1_cat_R2

C	-5.300351	0.736402	-0.553684
C	-4.667941	0.719318	0.777887
C	-2.544948	0.868137	-3.175052
H	-4.190962	1.646412	1.111296
H	-5.782410	1.674105	-0.850504
H	-3.610902	0.877712	-3.452561
H	-1.813972	0.707508	-3.979629
N	-2.125286	1.016776	-1.989270
H	-2.912242	1.127420	-1.335234
C	-0.777430	-1.001448	-0.168979
C	2.069148	-0.827184	-0.464198
C	-0.717178	-0.965369	1.220603
C	-1.741262	-1.817157	-0.750291
C	2.922296	-0.321280	0.511649
C	2.367251	-2.096294	-0.954961
C	-1.592758	-1.691588	2.013209
F	0.179072	-0.187552	1.829410
C	-2.632824	-2.553694	0.021015
F	-1.847396	-1.911295	-2.075615
C	4.031393	-1.033752	0.957890
F	2.713903	0.868505	1.075996
C	3.460623	-2.835808	-0.527724
F	1.560180	-2.647434	-1.871665
C	-2.554707	-2.488789	1.404146
F	-1.558504	-1.598980	3.338850
F	-3.589365	-3.281122	-0.554149
C	4.301125	-2.292179	0.436038
F	4.830298	-0.521787	1.888489
F	3.706123	-4.044586	-1.022035
F	-3.434076	-3.145790	2.149077
F	5.354139	-2.976524	0.861676
C	0.490736	1.750375	-0.430551
C	-0.520376	2.313996	0.337246
C	1.569868	2.569318	-0.742671
C	-0.457304	3.629245	0.783001
C	1.667729	3.883522	-0.311363
C	0.638921	4.416714	0.456401
F	-1.603284	1.614683	0.694537
F	-1.444117	4.136172	1.517539
F	0.704794	5.672323	0.877769
F	2.722250	4.628639	-0.622912
F	2.573315	2.066141	-1.471155
C	-5.315541	-0.298944	-1.402716
H	-5.841740	-0.235476	-2.356942
H	-4.807828	-1.239776	-1.171352
C	-4.677010	-0.339991	1.593639
H	-5.194061	-1.263611	1.317613
H	-4.187028	-0.310296	2.569257
As	0.463497	-0.002584	-1.322805

TS

C	-5.736607	0.207209	-1.720517
C	-5.281464	0.287160	-0.387879
C	-3.056327	0.425365	-2.541337
H	-5.707204	1.047150	0.270176
H	-6.517305	0.902756	-2.040492
H	-3.384199	1.258845	-3.169817
H	-2.608272	-0.431258	-3.053159
N	-2.754911	0.590781	-1.265603
H	-3.060975	1.517626	-0.951929
C	-0.564564	-1.220745	0.218899
C	2.063368	-0.469377	-0.668266
C	-0.185593	-1.219319	1.556977
C	-1.487650	-2.173582	-0.185300
C	2.980990	0.204393	0.131669
C	2.506525	-1.642079	-1.275494

C	-0.712813	-2.121818	2.469622
F	0.684975	-0.311765	2.001710
C	-2.041586	-3.085084	0.705409
F	-1.905129	-2.220349	-1.454769
C	4.283682	-0.255134	0.299257
F	2.653130	1.318030	0.786468
C	3.796107	-2.131124	-1.123792
F	1.654754	-2.348512	-2.030035
C	-1.647851	-3.058378	2.036927
F	-0.345474	-2.097224	3.745702
F	-2.959496	-3.954552	0.295127
C	4.691510	-1.423963	-0.330605
F	5.138545	0.412097	1.066945
F	4.176012	-3.256267	-1.719233
F	-2.167105	-3.919353	2.899785
F	5.930145	-1.867756	-0.169165
C	0.038162	1.722912	-0.189094
C	-0.853832	2.061232	0.821242
C	0.825075	2.748545	-0.702583
C	-0.951061	3.362139	1.305497
C	0.756058	4.053732	-0.241128
C	-0.145821	4.359812	0.771714
F	-1.658074	1.152689	1.379610
F	-1.812261	3.655580	2.274670
F	-0.235435	5.600693	1.229088
F	1.533952	5.001658	-0.750440
F	1.709641	2.462703	-1.664665
C	-5.071725	-0.528408	-2.677727
H	-5.389813	-0.481380	-3.720781
H	-4.506402	-1.417814	-2.399607
C	-4.127987	-0.372987	-0.013613
H	-3.858021	-1.312906	-0.492693
H	-3.677974	-0.198499	0.965645
As	0.180344	-0.001576	-1.124987

Product

C	-5.561515	-0.133976	-2.006295
C	-5.241789	-0.722728	-0.851539
C	-3.124641	-0.090007	-2.562731
H	-6.023366	-1.133756	-0.207192
H	-6.611424	-0.035491	-2.293791
H	-2.342968	0.474413	-3.092580
H	-3.005347	-1.148701	-2.842823
N	-2.873623	-0.004114	-1.118623
H	-3.055325	0.962380	-0.831560
C	-0.333820	-1.392953	0.246222
C	2.038514	-0.098670	-0.712176
C	0.073997	-1.347255	1.574869
C	-1.023245	-2.520288	-0.177426
C	2.847342	0.713760	0.076436
C	2.670213	-1.143167	-1.383301
C	-0.210417	-2.373649	2.464152
F	0.735275	-0.282882	2.032837
C	-1.332790	-3.560321	0.691254
F	-1.433241	-2.624440	-1.443722
C	4.220879	0.511291	0.171418
F	2.344780	1.722222	0.789789
C	4.035614	-1.377082	-1.305107
F	1.934915	-1.978554	-2.129074
C	-0.919760	-3.484018	2.015212
F	0.176774	-2.306371	3.732909
F	-2.027838	-4.611141	0.268336
C	4.815493	-0.535074	-0.521524
F	4.967119	1.307583	0.929610
F	4.596318	-2.387613	-1.960326
F	-1.206559	-4.467513	2.856096
F	6.122868	-0.734861	-0.429621
C	-0.342225	1.632396	-0.053101
C	-1.230013	1.756210	1.008858
C	0.216215	2.808086	-0.543496
C	-1.537920	2.992243	1.568264
C	-0.066231	4.055878	-0.008938
C	-0.954979	4.144436	1.056396
F	-1.833509	0.688863	1.541001
F	-2.389281	3.076989	2.584948
F	-1.245467	5.325337	1.583225
F	0.497961	5.153257	-0.498896
F	1.088906	2.736025	-1.554540
C	-4.518535	0.402693	-2.951752
H	-4.549901	1.507208	-2.953974
H	-4.751779	0.094443	-3.983224
C	-3.814220	-0.854922	-0.383051
H	-3.494592	-1.906663	-0.491505
H	-3.733519	-0.611501	0.686628
As	0.075315	-0.000695	-1.071473

Sb_cat

R1_cat

C	0.309261	-3.369852	-3.015748
H	0.341630	-4.449479	-3.221794
H	0.745760	-2.698919	-3.767667
N	-0.192569	-2.862760	-1.968345
H	-0.557056	-3.572297	-1.321549
C	1.408576	-1.052597	-0.069459
C	0.341839	1.813450	-0.455699
C	1.262326	-0.948261	1.307867
C	2.511779	-1.747933	-0.542311
C	-0.415575	2.440179	0.526205
C	1.473017	2.495061	-0.887708
C	2.171042	-1.517685	2.190518
F	0.215282	-0.298605	1.823752
C	3.442615	-2.330863	0.309661
F	2.708641	-1.876154	-1.862445
C	-0.075090	3.687386	1.040982
F	-1.511224	1.866351	1.032237
C	1.851076	3.738335	-0.399943
F	2.260793	1.925736	-1.818685
C	3.265929	-2.210076	1.682842
F	2.008324	-1.413905	3.505039
F	4.490885	-2.993945	-0.169615
C	1.060897	4.337089	0.573889
F	-0.821944	4.262299	1.979222
F	2.944448	4.352140	-0.842888
F	4.143350	-2.758232	2.512592
F	1.393938	5.525341	1.061532
C	-1.813522	-0.599088	-0.443894
C	-2.051662	-1.679841	0.391500
C	-2.889993	0.234000	-0.712058
C	-3.303160	-1.923171	0.946307
C	-4.154096	0.028683	-0.179433
C	-4.356555	-1.064371	0.656008
F	-1.070617	-2.541183	0.702437
F	-3.501887	-2.967551	1.745409
F	-5.555348	-1.287821	1.177363
F	-5.159496	0.852854	-0.452430
F	-2.699434	1.302936	-1.499283
Sb	0.038707	-0.132843	-1.479919

R1_cat_R2

C	-5.320389	0.671747	-0.550778
C	-4.702527	0.702386	0.787322
C	-2.684182	1.070883	-3.108091
H	-4.238338	1.644015	1.096671
H	-5.794395	1.600106	-0.887576
H	-3.768148	1.195115	-3.248031
H	-2.042617	1.123093	-3.998098
N	-2.149942	0.863360	-1.978961
H	-2.848064	0.816313	-1.223497
C	-0.876470	-1.129844	-0.121742
C	2.187638	-0.851893	-0.446537
C	-0.741787	-1.036949	1.258101
C	-1.893664	-1.939174	-0.608400
C	2.987622	-0.284313	0.537277
C	2.547018	-2.125021	-0.870861
C	-1.582528	-1.716109	2.129518
F	0.191587	-0.243192	1.789814
C	-2.753345	-2.631049	0.235131
F	-2.094268	-2.053113	-1.927047
C	4.096064	-0.941663	1.062284
F	2.720756	0.926149	1.037083
C	3.641408	-2.818611	-0.372775
F	1.795356	-2.736533	-1.805254
C	-2.591992	-2.516490	1.608681
F	-1.471417	-1.573192	3.446616
F	-3.764110	-3.349363	-0.254598
C	4.422699	-2.211912	0.603202
F	4.843330	-0.370822	2.003045
F	3.944837	-4.038273	-0.807924
F	-3.444133	-3.122029	2.426510
F	5.476402	-2.847169	1.100684
C	0.401613	1.853569	-0.430783
C	-0.603841	2.344048	0.388576
C	1.473309	2.699245	-0.677405
C	-0.551671	3.617194	0.943802
C	1.567057	3.975574	-0.142026
C	0.538995	4.434949	0.674105
F	-1.678598	1.598324	0.690000
F	-1.535496	4.056889	1.725021
F	0.600222	5.652716	1.196375
F	2.612721	4.754866	-0.396330
F	2.477500	2.260950	-1.450913
C	-5.331609	-0.394760	-1.360939
H	-5.845261	-0.363896	-2.323661
H	-4.834850	-1.330441	-1.088594
C	-4.705532	-0.332298	1.634528
H	-5.209164	-1.270399	1.383044
H	-4.223802	-0.266582	2.612336
Sb	0.404681	-0.040662	-1.499231

TS

C	-5.742756	0.475637	-1.547964
C	-5.208214	0.594065	-0.248864
C	-3.172492	0.495634	-2.579053
H	-5.548854	1.410802	0.390470
H	-6.495337	1.202186	-1.866088
H	-3.513458	1.336743	-3.189613
H	-2.809782	-0.382587	-3.122957
N	-2.706954	0.672623	-1.350110
H	-2.911602	1.623619	-1.028135
C	-0.730938	-1.292954	0.263437
C	2.123573	-0.594689	-0.676536
C	-0.318566	-1.234628	1.588172
C	-1.698850	-2.228639	-0.060189
C	3.035030	0.075641	0.129522
C	2.543714	-1.810823	-1.200802
C	-0.850507	-2.071200	2.561323
F	0.594918	-0.336973	1.967392
C	-2.261998	-3.076020	0.885028
F	-2.162934	-2.307320	-1.319425
C	4.305235	-0.429749	0.389376
F	2.727254	1.241723	0.705162
C	3.799342	-2.353478	-0.964631
F	1.693773	-2.517220	-1.968790
C	-1.827577	-2.995634	2.202565
F	-0.448362	-1.995794	3.825245
F	-3.222861	-3.930970	0.546691
C	4.686678	-1.647073	-0.161660
F	5.156363	0.236483	1.164109
F	4.156309	-3.523621	-1.485695
F	-2.351831	-3.793342	3.122716
F	5.894988	-2.137174	0.083965
C	0.056399	1.849916	-0.205048
C	-0.756983	2.192087	0.864601
C	0.913638	2.832517	-0.679857
C	-0.720203	3.455670	1.445074
C	0.982313	4.104089	-0.130685
C	0.152102	4.412986	0.941560
F	-1.616672	1.308055	1.387863
F	-1.511406	3.756105	2.470801
F	0.194042	5.621724	1.485273
F	1.820714	5.017407	-0.607526
F	1.735247	2.536184	-1.697828
C	-5.171264	-0.349013	-2.496496
H	-5.548310	-0.340204	-3.520851
H	-4.660664	-1.264084	-2.195566
C	-4.089303	-0.129949	0.100504
H	-3.899503	-1.095976	-0.361669
H	-3.565310	0.060283	1.038363
Sb	0.072066	-0.029077	-1.300569

Product

C	-5.617061	-0.675929	-1.648938
C	-5.037664	-1.401187	-0.689761
C	-3.333106	-0.120463	-2.500149
H	-5.640618	-2.043416	-0.043078
H	-6.702905	-0.701007	-1.768701
H	-2.735343	0.658304	-2.997736
H	-3.107397	-1.074738	-3.003706
N	-2.874044	-0.255037	-1.109384
H	-3.153742	0.593398	-0.608097
C	-0.233981	-1.557384	0.279540
C	2.080919	0.126398	-0.760710
C	0.138271	-1.350076	1.601832
C	-0.647087	-2.832957	-0.072056
C	2.764314	1.033228	0.039250
C	2.849587	-0.866196	-1.356159
C	0.068625	-2.360798	2.552089
F	0.577032	-0.151956	1.997143
C	-0.733409	-3.869250	0.849472
F	-1.000989	-3.093080	-1.340421
C	4.142775	0.969946	0.221320
F	2.123933	2.013053	0.685712
C	4.224729	-0.969433	-1.200003
F	2.237135	-1.794343	-2.115692
C	-0.371492	-3.624285	2.168764
F	0.417644	-2.140531	3.814788

F	-1.153990	-5.076919	0.486897
C	4.873131	-0.033727	-0.402740
F	4.768103	1.855813	0.991616
F	4.917962	-1.939219	-1.789374
F	-0.444203	-4.597777	3.066045
F	6.187358	-0.103004	-0.232159
C	-0.652380	1.694688	-0.065369
C	-1.483467	1.702075	1.045219
C	-0.173035	2.923443	-0.495615
C	-1.820180	2.874831	1.711537
C	-0.484078	4.117740	0.138285
C	-1.316504	4.086301	1.251674
F	-2.009092	0.559898	1.515089
F	-2.619709	2.850601	2.773309
F	-1.632751	5.213232	1.874609
F	-0.000682	5.275399	-0.297675
F	0.651639	2.962807	-1.551812
C	-4.826141	0.188310	-2.595786
H	-5.012404	1.254158	-2.373439
H	-5.173846	0.032115	-3.628901
C	-3.549648	-1.381180	-0.450053
H	-3.102762	-2.318290	-0.821160
H	-3.327730	-1.325484	0.625975
Sb	-0.083886	-0.036081	-1.259765
